# Transcription factors of *Schizophyllum commune* involved in mushroom formation and modulation of vegetative growth

**DOI:** 10.1038/s41598-017-00483-3

**Published:** 2017-03-22

**Authors:** Jordi F. Pelkmans, Mohini B. Patil, Thies Gehrmann, Marcel J. T. Reinders, Han A. B. Wösten, Luis G. Lugones

**Affiliations:** 1 Microbiology, Department of Biology, Utrecht University, Padualaan 8, 3584 CH Utrecht, The Netherlands; 2 Delft Bioinformatics Lab, Delft University of Technology, Mekelweg 4, 2628 CD Delft, The Netherlands

## Abstract

Mushrooms are the most conspicuous fungal structures. Transcription factors (TFs) Bri1 and Hom1 of the model fungus *Schizophyllum commune* are involved in late stages of mushroom development, while Wc-2, Hom2, and Fst4 function early in development. Here, it is shown that Bri1 and Hom1 also stimulate vegetative growth, while biomass formation is repressed by Wc-2, Hom2, and Fst4. The Δ*bri1*Δ*bri1* and the Δ*hom1*Δ*hom1* strains formed up to 0.6 fold less biomass when compared to wild-type, while Δ*wc-2*Δ*wc-2*, Δ*hom2*Δ*hom2*, and Δ*fst4*Δ*fst4* strains formed up to 2.8 fold more biomass. Inactivation of TF gene *tea1*, which was downregulated in the Δ*wc-2*Δ*wc-2*, Δ*hom2*Δ*hom2*, and Δ*fst4*Δ*fst4* strains, resulted in a strain that was severely affected in mushroom development and that produced 1.3 fold more biomass than the wild-type. In contrast, introducing a constitutive active version of *hom2* that had 4 predicted phosphorylation motifs eliminated resulted in radial growth inhibition and prompt fructification in both Δ*hom2* and wild-type strains, even in sterile monokaryons. Together, it is concluded that TFs involved in mushroom formation also modulate vegetative growth. Among these TFs is the homeodomain protein Hom2, being the first time that this class of regulatory proteins is implicated in repression of vegetative growth in a eukaryote.

## Introduction

Mushroom forming fungi degrade plant waste, establish mutual beneficial interactions with plants (mycorrhiza), and can be pathogens. Moreover, they are an important food source and produce bioactive components. Mushrooms are the most conspicuous fungal structures. Their formation involves a complex developmental program that has been studied in the model systems *Coprinopsis cinerea* and *Schizophyllum commune* ^1, 2^. Germination of a basidiospore of *S. commune* results in a monokaryotic mycelium that can fuse with another monokaryon with compatible mating types. Blue light is required to initiate fruiting in the resulting dikaryotic mycelium^3^, whereas high CO_2_ levels repress this developmental program^4, 5^. Initiation of mushroom formation starts with asymmetrical colony growth, followed by aggregation of aerial hyphae, and subsequent formation of primordia^2^. Primordia develop into fruiting bodies that form basidia within the hymenium. Karyogamy, meiosis, and one round of mitosis in the basidia results in haploid, binucleate basidiospores.

The blue light receptor complex of *S. commune* consists of Wc-1 that has a blue light sensing domain and the transcription factor Wc-2. Inactivation of *wc-1* and/or *wc-2* results in a blind phenotype^6^. Dikaryotic colonies of the homozygous deletion strains grow symmetrically in blue light (similar to dark-grown wild-type dikaryons) and do not produce aggregates, primordia, and fruiting bodies. Deletion of the homeodomain gene *hom2* and the DNA binding Bright domain protein gene *bri1* shows a similar phenotype^7^. Inactivation of the zinc finger transcription factor gene *fst4* results in dikaryons that still grow irregular in the light under low CO_2_ conditions but aggregates, primordia, and fruiting bodies are not produced^8^. Strains in which the Cys2His2 zinc finger protein gene *c2h2* has been inactivated are arrested at the aggregate stage^7^, while deletion strains of *fst3*, *gat1*and *hom1* form smaller fruiting bodies but in higher numbers^7, 8^. The zinc finger protein Fst3 was proposed to play a role in repression of outgrowth of fruiting bodies from primordia, while the GATA type zinc finger protein Gat1 and the homeodomain protein Hom1 have been proposed to play a role in expansion of the fruiting body. Homologues of the *S. commune* transcription factors have been identified in *Agaricus bisporus*, *Laccaria bicolor* and *C. cinerea*. Expression studies suggest the existence of a core regulatory program for fruiting body development in these and other basidiomycetes^8–10^, which is supported by the finding that over-expression of the *c2h2* homologue of *A. bisporus* results in faster fruiting body development^11^.

In this study, it is shown that transcription factors that are involved in mushroom formation in *S. commune* also modulate vegetative growth. Bri1 and Hom1 were shown to stimulate biomass formation, while Wc-1, Wc-2, Hom2, Fst4, as well as the newly identified transcription factor Tea1 repress vegetative growth. The interplay between vegetative growth and mushroom formation is illustrated by a constitutive version of Hom2 that strongly reduces colony formation and immediately induces mushroom formation under ambient CO_2_ conditions, even in a sterile strain.

## Results

### Genes involved in mushroom development also control vegetative growth

**Figure 1:**
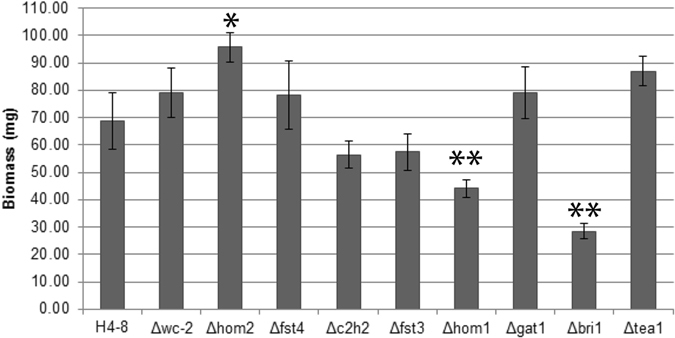
Biomass of biological triplicates of 6-day-old dark-grown agar cultures of the wild-type dikaryon and transcription factor deletion strains using glucose as carbon source. An independent sample t-test (p-value ≤ 0.05) was used to identify strains forming more (*) or less (**) biomass when compared to wild-type H4-8 (lines 506-507).

Dikaryotic colonies of wild-type H4-8, and the transcription deletion strains Δ*wc-2*Δ*wc-2*, Δ*hom2*Δ*hom2*, Δ*bri1*Δ*bri1*Δ*fst4*Δ*fst4*, Δ*c2h2*Δ*c2h2*, Δ*fst3*Δ*fst3*, Δ*hom1*Δ*hom1*, and Δ*gat1*Δ*gat1* were grown for 6 days on glucose MM in the dark. The Δ*hom2*Δ*hom2* strain formed 1.4-fold more biomass when compared to H4-8 (Fig. 1), which was accompanied by increased radial growth (not shown). In contrast, Δ*bri1*Δ*bri1* and Δ*hom1*Δ*hom1* formed 0.4 and 0.6-fold less biomass, respectively, while biomass formation of the other strains was not significantly different from H4-8. Strain Δ*hom2*Δ*hom2*Δ*fst4*Δ*fst4* also formed more biomass than the wild-type. There was a trend that it also formed more biomass when compared to Δ*hom2*Δ*hom2* (Supplementary Fig. 1), which was confirmed in liquid shaken cultures where it formed 1.7 fold more biomass when compared to the *hom2* deletion strain (Supplementary Fig. 1). These data show that *bri1* and *hom1* stimulate vegetative growth, while *hom2* and *fst4* repress biomass formation of dikaryotic strains when glucose is used as a carbon source. Microscopical analysis revealed that wild-type formed two types of hyphae at the colony periphery. Vacuole-rich hyphae with a diameter of 2.68 µm ± 0.14 were 5,7-fold less abundant than hyphae with few vacuoles and a diameter of 1.72 µm ± 0.12. Strain Δ*hom1*Δ*hom1* formed 5-fold less thick hyphae when compared to the wild-type, while Δ*bri1*Δ*bri1*, Δ*fst4*Δ*fst4*Δ*hom2*Δ*hom2* formed 1.3-, 2.0-, and 3.3-fold more vacuole-rich, wide hyphae (Supplementary Fig. 2).

Biomass formation of Δ*wc-2*Δ*wc-2*, Δ*hom2*Δ*hom2*and Δ*fst4*Δ*fst4* was assessed on xylose, pectin, and sucrose (Supplementary Fig. 3). The Δ*fst4*Δ*fst4* strain formed 2.8-fold more biomass on xylose when compared to H4-8, while Δ*wc-2*Δ*wc-2*, Δ*hom2*Δ*hom2*and Δ*fst4*Δ*fst4* formed more biomass on sucrose (1.2, 1.4, and 1.6-fold, respectively) and pectin (1.3, 1.4, and 1.6-fold, respectively) (Supplementary Fig. 3). Together, these data show that Wc-2, Hom2, and Fst4 repress vegetative growth on different carbon sources.

**Figure 2:**
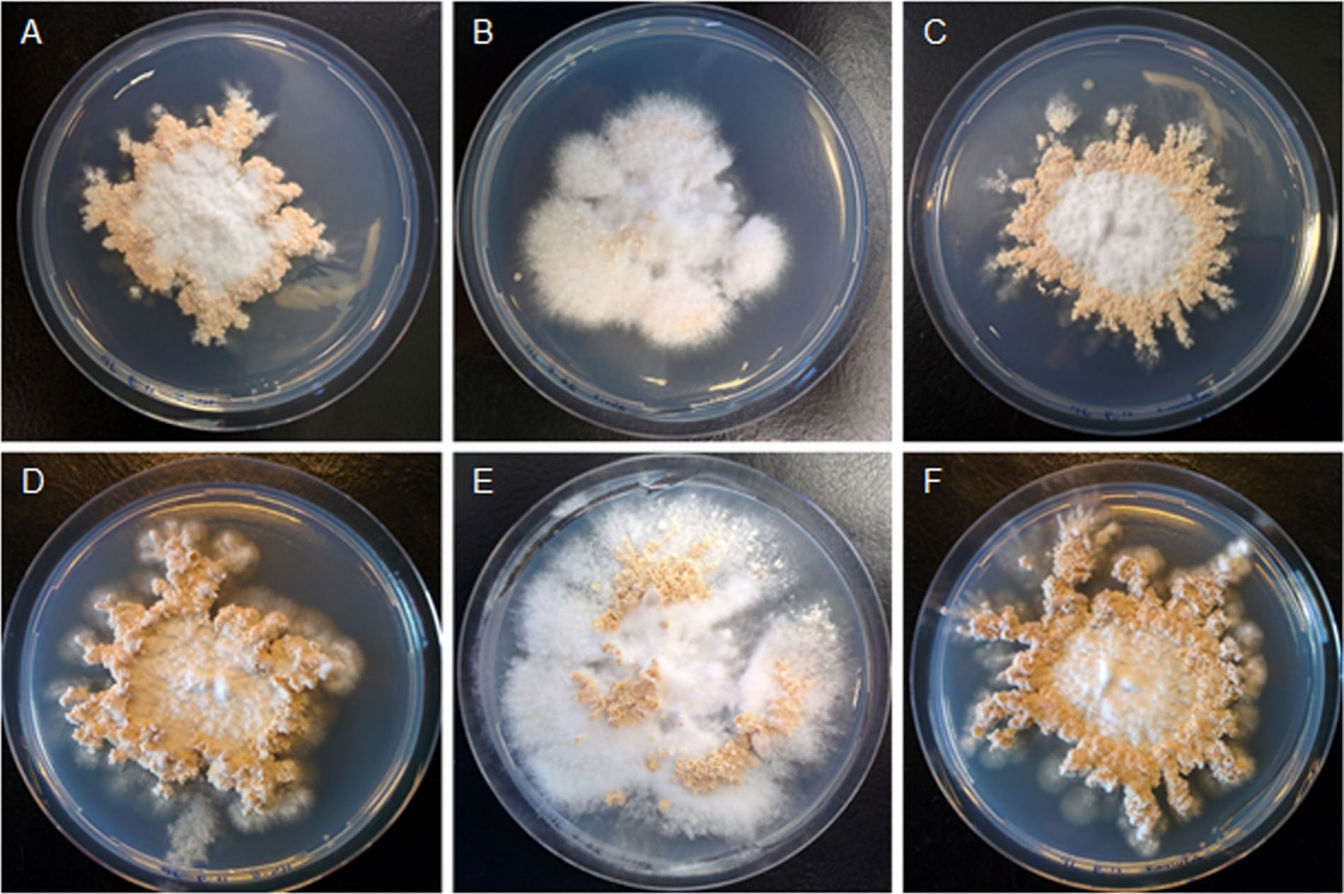
Fruiting body development of the wild-type dikaryon (**A**,**D**), Δ*bri1*Δ*bri1* (**B**,**E**), and Δ*hom1*Δ*hom1* (**C**,**F**) after 7 (**A–C**) and 15 (**D–F**) days of growth.

Reduced vegetative growth of the Δ*bri1*Δ*bri1* and Δ*hom1*Δ*hom1* strains may slow down fruiting body development. Therefore, fruiting was monitored after 7 days and 15 days of culturing. The Δ*hom1*Δ*hom1* strain had formed more but smaller mushrooms both after 7 and 15 days (Fig. 2). In contrast, Δ*bri1*Δ*bri1* had not formed fruiting bodies after 7 days^7^ but did so after 15 days showing that fruiting in this strain is delayed and not abolished as reported previously^7^.

### Genome-wide expression analysis

RNA composition of the wild-type H4-8 dikaryon was determined during vegetative growth in the dark, after transfer to the light, and during aggregate, primordium, and fruiting body formation. Expression of *wc-1*, *wc-2*, *hom2*, *fst4*, *fst3*, *gat1*, and *bri1* in whole cultures increased or decreased ≤2-fold during development when compared to the vegetative mycelium grown in the dark (Table 1). In contrast, *c2h2* and *hom1* expression increased gradually with a maximum fold change of 4.7 and 2, respectively, during the fruiting body stage.

**Table 1:** Temporal expression of the blue light sensor gene *wc-1* and transcription factor genes involved in fruiting body development in the wild-type dikaryon based on biological duplicates. Values are expressed in FKPM. Bold font indicates a significant ≥2-fold up-regulation when compared to dark grown vegetative mycelium.

	Vegetative Mycelium Dark	Vegetative mycelium Light (induced)	Aggregate	Primordia	Fruiting body
*wc-1*	37.30	40.06	54.83	51.97	62.23
*wc-2*	38.29	46.19	39.88	61.66	47.47
*hom2*	107.64	135.84	115.99	104.15	58.63
*tea1*	9.39	14.73	**18.88**	**62.40**	10.48
*fst4*	112.88	159.43	140.70	178.65	119.08
*c2h2*	22.06	29.61	**47.48**	**87.84**	**105.36**
*fst3*	103.23	97.82	102.70	119.53	115.64
*gat1*	75.01	65.00	72.79	61.87	126.09
*hom1*	114.38	108.69	149.72	200.48	**234.15**
*bri1*	30.03	34.22	30.56	34.99	29.95
*c2h2d*	3.72	**8.76**	**16.57**	**39.17**	**54.94**

Expression profiles of wild type, Δ*wc-1*Δ*wc-1*, Δ*wc-2*Δ*wc-2*, Δ*hom2*Δ*hom2*, Δ*fst4*Δ*fst4*, Δ*c2h2*Δ*c2h2*, Δ*fst3*Δ*fst3*, Δ*gat1*Δ*gat1*, Δ*hom1*Δ*hom1*, and Δ*bri1*Δ*bri1* strains were compared at the moment the wild-type formed aggregates (day 8) and fruiting bodies (day 12). Principal component analysis of the RNA profiles of 8-day-old colonies revealed a first and second component explaining 38% and 29% of the variation, respectively. Two distinct clusters were observed (Supplementary Fig. 4). The first cluster consisted of Δ*wc-1*Δ*wc-1*, Δ*wc-2*Δ*wc-2*, and Δ*hom2*Δ*hom2* that are all affected in early stages of fruiting body development. The second cluster consisted of Δ*gat1*Δ*gat1* and Δ*fst3*Δ*fst3* that are affected in late stages of development. The other deletion strains did not cluster but rather showed a gradual change in expression. Principal component analysis of the RNA profiles of 12-day-old colonies revealed a first and second component explaining 72% and 7% of the variation, respectively. In this case, Δ*wc-1*Δ*wc-1*, Δ*wc-2*Δ*wc-2*, Δ*hom2*Δ*hom2*and Δ*fst4*Δ*fst4* clustered, whereas the other strains clustered with the wild-type (Supplementary Fig. 4).

The number of up- and down-regulated genes were between 86 and 1392 and 131 and 1463, respectively, when expression of the 8- and 12-day-old cultures of the deletion strains was compared with wild type (Supplementary Table 1). Enriched functional categories in the upregulated genes of 8-day-old colonies of Δ*wc-1*Δ*wc-1*, Δ*wc-2*Δ*wc-2*, Δ*hom2*Δ*hom2*and Δ*fst4*Δ*fst4* were mainly linked to carbohydrate metabolism, when compared to the 8-day-old wild-type colonies (Supplementary Tables 2 & 3). Downregulated functional groups were linked to energy transfer in these strains. Functional groups involved in energy transfer and carbohydrate metabolism were upregulated in 12-day-old colonies of Δ*wc-1*Δ*wc-1* and Δ*wc-2*Δ*wc-2* (Supplementary Tables 3 & 4), while downregulated genes were enriched for nucleosome, catalytic and oxidoreductase activity. The functional group ATPase activity was also overrepresented within the downregulated genes of Δ*wc-2*Δ*wc-2*. In 12-day-old colonies of Δ*hom2*Δ*hom2* functional groups involved in translation, energy transfer, and carbohydrate metabolism were overrepresented in the upregulated genes, while functional groups involved in transport and energy transfer were overrepresented in the downregulated genes. In 12-days-old colonies of Δ*fst4*Δ*fst4* functional groups involved in carbohydrate processes and energy transfer were enriched in the upregulated genes. Groups involved in oxidoreductase activity, metabolic process and nucleosome, amongst others, were enriched in the downregulated genes. Functional categories involved in energy transfer and carbohydrate metabolism were upregulated in 8-day-old colonies of Δ*c2h2*Δ*c2h2*, while processes involved in cytoplasm, ATP binding, nucleosome and peptidase activity were enriched in the downregulated genes. In 12-day-old colonies of Δ*c2h2*Δ*c2h2* upregulated genes were enriched in functional groups related to energy transfer, carbohydrate metabolic process, and chitin catabolic process. Downregulated genes were enriched in groups involved in cell wall, catalytic activity, tryptophan and fatty acid synthesis. No functional groups were overrepresented in the upregulated genes in 8-day-old and 12-day-old colonies of Δ*hom1*Δ*hom1* but functional groups related to chitinase activity, carbohydrate metabolism and energy transfer were enriched in the downregulated genes. 12-day-old Δ*hom1*Δ*hom1* colonies showed overrepresentation of functional groups related to amino acid synthesis, oxidoreductase, and carbohydrate metabolism activity in the down regulated genes. The functional categories that were enriched in the upregulated genes of 8-day-old colonies of Δ*fst3*Δ*fst3* and Δ*gat1*Δ*gat1* were mainly involved in energy transfer, tryptophan metabolism, and cell wall processes. Genes involved in energy transfer and carbohydrate metabolism were enriched in the down-regulated genes. Groups involved in carbohydrate metabolism, methyltransferase, and leucine synthesis were enriched in the upregulated genes in 12-days-old colonies of Δ*fst3*Δ*fst3*. Downregulated genes were mainly enriched in cell wall processes and chitinase activity. Functional groups were upregulated for sphingolipid metabolism in 12-days-old colonies of Δ*gat1*Δ*gat1*. Downregulated genes were mainly enriched for translation and cell wall. No functional groups were overrepresented in the upregulated genes of 8-day-old and 12-day-old colonies of the Δ*bri1*Δ*bri1* strain. In contrast, functional groups related to metabolic process, carbohydrate metabolism, transcription and cell wall were overrepresented in the downregulated genes of 8-day-old colonies. Downregulated genes in 12-day-old Δ*bri1*Δ*bri1* colonies were mainly enriched for carbohydrate metabolism, hydrolase activity and transcription repressor activity.

**Figure 3:**
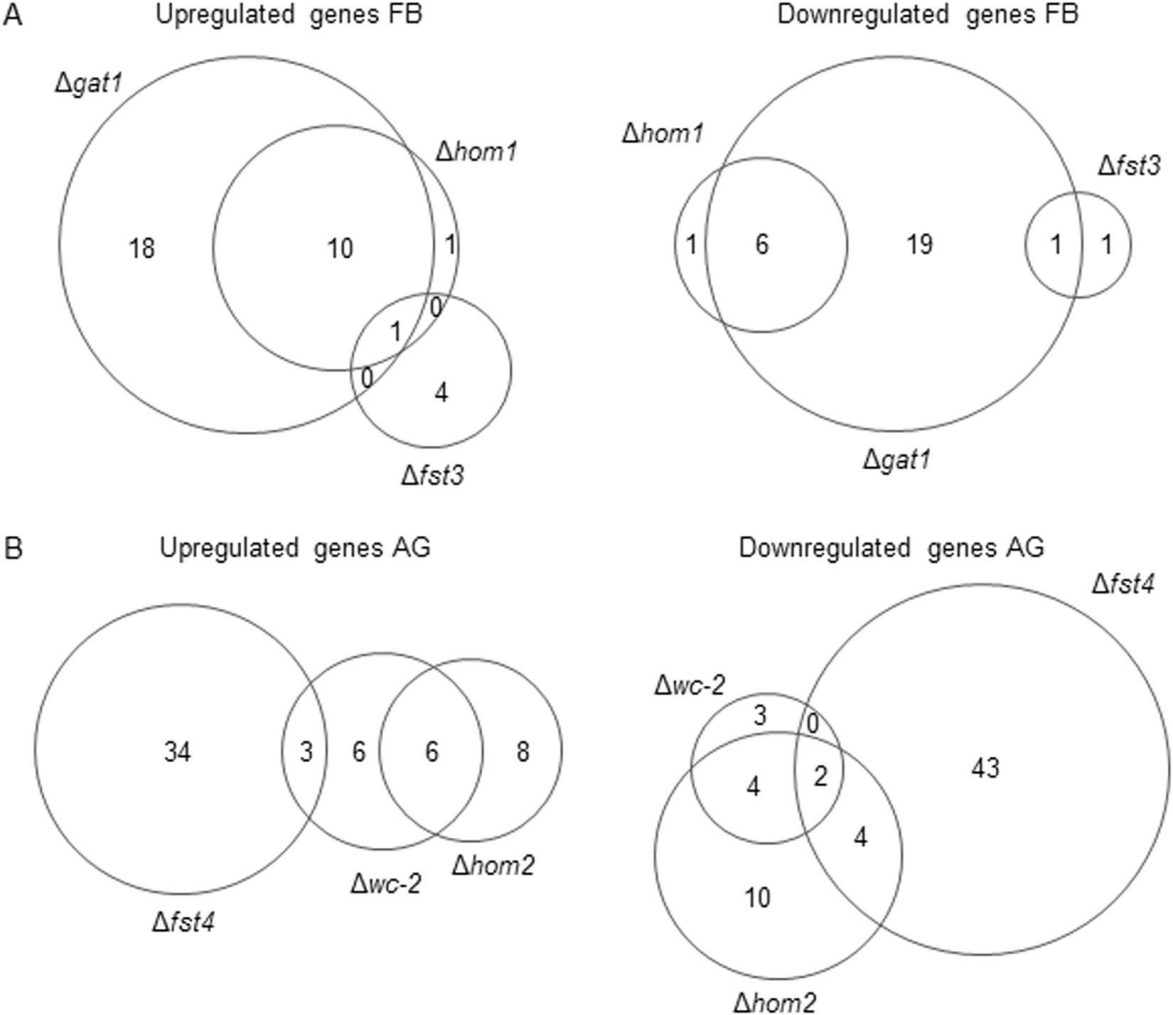
Venn diagrams showing overlapping differentially expressed transcription factor genes in Δ*fst3*Δ*fst3*Δ*hom1*Δ*hom1*and Δ*gat1*Δ*gat1* (**A**) and Δ*wc-2*Δ*wc-2*, Δ*hom2*Δ*hom2*, and Δ*fst4*Δ*fst4* (**B**) when compared to the wild-type aggregate and fruiting body stage, respectively.

Between 1 and 43 transcription factor genes were ≥2-fold up- or down-regulated in one of the deletion strains when compared to the wild-type (Fig. 3; Supplementary Table 5). Expression of *hom1* and *c2h2* had decreased in 12-day-old Δ*wc-1*Δ*wc-1* colonies. In 12-day-old colonies of Δ*wc-2*Δ*wc-2 hom2* expression was increased, while *c2h2* was downregulated. Expression of *c2h2*, *gat1*, and *hom1* was downregulated in 12-day-old Δ*hom2*Δ*hom2* colonies. In 8-day-old Δ*fst4*Δ*fst4* colonies expression of *hom1* was downregulated and in 12-day-old colonies *hom2* expression increased, while *c2h2* and *hom1* expression had decreased. In contrast, *hom1* levels were upregulated in 8-day-old Δ*fst3*Δ*fst3* colonies. Similarly, expression of *hom1* but also *c2h2* was increased in 8-day-old colonies of Δ*gat1*Δ*gat1*. In 8-day-old Δ*bri1*Δ*bri1* colonies *gat1* expression was increased, while *hom2* was increased in 12-day-old colonies (Supplementary Fig. 5). Together, these data show that transcription factors involved in fruiting control other regulatory genes involved in this process.

### Gene tea1 is involved in fruiting body development and represses vegetative growth

Transcription factor gene *tea1* (protein ID 2519514) showed ≥2-fold decreased expression in 8-day-old colonies of Δ*wc-1*Δ*wc-1*, Δ*wc-2*Δ*wc-2*, Δ*hom2*Δ*hom2*, Δ*bri1*Δ*bri1*and Δ*fst4*Δ*fst4* when compared to the 8-day-old aggregate forming colonies of wild-type, while it was upregulated ≥2-fold in 8-day-old colonies of Δ*fst3*Δ*fst3*, Δ*hom1*Δ*hom1*and Δ*gat1*Δ*gat1*. A ≥ 2-fold decreased or increased expression of *tea1* was not observed in the deletion strains at day 12 when the wild-type had formed fruiting bodies. This is explained by reduced expression of *tea1* in the wild-type colonies when compared to day 8. Gene *c2h2d* (proteinID 2703923) was also downregulated in Δ*wc-1*Δ*wc-1*, Δ*wc-2*Δ*wc-2*, Δ*hom2*Δ*hom2*and Δ*fst4*Δ*fst4* during aggregation, while it was upregulated ≥2-fold in Δ*gat1*Δ*gat1* (Supplementary Fig. 5). Furthermore, expression of *c2h2d* was decreased >2-fold in 12-day-old colonies of Δ*wc-1*Δ*wc-1*, Δ*wc-2*Δ*wc-2*, Δ*hom2*Δ*hom2*, Δ*fst4*Δ*fst4*, Δ*fst3*Δ*fst3*and Δ*gat1*Δ*gat1*. Gene *c2h2d* has a predicted C2H2 DNA binding domain, while *tea1* is a predicted TEA/ATTS transcription factor. Expression of *tea1* and *c2h2* in the wild-type strain peaked during primordia and fruiting body formation, respectively (Table 1).

**Figure 4:**
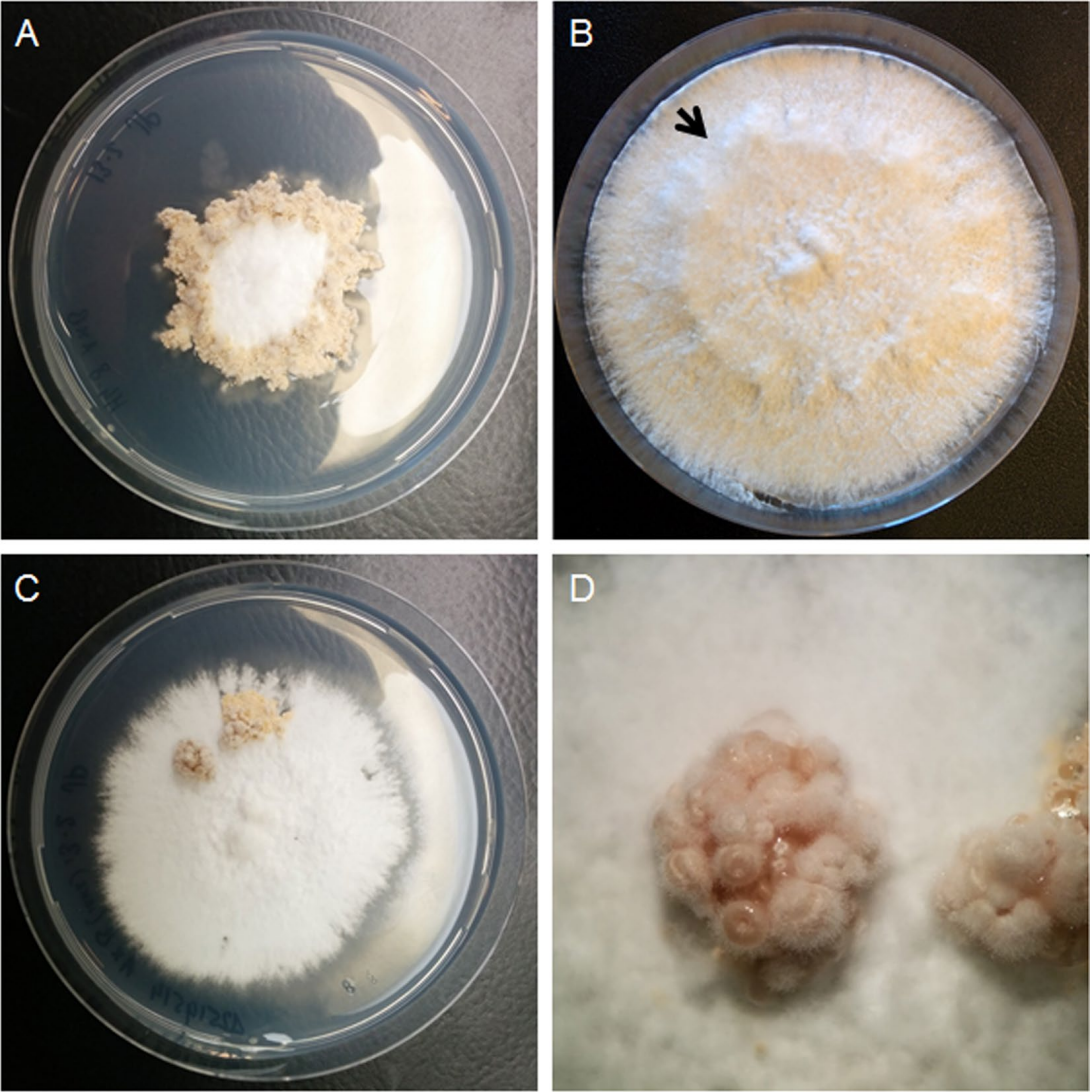
Aerial growth of dikaryotic colonies of H4-8 (**A**) and Δ*tea1* (**B**,**C**,**D**). Strain Δ*tea1*Δ*tea1* produced less dense and high aerial hyphae when transferred to light (**B**) and only forms small clusters of fruiting bodies (**C** and **D**). Arrow indicates the transition from dense aerial hyphae production to thinner aerial hyphae production upon light induction.

Deletion constructs of *c2h2d* and *tea1* were introduced in H4-8Δ*ku80*. PCR analysis confirmed that Δ*c2h2d* and Δ*tea1* strains were obtained. These transformants were crossed with the compatible H4-8 wild-type strain and siblings were selected with an intact *ku80* gene and a deleted *tea1* or *c2h2d* gene. Compatible monokaryons were identified and then crossed to obtain the Δ*tea1*Δ*tea1* and Δ*c2h2d*Δ*c2h2d* strains. Growth and mushroom formation of the Δ*c2h2d*Δ*c2h2d* strain were not affected (data not shown). In contrast, the Δ*tea1*Δ*tea1* strain showed a 1.3-fold increase in biomass when compared to the wild-type. This increase was similar to that of Δ*hom2*Δ*hom2* (Fig. 1). Transfer to the light did not induce irregular vegetative growth as observed in the wild-type. Moreover, newly formed light-exposed mycelium did not produce aerial hyphae (Fig. 4B). This resulted in a distinct border between the dark-grown mycelium and mycelium grown in the light. Fruiting body formation was almost completely abolished. The few spore-forming flask-like mushrooms were formed in clusters at random positions in the Δ*tea1*Δ*tea1* colony, while a typical ring of fruiting bodies is formed in the wild-type (Fig. 4C,D). This and the fact that fruiting body and biomass formation was restored to wild-type levels in 2 independent complemented strains (data not shown) shows that *tea1* represses vegetative growth and is involved in fruiting body formation.

### Hom2^con^ represses vegetative growth and induces fruiting body formation

**Figure 5:**
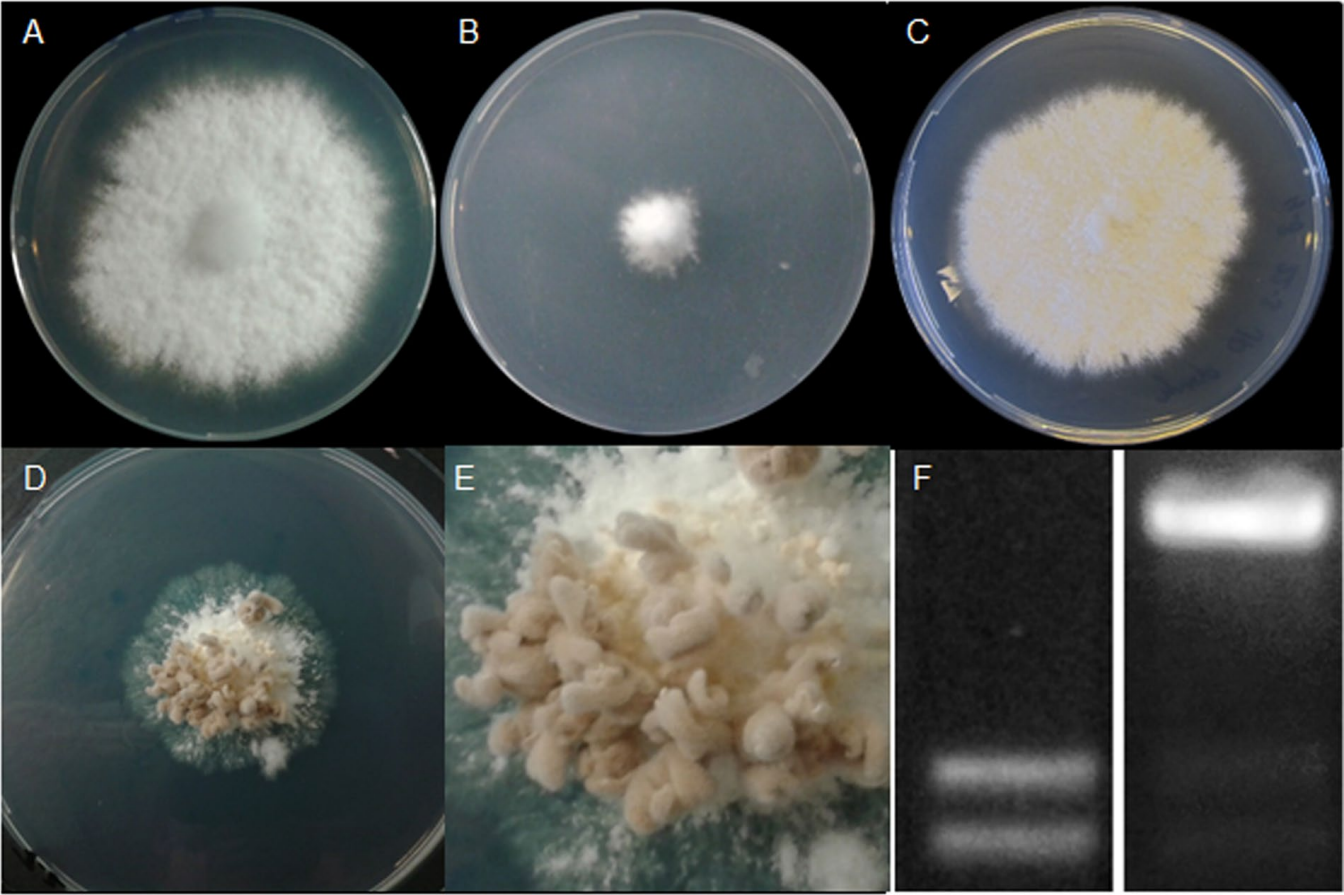
Six-day-old colonies of the wild type monokaryon (**A**,**C**) and a *hom2* ^*con*^ strain (**B**,**D**) grown continuously at 30 °C in the dark at high CO_2_ (**A,B**) or transferred to 25 °C, low CO_2_, and light (**C**,**D**). (**E**) represents a close up of fruiting bodies of the *hom2* ^*con*^ strain, while (**F**) shows relative *hom2* and *hom2* ^*con*^ expression in wild-type (left) and the *hom2* ^*con*^ strain T4 (right).

Hom2 is a key gene involved in mushroom formation but it does not show differential expression during development (see above). Therefore, we searched for motifs that could mediate post-translational modification of this homeodomain protein. Hom2 was found to contain 4 predicted RRXS phosphorylation motifs. The serine codons within these motifs were replaced for alanine codons, resulting in *hom2* ^*con*^ (name based on its phenotype; see below) and introduced in *S. commune* H4-8a and H4-8b. Two types of transformants were observed. 90% of the colonies had a growth speed similar to regenerating wild-type protoplasts, while 10% grew very slowly. Restriction PCR using RNA from colonies of the wild-type, normal growing transformants, and slow groing transformants showed that *hom2* ^*con*^ was much higher expressed than the endogenous copy in the case of slow growing transformants (Fig. 5F). These transformants were selected for further experiments. Monokaryotic *hom2* ^*con*^ transformants grew slower than wild type at 30 °C in the dark at high CO_2_. Clamp connections as observed in the wild-type dikaryon were absent (Fig. 5A,B). When colonies were transferred to fruiting conditions (low CO_2_, light, 25 °C) the monokaryotic *hom2* ^*con*^ transformants started to form flask like fruiting bodies that did not expose their spore forming gills (Fig. 5D,E). Similar results were obtained when *hom2* ^*con*^ was introduced in a Δ*hom2* monokaryon. These data show that Hom2^con^ functions independently of the endogenous Hom2 and that it can induce fruiting in a sterile monokaryon. Monokaryotic *hom2* ^*con*^ transformants crossed with the compatible wild type strain showed less severe phenotypes. However, they all grew slower than the wild type dikaryon and developed spore forming fruiting bodies immediately after transfer to the light at low CO_2_ conditions. In contrast, the wild-type continued vegetative growth for 1-2 days and then started to form fruiting bodies (Data not shown).

## Discussion

The transcription factor genes *wc-2*, *hom2*, *fst4*, *bri1c2h2*, *fst3*, *gat1*, and *hom1* have been reported to be involved in fruiting body formation in *S. commune* ^6–8^. We here showed that the homeodomain domain protein Hom2, the zinc finger transcription factor Fst4, and the blue light complex transcription factor Wc-2 also repress vegetative growth, while the DNA binding BRIGHT domain protein Bri1 and the homeodomain protein Hom1 stimulate vegetative growth. We also showed that a newly identified transcription factor, called Tea1, stimulates mushroom formation, while repressing vegetative growth.

**Figure 6:**
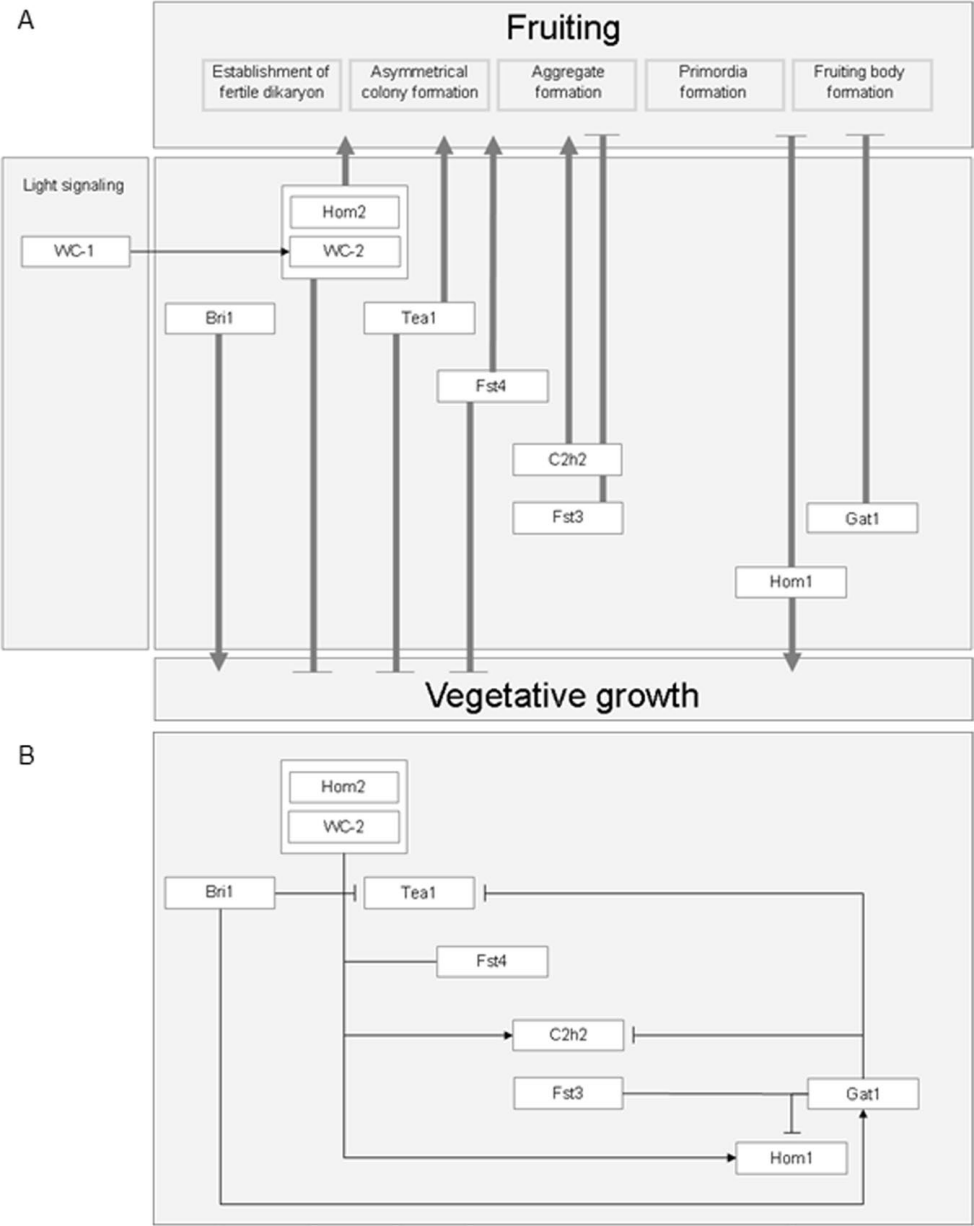
Model of regulation of vegetative growth and fruiting body formation in *S. commune*. Transcription factor genes control both vegetative growth and fruiting body development (**A**) and influence each other's expression levels (**B**).

The Δ*bri1*Δ*bri1* strain does not fruit after the standard growth period of 10 days^7^. However, we here showed that fully developed mushrooms had formed after 4 weeks. This shows that Bri1 is not required for fruiting. Delayed mushroom development may be the result of the reduced growth speed of Δ*bri1*Δ*bri1*. Lower biomass formation may well be explained by the fact that functional categories metabolic process, carbohydrate metabolism, catalytic activity, transcription, and cell wall were down-regulated in 8-day-old colonies of the deletion strain. 12-day-old colonies showed down-regulation of functional categories carbohydrate metabolism, hydrolase activity, and transcription repressor activity. As a consequence of reduced biomass formation, a quorum sensing pathway may become activated at a later moment delaying the switch to fruiting body formation^12^. Notably, Bri1 deletion has an effect on expression of *tea1* and of *gat1*. The repression of *tea1* may be a direct or an indirect effect due to its stimulatory effect on *gat1* expression. Together, Bri1 stimulates vegetative growth and functions in mushroom formation by its effect on *tea1* and *gat1* expression (Fig. 6).

Genes *hom2* and *wc-2* are involved in the switch from vegetative growth to fruiting. Inactivation of these genes abolishes early stages of fruiting body formation^6, 7^ but also increased the vegetative growth rate. Strain Δ*wc-2*Δ*wc-2* formed more biomass on sucrose and pectin when compared to the wild-type, while Δ*hom2*Δ*hom2* formed more biomass on glucose, sucrose, and pectin. Increased biomass formation was associated with an increased hyphal diameter and with a higher radial extension rate. In addition, upregulated genes were enriched in the functional class carbohydrate metabolism in 8-day-old colonies, and of functional groups involved in carbohydrate metabolism and energy transfer in 12-day old colonies. Deletion of *wc-2* and *hom2* resulted in a ≥2-fold downregulation of *c2h2* in 12-day-old colonies. Downregulation was also observed in 8-day-old colonies, although the effect was less pronounced. Together, this confirms that Wc-2 and Hom2 stimulate *c2h2* expression^6, 7^(Fig. 6).

Expression of *hom2* and *wc-2* is rather constant during development, suggesting post-translational regulation of these genes. In the case of Wc-2, this may be accomplished by the interaction with the blue light sensor Wc-1^6^, while post-translational modification of Hom2 was shown to be mediated via its 4 predicted protein kinase motifs. Introduction of *hom2* ^*con*^ that has its 4 protein kinase motifs eliminated resulted in severe phenotypes in sterile wild-type and Δ*hom2* monokaryons. The *hom2* ^*con*^ strains grew very slowly when compared to their parental strains, indicating that *hom2* in its non-phosphorylated state represses vegetative growth. Transformants even fruited when exposed to light and low CO_2_, thus resembling a dikaryon. This suggests that Hom2 is maintained in a phosphorylated state in the monokaryon resulting in fast vegetative growth and repression of fruiting. The fact that the Δ*hom2* monokaryon grows faster than the wild-type implies that a fraction of Hom2 in the monokaryon is not phosphorylated thus impacting vegetative growth to some extent. Data also indicate that the phosphorylation state of Hom2 is the only inhibitory factor that suppresses fruiting in monokaryons in conditions favorable for mushroom formation in the dikaryon. Moreover, data imply that fruiting body formation can be independent of the mating type genes. Indeed, *frt1* of *S. commune* also initiates fruiting in a homokaryon when copies of the gene are introduced in homokaryons with a different *frt1* allele^13^. The role of the mating type genes could be restricted to the establishment and maintenance of the dikaryotic state. Alternatively, different pathways exist that each can lead to fruiting body formation and that may at some point interconnect.

Gene *fst4* is constitutively expressed during the *S. commune* life cycle. Like Hom2 and Wc-2 it is involved in the switch from vegetative growth to fruiting. Strain Δ*fst4*Δ*fst4* grows irregular in the light like the wild-type but does not aggregate. It formed more biomass than the wild-type on xylose, sucrose, and pectin but not on glucose. Hyphae of Δ*fst4*Δ*fst4* were more wide compared to wild-type but not as wide as Δ*hom2*Δ*hom2*. Together, these data indicate that Fst4 and Hom2 represent different parts of the repression pathway of vegetative growth. In liquid shaken cultures with glucose as carbon source these pathways may merge explaining why Δ*hom2*Δ*hom2*Δ*fst4*Δ*fst4* formed more biomass than Δ*hom2*Δ*hom2*. Strain Δ*fst4*Δ*fst4* showed enrichment of carbohydrate metabolism in the upregulated genes of 8-day-old and 12-day-old-colonies similar to that observed in Δ*wc-2*Δ*wc-2* and Δ*hom2*Δ*hom2*. The fact that *fst4* expression is not affected in Δ*wc-2*Δ*wc-2* and Δ*hom2*Δ*hom2* strengthens the hypothesis that Fst4 and Hom2 represent different pathways. Gene *fst4* stimulates *c2h2* like *hom2* and *wc-2* do. This indicates that Fst4, Hom2, and Wc-2 input are channeled into the fruiting pathway via *c2h2* (Fig. 6).

Transcription factor gene *tea1* was downregulated in Δ*wc-1*Δ*wc-1*, Δ*wc-2*Δ*wc-2*, Δ*hom2*Δ*hom2*, Δ*bri1*Δ*bri1*and Δ*fst4*Δ*fst4* when compared to the aggregating wild-type (8-day-old colonies), while it was upregulated in Δ*fst3*Δ*fst3*, Δ*hom1*Δ*hom1*and Δ*gat1*Δ*gat1*. This indicates it is upregulated during early stages of development while it is repressed during late stages of mushroom formation. This agrees with the expression profile in the wild-type. The Δ*tea1*Δ*tea1* strain formed more biomass on glucose when compared to the wild-type. Moreover, it was severely affected in mushroom formation. Local clusters of fully developed mushrooms were only occasionally formed in the deletion strain. This phenotype may be explained by a reduced sensitivity of a signaling pathway leading to a developmental switch from “off” to “on”.

Expression of *c2h2* increased > 2-fold when RNA profiles of 5- and 8-day-old colonies were compared. The increased expression during the aggregation stage and the further increased expression in primordia and fruiting bodies agrees with the phenotype of Δ*c2h2*Δ*c2h2* forming aggregates but not primordia and fruiting bodies^7^ (Fig. 6). C2H2 did not affect biomass formation implying that it is downstream of the switch between vegetative growth and mushroom development.

Deletion of *fst3*, *gat1*, or *hom1* results in more, but smaller mushrooms^7^. In addition, Hom1 and Gat1 are involved in mushroom tissue formation^7^. Gene *fst3* is constitutively expressed and its expression is not affected by any of the other transcriptional regulators. This suggests that Fst3 is subject to post-transcriptional regulation. The fact that a higher number of genes are differentially expressed in 8-day-old colonies (wild-type forming aggregates) when compared to 12-day-old colonies (wild-type forming fruiting bodies) (i.e. 882 and 217 genes, respectively) suggest that Fst3 exerts its effect already early in mushroom development. Upregulated functional groups were involved in energy transfer and cell wall processes in 8-day-old colonies, while downregulated functional groups were involved in carbohydrate metabolism. In 12-day-old colonies these groups were regulated in the opposite direction. Expression of *gat1* was highest during fruiting body formation. It is repressed by Bri1 in 8-day-old colonies, while it is activated by Hom2 during fruiting body formation. Expression of *hom1* gradually increases during progression of fruiting. Wc-1, Hom2, Fst4, and probably Wc-2, stimulate expression of *hom1*, while Fst3 and Gat1 have the opposite effect. The fact that Hom1 has both an effect on vegetative growth and on tissue formation in mushrooms suggests that Hom1 operates at two distinct stages of development (Fig. 6). Alternatively, the reduced size of the Δ*hom1*Δ*hom1* fruiting bodies and the effect of Hom1 on tissue formation in mushrooms may be explained solely by the reduced size of the vegetative feeding mycelium.

The model of development of *S. commune* may apply to other mushroom forming basidiomycetes as well. Hom2, Hom1, Fst3, Fst4, C2H2, and Gat1 are basidiomycete-specific regulatory proteins^14^. Homologues of these genes were identified in *L. bicolor* and *A. bisporus* ^8, 9^. The homologues for *fst4*, *fst3*, *c2h2*, and *hom1* were upregulated during sexual development in two *A. bisporus* varieties^9^, while homologues of *hom2*, *fst4*, *c2h2*, *fst3*, *gat1* and *hom1* showed similar expression expression profiles in *L. bicolor* showed similarity to *S. commune* ^9^. Expression patterns of *c2h2*, *fst3*, *hom1* and *gat1* were also found to be similar in *C. cinerea* ^10^. Moreover, it was recently shown that over-expression of the *c2h2* homoloque of *A. bisporus* accelerated mushroom development in this mushroom forming fungus^11^, which agrees with the phenotype of the Δ*c2h2*Δ*c2h2* phenotype of *S. commune.*

This is the first time a direct link has been shown between repression of vegetative growth and initiation of sexual reproduction. Previously, a link has been shown between vegetative growth and asexual development in *Aspergillus* ^15^. This link involves trimeric G-protein signaling. The activity of the Gα-subunit of *Aspergillus* is regulated by the FlbA protein^16^. Inactivation of this gene results in a strain that cannot initiate asexual development. Notably, *S. commune* has a homologue of *flbA* called *thn*. Inactivation of this gene results in a strain unable to form fruiting bodies^17^. This suggests that similar signaling pathways are involved in the decision to stop vegetative growth and to invest in reproduction in ascomycetes and basidiomycetes. A link between vegetative growth and development may also exist in *Podospora anserina*. Inactivation of the homeodomain gene *pah1* resulted in increased production of male gametes and increased branching of vegetative hyphae, resulting in smaller but more dense colonies^18, 19^.

## Methods

### Culture conditions and strains

The compatible *S. commune* strains H4-8 (*mat*A43*mat*B41; FGSC 9210)^8^ and H4-8b (*mat*A41*mat*B43)^20^, their derived wild-type dikaryotic strain, as well as the deletion strains Δ*wc-1*Δ*wc-1*, Δ*wc-2*Δ*wc-2*, Δ*hom2*Δ*hom2*, Δ*fst4*Δ*fst4*, Δ*c2h2*Δ*c2h2*, Δ*fst3*Δ*fst3*, Δ*hom1*Δ*hom1*Δ*bri1*Δ*bri1*, Δ*gat1*Δ*gat1* ^6–8^, Δ*hom2*Δ*hom2*Δ*fst4*Δ*fst4* and Δ*hom2*Δ*hom2*Δ*fst3*Δ*fst3* were used in this study. The Δ*ku80* H4-8 strain^21^ was used for gene inactivation. Strains were grown in the dark or in the light (1200 lux white LED light; Conrad Electronic, Hirschau, Germany) at 25 °C on minimal medium (MM) containing 1.5% agar and 2% glucose^22^. Cultures were inoculated with a point inoculum taken from the periphery of a 7-day-old colony. Liquid shaken cultures were inoculated with a mycelial homogenate^23^ and grown at 250 rpm in 250 ml Erlenmeyers containing 100 ml MM. To assess growth on other carbon sources, glucose was replaced by 4% xylose, 3.4% sucrose, or 1% pectin.

### Gene inactivation and complementation

Deletion vectors for *tea1* (Protein ID 2519514; http://genome.jgi-psf.org/Schco3) and *c2h2d* (Protein ID 2703923) were constructed using pDelcas that contains a nourseothricine and a phleomycin resistance cassette^20^. Upstream and downstream flanks of *tea1* and *c2h2d* were cloned at either site of the nourseothricine resistance cassette. To this end, the flanks were amplified by PCR using Taq polymerase and H4-8 chromosomal DNA as template. The 906 bp upstream flank and the 946 bp downstream flank of *tea1* were amplified using the primer combination Δ2519514ufw/Δ2519514urv and Δ2519514dfw/Δ2519514drv, respectively (Supplementary Table 6). Primer pair combinations ∆2703923ufw/∆2703923urv and ∆2703923dfw/∆2703923drv were used to amplify the 897 bp upstream and 975 bp downstream flank of *c2h2d*, respectively (Supplementary Table 6). The PCR products were cloned into pGEM-T Easy (Promega, Madison, USA). The upstream flanks were retrieved from the resulting constructs using Van91I and introduced into the Van91I site of pDelcas, resulting in pDel-2519514-UF and pDel-2703923-UF. The downstream flanks were retrieved from the pGEM-T Easy derived constructs using SfiI and introduced into the SfiI site of pDel_2519514-UF and pDel_2703923-UF. This resulted in the knock-out constructs pDelcas-2519514 and pDelcas-2703923.

To complement strain Δ*tea1* the coding region of *tea1* was amplified by PCR using primer pair tea1fw1/tea1rv1 (Supplementary Table 6) that introduce AarI and BamHI sites, respectively. The AarI/BamHI fragment was introduced in NcoI/BamHI cut vector pRO151^24^ that has a pUC20 backbone containing 1 kb HindIII/NcoI promoter sequence of *gpd1* gene from *S. commune*, a 350 bp BamHI/EcoRI terminator sequence from the *S. commune sc3* gene, and a EcoRI fragment containing a phleomycin resistance cassette. This resulted in pTEA1^comp^.

### Expression vector for Hom2^con^ of S. commune

Plasmid pHom2 is a pGMTphleoB derivative containing the *S. commune* gene *hom2* ^7^. The 1832 bp HindIII/SbfI fragment of pHom2 containing *hom2* coding and flanking sequences was cloned in pSP72 using the same sites, resulting in pHom2HF. In the next step, a 692 *hom2* coding fragment was synthesized (Genscript, Piscataway, NJ, USA), in which the serine codons in the 4 RRXS motifs were replaced for alanine (*hom2* ^*con*^). As a consequence of the substitution in the second motif a unique PvuI site was removed in *hom2* ^*con*^. A fusion PCR was performed with the 692 bp fragment and pHom2HF using T7 and SP6 primers annealing at both sides of the multiple cloning site of pHom2HF. The fusion fragment resulted in a mixture of 1922 bp *hom2* and *hom2* ^*con*^ fragments. The wild-type fragment was digested with PvuI. The remaining 1922 bp band was cut with HindIII/SbfI and used to replace the corresponding fragment in pHom2. This resulted in plasmid pHom2* containing the *hom2* ^*con*^ gene.

### Transformation of S. commune

Constructs were introduced in H4-8 or H4-8Δ*ku80* as described^22^ using 1·10^7^ protoplasts. For gene deletions, protoplasts were incubated with 20 μg vector DNA and regenerated overnight without antibiotic. Selection of transformants took place for 4 days at 30 °C on MM plates containing 8 μg ml^−1^ nourseothricin. Transformants were transferred to a second selection plate containing 5 μg ml^−1^ phleomycin to distinguish between homologous and ectopic integrations. Gene deletion was confirmed by PCR using primers outside the flanks and inside the nourseothricin cassette. Primer pairs 2519514ufcfw/nourdelrev and 2519514dfcrv/sc3tersqf were used to screen for *tea1* deletion (Supplementary Table 6), while primer pairs c2h2dufcfw/nourdelrev and c2h2ddfcrv/sc3tersqf were used to confirm *c2h2d* deletion (Supplementary Table 6).

For complementation or expression constructs, protoplasts were incubated with 5 μg vector DNA and regenerated overnight in the presence of 25 μg ml^−1^ phleomycin. Selection of transformants took place for 4 days at 30 °C on MM plates containing 25 μg ml^−1^ phleomycin.

### Whole genome expression analysis

The wild-type dikaryon and strains Δ*wc-1*Δ*wc-1*, Δ*wc-2*Δ*wc-2*, Δ*hom2*Δ*hom2*, Δ*fst4*Δ*fst4*, Δ*c2h2*Δ*c2h2*, Δ*fst3*Δ*fst3*, Δ*hom1*Δ*hom1*, Δ*bri1*Δ*bri1*, and Δ*gat1*Δ*gat1* were grown for 5 days in the dark at 22 °C, after which they were transferred to the light^25^. Biological duplicate whole cultures were harvested at the moment the wild-type dikaryon formed aggregates (day 8) or fruiting bodies (day 12). H4-8 colonies were also harvested at the moment they were transferred to the light (day 5) and when they had formed primordia (day 10). Mycelium was frozen in liquid nitrogen and homogenized using the TissueLyser II (Qiagen, Düsseldorf, Germany). RNA was extracted using TriZol (Life technologies, Carlsbad, USA) and purified using the NucleoSpin RNA kit (Macherey-Nagel, Düren, Germany). Quality of RNA was checked using the BioAnalyzer and sent to ServiceXS (Leiden, the Netherlands) for Illumina Next Generation Sequencing. RNAseq data have been deposited at NCBI under BioProject PRJNA323434.

### RNA-Seq Analysis Pipeline

STAR aligner^26^ was used to align the 100 bp paired end reads to the *S. commune* v3.0 genome (http://genome.jgi-psf.org/Schco3/Schco3.home.html). The size of introns was limited to a maximum of 1500 bp based on the largest intron sizes in the genome annotation. Abundance estimation and differential expression were analysed by Cufflinks version 2.1.1^27^, and Cuffdiff using a Benjamini Hochberg false discovery rate of 0.05^28^ excluding genes that had a number of fragments per kilobase of exon per million <1 in one or both of the strains or conditions. Enrichments of GO terms were analysed within sets of differentially expressed genes. Proteins annotated to contain a DNA-binding or regulatory protein domain were defined as transcription factors^8^.

### Restriction PCR

RNA was extracted from 10-day-old colonies grown on MM plates on top of a polycarbonate membrane using Trizol (Thermo Scientific, Waltham, MA, USA). cDNA was made using the QuantiTect Reverse Transcription Kit (Qiagen, Düsseldorf, Germany). In the next step, cDNA was amplified by PCR with the intron spanning primers hom2rtfw and hom2rtrv (Supplementary Table 6). The amplified wild-type 1117 bp fragment was cut with PvuI resulting in fragments of 609 and 508 bp. The *hom2* ^*con*^ fragment could not be cut by this enzyme, leaving the 1117 bp band intact.

### Bioinformatics

RRXS domains were identified in the protein sequences of *S. commune* using a custom Python script. Enrichment analyses were performed with Python and R to analyze over- and under-representation of functional annotation terms in sets of genes using the Fisher Exact test. The Benjamini-Hochberg correction was used to correct for multiple testing using a *p*-value ≤ 0.05. Orthologues of *S. commune hom2* in other basidiomycetes were identified by the presence of a homeobox PFAM domain (PF00046)^29^ and a reciprocal best blastp hit to *hom2*. These hits were manually curated using MycoCosm^30^ based on the available expression and homology data for that genome. The protein sequences were aligned using MAFFT version 7.123^31^ with the E-INS-i method. The alignment was visualized and manually curated using Jalview 2.8.2^32^. Conserved RRXS sites were identified in the alignment. A phylogenetic tree was reconstructed using RaxML version 8.1.16^33^ with the PROTGAMMAWAG model with 100 rapid bootstrap partitions. The tree and domain structures were visualized using the ETE Toolkit version 2.2.

### Biomass of colonies

Colonies were grown as liquid shaken cultures or on agar medium on a PC-membrane (diameter 76 mm, pore size 0.1 µm; Osmonics, GE Water Technologies) using biological triplicates. Mycelium of liquid cultures was separated from the medium using Miracloth filter (Merck Millipore, Billerica, USA). Mycelium was freeze-dried and weighed. Statistical analysis was done with an independent sample t-test (p-value ≤ 0.05) using IBM SPSS 20.

### Microscopy

Strains were grown in Petri dishes with a diameter of 4.5 cm within a 125 µm layer of MM agar for 2 days at 30 °C. Petri dishes were covered with wet tissue, 2 layers of plastic foil, and 1 layer of aluminum foil. Morphology of hyphae was studied with an inverted microscope using a 400-fold magnification. Statistical analysis was done with a χ^2^ test (p-value ≤ 0.05).

## Electronic supplementary material

Supplementary Tables and Figures
