# Supplementary material for: Transcription factors of Schizophyllum commune involved in mushroom formation and modulation of vegetative growth
Source: Sci Rep. 2017 Mar 22;7:310. doi: 10.1038/s41598-017-00483-3 (PMC5428507; doi:10.1038/s41598-017-00483-3)
Supplement: Supplementary file 1 — Supplementary Tables and Figures [file 41598_2017_483_MOESM1_ESM.pdf]

Supplementary Information

Transcription factors of *Schizophyllum commune* involved in mushroom formation and modulation of vegetative growth

Jordi F Pelkmans, Mohini B Patil, Thies Gehrmann, Marcel JT Reinders, Han A.B. Wösten, and Luis G. Lugones

Supplementary Table S1. Impact of deletion of transcription factor genes. Number of genes significantly up- and downregulated compared to wildtype at the moment the wild-type had formed aggregates (AG) and fruiting bodies (FB). Analysis is based on biological duplicates.

	Upregulated		Downregulated	
	AG	FB	AG	FB
Δ*wc1*Δ*wc1*	415	1195	431	1298
Δ*wc2*Δ*wc2*	375	1392	500	1421
Δ*hom2*Δ*hom2*	494	1267	652	1462
Δ*fst4*Δ*fst4*	1267	1150	1226	1330
Δ*c2h2*Δ*c2h2*	668	306	644	327
Δ*fst3*Δ*fst3*	400	86	482	131
Δ*hom1*Δ*hom1*	194	317	247	400
Δ*gat1*Δ*gat1*	480	662	556	991

Supplementary Table S2. Impact of deletion of transcription factor genes on whole genome expression. Enrichment of GO terms in up- and downregulated genes of 8-day-old colonies of Δ*wc-1*Δ*wc-1* and transcription factor deletion strains Δ*wc-2*Δ*wc-2*, Δ*hom2*Δ*hom2*, Δ*fst4*Δ*fst4*, Δ*c2h2*Δ*c2h2,* Δ*fst3*Δ*fst3,* Δ*hom1*Δ*hom1*, Δ*gat1*Δ*gat1,* and Δ*briI*Δ*briI* when compared to the aggregating wild-type strain. Analysis is based on biological duplicates.

Δwc-1Δwc-1

Up-regulated genes in mutant strain		Down-regulated genes in mutant strain	
GO term	Functional annotation	GO term	Functional annotation
0005975	carbohydrate metabolic process	0004497	oxidoreductase activity
0004553	hydrolase activity, hydrolyzing O-glycosyl compounds	0015171	monooxygenase activity
0008643	carbohydrate transport	0006865	amino acid transmembrane transporter activity
0008643	L-arabinose isomerase activity	0016491	electron transport
0005351	sugar:hydrogen symporter activity	0005618	amino acid transport
0016810	hydrolase activity, acting on carbon-nitrogen (but not peptide)	0006118	metabolic process
0005506	iron ion binding	0005199	glutathione transferase activity
0016614	oxidoreductase activity, acting on CH-OH group of donors	0004194	structural constituent of cell wall
0009082	branched chain family amino acid biosynthetic process	0006810	cell wall
0008812	choline dehydrogenase activity		

Δwc-2Δwc-2

Up-regulated genes in mutant strain		Down-regulated genes in mutant strain	
GO term	Functional annotation	GO term	Functional annotation
0005975	carbohydrate metabolic process	0016491	oxidoreductase activity
0004553	hydrolase activity, hydrolyzing O-glycosyl compounds	0004497	monooxygenase activity
0005506	iron ion binding	0043039	amino acid transmembrane transporter activity
0004497	monooxygenase activity	0006118	electron transport
0050381	unspecific monooxygenase activity	0006865	amino acid transport
0020037	heme binding	0008152	metabolic process
0016614	oxidoreductase activity, acting on CH-OH group of donors	0004364	glutathione transferase activity
0008812	choline dehydrogenase activity	0005199	structural constituent of cell wall
0009082	branched chain family amino acid biosynthetic process	0005618	cell wall
0006066	alcohol metabolic process	0050162	oxalate oxidase activity

Δhom2Δhom2

Up-regulated genes in mutant strain		Down-regulated genes in mutant strain	
GO term	Functional annotation	GO term	Functional annotation

0005975	carbohydrate metabolic process	0004497	monooxygenase activity
0004553	hydrolase activity, hydrolyzing O-glycosyl compounds	0006810	transport
0030246	carbohydrate binding	0020037	heme binding
0016491	oxidoreductase activity	0006118	electron transport
0003824	catalytic activity	0005215	transporter activity
0005215	transporter activity	0050381	unspecific monooxygenase activity
0008152	metabolic process	0005506	iron ion binding
0016021	integral to membrane	0015171	amino acid transmembrane transporter activity
0008422	beta-glucosidase activity	0006865	amino acid transport
0006118	electron transport	0016614	oxidoreductase activity, acting on CH-OH group of donors

Δfst4Δfst4

Up-regulated genes in mutant strain		Down-regulated genes in mutant strain	
GO term	Functional annotation	GO term	Functional annotation
0005975	carbohydrate metabolic process	0005524	ATP binding
0008733	L-arabinose isomerase activity	0003677	DNA binding
0005351	sugar:hydrogen symporter activity	0000166	nucleotide binding
0008643	carbohydrate transport	0008152	metabolic process
0005215	transporter activity	0005737	cytoplasm
0006810	transport	0017111	nucleoside-triphosphatase activity
0004553	hydrolase activity, hydrolyzing O-glycosyl compounds	0016491	oxidoreductase activity
0016021	integral to membrane	0003824	catalytic activity
0030246	carbohydrate binding	0016887	ATPase activity
0015171	amino acid transmembrane transporter activity	0005643	nuclear pore

Δc2h2Δc2h2

Up-regulated genes in mutant strain		Down-regulated genes in mutant strain	
GO term	Functional annotation	GO term	Functional annotation
0005506	iron ion binding	0005737	cytoplasm
0004497	monooxygenase activity	0005524	ATP binding
0006118	electron transport	0008152	metabolic process
0004553	hydrolase activity, hydrolyzing O-glycosyl compounds	0003824	catalytic activity
0020037	heme binding	0016491	oxidoreductase activity

0008733	L-arabinose isomerase activity	0044267	cellular protein metabolic process
0050381	unspecific monooxygenase activity	0000786	nucleosome
0005351	sugar:hydrogen symporter activity	0006334	nucleosome assembly
0050660	FAD binding	0004298	threonine endopeptidase activity
0008643	carbohydrate transport	0004299	proteasome endopeptidase activity

Δfst3Δfst3

Up-regulated genes in mutant strain		Down-regulated genes in mutant strain	
GO term	Functional annotation	GO term	Functional annotation
0005199	structural constituent of cell wall	0005506	iron ion binding
0005618	cell wall	0004497	monooxygenase activity
0016491	oxidoreductase activity	0020037	heme binding
0008152	metabolic process	0005975	carbohydrate metabolic process
0050660	FAD binding	0006118	electron transport
0016614	oxidoreductase activity, acting on CH-OH group of donors	0004553	hydrolase activity, hydrolyzing O-glycosyl compounds
0033754	indoleamine 2,3-dioxygenase activity	0050381	unspecific monooxygenase activity
0016717	oxidoreductase activity, acting on paired donors, with oxidation	0004568	chitinase activity
0020037	heme binding	0008843	endochitinase activity
0005215	transporter activity	0006032	chitin catabolic process

Δhom1Δhom1

Up-regulated genes in mutant strain		Down-regulated genes in mutant strain	
GO term	Functional annotation	GO term	Functional annotation
	NONE	0004568	chitinase activity
		0008843	endochitinase activity
		0004497	monooxygenase activity
		0016491	oxidoreductase activity
		0005975	carbohydrate metabolic process
		0004553	hydrolase activity, hydrolyzing O-glycosyl compounds
		0008115	sarcosine oxidase activity
		0006118	electron transport
		0006032	chitin catabolic process
		0006725	aromatic compound metabolic process

Δgat1Δgat1

Up-regulated genes in mutant strain		Down-regulated genes in mutant strain	
GO term	Functional annotation	GO term	Functional annotation
0005199	structural constituent of cell wall	0005975	carbohydrate metabolic process
0005618	cell wall	0005506	iron ion binding
0016491	oxidoreductase activity	0020037	heme binding
0008152	metabolic process	0006118	electron transport
0004497	monooxygenase activity	0004497	monooxygenase activity
0050660	FAD binding	0005524	ATP binding
0020037	heme binding	0004316	3-oxoacyl-[acyl-carrier-protein] reductase activity
0033754	indoleamine 2,3-dioxygenase activity	0004553	hydrolase activity, hydrolyzing O-glycosyl compounds
0016614	oxidoreductase activity, acting on CH-OH group of donors	0050381	unspecific monooxygenase activity
0006118	electron transport	0005215	transporter activity

Δbri1Δbri1

Up-regulated genes in mutant strain		Down-regulated genes in mutant strain	
GO term	Functional annotation	GO term	Functional annotation
	NONE	0008152	metabolic process
		0005975	carbohydrate metabolic process
		0003824	catalytic activity
		0000786	nucleosome
		0005215	transporter activity
		0005199	structural constituent of cell wall
		0006334	nucleosome assembly
		0005618	cell wall
		0006810	transport
		0016491	oxidoreductase activity

Supplementary Table S3. Genes differentially expressed in dikaryotic transcription factor deletion strains of *S. commune.* Analysis is based on biological duplicates.

Strain	Colony age	Protein ID	Up- / Down	Go Accession	Q value	Description
Δbri1	8-day	1147022	down	GO:0008152	2,59E-07	metabolic process
Δbri1	8-day	1193333	down	GO:0008152	2,59E-07	metabolic process
Δbri1	8-day	2213880	down	GO:0008152	2,59E-07	metabolic process
Δbri1	8-day	2499736	down	GO:0008152	2,59E-07	metabolic process
Δbri1	8-day	2505594	down	GO:0008152	2,59E-07	metabolic process
Δbri1	8-day	2508441	down	GO:0008152	2,59E-07	metabolic process
Δbri1	8-day	2512248	down	GO:0008152	2,59E-07	metabolic process
Δbri1	8-day	2534187	down	GO:0008152	2,59E-07	metabolic process
Δbri1	8-day	2556971	down	GO:0008152	2,59E-07	metabolic process
Δbri1	8-day	2589496	down	GO:0008152	2,59E-07	metabolic process
Δbri1	8-day	2601905	down	GO:0008152	2,59E-07	metabolic process
Δbri1	8-day	2604917	down	GO:0008152	2,59E-07	metabolic process
Δbri1	8-day	2615529	down	GO:0008152	2,59E-07	metabolic process
Δbri1	8-day	2630824	down	GO:0008152	2,59E-07	metabolic process
Δbri1	8-day	2641520	down	GO:0008152	2,59E-07	metabolic process
Δbri1	8-day	2642124	down	GO:0008152	2,59E-07	metabolic process
Δbri1	8-day	2642185	down	GO:0008152	2,59E-07	metabolic process
Δbri1	8-day	2673857	down	GO:0008152	2,59E-07	metabolic process
Δbri1	8-day	2690641	down	GO:0008152	2,59E-07	metabolic process
Δbri1	8-day	2738806	down	GO:0008152	2,59E-07	metabolic process
Δbri1	8-day	2746577	down	GO:0008152	2,59E-07	metabolic process
Δbri1	8-day	1129508	down	GO:0005215	3,04E-07	transporter activity
Δbri1	8-day	2515031	down	GO:0005215	3,04E-07	transporter activity
Δbri1	8-day	2550918	down	GO:0005215	3,04E-07	transporter activity
Δbri1	8-day	2600551	down	GO:0005215	3,04E-07	transporter activity
Δbri1	8-day	2616189	down	GO:0005215	3,04E-07	transporter activity
Δbri1	8-day	2630802	down	GO:0005215	3,04E-07	transporter activity
Δbri1	8-day	2637509	down	GO:0005215	3,04E-07	transporter activity

Δbri1	8-day	2642553	down	GO:0005215	3,04E-07	transporter activity
Δbri1	8-day	2704067	down	GO:0005215	3,04E-07	transporter activity
Δbri1	8-day	15462	down	GO:0005975	7,16E-07	carbohydrate metabolic process
Δbri1	8-day	73246	down	GO:0005975	7,16E-07	carbohydrate metabolic process
Δbri1	8-day	2499969	down	GO:0005975	7,16E-07	carbohydrate metabolic process
Δbri1	8-day	2557656	down	GO:0005975	7,16E-07	carbohydrate metabolic process
Δbri1	8-day	2570936	down	GO:0005975	7,16E-07	carbohydrate metabolic process
Δbri1	8-day	2609957	down	GO:0005975	7,16E-07	carbohydrate metabolic process
Δbri1	8-day	2613657	down	GO:0005975	7,16E-07	carbohydrate metabolic process
Δbri1	8-day	2621806	down	GO:0005975	7,16E-07	carbohydrate metabolic process
Δbri1	8-day	2628008	down	GO:0005975	7,16E-07	carbohydrate metabolic process
Δbri1	8-day	2645822	down	GO:0005975	7,16E-07	carbohydrate metabolic process
Δbri1	8-day	2645945	down	GO:0005975	7,16E-07	carbohydrate metabolic process
Δbri1	8-day	2686544	down	GO:0005975	7,16E-07	carbohydrate metabolic process
Δbri1	8-day	2706631	down	GO:0005975	7,16E-07	carbohydrate metabolic process
Δbri1	8-day	1129508	down	GO:0006810	7,85E-06	transport
Δbri1	8-day	1342503	down	GO:0006810	7,85E-06	transport
Δbri1	8-day	2515031	down	GO:0006810	7,85E-06	transport
Δbri1	8-day	2524154	down	GO:0006810	7,85E-06	transport
Δbri1	8-day	2550918	down	GO:0006810	7,85E-06	transport
Δbri1	8-day	2600551	down	GO:0006810	7,85E-06	transport
Δbri1	8-day	2616189	down	GO:0006810	7,85E-06	transport
Δbri1	8-day	2630802	down	GO:0006810	7,85E-06	transport
Δbri1	8-day	2637509	down	GO:0006810	7,85E-06	transport
Δbri1	8-day	2704067	down	GO:0006810	7,85E-06	transport
Δbri1	8-day	2746577	down	GO:0006810	7,85E-06	transport
Δbri1	8-day	2749926	down	GO:0006810	7,85E-06	transport
Δbri1	8-day	1147022	down	GO:0003824	1,25E-05	catalytic activity
Δbri1	8-day	1160309	down	GO:0003824	1,25E-05	catalytic activity
Δbri1	8-day	1193333	down	GO:0003824	1,25E-05	catalytic activity
Δbri1	8-day	2499736	down	GO:0003824	1,25E-05	catalytic activity
Δbri1	8-day	2556971	down	GO:0003824	1,25E-05	catalytic activity
Δbri1	8-day	2557656	down	GO:0003824	1,25E-05	catalytic activity
Δbri1	8-day	2570936	down	GO:0003824	1,25E-05	catalytic activity

Δbri1	8-day	2606333	down	GO:0003824	1,25E-05	catalytic activity
Δbri1	8-day	2615529	down	GO:0003824	1,25E-05	catalytic activity
Δbri1	8-day	2628008	down	GO:0003824	1,25E-05	catalytic activity
Δbri1	8-day	2631920	down	GO:0003824	1,25E-05	catalytic activity
Δbri1	8-day	2641520	down	GO:0003824	1,25E-05	catalytic activity
Δbri1	8-day	2642124	down	GO:0003824	1,25E-05	catalytic activity
Δbri1	8-day	2642438	down	GO:0003824	1,25E-05	catalytic activity
Δbri1	8-day	2673857	down	GO:0003824	1,25E-05	catalytic activity
Δbri1	8-day	2690641	down	GO:0003824	1,25E-05	catalytic activity
Δbri1	8-day	2746577	down	GO:0003824	1,25E-05	catalytic activity
Δbri1	8-day	2633783	down	GO:0000786	2,35E-05	nucleosome
Δbri1	8-day	2629603	down	GO:0005199	3,04E-05	structural constituent of cell wall
Δbri1	8-day	2629603	down	GO:0005618	4,41E-05	cell wall
Δbri1	8-day	2633783	down	GO:0006334	4,41E-05	nucleosome assembly
Δbri1	8-day	1147022	down	GO:0016491	7,01E-05	oxidoreductase activity
Δbri1	8-day	1160309	down	GO:0016491	7,01E-05	oxidoreductase activity
Δbri1	8-day	1194451	down	GO:0016491	7,01E-05	oxidoreductase activity
Δbri1	8-day	2213880	down	GO:0016491	7,01E-05	oxidoreductase activity
Δbri1	8-day	2481224	down	GO:0016491	7,01E-05	oxidoreductase activity
Δbri1	8-day	2490518	down	GO:0016491	7,01E-05	oxidoreductase activity
Δbri1	8-day	2499736	down	GO:0016491	7,01E-05	oxidoreductase activity
Δbri1	8-day	2504654	down	GO:0016491	7,01E-05	oxidoreductase activity
Δbri1	8-day	2511616	down	GO:0016491	7,01E-05	oxidoreductase activity
Δbri1	8-day	2517867	down	GO:0016491	7,01E-05	oxidoreductase activity
Δbri1	8-day	2534187	down	GO:0016491	7,01E-05	oxidoreductase activity
Δbri1	8-day	2556971	down	GO:0016491	7,01E-05	oxidoreductase activity
Δbri1	8-day	2603449	down	GO:0016491	7,01E-05	oxidoreductase activity
Δbri1	8-day	2615184	down	GO:0016491	7,01E-05	oxidoreductase activity
Δbri1	8-day	2618220	down	GO:0016491	7,01E-05	oxidoreductase activity
Δbri1	8-day	2622733	down	GO:0016491	7,01E-05	oxidoreductase activity
Δbri1	8-day	2639682	down	GO:0016491	7,01E-05	oxidoreductase activity
Δbri1	8-day	2641520	down	GO:0016491	7,01E-05	oxidoreductase activity
Δbri1	8-day	2642438	down	GO:0016491	7,01E-05	oxidoreductase activity
Δbri1	8-day	2521109	down	GO:0008236	0,000330993	serine-type peptidase activity

Δbri1	8-day	2627176	down	GO:0008236	0,000330993	serine-type peptidase activity
Δbri1	8-day	1129508	down	GO:0016021	0,000914733	integral to membrane
Δbri1	8-day	2515031	down	GO:0016021	0,000914733	integral to membrane
Δbri1	8-day	2524154	down	GO:0016021	0,000914733	integral to membrane
Δbri1	8-day	2550918	down	GO:0016021	0,000914733	integral to membrane
Δbri1	8-day	2603449	down	GO:0016021	0,000914733	integral to membrane
Δbri1	8-day	2616189	down	GO:0016021	0,000914733	integral to membrane
Δbri1	8-day	2625385	down	GO:0016021	0,000914733	integral to membrane
Δbri1	8-day	2637509	down	GO:0016021	0,000914733	integral to membrane
Δbri1	8-day	2704067	down	GO:0016021	0,000914733	integral to membrane
Δbri1	8-day	2746577	down	GO:0016021	0,000914733	integral to membrane
Δbri1	8-day	2515031	down	GO:0008733	0,002012671	L-arabinose isomerase activity
Δbri1	8-day	2550918	down	GO:0008733	0,002012671	L-arabinose isomerase activity
Δbri1	8-day	2616189	down	GO:0008733	0,002012671	L-arabinose isomerase activity
Δbri1	8-day	2704067	down	GO:0008733	0,002012671	L-arabinose isomerase activity
Δbri1	8-day	15462	down	GO:0004553	0,002945589	hydrolase activity, hydrolyzing O-glycosyl compounds
Δbri1	8-day	73246	down	GO:0004553	0,002945589	hydrolase activity, hydrolyzing O-glycosyl compounds
Δbri1	8-day	2499969	down	GO:0004553	0,002945589	hydrolase activity, hydrolyzing O-glycosyl compounds
Δbri1	8-day	2609957	down	GO:0004553	0,002945589	hydrolase activity, hydrolyzing O-glycosyl compounds
Δbri1	8-day	2613657	down	GO:0004553	0,002945589	hydrolase activity, hydrolyzing O-glycosyl compounds
Δbri1	8-day	2621806	down	GO:0004553	0,002945589	hydrolase activity, hydrolyzing O-glycosyl compounds
Δbri1	8-day	2645822	down	GO:0004553	0,002945589	hydrolase activity, hydrolyzing O-glycosyl compounds
Δbri1	8-day	2686544	down	GO:0004553	0,002945589	hydrolase activity, hydrolyzing O-glycosyl compounds
Δbri1	8-day	2706631	down	GO:0004553	0,002945589	hydrolase activity, hydrolyzing O-glycosyl compounds
Δbri1	8-day	2637509	down	GO:0015520	0,002945589	tetracycline:hydrogen antiporter activity
Δbri1	8-day	2637509	down	GO:0015904	0,002945589	tetracycline transport
Δbri1	8-day	81631	down	GO:0005506	0,006473026	iron ion binding
Δbri1	8-day	1188937	down	GO:0005506	0,006473026	iron ion binding
Δbri1	8-day	2537529	down	GO:0005506	0,006473026	iron ion binding
Δbri1	8-day	2583618	down	GO:0005506	0,006473026	iron ion binding
Δbri1	8-day	2603449	down	GO:0005506	0,006473026	iron ion binding
Δbri1	8-day	2618220	down	GO:0005506	0,006473026	iron ion binding
Δbri1	8-day	2633776	down	GO:0005506	0,006473026	iron ion binding
Δbri1	8-day	2635487	down	GO:0005506	0,006473026	iron ion binding

Δbri1	8-day	75642	down	GO:0006508	0,006473026	proteolysis
Δbri1	8-day	2009250	down	GO:0006508	0,006473026	proteolysis
Δbri1	8-day	2508594	down	GO:0006508	0,006473026	proteolysis
Δbri1	8-day	2521045	down	GO:0006508	0,006473026	proteolysis
Δbri1	8-day	2521109	down	GO:0006508	0,006473026	proteolysis
Δbri1	8-day	2627176	down	GO:0006508	0,006473026	proteolysis
Δbri1	8-day	2636358	down	GO:0006508	0,006473026	proteolysis
Δbri1	8-day	2515031	down	GO:0008643	0,00821797	carbohydrate transport
Δbri1	8-day	2550918	down	GO:0008643	0,00821797	carbohydrate transport
Δbri1	8-day	2616189	down	GO:0008643	0,00821797	carbohydrate transport
Δbri1	8-day	2704067	down	GO:0008643	0,00821797	carbohydrate transport
Δbri1	8-day	2637509	down	GO:0046677	0,010853578	response to antibiotic
Δbri1	8-day	81631	down	GO:0020037	0,010889651	heme binding
Δbri1	8-day	1188937	down	GO:0020037	0,010889651	heme binding
Δbri1	8-day	2537529	down	GO:0020037	0,010889651	heme binding
Δbri1	8-day	2583618	down	GO:0020037	0,010889651	heme binding
Δbri1	8-day	2633776	down	GO:0020037	0,010889651	heme binding
Δbri1	8-day	2635487	down	GO:0020037	0,010889651	heme binding
Δbri1	8-day	2640585	down	GO:0020037	0,010889651	heme binding
Δbri1	8-day	2515031	down	GO:0005351	0,015681867	sugar:hydrogen symporter activity
Δbri1	8-day	2550918	down	GO:0005351	0,015681867	sugar:hydrogen symporter activity
Δbri1	8-day	2616189	down	GO:0005351	0,015681867	sugar:hydrogen symporter activity
Δbri1	8-day	2704067	down	GO:0005351	0,015681867	sugar:hydrogen symporter activity
Δbri1	8-day	81631	down	GO:0004497	0,021208348	monooxygenase activity
Δbri1	8-day	1188937	down	GO:0004497	0,021208348	monooxygenase activity
Δbri1	8-day	2213880	down	GO:0004497	0,021208348	monooxygenase activity
Δbri1	8-day	2534187	down	GO:0004497	0,021208348	monooxygenase activity
Δbri1	8-day	2537529	down	GO:0004497	0,021208348	monooxygenase activity
Δbri1	8-day	2583618	down	GO:0004497	0,021208348	monooxygenase activity
Δbri1	8-day	2633776	down	GO:0004497	0,021208348	monooxygenase activity
Δbri1	8-day	2635487	down	GO:0004497	0,021208348	monooxygenase activity
Δbri1	8-day	2627176	down	GO:0019131	0,038454236	tripeptidyl-peptidase I activity
Δc2h2	8-day	2487208	up	GO:0005506	5,66E-09	iron ion binding
Δc2h2	8-day	2517480	up	GO:0005506	5,66E-09	iron ion binding

Δc2h2	8-day	2609502	up	GO:0005506	5,66E-09	iron ion binding
Δc2h2	8-day	2620407	up	GO:0005506	5,66E-09	iron ion binding
Δc2h2	8-day	2623189	up	GO:0005506	5,66E-09	iron ion binding
Δc2h2	8-day	2623701	up	GO:0005506	5,66E-09	iron ion binding
Δc2h2	8-day	2627525	up	GO:0005506	5,66E-09	iron ion binding
Δc2h2	8-day	2629864	up	GO:0005506	5,66E-09	iron ion binding
Δc2h2	8-day	2732977	up	GO:0005506	5,66E-09	iron ion binding
Δc2h2	8-day	2517480	up	GO:0004497	3,52E-08	monooxygenase activity
Δc2h2	8-day	2609502	up	GO:0004497	3,52E-08	monooxygenase activity
Δc2h2	8-day	2620407	up	GO:0004497	3,52E-08	monooxygenase activity
Δc2h2	8-day	2623701	up	GO:0004497	3,52E-08	monooxygenase activity
Δc2h2	8-day	2627525	up	GO:0004497	3,52E-08	monooxygenase activity
Δc2h2	8-day	2629864	up	GO:0004497	3,52E-08	monooxygenase activity
Δc2h2	8-day	2732977	up	GO:0004497	3,52E-08	monooxygenase activity
Δc2h2	8-day	2607659	up	GO:0008733	3,84E-08	L-arabinose isomerase activity
Δc2h2	8-day	2607659	up	GO:0005351	8,96E-08	sugar:hydrogen symporter activity
Δc2h2	8-day	2610180	up	GO:0005351	8,96E-08	sugar:hydrogen symporter activity
Δc2h2	8-day	2607659	up	GO:0008643	1,39E-07	carbohydrate transport
Δc2h2	8-day	59565	up	GO:0004553	3,93E-07	hydrolase activity, hydrolyzing O-glycosyl compounds
Δc2h2	8-day	2496051	up	GO:0004553	3,93E-07	hydrolase activity, hydrolyzing O-glycosyl compounds
Δc2h2	8-day	2506813	up	GO:0004553	3,93E-07	hydrolase activity, hydrolyzing O-glycosyl compounds
Δc2h2	8-day	2622563	up	GO:0004553	3,93E-07	hydrolase activity, hydrolyzing O-glycosyl compounds
Δc2h2	8-day	2623407	up	GO:0004553	3,93E-07	hydrolase activity, hydrolyzing O-glycosyl compounds
Δc2h2	8-day	2637065	up	GO:0004553	3,93E-07	hydrolase activity, hydrolyzing O-glycosyl compounds
Δc2h2	8-day	2499521	up	GO:0006118	4,03E-07	electron transport
Δc2h2	8-day	2517480	up	GO:0006118	4,03E-07	electron transport
Δc2h2	8-day	2582924	up	GO:0006118	4,03E-07	electron transport
Δc2h2	8-day	2606221	up	GO:0006118	4,03E-07	electron transport
Δc2h2	8-day	2608235	up	GO:0006118	4,03E-07	electron transport
Δc2h2	8-day	2609502	up	GO:0006118	4,03E-07	electron transport
Δc2h2	8-day	2612998	up	GO:0006118	4,03E-07	electron transport
Δc2h2	8-day	2613466	up	GO:0006118	4,03E-07	electron transport
Δc2h2	8-day	2614499	up	GO:0006118	4,03E-07	electron transport
Δc2h2	8-day	2620407	up	GO:0006118	4,03E-07	electron transport

Δc2h2	8-day	2623701	up	GO:0006118	4,03E-07	electron transport
Δc2h2	8-day	2627525	up	GO:0006118	4,03E-07	electron transport
Δc2h2	8-day	2628120	up	GO:0006118	4,03E-07	electron transport
Δc2h2	8-day	2629850	up	GO:0006118	4,03E-07	electron transport
Δc2h2	8-day	2629864	up	GO:0006118	4,03E-07	electron transport
Δc2h2	8-day	2643648	up	GO:0006118	4,03E-07	electron transport
Δc2h2	8-day	2671376	up	GO:0006118	4,03E-07	electron transport
Δc2h2	8-day	2732977	up	GO:0006118	4,03E-07	electron transport
Δc2h2	8-day	2487208	up	GO:0020037	1,88E-06	heme binding
Δc2h2	8-day	2517480	up	GO:0020037	1,88E-06	heme binding
Δc2h2	8-day	2582924	up	GO:0020037	1,88E-06	heme binding
Δc2h2	8-day	2609502	up	GO:0020037	1,88E-06	heme binding
Δc2h2	8-day	2620407	up	GO:0020037	1,88E-06	heme binding
Δc2h2	8-day	2623701	up	GO:0020037	1,88E-06	heme binding
Δc2h2	8-day	2627525	up	GO:0020037	1,88E-06	heme binding
Δc2h2	8-day	2629864	up	GO:0020037	1,88E-06	heme binding
Δc2h2	8-day	2732977	up	GO:0020037	1,88E-06	heme binding
Δc2h2	8-day	59565	up	GO:0005975	2,13E-06	carbohydrate metabolic process
Δc2h2	8-day	76183	up	GO:0005975	2,13E-06	carbohydrate metabolic process
Δc2h2	8-day	1087819	up	GO:0005975	2,13E-06	carbohydrate metabolic process
Δc2h2	8-day	2496051	up	GO:0005975	2,13E-06	carbohydrate metabolic process
Δc2h2	8-day	2506813	up	GO:0005975	2,13E-06	carbohydrate metabolic process
Δc2h2	8-day	2514546	up	GO:0005975	2,13E-06	carbohydrate metabolic process
Δc2h2	8-day	2607436	up	GO:0005975	2,13E-06	carbohydrate metabolic process
Δc2h2	8-day	2607718	up	GO:0005975	2,13E-06	carbohydrate metabolic process
Δc2h2	8-day	2612062	up	GO:0005975	2,13E-06	carbohydrate metabolic process
Δc2h2	8-day	2620263	up	GO:0005975	2,13E-06	carbohydrate metabolic process
Δc2h2	8-day	2622563	up	GO:0005975	2,13E-06	carbohydrate metabolic process
Δc2h2	8-day	2623407	up	GO:0005975	2,13E-06	carbohydrate metabolic process
Δc2h2	8-day	2624588	up	GO:0005975	2,13E-06	carbohydrate metabolic process
Δc2h2	8-day	2637065	up	GO:0005975	2,13E-06	carbohydrate metabolic process
Δc2h2	8-day	2620407	up	GO:0050381	3,10E-06	unspecific monooxygenase activity
Δc2h2	8-day	2732977	up	GO:0050381	3,10E-06	unspecific monooxygenase activity
Δc2h2	8-day	1087819	up	GO:0016614	3,86E-05	oxidoreductase activity, acting on CH-OH group of donors

Δc2h2	8-day	2614499	up	GO:0050660	4,33E-05	FAD binding
Δc2h2	8-day	2643648	up	GO:0050660	4,33E-05	FAD binding
Δc2h2	8-day	2488637	up	GO:0005215	0,000222054	transporter activity
Δc2h2	8-day	2590808	up	GO:0005215	0,000222054	transporter activity
Δc2h2	8-day	2607659	up	GO:0005215	0,000222054	transporter activity
Δc2h2	8-day	2607663	up	GO:0005215	0,000222054	transporter activity
Δc2h2	8-day	2611460	up	GO:0005215	0,000222054	transporter activity
Δc2h2	8-day	2612474	up	GO:0005215	0,000222054	transporter activity
Δc2h2	8-day	2620170	up	GO:0005215	0,000222054	transporter activity
Δc2h2	8-day	2622931	up	GO:0005215	0,000222054	transporter activity
Δc2h2	8-day	2628982	up	GO:0005215	0,000222054	transporter activity
Δc2h2	8-day	2633596	up	GO:0005215	0,000222054	transporter activity
Δc2h2	8-day	59565	up	GO:0004568	0,000245859	chitinase activity
Δc2h2	8-day	59565	up	GO:0008843	0,000245859	endochitinase activity
Δc2h2	8-day	2488637	up	GO:0006810	0,005134219	transport
Δc2h2	8-day	2569288	up	GO:0006810	0,005134219	transport
Δc2h2	8-day	2576922	up	GO:0006810	0,005134219	transport
Δc2h2	8-day	2590808	up	GO:0006810	0,005134219	transport
Δc2h2	8-day	2605313	up	GO:0006810	0,005134219	transport
Δc2h2	8-day	2607659	up	GO:0006810	0,005134219	transport
Δc2h2	8-day	2607663	up	GO:0006810	0,005134219	transport
Δc2h2	8-day	2611460	up	GO:0006810	0,005134219	transport
Δc2h2	8-day	2612474	up	GO:0006810	0,005134219	transport
Δc2h2	8-day	2620170	up	GO:0006810	0,005134219	transport
Δc2h2	8-day	2620257	up	GO:0006810	0,005134219	transport
Δc2h2	8-day	2622931	up	GO:0006810	0,005134219	transport
Δc2h2	8-day	2623894	up	GO:0006810	0,005134219	transport
Δc2h2	8-day	2628982	up	GO:0006810	0,005134219	transport
Δc2h2	8-day	2633596	up	GO:0006810	0,005134219	transport
Δc2h2	8-day	2637783	up	GO:0016831	0,005190225	carboxy-lyase activity
Δc2h2	8-day	59565	up	GO:0006032	0,012818818	chitin catabolic process
Δc2h2	8-day	1150677	up	GO:0051734	0,035764549	ATP-dependent polynucleotide kinase activity
Δc2h2	8-day	1150677	up	GO:0016538	0,035764549	cyclin-dependent protein kinase regulator activity
Δc2h2	8-day	1150677	up	GO:0008819	0,035764549	cobinamide kinase activity

Δc2h2	8-day	1150677	up	GO:0042556	0,035764549	eukaryotic elongation factor-2 kinase regulator activity
Δc2h2	8-day	1150677	up	GO:0042557	0,035764549	eukaryotic elongation factor-2 kinase activator activity
Δc2h2	8-day	1150677	up	GO:0008607	0,035764549	phosphorylase kinase regulator activity
Δc2h2	8-day	1150677	up	GO:0043841	0,035764549	(S)-lactate 2-kinase activity
Δc2h2	8-day	1150677	up	GO:0032942	0,035764549	inositol tetrakisphosphate 2-kinase activity
Δc2h2	8-day	1150677	up	GO:0043798	0,035764549	glycerate 2-kinase activity
Δc2h2	8-day	1150677	up	GO:0016307	0,035764549	phosphatidylinositol phosphate kinase activity
Δc2h2	8-day	1150677	up	GO:0019914	0,035764549	cyclin-dependent protein kinase activating kinase regulator activity
Δc2h2	8-day	1150677	up	GO:0043771	0,035764549	cytidine kinase activity
Δc2h2	8-day	1150677	up	GO:0008443	0,035764549	phosphofructokinase activity
Δc2h2	8-day	1150677	up	GO:0018720	0,035764549	phenol kinase activity
Δc2h2	8-day	1150677	up	GO:0043743	0,035764549	LPPG:FO 2-phospho-L-lactate transferase activity
Δc2h2	8-day	1150677	up	GO:0051731	0,035764549	polynucleotide kinase activity
Δc2h2	8-day	1150677	up	GO:0051735	0,035764549	GTP-dependent polynucleotide kinase activity
Δc2h2	8-day	1150677	up	GO:0035004	0,035764549	phosphoinositide 3-kinase activity
Δc2h2	8-day	2488637	up	GO:0016021	0,039386229	integral to membrane
Δc2h2	8-day	2507178	up	GO:0016021	0,039386229	integral to membrane
Δc2h2	8-day	2604210	up	GO:0016021	0,039386229	integral to membrane
Δc2h2	8-day	2607659	up	GO:0016021	0,039386229	integral to membrane
Δc2h2	8-day	2607663	up	GO:0016021	0,039386229	integral to membrane
Δc2h2	8-day	2612474	up	GO:0016021	0,039386229	integral to membrane
Δc2h2	8-day	2619090	up	GO:0016021	0,039386229	integral to membrane
Δc2h2	8-day	2620170	up	GO:0016021	0,039386229	integral to membrane
Δc2h2	8-day	2622931	up	GO:0016021	0,039386229	integral to membrane
Δc2h2	8-day	2628982	up	GO:0016021	0,039386229	integral to membrane
Δc2h2	8-day	1147022	down	GO:0008152	5,46E-09	metabolic process
Δc2h2	8-day	1156481	down	GO:0008152	5,46E-09	metabolic process
Δc2h2	8-day	1212612	down	GO:0008152	5,46E-09	metabolic process
Δc2h2	8-day	1332726	down	GO:0008152	5,46E-09	metabolic process
Δc2h2	8-day	2213880	down	GO:0008152	5,46E-09	metabolic process
Δc2h2	8-day	2373661	down	GO:0008152	5,46E-09	metabolic process
Δc2h2	8-day	2499736	down	GO:0008152	5,46E-09	metabolic process
Δc2h2	8-day	2501673	down	GO:0008152	5,46E-09	metabolic process
Δc2h2	8-day	2505594	down	GO:0008152	5,46E-09	metabolic process

Δc2h2	8-day	2511020	down	GO:0008152	5,46E-09	metabolic process
Δc2h2	8-day	2525914	down	GO:0008152	5,46E-09	metabolic process
Δc2h2	8-day	2573393	down	GO:0008152	5,46E-09	metabolic process
Δc2h2	8-day	2573396	down	GO:0008152	5,46E-09	metabolic process
Δc2h2	8-day	2589496	down	GO:0008152	5,46E-09	metabolic process
Δc2h2	8-day	2602078	down	GO:0008152	5,46E-09	metabolic process
Δc2h2	8-day	2604130	down	GO:0008152	5,46E-09	metabolic process
Δc2h2	8-day	2611306	down	GO:0008152	5,46E-09	metabolic process
Δc2h2	8-day	2623956	down	GO:0008152	5,46E-09	metabolic process
Δc2h2	8-day	2624569	down	GO:0008152	5,46E-09	metabolic process
Δc2h2	8-day	2625193	down	GO:0008152	5,46E-09	metabolic process
Δc2h2	8-day	2625782	down	GO:0008152	5,46E-09	metabolic process
Δc2h2	8-day	2625836	down	GO:0008152	5,46E-09	metabolic process
Δc2h2	8-day	2629548	down	GO:0008152	5,46E-09	metabolic process
Δc2h2	8-day	2631112	down	GO:0008152	5,46E-09	metabolic process
Δc2h2	8-day	2633167	down	GO:0008152	5,46E-09	metabolic process
Δc2h2	8-day	2637708	down	GO:0008152	5,46E-09	metabolic process
Δc2h2	8-day	2638445	down	GO:0008152	5,46E-09	metabolic process
Δc2h2	8-day	2641553	down	GO:0008152	5,46E-09	metabolic process
Δc2h2	8-day	2666990	down	GO:0008152	5,46E-09	metabolic process
Δc2h2	8-day	2673857	down	GO:0008152	5,46E-09	metabolic process
Δc2h2	8-day	2698029	down	GO:0008152	5,46E-09	metabolic process
Δc2h2	8-day	2705669	down	GO:0008152	5,46E-09	metabolic process
Δc2h2	8-day	15246	down	GO:0005524	1,21E-07	ATP binding
Δc2h2	8-day	1129062	down	GO:0005524	1,21E-07	ATP binding
Δc2h2	8-day	1131365	down	GO:0005524	1,21E-07	ATP binding
Δc2h2	8-day	1172108	down	GO:0005524	1,21E-07	ATP binding
Δc2h2	8-day	2253810	down	GO:0005524	1,21E-07	ATP binding
Δc2h2	8-day	2459580	down	GO:0005524	1,21E-07	ATP binding
Δc2h2	8-day	2527752	down	GO:0005524	1,21E-07	ATP binding
Δc2h2	8-day	2535527	down	GO:0005524	1,21E-07	ATP binding
Δc2h2	8-day	2610226	down	GO:0005524	1,21E-07	ATP binding
Δc2h2	8-day	2621253	down	GO:0005524	1,21E-07	ATP binding
Δc2h2	8-day	2642188	down	GO:0005524	1,21E-07	ATP binding

Δc2h2	8-day	2693043	down	GO:0005524	1,21E-07	ATP binding
Δc2h2	8-day	1147022	down	GO:0003824	2,19E-07	catalytic activity
Δc2h2	8-day	1158760	down	GO:0003824	2,19E-07	catalytic activity
Δc2h2	8-day	1212612	down	GO:0003824	2,19E-07	catalytic activity
Δc2h2	8-day	2373661	down	GO:0003824	2,19E-07	catalytic activity
Δc2h2	8-day	2499736	down	GO:0003824	2,19E-07	catalytic activity
Δc2h2	8-day	2511020	down	GO:0003824	2,19E-07	catalytic activity
Δc2h2	8-day	2525914	down	GO:0003824	2,19E-07	catalytic activity
Δc2h2	8-day	2557656	down	GO:0003824	2,19E-07	catalytic activity
Δc2h2	8-day	2598960	down	GO:0003824	2,19E-07	catalytic activity
Δc2h2	8-day	2607034	down	GO:0003824	2,19E-07	catalytic activity
Δc2h2	8-day	2620314	down	GO:0003824	2,19E-07	catalytic activity
Δc2h2	8-day	2623663	down	GO:0003824	2,19E-07	catalytic activity
Δc2h2	8-day	2625193	down	GO:0003824	2,19E-07	catalytic activity
Δc2h2	8-day	2625782	down	GO:0003824	2,19E-07	catalytic activity
Δc2h2	8-day	2626643	down	GO:0003824	2,19E-07	catalytic activity
Δc2h2	8-day	2628448	down	GO:0003824	2,19E-07	catalytic activity
Δc2h2	8-day	2629548	down	GO:0003824	2,19E-07	catalytic activity
Δc2h2	8-day	2630634	down	GO:0003824	2,19E-07	catalytic activity
Δc2h2	8-day	2630806	down	GO:0003824	2,19E-07	catalytic activity
Δc2h2	8-day	2631112	down	GO:0003824	2,19E-07	catalytic activity
Δc2h2	8-day	2631920	down	GO:0003824	2,19E-07	catalytic activity
Δc2h2	8-day	2633144	down	GO:0003824	2,19E-07	catalytic activity
Δc2h2	8-day	2633167	down	GO:0003824	2,19E-07	catalytic activity
Δc2h2	8-day	2635216	down	GO:0003824	2,19E-07	catalytic activity
Δc2h2	8-day	2638445	down	GO:0003824	2,19E-07	catalytic activity
Δc2h2	8-day	2641553	down	GO:0003824	2,19E-07	catalytic activity
Δc2h2	8-day	2642438	down	GO:0003824	2,19E-07	catalytic activity
Δc2h2	8-day	2666990	down	GO:0003824	2,19E-07	catalytic activity
Δc2h2	8-day	2673857	down	GO:0003824	2,19E-07	catalytic activity
Δc2h2	8-day	2688172	down	GO:0003824	2,19E-07	catalytic activity
Δc2h2	8-day	2698029	down	GO:0003824	2,19E-07	catalytic activity
Δc2h2	8-day	257768	down	GO:0016491	1,10E-06	oxidoreductase activity
Δc2h2	8-day	1147022	down	GO:0016491	1,10E-06	oxidoreductase activity

Δc2h2	8-day	1156481	down	GO:0016491	1,10E-06	oxidoreductase activity
Δc2h2	8-day	1332726	down	GO:0016491	1,10E-06	oxidoreductase activity
Δc2h2	8-day	2213880	down	GO:0016491	1,10E-06	oxidoreductase activity
Δc2h2	8-day	2312801	down	GO:0016491	1,10E-06	oxidoreductase activity
Δc2h2	8-day	2499736	down	GO:0016491	1,10E-06	oxidoreductase activity
Δc2h2	8-day	2501673	down	GO:0016491	1,10E-06	oxidoreductase activity
Δc2h2	8-day	2503108	down	GO:0016491	1,10E-06	oxidoreductase activity
Δc2h2	8-day	2515251	down	GO:0016491	1,10E-06	oxidoreductase activity
Δc2h2	8-day	2515739	down	GO:0016491	1,10E-06	oxidoreductase activity
Δc2h2	8-day	2517867	down	GO:0016491	1,10E-06	oxidoreductase activity
Δc2h2	8-day	2573393	down	GO:0016491	1,10E-06	oxidoreductase activity
Δc2h2	8-day	2573396	down	GO:0016491	1,10E-06	oxidoreductase activity
Δc2h2	8-day	2603449	down	GO:0016491	1,10E-06	oxidoreductase activity
Δc2h2	8-day	2611263	down	GO:0016491	1,10E-06	oxidoreductase activity
Δc2h2	8-day	2615184	down	GO:0016491	1,10E-06	oxidoreductase activity
Δc2h2	8-day	2618220	down	GO:0016491	1,10E-06	oxidoreductase activity
Δc2h2	8-day	2620314	down	GO:0016491	1,10E-06	oxidoreductase activity
Δc2h2	8-day	2622733	down	GO:0016491	1,10E-06	oxidoreductase activity
Δc2h2	8-day	2623663	down	GO:0016491	1,10E-06	oxidoreductase activity
Δc2h2	8-day	2625836	down	GO:0016491	1,10E-06	oxidoreductase activity
Δc2h2	8-day	2630033	down	GO:0016491	1,10E-06	oxidoreductase activity
Δc2h2	8-day	2633167	down	GO:0016491	1,10E-06	oxidoreductase activity
Δc2h2	8-day	2638445	down	GO:0016491	1,10E-06	oxidoreductase activity
Δc2h2	8-day	2642438	down	GO:0016491	1,10E-06	oxidoreductase activity
Δc2h2	8-day	2688172	down	GO:0016491	1,10E-06	oxidoreductase activity
Δc2h2	8-day	241481	down	GO:0016301	0,010029993	kinase activity
Δc2h2	8-day	1147022	down	GO:0005488	0,011216474	binding
Δc2h2	8-day	1212612	down	GO:0005488	0,011216474	binding
Δc2h2	8-day	2373661	down	GO:0005488	0,011216474	binding
Δc2h2	8-day	2499736	down	GO:0005488	0,011216474	binding
Δc2h2	8-day	2511020	down	GO:0005488	0,011216474	binding
Δc2h2	8-day	2538820	down	GO:0005488	0,011216474	binding
Δc2h2	8-day	2603669	down	GO:0005488	0,011216474	binding
Δc2h2	8-day	2620649	down	GO:0005488	0,011216474	binding

Δc2h2	8-day	2625193	down	GO:0005488	0,011216474	binding
Δc2h2	8-day	2625782	down	GO:0005488	0,011216474	binding
Δc2h2	8-day	2627883	down	GO:0005488	0,011216474	binding
Δc2h2	8-day	2630802	down	GO:0005488	0,011216474	binding
Δc2h2	8-day	2633167	down	GO:0005488	0,011216474	binding
Δc2h2	8-day	2638445	down	GO:0005488	0,011216474	binding
Δc2h2	8-day	2641553	down	GO:0005488	0,011216474	binding
Δc2h2	8-day	2673857	down	GO:0005488	0,011216474	binding
Δc2h2	8-day	2698029	down	GO:0005488	0,011216474	binding
Δc2h2	8-day	2749926	down	GO:0005488	0,011216474	binding
Δc2h2	8-day	1172108	down	GO:0000166	0,011780559	nucleotide binding
Δc2h2	8-day	2625193	down	GO:0050662	0,012318055	coenzyme binding
Δc2h2	8-day	2698029	down	GO:0050662	0,012318055	coenzyme binding
Δc2h2	8-day	2566425	down	GO:0006412	0,017903837	translation
Δc2h2	8-day	2621584	down	GO:0006412	0,017903837	translation
Δc2h2	8-day	2640577	down	GO:0006412	0,017903837	translation
Δc2h2	8-day	2560780	down	GO:0030170	0,033049543	pyridoxal phosphate binding
Δc2h2	8-day	2623956	down	GO:0030170	0,033049543	pyridoxal phosphate binding
Δc2h2	8-day	2625520	down	GO:0030170	0,033049543	pyridoxal phosphate binding
Δc2h2	8-day	2636719	down	GO:0030170	0,033049543	pyridoxal phosphate binding
Δc2h2	8-day	2625193	down	GO:0044237	0,033049543	cellular metabolic process
Δc2h2	8-day	2698029	down	GO:0044237	0,033049543	cellular metabolic process
Δc2h2	8-day	2626643	down	GO:0000287	0,047718317	magnesium ion binding
Δfst3	8-day	2629603	up	GO:0005199	1,34E-11	structural constituent of cell wall
Δfst3	8-day	2614257	up	GO:0005618	1,85E-11	cell wall
Δfst3	8-day	2629603	up	GO:0005618	1,85E-11	cell wall
Δfst3	8-day	257768	up	GO:0016491	3,82E-07	oxidoreductase activity
Δfst3	8-day	2255031	up	GO:0016491	3,82E-07	oxidoreductase activity
Δfst3	8-day	2333133	up	GO:0016491	3,82E-07	oxidoreductase activity
Δfst3	8-day	2498556	up	GO:0016491	3,82E-07	oxidoreductase activity
Δfst3	8-day	2503108	up	GO:0016491	3,82E-07	oxidoreductase activity
Δfst3	8-day	2604174	up	GO:0016491	3,82E-07	oxidoreductase activity
Δfst3	8-day	2608048	up	GO:0016491	3,82E-07	oxidoreductase activity
Δfst3	8-day	2609840	up	GO:0016491	3,82E-07	oxidoreductase activity

Δfst3	8-day	2617060	up	GO:0016491	3,82E-07	oxidoreductase activity
Δfst3	8-day	2619840	up	GO:0016491	3,82E-07	oxidoreductase activity
Δfst3	8-day	2621873	up	GO:0016491	3,82E-07	oxidoreductase activity
Δfst3	8-day	2627335	up	GO:0016491	3,82E-07	oxidoreductase activity
Δfst3	8-day	2634903	up	GO:0016491	3,82E-07	oxidoreductase activity
Δfst3	8-day	2638484	up	GO:0016491	3,82E-07	oxidoreductase activity
Δfst3	8-day	2639667	up	GO:0016491	3,82E-07	oxidoreductase activity
Δfst3	8-day	2641506	up	GO:0016491	3,82E-07	oxidoreductase activity
Δfst3	8-day	2641520	up	GO:0016491	3,82E-07	oxidoreductase activity
Δfst3	8-day	2667681	up	GO:0016491	3,82E-07	oxidoreductase activity
Δfst3	8-day	2681649	up	GO:0016491	3,82E-07	oxidoreductase activity
Δfst3	8-day	2688172	up	GO:0016491	3,82E-07	oxidoreductase activity
Δfst3	8-day	2248442	up	GO:0008152	3,69E-06	metabolic process
Δfst3	8-day	2255031	up	GO:0008152	3,69E-06	metabolic process
Δfst3	8-day	2333133	up	GO:0008152	3,69E-06	metabolic process
Δfst3	8-day	2373661	up	GO:0008152	3,69E-06	metabolic process
Δfst3	8-day	2492878	up	GO:0008152	3,69E-06	metabolic process
Δfst3	8-day	2541321	up	GO:0008152	3,69E-06	metabolic process
Δfst3	8-day	2577440	up	GO:0008152	3,69E-06	metabolic process
Δfst3	8-day	2608048	up	GO:0008152	3,69E-06	metabolic process
Δfst3	8-day	2611836	up	GO:0008152	3,69E-06	metabolic process
Δfst3	8-day	2615598	up	GO:0008152	3,69E-06	metabolic process
Δfst3	8-day	2619840	up	GO:0008152	3,69E-06	metabolic process
Δfst3	8-day	2621873	up	GO:0008152	3,69E-06	metabolic process
Δfst3	8-day	2625782	up	GO:0008152	3,69E-06	metabolic process
Δfst3	8-day	2626510	up	GO:0008152	3,69E-06	metabolic process
Δfst3	8-day	2627257	up	GO:0008152	3,69E-06	metabolic process
Δfst3	8-day	2629548	up	GO:0008152	3,69E-06	metabolic process
Δfst3	8-day	2632611	up	GO:0008152	3,69E-06	metabolic process
Δfst3	8-day	2634903	up	GO:0008152	3,69E-06	metabolic process
Δfst3	8-day	2637643	up	GO:0008152	3,69E-06	metabolic process
Δfst3	8-day	2638484	up	GO:0008152	3,69E-06	metabolic process
Δfst3	8-day	2640472	up	GO:0008152	3,69E-06	metabolic process
Δfst3	8-day	2640484	up	GO:0008152	3,69E-06	metabolic process

Δfst3	8-day	2641506	up	GO:0008152	3,69E-06	metabolic process
Δfst3	8-day	2641520	up	GO:0008152	3,69E-06	metabolic process
Δfst3	8-day	2643650	up	GO:0008152	3,69E-06	metabolic process
Δfst3	8-day	2673857	up	GO:0008152	3,69E-06	metabolic process
Δfst3	8-day	2681649	up	GO:0008152	3,69E-06	metabolic process
Δfst3	8-day	2696676	up	GO:0008152	3,69E-06	metabolic process
Δfst3	8-day	2699688	up	GO:0008152	3,69E-06	metabolic process
Δfst3	8-day	2711060	up	GO:0008152	3,69E-06	metabolic process
Δfst3	8-day	257768	up	GO:0050660	4,31E-05	FAD binding
Δfst3	8-day	2343034	up	GO:0050660	4,31E-05	FAD binding
Δfst3	8-day	2503108	up	GO:0050660	4,31E-05	FAD binding
Δfst3	8-day	2607677	up	GO:0050660	4,31E-05	FAD binding
Δfst3	8-day	2610771	up	GO:0050660	4,31E-05	FAD binding
Δfst3	8-day	2643012	up	GO:0050660	4,31E-05	FAD binding
Δfst3	8-day	2688172	up	GO:0050660	4,31E-05	FAD binding
Δfst3	8-day	78628	up	GO:0020037	0,000782424	heme binding
Δfst3	8-day	81631	up	GO:0020037	0,000782424	heme binding
Δfst3	8-day	1139445	up	GO:0020037	0,000782424	heme binding
Δfst3	8-day	1189794	up	GO:0020037	0,000782424	heme binding
Δfst3	8-day	2327903	up	GO:0020037	0,000782424	heme binding
Δfst3	8-day	2516208	up	GO:0020037	0,000782424	heme binding
Δfst3	8-day	2604096	up	GO:0020037	0,000782424	heme binding
Δfst3	8-day	2604458	up	GO:0020037	0,000782424	heme binding
Δfst3	8-day	2604628	up	GO:0020037	0,000782424	heme binding
Δfst3	8-day	2604735	up	GO:0020037	0,000782424	heme binding
Δfst3	8-day	2609840	up	GO:0020037	0,000782424	heme binding
Δfst3	8-day	2616301	up	GO:0020037	0,000782424	heme binding
Δfst3	8-day	2621855	up	GO:0020037	0,000782424	heme binding
Δfst3	8-day	2623115	up	GO:0020037	0,000782424	heme binding
Δfst3	8-day	2628651	up	GO:0020037	0,000782424	heme binding
Δfst3	8-day	2632925	up	GO:0020037	0,000782424	heme binding
Δfst3	8-day	2633776	up	GO:0020037	0,000782424	heme binding
Δfst3	8-day	2635487	up	GO:0020037	0,000782424	heme binding
Δfst3	8-day	2636544	up	GO:0020037	0,000782424	heme binding

Δfst3	8-day	2662483	up	GO:0020037	0,000782424	heme binding
Δfst3	8-day	2703628	up	GO:0020037	0,000782424	heme binding
Δfst3	8-day	2703966	up	GO:0020037	0,000782424	heme binding
Δfst3	8-day	2343034	up	GO:0016614	0,0015783	oxidoreductase activity, acting on CH-OH group of donors
Δfst3	8-day	2607677	up	GO:0016614	0,0015783	oxidoreductase activity, acting on CH-OH group of donors
Δfst3	8-day	2610771	up	GO:0016614	0,0015783	oxidoreductase activity, acting on CH-OH group of donors
Δfst3	8-day	2643012	up	GO:0016614	0,0015783	oxidoreductase activity, acting on CH-OH group of donors
Δfst3	8-day	78628	up	GO:0004497	0,0015783	monooxygenase activity
Δfst3	8-day	81631	up	GO:0004497	0,0015783	monooxygenase activity
Δfst3	8-day	1139445	up	GO:0004497	0,0015783	monooxygenase activity
Δfst3	8-day	1189794	up	GO:0004497	0,0015783	monooxygenase activity
Δfst3	8-day	2327903	up	GO:0004497	0,0015783	monooxygenase activity
Δfst3	8-day	2516208	up	GO:0004497	0,0015783	monooxygenase activity
Δfst3	8-day	2604096	up	GO:0004497	0,0015783	monooxygenase activity
Δfst3	8-day	2604458	up	GO:0004497	0,0015783	monooxygenase activity
Δfst3	8-day	2604735	up	GO:0004497	0,0015783	monooxygenase activity
Δfst3	8-day	2616301	up	GO:0004497	0,0015783	monooxygenase activity
Δfst3	8-day	2619840	up	GO:0004497	0,0015783	monooxygenase activity
Δfst3	8-day	2623115	up	GO:0004497	0,0015783	monooxygenase activity
Δfst3	8-day	2628651	up	GO:0004497	0,0015783	monooxygenase activity
Δfst3	8-day	2632925	up	GO:0004497	0,0015783	monooxygenase activity
Δfst3	8-day	2633776	up	GO:0004497	0,0015783	monooxygenase activity
Δfst3	8-day	2635487	up	GO:0004497	0,0015783	monooxygenase activity
Δfst3	8-day	2636544	up	GO:0004497	0,0015783	monooxygenase activity
Δfst3	8-day	2662483	up	GO:0004497	0,0015783	monooxygenase activity
Δfst3	8-day	2703628	up	GO:0004497	0,0015783	monooxygenase activity
Δfst3	8-day	2703966	up	GO:0004497	0,0015783	monooxygenase activity
Δfst3	8-day	78628	up	GO:0006118	0,007592149	electron transport
Δfst3	8-day	81631	up	GO:0006118	0,007592149	electron transport
Δfst3	8-day	257768	up	GO:0006118	0,007592149	electron transport
Δfst3	8-day	1139445	up	GO:0006118	0,007592149	electron transport
Δfst3	8-day	1189794	up	GO:0006118	0,007592149	electron transport
Δfst3	8-day	2327903	up	GO:0006118	0,007592149	electron transport
Δfst3	8-day	2498556	up	GO:0006118	0,007592149	electron transport

Δfst3	8-day	2503108	up	GO:0006118	0,007592149	electron transport
Δfst3	8-day	2507628	up	GO:0006118	0,007592149	electron transport
Δfst3	8-day	2516208	up	GO:0006118	0,007592149	electron transport
Δfst3	8-day	2604096	up	GO:0006118	0,007592149	electron transport
Δfst3	8-day	2604458	up	GO:0006118	0,007592149	electron transport
Δfst3	8-day	2604628	up	GO:0006118	0,007592149	electron transport
Δfst3	8-day	2604735	up	GO:0006118	0,007592149	electron transport
Δfst3	8-day	2609831	up	GO:0006118	0,007592149	electron transport
Δfst3	8-day	2609840	up	GO:0006118	0,007592149	electron transport
Δfst3	8-day	2616301	up	GO:0006118	0,007592149	electron transport
Δfst3	8-day	2619840	up	GO:0006118	0,007592149	electron transport
Δfst3	8-day	2620726	up	GO:0006118	0,007592149	electron transport
Δfst3	8-day	2621855	up	GO:0006118	0,007592149	electron transport
Δfst3	8-day	2623115	up	GO:0006118	0,007592149	electron transport
Δfst3	8-day	2628651	up	GO:0006118	0,007592149	electron transport
Δfst3	8-day	2632925	up	GO:0006118	0,007592149	electron transport
Δfst3	8-day	2633776	up	GO:0006118	0,007592149	electron transport
Δfst3	8-day	2635487	up	GO:0006118	0,007592149	electron transport
Δfst3	8-day	2636544	up	GO:0006118	0,007592149	electron transport
Δfst3	8-day	2662483	up	GO:0006118	0,007592149	electron transport
Δfst3	8-day	2703628	up	GO:0006118	0,007592149	electron transport
Δfst3	8-day	2703966	up	GO:0006118	0,007592149	electron transport
Δfst3	8-day	78628	up	GO:0005506	0,013346341	iron ion binding
Δfst3	8-day	81631	up	GO:0005506	0,013346341	iron ion binding
Δfst3	8-day	1139445	up	GO:0005506	0,013346341	iron ion binding
Δfst3	8-day	1189794	up	GO:0005506	0,013346341	iron ion binding
Δfst3	8-day	2327903	up	GO:0005506	0,013346341	iron ion binding
Δfst3	8-day	2503108	up	GO:0005506	0,013346341	iron ion binding
Δfst3	8-day	2516208	up	GO:0005506	0,013346341	iron ion binding
Δfst3	8-day	2604096	up	GO:0005506	0,013346341	iron ion binding
Δfst3	8-day	2604458	up	GO:0005506	0,013346341	iron ion binding
Δfst3	8-day	2604628	up	GO:0005506	0,013346341	iron ion binding
Δfst3	8-day	2604735	up	GO:0005506	0,013346341	iron ion binding
Δfst3	8-day	2616301	up	GO:0005506	0,013346341	iron ion binding

Δfst3	8-day	2617060	up	GO:0005506	0,013346341	iron ion binding
Δfst3	8-day	2621855	up	GO:0005506	0,013346341	iron ion binding
Δfst3	8-day	2623115	up	GO:0005506	0,013346341	iron ion binding
Δfst3	8-day	2628651	up	GO:0005506	0,013346341	iron ion binding
Δfst3	8-day	2632925	up	GO:0005506	0,013346341	iron ion binding
Δfst3	8-day	2633776	up	GO:0005506	0,013346341	iron ion binding
Δfst3	8-day	2635487	up	GO:0005506	0,013346341	iron ion binding
Δfst3	8-day	2636544	up	GO:0005506	0,013346341	iron ion binding
Δfst3	8-day	2662483	up	GO:0005506	0,013346341	iron ion binding
Δfst3	8-day	2703628	up	GO:0005506	0,013346341	iron ion binding
Δfst3	8-day	2703966	up	GO:0005506	0,013346341	iron ion binding
Δfst3	8-day	2577440	up	GO:0000162	0,02352372	tryptophan biosynthetic process
Δfst3	8-day	2343034	up	GO:0006066	0,024633906	alcohol metabolic process
Δfst3	8-day	2607677	up	GO:0006066	0,024633906	alcohol metabolic process
Δfst3	8-day	2610771	up	GO:0006066	0,024633906	alcohol metabolic process
Δfst3	8-day	2643012	up	GO:0006066	0,024633906	alcohol metabolic process
Δfst3	8-day	2343034	up	GO:0008812	0,024633906	choline dehydrogenase activity
Δfst3	8-day	2607677	up	GO:0008812	0,024633906	choline dehydrogenase activity
Δfst3	8-day	2610771	up	GO:0008812	0,024633906	choline dehydrogenase activity
Δfst3	8-day	2643012	up	GO:0008812	0,024633906	choline dehydrogenase activity
Δfst3	8-day	68300	up	GO:0005215	0,040947206	transporter activity
Δfst3	8-day	2492878	up	GO:0005215	0,040947206	transporter activity
Δfst3	8-day	2607025	up	GO:0005215	0,040947206	transporter activity
Δfst3	8-day	2612313	up	GO:0005215	0,040947206	transporter activity
Δfst3	8-day	2616189	up	GO:0005215	0,040947206	transporter activity
Δfst3	8-day	2616961	up	GO:0005215	0,040947206	transporter activity
Δfst3	8-day	2620178	up	GO:0005215	0,040947206	transporter activity
Δfst3	8-day	2623043	up	GO:0005215	0,040947206	transporter activity
Δfst3	8-day	2627703	up	GO:0005215	0,040947206	transporter activity
Δfst3	8-day	2630802	up	GO:0005215	0,040947206	transporter activity
Δfst3	8-day	2634793	up	GO:0005215	0,040947206	transporter activity
Δfst3	8-day	2637509	up	GO:0005215	0,040947206	transporter activity
Δfst3	8-day	2641735	up	GO:0005215	0,040947206	transporter activity
Δfst3	8-day	1083109	down	GO:0005506	5,74E-07	iron ion binding

Δfst3	8-day	1120318	down	GO:0005506	5,74E-07	iron ion binding
Δfst3	8-day	2364606	down	GO:0005506	5,74E-07	iron ion binding
Δfst3	8-day	2491624	down	GO:0005506	5,74E-07	iron ion binding
Δfst3	8-day	2501258	down	GO:0005506	5,74E-07	iron ion binding
Δfst3	8-day	2511002	down	GO:0005506	5,74E-07	iron ion binding
Δfst3	8-day	2537529	down	GO:0005506	5,74E-07	iron ion binding
Δfst3	8-day	2559987	down	GO:0005506	5,74E-07	iron ion binding
Δfst3	8-day	2618220	down	GO:0005506	5,74E-07	iron ion binding
Δfst3	8-day	2619716	down	GO:0005506	5,74E-07	iron ion binding
Δfst3	8-day	2628941	down	GO:0005506	5,74E-07	iron ion binding
Δfst3	8-day	2635870	down	GO:0005506	5,74E-07	iron ion binding
Δfst3	8-day	2703965	down	GO:0005506	5,74E-07	iron ion binding
Δfst3	8-day	1083109	down	GO:0020037	5,50E-06	heme binding
Δfst3	8-day	1120318	down	GO:0020037	5,50E-06	heme binding
Δfst3	8-day	2364606	down	GO:0020037	5,50E-06	heme binding
Δfst3	8-day	2491624	down	GO:0020037	5,50E-06	heme binding
Δfst3	8-day	2501258	down	GO:0020037	5,50E-06	heme binding
Δfst3	8-day	2511002	down	GO:0020037	5,50E-06	heme binding
Δfst3	8-day	2537529	down	GO:0020037	5,50E-06	heme binding
Δfst3	8-day	2559987	down	GO:0020037	5,50E-06	heme binding
Δfst3	8-day	2608091	down	GO:0020037	5,50E-06	heme binding
Δfst3	8-day	2619716	down	GO:0020037	5,50E-06	heme binding
Δfst3	8-day	2622893	down	GO:0020037	5,50E-06	heme binding
Δfst3	8-day	2628941	down	GO:0020037	5,50E-06	heme binding
Δfst3	8-day	2635870	down	GO:0020037	5,50E-06	heme binding
Δfst3	8-day	2640585	down	GO:0020037	5,50E-06	heme binding
Δfst3	8-day	2673121	down	GO:0020037	5,50E-06	heme binding
Δfst3	8-day	2703965	down	GO:0020037	5,50E-06	heme binding
Δfst3	8-day	1120318	down	GO:0004497	5,50E-06	monooxygenase activity
Δfst3	8-day	1342670	down	GO:0004497	5,50E-06	monooxygenase activity
Δfst3	8-day	2364606	down	GO:0004497	5,50E-06	monooxygenase activity
Δfst3	8-day	2491624	down	GO:0004497	5,50E-06	monooxygenase activity
Δfst3	8-day	2501258	down	GO:0004497	5,50E-06	monooxygenase activity
Δfst3	8-day	2537529	down	GO:0004497	5,50E-06	monooxygenase activity

Δfst3	8-day	2559987	down	GO:0004497	5,50E-06	monooxygenase activity
Δfst3	8-day	2573393	down	GO:0004497	5,50E-06	monooxygenase activity
Δfst3	8-day	2573396	down	GO:0004497	5,50E-06	monooxygenase activity
Δfst3	8-day	2619716	down	GO:0004497	5,50E-06	monooxygenase activity
Δfst3	8-day	2628941	down	GO:0004497	5,50E-06	monooxygenase activity
Δfst3	8-day	2635870	down	GO:0004497	5,50E-06	monooxygenase activity
Δfst3	8-day	2703927	down	GO:0004497	5,50E-06	monooxygenase activity
Δfst3	8-day	2703965	down	GO:0004497	5,50E-06	monooxygenase activity
Δfst3	8-day	1120318	down	GO:0006118	1,72E-05	electron transport
Δfst3	8-day	1342670	down	GO:0006118	1,72E-05	electron transport
Δfst3	8-day	1356674	down	GO:0006118	1,72E-05	electron transport
Δfst3	8-day	2364606	down	GO:0006118	1,72E-05	electron transport
Δfst3	8-day	2491624	down	GO:0006118	1,72E-05	electron transport
Δfst3	8-day	2501258	down	GO:0006118	1,72E-05	electron transport
Δfst3	8-day	2508276	down	GO:0006118	1,72E-05	electron transport
Δfst3	8-day	2537529	down	GO:0006118	1,72E-05	electron transport
Δfst3	8-day	2559987	down	GO:0006118	1,72E-05	electron transport
Δfst3	8-day	2573393	down	GO:0006118	1,72E-05	electron transport
Δfst3	8-day	2573396	down	GO:0006118	1,72E-05	electron transport
Δfst3	8-day	2605889	down	GO:0006118	1,72E-05	electron transport
Δfst3	8-day	2619716	down	GO:0006118	1,72E-05	electron transport
Δfst3	8-day	2628120	down	GO:0006118	1,72E-05	electron transport
Δfst3	8-day	2628941	down	GO:0006118	1,72E-05	electron transport
Δfst3	8-day	2635870	down	GO:0006118	1,72E-05	electron transport
Δfst3	8-day	2638473	down	GO:0006118	1,72E-05	electron transport
Δfst3	8-day	2666009	down	GO:0006118	1,72E-05	electron transport
Δfst3	8-day	2703927	down	GO:0006118	1,72E-05	electron transport
Δfst3	8-day	2703965	down	GO:0006118	1,72E-05	electron transport
Δfst3	8-day	1087819	down	GO:0005975	6,13E-05	carbohydrate metabolic process
Δfst3	8-day	2514546	down	GO:0005975	6,13E-05	carbohydrate metabolic process
Δfst3	8-day	2605157	down	GO:0005975	6,13E-05	carbohydrate metabolic process
Δfst3	8-day	2612719	down	GO:0005975	6,13E-05	carbohydrate metabolic process
Δfst3	8-day	2613657	down	GO:0005975	6,13E-05	carbohydrate metabolic process
Δfst3	8-day	2636145	down	GO:0005975	6,13E-05	carbohydrate metabolic process

Δfst3	8-day	1120318	down	GO:0050381	6,26E-05	unspecific monooxygenase activity
Δfst3	8-day	2491624	down	GO:0050381	6,26E-05	unspecific monooxygenase activity
Δfst3	8-day	2559987	down	GO:0050381	6,26E-05	unspecific monooxygenase activity
Δfst3	8-day	2619716	down	GO:0050381	6,26E-05	unspecific monooxygenase activity
Δfst3	8-day	2703965	down	GO:0050381	6,26E-05	unspecific monooxygenase activity
Δfst3	8-day	1158800	down	GO:0005524	0,000119131	ATP binding
Δfst3	8-day	1181112	down	GO:0005524	0,000119131	ATP binding
Δfst3	8-day	2486797	down	GO:0005524	0,000119131	ATP binding
Δfst3	8-day	2490823	down	GO:0005524	0,000119131	ATP binding
Δfst3	8-day	2510275	down	GO:0005524	0,000119131	ATP binding
Δfst3	8-day	2510924	down	GO:0005524	0,000119131	ATP binding
Δfst3	8-day	2511561	down	GO:0005524	0,000119131	ATP binding
Δfst3	8-day	2514780	down	GO:0005524	0,000119131	ATP binding
Δfst3	8-day	2535079	down	GO:0005524	0,000119131	ATP binding
Δfst3	8-day	2581797	down	GO:0005524	0,000119131	ATP binding
Δfst3	8-day	2631390	down	GO:0005524	0,000119131	ATP binding
Δfst3	8-day	2636368	down	GO:0005524	0,000119131	ATP binding
Δfst3	8-day	2641678	down	GO:0005524	0,000119131	ATP binding
Δfst3	8-day	2661304	down	GO:0005524	0,000119131	ATP binding
Δfst3	8-day	2675859	down	GO:0005524	0,000119131	ATP binding
Δfst3	8-day	2605157	down	GO:0004553	0,000632868	hydrolase activity, hydrolyzing O-glycosyl compounds
Δfst3	8-day	2612719	down	GO:0004553	0,000632868	hydrolase activity, hydrolyzing O-glycosyl compounds
Δfst3	8-day	2613657	down	GO:0004553	0,000632868	hydrolase activity, hydrolyzing O-glycosyl compounds
Δfst3	8-day	13059	down	GO:0006508	0,016298351	proteolysis
Δfst3	8-day	2493797	down	GO:0006508	0,016298351	proteolysis
Δfst3	8-day	2494345	down	GO:0006508	0,016298351	proteolysis
Δfst3	8-day	2501086	down	GO:0006508	0,016298351	proteolysis
Δfst3	8-day	2625721	down	GO:0006508	0,016298351	proteolysis
Δfst3	8-day	2627176	down	GO:0006508	0,016298351	proteolysis
Δfst3	8-day	2639300	down	GO:0006508	0,016298351	proteolysis
Δfst4	8-day	1087819	up	GO:0005975	2,26E-05	carbohydrate metabolic process
Δfst4	8-day	1110887	up	GO:0005975	2,26E-05	carbohydrate metabolic process
Δfst4	8-day	1186967	up	GO:0005975	2,26E-05	carbohydrate metabolic process
Δfst4	8-day	2501021	up	GO:0005975	2,26E-05	carbohydrate metabolic process

Δfst4	8-day	2514546	up	GO:0005975	2,26E-05	carbohydrate metabolic process
Δfst4	8-day	2603975	up	GO:0005975	2,26E-05	carbohydrate metabolic process
Δfst4	8-day	2607436	up	GO:0005975	2,26E-05	carbohydrate metabolic process
Δfst4	8-day	2620263	up	GO:0005975	2,26E-05	carbohydrate metabolic process
Δfst4	8-day	2622489	up	GO:0005975	2,26E-05	carbohydrate metabolic process
Δfst4	8-day	2622563	up	GO:0005975	2,26E-05	carbohydrate metabolic process
Δfst4	8-day	2623407	up	GO:0005975	2,26E-05	carbohydrate metabolic process
Δfst4	8-day	2626756	up	GO:0005975	2,26E-05	carbohydrate metabolic process
Δfst4	8-day	2630789	up	GO:0005975	2,26E-05	carbohydrate metabolic process
Δfst4	8-day	2633816	up	GO:0005975	2,26E-05	carbohydrate metabolic process
Δfst4	8-day	2641022	up	GO:0005975	2,26E-05	carbohydrate metabolic process
Δfst4	8-day	2645822	up	GO:0005975	2,26E-05	carbohydrate metabolic process
Δfst4	8-day	2689018	up	GO:0005975	2,26E-05	carbohydrate metabolic process
Δfst4	8-day	2607659	up	GO:0008733	5,85E-05	L-arabinose isomerase activity
Δfst4	8-day	2623420	up	GO:0008733	5,85E-05	L-arabinose isomerase activity
Δfst4	8-day	2641095	up	GO:0008733	5,85E-05	L-arabinose isomerase activity
Δfst4	8-day	1171024	up	GO:0005215	0,000120554	transporter activity
Δfst4	8-day	1196424	up	GO:0005215	0,000120554	transporter activity
Δfst4	8-day	2358954	up	GO:0005215	0,000120554	transporter activity
Δfst4	8-day	2488637	up	GO:0005215	0,000120554	transporter activity
Δfst4	8-day	2499472	up	GO:0005215	0,000120554	transporter activity
Δfst4	8-day	2502284	up	GO:0005215	0,000120554	transporter activity
Δfst4	8-day	2508421	up	GO:0005215	0,000120554	transporter activity
Δfst4	8-day	2543516	up	GO:0005215	0,000120554	transporter activity
Δfst4	8-day	2550496	up	GO:0005215	0,000120554	transporter activity
Δfst4	8-day	2590808	up	GO:0005215	0,000120554	transporter activity
Δfst4	8-day	2607659	up	GO:0005215	0,000120554	transporter activity
Δfst4	8-day	2607663	up	GO:0005215	0,000120554	transporter activity
Δfst4	8-day	2610799	up	GO:0005215	0,000120554	transporter activity
Δfst4	8-day	2611460	up	GO:0005215	0,000120554	transporter activity
Δfst4	8-day	2611686	up	GO:0005215	0,000120554	transporter activity
Δfst4	8-day	2615463	up	GO:0005215	0,000120554	transporter activity
Δfst4	8-day	2616961	up	GO:0005215	0,000120554	transporter activity
Δfst4	8-day	2618842	up	GO:0005215	0,000120554	transporter activity

Δfst4	8-day	2620170	up	GO:0005215	0,000120554	transporter activity
Δfst4	8-day	2622931	up	GO:0005215	0,000120554	transporter activity
Δfst4	8-day	2623420	up	GO:0005215	0,000120554	transporter activity
Δfst4	8-day	2624743	up	GO:0005215	0,000120554	transporter activity
Δfst4	8-day	2628098	up	GO:0005215	0,000120554	transporter activity
Δfst4	8-day	2628751	up	GO:0005215	0,000120554	transporter activity
Δfst4	8-day	2628982	up	GO:0005215	0,000120554	transporter activity
Δfst4	8-day	2631348	up	GO:0005215	0,000120554	transporter activity
Δfst4	8-day	2632105	up	GO:0005215	0,000120554	transporter activity
Δfst4	8-day	2633354	up	GO:0005215	0,000120554	transporter activity
Δfst4	8-day	2633596	up	GO:0005215	0,000120554	transporter activity
Δfst4	8-day	2637509	up	GO:0005215	0,000120554	transporter activity
Δfst4	8-day	2637652	up	GO:0005215	0,000120554	transporter activity
Δfst4	8-day	2641095	up	GO:0005215	0,000120554	transporter activity
Δfst4	8-day	2641989	up	GO:0005215	0,000120554	transporter activity
Δfst4	8-day	2644053	up	GO:0005215	0,000120554	transporter activity
Δfst4	8-day	2607659	up	GO:0005351	0,000120554	sugar:hydrogen symporter activity
Δfst4	8-day	2610180	up	GO:0005351	0,000120554	sugar:hydrogen symporter activity
Δfst4	8-day	2623420	up	GO:0005351	0,000120554	sugar:hydrogen symporter activity
Δfst4	8-day	2641095	up	GO:0005351	0,000120554	sugar:hydrogen symporter activity
Δfst4	8-day	2607659	up	GO:0008643	0,000120554	carbohydrate transport
Δfst4	8-day	2623420	up	GO:0008643	0,000120554	carbohydrate transport
Δfst4	8-day	2641095	up	GO:0008643	0,000120554	carbohydrate transport
Δfst4	8-day	1110887	up	GO:0004553	0,000124742	hydrolase activity, hydrolyzing O-glycosyl compounds
Δfst4	8-day	2603975	up	GO:0004553	0,000124742	hydrolase activity, hydrolyzing O-glycosyl compounds
Δfst4	8-day	2622489	up	GO:0004553	0,000124742	hydrolase activity, hydrolyzing O-glycosyl compounds
Δfst4	8-day	2622563	up	GO:0004553	0,000124742	hydrolase activity, hydrolyzing O-glycosyl compounds
Δfst4	8-day	2623407	up	GO:0004553	0,000124742	hydrolase activity, hydrolyzing O-glycosyl compounds
Δfst4	8-day	2630789	up	GO:0004553	0,000124742	hydrolase activity, hydrolyzing O-glycosyl compounds
Δfst4	8-day	2633816	up	GO:0004553	0,000124742	hydrolase activity, hydrolyzing O-glycosyl compounds
Δfst4	8-day	2645822	up	GO:0004553	0,000124742	hydrolase activity, hydrolyzing O-glycosyl compounds
Δfst4	8-day	2689018	up	GO:0004553	0,000124742	hydrolase activity, hydrolyzing O-glycosyl compounds
Δfst4	8-day	1171024	up	GO:0006810	0,000156836	transport
Δfst4	8-day	1193256	up	GO:0006810	0,000156836	transport

Δfst4	8-day	1196424	up	GO:0006810	0,000156836	transport
Δfst4	8-day	2279823	up	GO:0006810	0,000156836	transport
Δfst4	8-day	2484205	up	GO:0006810	0,000156836	transport
Δfst4	8-day	2488637	up	GO:0006810	0,000156836	transport
Δfst4	8-day	2499472	up	GO:0006810	0,000156836	transport
Δfst4	8-day	2499519	up	GO:0006810	0,000156836	transport
Δfst4	8-day	2502058	up	GO:0006810	0,000156836	transport
Δfst4	8-day	2502284	up	GO:0006810	0,000156836	transport
Δfst4	8-day	2508421	up	GO:0006810	0,000156836	transport
Δfst4	8-day	2517716	up	GO:0006810	0,000156836	transport
Δfst4	8-day	2543516	up	GO:0006810	0,000156836	transport
Δfst4	8-day	2550496	up	GO:0006810	0,000156836	transport
Δfst4	8-day	2569288	up	GO:0006810	0,000156836	transport
Δfst4	8-day	2590808	up	GO:0006810	0,000156836	transport
Δfst4	8-day	2605313	up	GO:0006810	0,000156836	transport
Δfst4	8-day	2607659	up	GO:0006810	0,000156836	transport
Δfst4	8-day	2607663	up	GO:0006810	0,000156836	transport
Δfst4	8-day	2610799	up	GO:0006810	0,000156836	transport
Δfst4	8-day	2611460	up	GO:0006810	0,000156836	transport
Δfst4	8-day	2611686	up	GO:0006810	0,000156836	transport
Δfst4	8-day	2615463	up	GO:0006810	0,000156836	transport
Δfst4	8-day	2616961	up	GO:0006810	0,000156836	transport
Δfst4	8-day	2618842	up	GO:0006810	0,000156836	transport
Δfst4	8-day	2620170	up	GO:0006810	0,000156836	transport
Δfst4	8-day	2620257	up	GO:0006810	0,000156836	transport
Δfst4	8-day	2622931	up	GO:0006810	0,000156836	transport
Δfst4	8-day	2623420	up	GO:0006810	0,000156836	transport
Δfst4	8-day	2623894	up	GO:0006810	0,000156836	transport
Δfst4	8-day	2624743	up	GO:0006810	0,000156836	transport
Δfst4	8-day	2628098	up	GO:0006810	0,000156836	transport
Δfst4	8-day	2628751	up	GO:0006810	0,000156836	transport
Δfst4	8-day	2628982	up	GO:0006810	0,000156836	transport
Δfst4	8-day	2629191	up	GO:0006810	0,000156836	transport
Δfst4	8-day	2631348	up	GO:0006810	0,000156836	transport

Δfst4	8-day	2632105	up	GO:0006810	0,000156836	transport
Δfst4	8-day	2633354	up	GO:0006810	0,000156836	transport
Δfst4	8-day	2633596	up	GO:0006810	0,000156836	transport
Δfst4	8-day	2637509	up	GO:0006810	0,000156836	transport
Δfst4	8-day	2637652	up	GO:0006810	0,000156836	transport
Δfst4	8-day	2638969	up	GO:0006810	0,000156836	transport
Δfst4	8-day	2641095	up	GO:0006810	0,000156836	transport
Δfst4	8-day	2641989	up	GO:0006810	0,000156836	transport
Δfst4	8-day	2642427	up	GO:0006810	0,000156836	transport
Δfst4	8-day	2643281	up	GO:0006810	0,000156836	transport
Δfst4	8-day	2644053	up	GO:0006810	0,000156836	transport
Δfst4	8-day	2675831	up	GO:0006810	0,000156836	transport
Δfst4	8-day	2675859	up	GO:0006810	0,000156836	transport
Δfst4	8-day	2676409	up	GO:0006810	0,000156836	transport
Δfst4	8-day	1034973	up	GO:0016021	0,000235898	integral to membrane
Δfst4	8-day	1171024	up	GO:0016021	0,000235898	integral to membrane
Δfst4	8-day	1193256	up	GO:0016021	0,000235898	integral to membrane
Δfst4	8-day	2279823	up	GO:0016021	0,000235898	integral to membrane
Δfst4	8-day	2484205	up	GO:0016021	0,000235898	integral to membrane
Δfst4	8-day	2488637	up	GO:0016021	0,000235898	integral to membrane
Δfst4	8-day	2499472	up	GO:0016021	0,000235898	integral to membrane
Δfst4	8-day	2500392	up	GO:0016021	0,000235898	integral to membrane
Δfst4	8-day	2507178	up	GO:0016021	0,000235898	integral to membrane
Δfst4	8-day	2508421	up	GO:0016021	0,000235898	integral to membrane
Δfst4	8-day	2517716	up	GO:0016021	0,000235898	integral to membrane
Δfst4	8-day	2543516	up	GO:0016021	0,000235898	integral to membrane
Δfst4	8-day	2549530	up	GO:0016021	0,000235898	integral to membrane
Δfst4	8-day	2603898	up	GO:0016021	0,000235898	integral to membrane
Δfst4	8-day	2604210	up	GO:0016021	0,000235898	integral to membrane
Δfst4	8-day	2607659	up	GO:0016021	0,000235898	integral to membrane
Δfst4	8-day	2607663	up	GO:0016021	0,000235898	integral to membrane
Δfst4	8-day	2611686	up	GO:0016021	0,000235898	integral to membrane
Δfst4	8-day	2616961	up	GO:0016021	0,000235898	integral to membrane
Δfst4	8-day	2618842	up	GO:0016021	0,000235898	integral to membrane

Δfst4	8-day	2619090	up	GO:0016021	0,000235898	integral to membrane
Δfst4	8-day	2620170	up	GO:0016021	0,000235898	integral to membrane
Δfst4	8-day	2622931	up	GO:0016021	0,000235898	integral to membrane
Δfst4	8-day	2623420	up	GO:0016021	0,000235898	integral to membrane
Δfst4	8-day	2624743	up	GO:0016021	0,000235898	integral to membrane
Δfst4	8-day	2626626	up	GO:0016021	0,000235898	integral to membrane
Δfst4	8-day	2628982	up	GO:0016021	0,000235898	integral to membrane
Δfst4	8-day	2631348	up	GO:0016021	0,000235898	integral to membrane
Δfst4	8-day	2632105	up	GO:0016021	0,000235898	integral to membrane
Δfst4	8-day	2637509	up	GO:0016021	0,000235898	integral to membrane
Δfst4	8-day	2637652	up	GO:0016021	0,000235898	integral to membrane
Δfst4	8-day	2638177	up	GO:0016021	0,000235898	integral to membrane
Δfst4	8-day	2638969	up	GO:0016021	0,000235898	integral to membrane
Δfst4	8-day	2641095	up	GO:0016021	0,000235898	integral to membrane
Δfst4	8-day	2641989	up	GO:0016021	0,000235898	integral to membrane
Δfst4	8-day	2642427	up	GO:0016021	0,000235898	integral to membrane
Δfst4	8-day	2644053	up	GO:0016021	0,000235898	integral to membrane
Δfst4	8-day	2675859	up	GO:0016021	0,000235898	integral to membrane
Δfst4	8-day	2676409	up	GO:0016021	0,000235898	integral to membrane
Δfst4	8-day	2704852	up	GO:0016021	0,000235898	integral to membrane
Δfst4	8-day	2638921	up	GO:0004091	0,011782709	carboxylesterase activity
Δfst4	8-day	17460	up	GO:0005524	0,029754011	ATP binding
Δfst4	8-day	68280	up	GO:0005524	0,029754011	ATP binding
Δfst4	8-day	83108	up	GO:0005524	0,029754011	ATP binding
Δfst4	8-day	1141641	up	GO:0005524	0,029754011	ATP binding
Δfst4	8-day	1157948	up	GO:0005524	0,029754011	ATP binding
Δfst4	8-day	1165803	up	GO:0005524	0,029754011	ATP binding
Δfst4	8-day	1166149	up	GO:0005524	0,029754011	ATP binding
Δfst4	8-day	1193256	up	GO:0005524	0,029754011	ATP binding
Δfst4	8-day	1196424	up	GO:0005524	0,029754011	ATP binding
Δfst4	8-day	2007168	up	GO:0005524	0,029754011	ATP binding
Δfst4	8-day	2018969	up	GO:0005524	0,029754011	ATP binding
Δfst4	8-day	2187522	up	GO:0005524	0,029754011	ATP binding
Δfst4	8-day	2276863	up	GO:0005524	0,029754011	ATP binding

Δfst4	8-day	2279823	up	GO:0005524	0,029754011	ATP binding
Δfst4	8-day	2481145	up	GO:0005524	0,029754011	ATP binding
Δfst4	8-day	2481375	up	GO:0005524	0,029754011	ATP binding
Δfst4	8-day	2484205	up	GO:0005524	0,029754011	ATP binding
Δfst4	8-day	2484841	up	GO:0005524	0,029754011	ATP binding
Δfst4	8-day	2485507	up	GO:0005524	0,029754011	ATP binding
Δfst4	8-day	2485669	up	GO:0005524	0,029754011	ATP binding
Δfst4	8-day	2486219	up	GO:0005524	0,029754011	ATP binding
Δfst4	8-day	2486797	up	GO:0005524	0,029754011	ATP binding
Δfst4	8-day	2489133	up	GO:0005524	0,029754011	ATP binding
Δfst4	8-day	2490024	up	GO:0005524	0,029754011	ATP binding
Δfst4	8-day	2491745	up	GO:0005524	0,029754011	ATP binding
Δfst4	8-day	2495301	up	GO:0005524	0,029754011	ATP binding
Δfst4	8-day	2501434	up	GO:0005524	0,029754011	ATP binding
Δfst4	8-day	2501652	up	GO:0005524	0,029754011	ATP binding
Δfst4	8-day	2502058	up	GO:0005524	0,029754011	ATP binding
Δfst4	8-day	2505052	up	GO:0005524	0,029754011	ATP binding
Δfst4	8-day	2510275	up	GO:0005524	0,029754011	ATP binding
Δfst4	8-day	2511765	up	GO:0005524	0,029754011	ATP binding
Δfst4	8-day	2514250	up	GO:0005524	0,029754011	ATP binding
Δfst4	8-day	2514780	up	GO:0005524	0,029754011	ATP binding
Δfst4	8-day	2517716	up	GO:0005524	0,029754011	ATP binding
Δfst4	8-day	2519304	up	GO:0005524	0,029754011	ATP binding
Δfst4	8-day	2520682	up	GO:0005524	0,029754011	ATP binding
Δfst4	8-day	2530387	up	GO:0005524	0,029754011	ATP binding
Δfst4	8-day	2536562	up	GO:0005524	0,029754011	ATP binding
Δfst4	8-day	2540501	up	GO:0005524	0,029754011	ATP binding
Δfst4	8-day	2550266	up	GO:0005524	0,029754011	ATP binding
Δfst4	8-day	2553325	up	GO:0005524	0,029754011	ATP binding
Δfst4	8-day	2557283	up	GO:0005524	0,029754011	ATP binding
Δfst4	8-day	2564434	up	GO:0005524	0,029754011	ATP binding
Δfst4	8-day	2568673	up	GO:0005524	0,029754011	ATP binding
Δfst4	8-day	2572775	up	GO:0005524	0,029754011	ATP binding
Δfst4	8-day	2574746	up	GO:0005524	0,029754011	ATP binding

Δfst4	8-day	2583917	up	GO:0005524	0,029754011	ATP binding
Δfst4	8-day	2585476	up	GO:0005524	0,029754011	ATP binding
Δfst4	8-day	2601119	up	GO:0005524	0,029754011	ATP binding
Δfst4	8-day	2604428	up	GO:0005524	0,029754011	ATP binding
Δfst4	8-day	2604805	up	GO:0005524	0,029754011	ATP binding
Δfst4	8-day	2604894	up	GO:0005524	0,029754011	ATP binding
Δfst4	8-day	2605230	up	GO:0005524	0,029754011	ATP binding
Δfst4	8-day	2605455	up	GO:0005524	0,029754011	ATP binding
Δfst4	8-day	2606752	up	GO:0005524	0,029754011	ATP binding
Δfst4	8-day	2609653	up	GO:0005524	0,029754011	ATP binding
Δfst4	8-day	2610180	up	GO:0005524	0,029754011	ATP binding
Δfst4	8-day	2610629	up	GO:0005524	0,029754011	ATP binding
Δfst4	8-day	2611700	up	GO:0005524	0,029754011	ATP binding
Δfst4	8-day	2612206	up	GO:0005524	0,029754011	ATP binding
Δfst4	8-day	2612608	up	GO:0005524	0,029754011	ATP binding
Δfst4	8-day	2614761	up	GO:0005524	0,029754011	ATP binding
Δfst4	8-day	2614894	up	GO:0005524	0,029754011	ATP binding
Δfst4	8-day	2615290	up	GO:0005524	0,029754011	ATP binding
Δfst4	8-day	2616310	up	GO:0005524	0,029754011	ATP binding
Δfst4	8-day	2616339	up	GO:0005524	0,029754011	ATP binding
Δfst4	8-day	2616964	up	GO:0005524	0,029754011	ATP binding
Δfst4	8-day	2618594	up	GO:0005524	0,029754011	ATP binding
Δfst4	8-day	2618956	up	GO:0005524	0,029754011	ATP binding
Δfst4	8-day	2619461	up	GO:0005524	0,029754011	ATP binding
Δfst4	8-day	2620364	up	GO:0005524	0,029754011	ATP binding
Δfst4	8-day	2620969	up	GO:0005524	0,029754011	ATP binding
Δfst4	8-day	2621741	up	GO:0005524	0,029754011	ATP binding
Δfst4	8-day	2621763	up	GO:0005524	0,029754011	ATP binding
Δfst4	8-day	2624145	up	GO:0005524	0,029754011	ATP binding
Δfst4	8-day	2624360	up	GO:0005524	0,029754011	ATP binding
Δfst4	8-day	2626895	up	GO:0005524	0,029754011	ATP binding
Δfst4	8-day	2627081	up	GO:0005524	0,029754011	ATP binding
Δfst4	8-day	2627181	up	GO:0005524	0,029754011	ATP binding
Δfst4	8-day	2630261	up	GO:0005524	0,029754011	ATP binding

Δfst4	8-day	2631055	up	GO:0005524	0,029754011	ATP binding
Δfst4	8-day	2631390	up	GO:0005524	0,029754011	ATP binding
Δfst4	8-day	2632216	up	GO:0005524	0,029754011	ATP binding
Δfst4	8-day	2632420	up	GO:0005524	0,029754011	ATP binding
Δfst4	8-day	2633620	up	GO:0005524	0,029754011	ATP binding
Δfst4	8-day	2633672	up	GO:0005524	0,029754011	ATP binding
Δfst4	8-day	2633881	up	GO:0005524	0,029754011	ATP binding
Δfst4	8-day	2634258	up	GO:0005524	0,029754011	ATP binding
Δfst4	8-day	2634784	up	GO:0005524	0,029754011	ATP binding
Δfst4	8-day	2636406	up	GO:0005524	0,029754011	ATP binding
Δfst4	8-day	2638762	up	GO:0005524	0,029754011	ATP binding
Δfst4	8-day	2638986	up	GO:0005524	0,029754011	ATP binding
Δfst4	8-day	2642427	up	GO:0005524	0,029754011	ATP binding
Δfst4	8-day	2643236	up	GO:0005524	0,029754011	ATP binding
Δfst4	8-day	2645192	up	GO:0005524	0,029754011	ATP binding
Δfst4	8-day	2645217	up	GO:0005524	0,029754011	ATP binding
Δfst4	8-day	2662599	up	GO:0005524	0,029754011	ATP binding
Δfst4	8-day	2671089	up	GO:0005524	0,029754011	ATP binding
Δfst4	8-day	2672807	up	GO:0005524	0,029754011	ATP binding
Δfst4	8-day	2674869	up	GO:0005524	0,029754011	ATP binding
Δfst4	8-day	2675859	up	GO:0005524	0,029754011	ATP binding
Δfst4	8-day	2676409	up	GO:0005524	0,029754011	ATP binding
Δfst4	8-day	2681506	up	GO:0005524	0,029754011	ATP binding
Δfst4	8-day	2689581	up	GO:0005524	0,029754011	ATP binding
Δfst4	8-day	2691288	up	GO:0005524	0,029754011	ATP binding
Δfst4	8-day	2706463	up	GO:0005524	0,029754011	ATP binding
Δfst4	8-day	2745977	up	GO:0005524	0,029754011	ATP binding
Δfst4	8-day	2750974	up	GO:0005524	0,029754011	ATP binding
Δfst4	8-day	2753335	up	GO:0005524	0,029754011	ATP binding
Δfst4	8-day	1120318	up	GO:0005506	0,044746953	iron ion binding
Δfst4	8-day	1193647	up	GO:0005506	0,044746953	iron ion binding
Δfst4	8-day	2376858	up	GO:0005506	0,044746953	iron ion binding
Δfst4	8-day	2487208	up	GO:0005506	0,044746953	iron ion binding
Δfst4	8-day	2493935	up	GO:0005506	0,044746953	iron ion binding

Δfst4	8-day	2497674	up	GO:0005506	0,044746953	iron ion binding
Δfst4	8-day	2501258	up	GO:0005506	0,044746953	iron ion binding
Δfst4	8-day	2517480	up	GO:0005506	0,044746953	iron ion binding
Δfst4	8-day	2604096	up	GO:0005506	0,044746953	iron ion binding
Δfst4	8-day	2604458	up	GO:0005506	0,044746953	iron ion binding
Δfst4	8-day	2609502	up	GO:0005506	0,044746953	iron ion binding
Δfst4	8-day	2618018	up	GO:0005506	0,044746953	iron ion binding
Δfst4	8-day	2620407	up	GO:0005506	0,044746953	iron ion binding
Δfst4	8-day	2623115	up	GO:0005506	0,044746953	iron ion binding
Δfst4	8-day	2623122	up	GO:0005506	0,044746953	iron ion binding
Δfst4	8-day	2623189	up	GO:0005506	0,044746953	iron ion binding
Δfst4	8-day	2623701	up	GO:0005506	0,044746953	iron ion binding
Δfst4	8-day	2624973	up	GO:0005506	0,044746953	iron ion binding
Δfst4	8-day	2626362	up	GO:0005506	0,044746953	iron ion binding
Δfst4	8-day	2627525	up	GO:0005506	0,044746953	iron ion binding
Δfst4	8-day	2633776	up	GO:0005506	0,044746953	iron ion binding
Δfst4	8-day	2635869	up	GO:0005506	0,044746953	iron ion binding
Δfst4	8-day	2635870	up	GO:0005506	0,044746953	iron ion binding
Δfst4	8-day	2636160	up	GO:0005506	0,044746953	iron ion binding
Δfst4	8-day	2644002	up	GO:0005506	0,044746953	iron ion binding
Δfst4	8-day	2666440	up	GO:0005506	0,044746953	iron ion binding
Δfst4	8-day	2732977	up	GO:0005506	0,044746953	iron ion binding
Δfst4	8-day	15246	down	GO:0005524	2,67E-12	ATP binding
Δfst4	8-day	63296	down	GO:0005524	2,67E-12	ATP binding
Δfst4	8-day	1127007	down	GO:0005524	2,67E-12	ATP binding
Δfst4	8-day	1131365	down	GO:0005524	2,67E-12	ATP binding
Δfst4	8-day	2206419	down	GO:0005524	2,67E-12	ATP binding
Δfst4	8-day	2212909	down	GO:0005524	2,67E-12	ATP binding
Δfst4	8-day	2483083	down	GO:0005524	2,67E-12	ATP binding
Δfst4	8-day	2493870	down	GO:0005524	2,67E-12	ATP binding
Δfst4	8-day	2504654	down	GO:0005524	2,67E-12	ATP binding
Δfst4	8-day	2512873	down	GO:0005524	2,67E-12	ATP binding
Δfst4	8-day	2535618	down	GO:0005524	2,67E-12	ATP binding
Δfst4	8-day	2553433	down	GO:0005524	2,67E-12	ATP binding

Δfst4	8-day	2563594	down	GO:0005524	2,67E-12	ATP binding
Δfst4	8-day	2565471	down	GO:0005524	2,67E-12	ATP binding
Δfst4	8-day	2610226	down	GO:0005524	2,67E-12	ATP binding
Δfst4	8-day	2616602	down	GO:0005524	2,67E-12	ATP binding
Δfst4	8-day	2621253	down	GO:0005524	2,67E-12	ATP binding
Δfst4	8-day	2622087	down	GO:0005524	2,67E-12	ATP binding
Δfst4	8-day	2625496	down	GO:0005524	2,67E-12	ATP binding
Δfst4	8-day	2629497	down	GO:0005524	2,67E-12	ATP binding
Δfst4	8-day	2633314	down	GO:0005524	2,67E-12	ATP binding
Δfst4	8-day	2636918	down	GO:0005524	2,67E-12	ATP binding
Δfst4	8-day	2642188	down	GO:0005524	2,67E-12	ATP binding
Δfst4	8-day	2661112	down	GO:0005524	2,67E-12	ATP binding
Δfst4	8-day	2684679	down	GO:0005524	2,67E-12	ATP binding
Δfst4	8-day	2690708	down	GO:0005524	2,67E-12	ATP binding
Δfst4	8-day	2695021	down	GO:0005524	2,67E-12	ATP binding
Δfst4	8-day	2748506	down	GO:0005524	2,67E-12	ATP binding
Δfst4	8-day	1120237	down	GO:0003677	1,94E-08	DNA binding
Δfst4	8-day	1136828	down	GO:0003677	1,94E-08	DNA binding
Δfst4	8-day	1192535	down	GO:0003677	1,94E-08	DNA binding
Δfst4	8-day	2467966	down	GO:0003677	1,94E-08	DNA binding
Δfst4	8-day	2510481	down	GO:0003677	1,94E-08	DNA binding
Δfst4	8-day	2596857	down	GO:0003677	1,94E-08	DNA binding
Δfst4	8-day	2605931	down	GO:0003677	1,94E-08	DNA binding
Δfst4	8-day	2607126	down	GO:0003677	1,94E-08	DNA binding
Δfst4	8-day	2608348	down	GO:0003677	1,94E-08	DNA binding
Δfst4	8-day	2609231	down	GO:0003677	1,94E-08	DNA binding
Δfst4	8-day	2615561	down	GO:0003677	1,94E-08	DNA binding
Δfst4	8-day	2620175	down	GO:0003677	1,94E-08	DNA binding
Δfst4	8-day	2622267	down	GO:0003677	1,94E-08	DNA binding
Δfst4	8-day	2623333	down	GO:0003677	1,94E-08	DNA binding
Δfst4	8-day	2625496	down	GO:0003677	1,94E-08	DNA binding
Δfst4	8-day	2625706	down	GO:0003677	1,94E-08	DNA binding
Δfst4	8-day	2629497	down	GO:0003677	1,94E-08	DNA binding
Δfst4	8-day	2703742	down	GO:0003677	1,94E-08	DNA binding

Δfst4	8-day	1127007	down	GO:0000166	2,57E-08	nucleotide binding
Δfst4	8-day	2493870	down	GO:0000166	2,57E-08	nucleotide binding
Δfst4	8-day	2504654	down	GO:0000166	2,57E-08	nucleotide binding
Δfst4	8-day	2512873	down	GO:0000166	2,57E-08	nucleotide binding
Δfst4	8-day	2565471	down	GO:0000166	2,57E-08	nucleotide binding
Δfst4	8-day	2661112	down	GO:0000166	2,57E-08	nucleotide binding
Δfst4	8-day	2748506	down	GO:0000166	2,57E-08	nucleotide binding
Δfst4	8-day	1131965	down	GO:0008152	2,16E-07	metabolic process
Δfst4	8-day	1152293	down	GO:0008152	2,16E-07	metabolic process
Δfst4	8-day	1156481	down	GO:0008152	2,16E-07	metabolic process
Δfst4	8-day	1193333	down	GO:0008152	2,16E-07	metabolic process
Δfst4	8-day	1200798	down	GO:0008152	2,16E-07	metabolic process
Δfst4	8-day	2072306	down	GO:0008152	2,16E-07	metabolic process
Δfst4	8-day	2110046	down	GO:0008152	2,16E-07	metabolic process
Δfst4	8-day	2135332	down	GO:0008152	2,16E-07	metabolic process
Δfst4	8-day	2333133	down	GO:0008152	2,16E-07	metabolic process
Δfst4	8-day	2350414	down	GO:0008152	2,16E-07	metabolic process
Δfst4	8-day	2373661	down	GO:0008152	2,16E-07	metabolic process
Δfst4	8-day	2485727	down	GO:0008152	2,16E-07	metabolic process
Δfst4	8-day	2492878	down	GO:0008152	2,16E-07	metabolic process
Δfst4	8-day	2495855	down	GO:0008152	2,16E-07	metabolic process
Δfst4	8-day	2505594	down	GO:0008152	2,16E-07	metabolic process
Δfst4	8-day	2510775	down	GO:0008152	2,16E-07	metabolic process
Δfst4	8-day	2511020	down	GO:0008152	2,16E-07	metabolic process
Δfst4	8-day	2515850	down	GO:0008152	2,16E-07	metabolic process
Δfst4	8-day	2530601	down	GO:0008152	2,16E-07	metabolic process
Δfst4	8-day	2533120	down	GO:0008152	2,16E-07	metabolic process
Δfst4	8-day	2534187	down	GO:0008152	2,16E-07	metabolic process
Δfst4	8-day	2572586	down	GO:0008152	2,16E-07	metabolic process
Δfst4	8-day	2575853	down	GO:0008152	2,16E-07	metabolic process
Δfst4	8-day	2587289	down	GO:0008152	2,16E-07	metabolic process
Δfst4	8-day	2591025	down	GO:0008152	2,16E-07	metabolic process
Δfst4	8-day	2591516	down	GO:0008152	2,16E-07	metabolic process
Δfst4	8-day	2595361	down	GO:0008152	2,16E-07	metabolic process

Δfst4	8-day	2601905	down	GO:0008152	2,16E-07	metabolic process
Δfst4	8-day	2604699	down	GO:0008152	2,16E-07	metabolic process
Δfst4	8-day	2604917	down	GO:0008152	2,16E-07	metabolic process
Δfst4	8-day	2608380	down	GO:0008152	2,16E-07	metabolic process
Δfst4	8-day	2608416	down	GO:0008152	2,16E-07	metabolic process
Δfst4	8-day	2609281	down	GO:0008152	2,16E-07	metabolic process
Δfst4	8-day	2610587	down	GO:0008152	2,16E-07	metabolic process
Δfst4	8-day	2610599	down	GO:0008152	2,16E-07	metabolic process
Δfst4	8-day	2611001	down	GO:0008152	2,16E-07	metabolic process
Δfst4	8-day	2611306	down	GO:0008152	2,16E-07	metabolic process
Δfst4	8-day	2612591	down	GO:0008152	2,16E-07	metabolic process
Δfst4	8-day	2615529	down	GO:0008152	2,16E-07	metabolic process
Δfst4	8-day	2615598	down	GO:0008152	2,16E-07	metabolic process
Δfst4	8-day	2616264	down	GO:0008152	2,16E-07	metabolic process
Δfst4	8-day	2616512	down	GO:0008152	2,16E-07	metabolic process
Δfst4	8-day	2616602	down	GO:0008152	2,16E-07	metabolic process
Δfst4	8-day	2616676	down	GO:0008152	2,16E-07	metabolic process
Δfst4	8-day	2622087	down	GO:0008152	2,16E-07	metabolic process
Δfst4	8-day	2622385	down	GO:0008152	2,16E-07	metabolic process
Δfst4	8-day	2623062	down	GO:0008152	2,16E-07	metabolic process
Δfst4	8-day	2623103	down	GO:0008152	2,16E-07	metabolic process
Δfst4	8-day	2623956	down	GO:0008152	2,16E-07	metabolic process
Δfst4	8-day	2625208	down	GO:0008152	2,16E-07	metabolic process
Δfst4	8-day	2625706	down	GO:0008152	2,16E-07	metabolic process
Δfst4	8-day	2625782	down	GO:0008152	2,16E-07	metabolic process
Δfst4	8-day	2627113	down	GO:0008152	2,16E-07	metabolic process
Δfst4	8-day	2629497	down	GO:0008152	2,16E-07	metabolic process
Δfst4	8-day	2629526	down	GO:0008152	2,16E-07	metabolic process
Δfst4	8-day	2629548	down	GO:0008152	2,16E-07	metabolic process
Δfst4	8-day	2630590	down	GO:0008152	2,16E-07	metabolic process
Δfst4	8-day	2631112	down	GO:0008152	2,16E-07	metabolic process
Δfst4	8-day	2631368	down	GO:0008152	2,16E-07	metabolic process
Δfst4	8-day	2631774	down	GO:0008152	2,16E-07	metabolic process
Δfst4	8-day	2632611	down	GO:0008152	2,16E-07	metabolic process

Δfst4	8-day	2633167	down	GO:0008152	2,16E-07	metabolic process
Δfst4	8-day	2633644	down	GO:0008152	2,16E-07	metabolic process
Δfst4	8-day	2633989	down	GO:0008152	2,16E-07	metabolic process
Δfst4	8-day	2634044	down	GO:0008152	2,16E-07	metabolic process
Δfst4	8-day	2635567	down	GO:0008152	2,16E-07	metabolic process
Δfst4	8-day	2636283	down	GO:0008152	2,16E-07	metabolic process
Δfst4	8-day	2637104	down	GO:0008152	2,16E-07	metabolic process
Δfst4	8-day	2637221	down	GO:0008152	2,16E-07	metabolic process
Δfst4	8-day	2637668	down	GO:0008152	2,16E-07	metabolic process
Δfst4	8-day	2638322	down	GO:0008152	2,16E-07	metabolic process
Δfst4	8-day	2641506	down	GO:0008152	2,16E-07	metabolic process
Δfst4	8-day	2641520	down	GO:0008152	2,16E-07	metabolic process
Δfst4	8-day	2641761	down	GO:0008152	2,16E-07	metabolic process
Δfst4	8-day	2644309	down	GO:0008152	2,16E-07	metabolic process
Δfst4	8-day	2645207	down	GO:0008152	2,16E-07	metabolic process
Δfst4	8-day	2663997	down	GO:0008152	2,16E-07	metabolic process
Δfst4	8-day	2666990	down	GO:0008152	2,16E-07	metabolic process
Δfst4	8-day	2671785	down	GO:0008152	2,16E-07	metabolic process
Δfst4	8-day	2681358	down	GO:0008152	2,16E-07	metabolic process
Δfst4	8-day	2693285	down	GO:0008152	2,16E-07	metabolic process
Δfst4	8-day	2696676	down	GO:0008152	2,16E-07	metabolic process
Δfst4	8-day	2699810	down	GO:0008152	2,16E-07	metabolic process
Δfst4	8-day	2738806	down	GO:0008152	2,16E-07	metabolic process
Δfst4	8-day	1127007	down	GO:0017111	3,53E-06	nucleoside-triphosphatase activity
Δfst4	8-day	2493870	down	GO:0017111	3,53E-06	nucleoside-triphosphatase activity
Δfst4	8-day	2565471	down	GO:0017111	3,53E-06	nucleoside-triphosphatase activity
Δfst4	8-day	2661112	down	GO:0017111	3,53E-06	nucleoside-triphosphatase activity
Δfst4	8-day	1093363	down	GO:0003824	7,43E-06	catalytic activity
Δfst4	8-day	1131965	down	GO:0003824	7,43E-06	catalytic activity
Δfst4	8-day	1152293	down	GO:0003824	7,43E-06	catalytic activity
Δfst4	8-day	1158760	down	GO:0003824	7,43E-06	catalytic activity
Δfst4	8-day	1160309	down	GO:0003824	7,43E-06	catalytic activity
Δfst4	8-day	1185226	down	GO:0003824	7,43E-06	catalytic activity
Δfst4	8-day	1191999	down	GO:0003824	7,43E-06	catalytic activity

Δfst4	8-day	1193333	down	GO:0003824	7,43E-06	catalytic activity
Δfst4	8-day	1200798	down	GO:0003824	7,43E-06	catalytic activity
Δfst4	8-day	2055308	down	GO:0003824	7,43E-06	catalytic activity
Δfst4	8-day	2072306	down	GO:0003824	7,43E-06	catalytic activity
Δfst4	8-day	2110046	down	GO:0003824	7,43E-06	catalytic activity
Δfst4	8-day	2135332	down	GO:0003824	7,43E-06	catalytic activity
Δfst4	8-day	2333133	down	GO:0003824	7,43E-06	catalytic activity
Δfst4	8-day	2350414	down	GO:0003824	7,43E-06	catalytic activity
Δfst4	8-day	2373661	down	GO:0003824	7,43E-06	catalytic activity
Δfst4	8-day	2492878	down	GO:0003824	7,43E-06	catalytic activity
Δfst4	8-day	2495855	down	GO:0003824	7,43E-06	catalytic activity
Δfst4	8-day	2510775	down	GO:0003824	7,43E-06	catalytic activity
Δfst4	8-day	2511020	down	GO:0003824	7,43E-06	catalytic activity
Δfst4	8-day	2512030	down	GO:0003824	7,43E-06	catalytic activity
Δfst4	8-day	2512873	down	GO:0003824	7,43E-06	catalytic activity
Δfst4	8-day	2516444	down	GO:0003824	7,43E-06	catalytic activity
Δfst4	8-day	2530601	down	GO:0003824	7,43E-06	catalytic activity
Δfst4	8-day	2557656	down	GO:0003824	7,43E-06	catalytic activity
Δfst4	8-day	2572586	down	GO:0003824	7,43E-06	catalytic activity
Δfst4	8-day	2575853	down	GO:0003824	7,43E-06	catalytic activity
Δfst4	8-day	2587289	down	GO:0003824	7,43E-06	catalytic activity
Δfst4	8-day	2591516	down	GO:0003824	7,43E-06	catalytic activity
Δfst4	8-day	2595361	down	GO:0003824	7,43E-06	catalytic activity
Δfst4	8-day	2598960	down	GO:0003824	7,43E-06	catalytic activity
Δfst4	8-day	2604699	down	GO:0003824	7,43E-06	catalytic activity
Δfst4	8-day	2608014	down	GO:0003824	7,43E-06	catalytic activity
Δfst4	8-day	2609281	down	GO:0003824	7,43E-06	catalytic activity
Δfst4	8-day	2610587	down	GO:0003824	7,43E-06	catalytic activity
Δfst4	8-day	2610599	down	GO:0003824	7,43E-06	catalytic activity
Δfst4	8-day	2612591	down	GO:0003824	7,43E-06	catalytic activity
Δfst4	8-day	2614163	down	GO:0003824	7,43E-06	catalytic activity
Δfst4	8-day	2615529	down	GO:0003824	7,43E-06	catalytic activity
Δfst4	8-day	2615598	down	GO:0003824	7,43E-06	catalytic activity
Δfst4	8-day	2616264	down	GO:0003824	7,43E-06	catalytic activity

Δfst4	8-day	2616491	down	GO:0003824	7,43E-06	catalytic activity
Δfst4	8-day	2616512	down	GO:0003824	7,43E-06	catalytic activity
Δfst4	8-day	2616602	down	GO:0003824	7,43E-06	catalytic activity
Δfst4	8-day	2616873	down	GO:0003824	7,43E-06	catalytic activity
Δfst4	8-day	2620185	down	GO:0003824	7,43E-06	catalytic activity
Δfst4	8-day	2623062	down	GO:0003824	7,43E-06	catalytic activity
Δfst4	8-day	2623103	down	GO:0003824	7,43E-06	catalytic activity
Δfst4	8-day	2625782	down	GO:0003824	7,43E-06	catalytic activity
Δfst4	8-day	2626529	down	GO:0003824	7,43E-06	catalytic activity
Δfst4	8-day	2626643	down	GO:0003824	7,43E-06	catalytic activity
Δfst4	8-day	2627113	down	GO:0003824	7,43E-06	catalytic activity
Δfst4	8-day	2629526	down	GO:0003824	7,43E-06	catalytic activity
Δfst4	8-day	2629548	down	GO:0003824	7,43E-06	catalytic activity
Δfst4	8-day	2630590	down	GO:0003824	7,43E-06	catalytic activity
Δfst4	8-day	2630634	down	GO:0003824	7,43E-06	catalytic activity
Δfst4	8-day	2630806	down	GO:0003824	7,43E-06	catalytic activity
Δfst4	8-day	2631112	down	GO:0003824	7,43E-06	catalytic activity
Δfst4	8-day	2631368	down	GO:0003824	7,43E-06	catalytic activity
Δfst4	8-day	2631634	down	GO:0003824	7,43E-06	catalytic activity
Δfst4	8-day	2631920	down	GO:0003824	7,43E-06	catalytic activity
Δfst4	8-day	2632611	down	GO:0003824	7,43E-06	catalytic activity
Δfst4	8-day	2633144	down	GO:0003824	7,43E-06	catalytic activity
Δfst4	8-day	2633167	down	GO:0003824	7,43E-06	catalytic activity
Δfst4	8-day	2635216	down	GO:0003824	7,43E-06	catalytic activity
Δfst4	8-day	2635567	down	GO:0003824	7,43E-06	catalytic activity
Δfst4	8-day	2636283	down	GO:0003824	7,43E-06	catalytic activity
Δfst4	8-day	2636333	down	GO:0003824	7,43E-06	catalytic activity
Δfst4	8-day	2636417	down	GO:0003824	7,43E-06	catalytic activity
Δfst4	8-day	2636420	down	GO:0003824	7,43E-06	catalytic activity
Δfst4	8-day	2636681	down	GO:0003824	7,43E-06	catalytic activity
Δfst4	8-day	2637104	down	GO:0003824	7,43E-06	catalytic activity
Δfst4	8-day	2637668	down	GO:0003824	7,43E-06	catalytic activity
Δfst4	8-day	2637845	down	GO:0003824	7,43E-06	catalytic activity
Δfst4	8-day	2638783	down	GO:0003824	7,43E-06	catalytic activity

Δfst4	8-day	2638893	down	GO:0003824	7,43E-06	catalytic activity
Δfst4	8-day	2641506	down	GO:0003824	7,43E-06	catalytic activity
Δfst4	8-day	2641520	down	GO:0003824	7,43E-06	catalytic activity
Δfst4	8-day	2642057	down	GO:0003824	7,43E-06	catalytic activity
Δfst4	8-day	2642275	down	GO:0003824	7,43E-06	catalytic activity
Δfst4	8-day	2642438	down	GO:0003824	7,43E-06	catalytic activity
Δfst4	8-day	2644309	down	GO:0003824	7,43E-06	catalytic activity
Δfst4	8-day	2645207	down	GO:0003824	7,43E-06	catalytic activity
Δfst4	8-day	2663997	down	GO:0003824	7,43E-06	catalytic activity
Δfst4	8-day	2666990	down	GO:0003824	7,43E-06	catalytic activity
Δfst4	8-day	2676633	down	GO:0003824	7,43E-06	catalytic activity
Δfst4	8-day	2681358	down	GO:0003824	7,43E-06	catalytic activity
Δfst4	8-day	2684249	down	GO:0003824	7,43E-06	catalytic activity
Δfst4	8-day	2693285	down	GO:0003824	7,43E-06	catalytic activity
Δfst4	8-day	2699217	down	GO:0003824	7,43E-06	catalytic activity
Δfst4	8-day	2504654	down	GO:0005737	8,55E-06	cytoplasm
Δfst4	8-day	2512873	down	GO:0005737	8,55E-06	cytoplasm
Δfst4	8-day	2607695	down	GO:0005737	8,55E-06	cytoplasm
Δfst4	8-day	2748506	down	GO:0005737	8,55E-06	cytoplasm
Δfst4	8-day	257768	down	GO:0016491	4,54E-05	oxidoreductase activity
Δfst4	8-day	1131965	down	GO:0016491	4,54E-05	oxidoreductase activity
Δfst4	8-day	1147332	down	GO:0016491	4,54E-05	oxidoreductase activity
Δfst4	8-day	1152293	down	GO:0016491	4,54E-05	oxidoreductase activity
Δfst4	8-day	1156481	down	GO:0016491	4,54E-05	oxidoreductase activity
Δfst4	8-day	1160309	down	GO:0016491	4,54E-05	oxidoreductase activity
Δfst4	8-day	1350659	down	GO:0016491	4,54E-05	oxidoreductase activity
Δfst4	8-day	2072306	down	GO:0016491	4,54E-05	oxidoreductase activity
Δfst4	8-day	2110046	down	GO:0016491	4,54E-05	oxidoreductase activity
Δfst4	8-day	2333133	down	GO:0016491	4,54E-05	oxidoreductase activity
Δfst4	8-day	2495855	down	GO:0016491	4,54E-05	oxidoreductase activity
Δfst4	8-day	2498327	down	GO:0016491	4,54E-05	oxidoreductase activity
Δfst4	8-day	2503108	down	GO:0016491	4,54E-05	oxidoreductase activity
Δfst4	8-day	2504654	down	GO:0016491	4,54E-05	oxidoreductase activity
Δfst4	8-day	2515850	down	GO:0016491	4,54E-05	oxidoreductase activity

Δfst4	8-day	2516444	down	GO:0016491	4,54E-05	oxidoreductase activity
Δfst4	8-day	2532967	down	GO:0016491	4,54E-05	oxidoreductase activity
Δfst4	8-day	2534187	down	GO:0016491	4,54E-05	oxidoreductase activity
Δfst4	8-day	2575853	down	GO:0016491	4,54E-05	oxidoreductase activity
Δfst4	8-day	2587289	down	GO:0016491	4,54E-05	oxidoreductase activity
Δfst4	8-day	2591025	down	GO:0016491	4,54E-05	oxidoreductase activity
Δfst4	8-day	2591516	down	GO:0016491	4,54E-05	oxidoreductase activity
Δfst4	8-day	2603449	down	GO:0016491	4,54E-05	oxidoreductase activity
Δfst4	8-day	2604699	down	GO:0016491	4,54E-05	oxidoreductase activity
Δfst4	8-day	2609281	down	GO:0016491	4,54E-05	oxidoreductase activity
Δfst4	8-day	2610587	down	GO:0016491	4,54E-05	oxidoreductase activity
Δfst4	8-day	2610599	down	GO:0016491	4,54E-05	oxidoreductase activity
Δfst4	8-day	2611001	down	GO:0016491	4,54E-05	oxidoreductase activity
Δfst4	8-day	2611263	down	GO:0016491	4,54E-05	oxidoreductase activity
Δfst4	8-day	2612591	down	GO:0016491	4,54E-05	oxidoreductase activity
Δfst4	8-day	2614858	down	GO:0016491	4,54E-05	oxidoreductase activity
Δfst4	8-day	2615184	down	GO:0016491	4,54E-05	oxidoreductase activity
Δfst4	8-day	2622733	down	GO:0016491	4,54E-05	oxidoreductase activity
Δfst4	8-day	2624407	down	GO:0016491	4,54E-05	oxidoreductase activity
Δfst4	8-day	2625208	down	GO:0016491	4,54E-05	oxidoreductase activity
Δfst4	8-day	2625917	down	GO:0016491	4,54E-05	oxidoreductase activity
Δfst4	8-day	2627113	down	GO:0016491	4,54E-05	oxidoreductase activity
Δfst4	8-day	2628264	down	GO:0016491	4,54E-05	oxidoreductase activity
Δfst4	8-day	2629526	down	GO:0016491	4,54E-05	oxidoreductase activity
Δfst4	8-day	2630033	down	GO:0016491	4,54E-05	oxidoreductase activity
Δfst4	8-day	2630590	down	GO:0016491	4,54E-05	oxidoreductase activity
Δfst4	8-day	2631774	down	GO:0016491	4,54E-05	oxidoreductase activity
Δfst4	8-day	2633167	down	GO:0016491	4,54E-05	oxidoreductase activity
Δfst4	8-day	2634044	down	GO:0016491	4,54E-05	oxidoreductase activity
Δfst4	8-day	2635567	down	GO:0016491	4,54E-05	oxidoreductase activity
Δfst4	8-day	2637104	down	GO:0016491	4,54E-05	oxidoreductase activity
Δfst4	8-day	2638893	down	GO:0016491	4,54E-05	oxidoreductase activity
Δfst4	8-day	2641506	down	GO:0016491	4,54E-05	oxidoreductase activity
Δfst4	8-day	2641520	down	GO:0016491	4,54E-05	oxidoreductase activity

Δfst4	8-day	2642438	down	GO:0016491	4,54E-05	oxidoreductase activity
Δfst4	8-day	2661224	down	GO:0016491	4,54E-05	oxidoreductase activity
Δfst4	8-day	2671386	down	GO:0016491	4,54E-05	oxidoreductase activity
Δfst4	8-day	2671785	down	GO:0016491	4,54E-05	oxidoreductase activity
Δfst4	8-day	2743308	down	GO:0016491	4,54E-05	oxidoreductase activity
Δfst4	8-day	2493870	down	GO:0016887	6,16E-05	ATPase activity
Δfst4	8-day	2565471	down	GO:0016887	6,16E-05	ATPase activity
Δfst4	8-day	2661112	down	GO:0016887	6,16E-05	ATPase activity
Δfst4	8-day	2677580	down	GO:0016887	6,16E-05	ATPase activity
Δfst4	8-day	2695021	down	GO:0016887	6,16E-05	ATPase activity
Δfst4	8-day	2623956	down	GO:0030170	0,000350904	pyridoxal phosphate binding
Δfst4	8-day	2632714	down	GO:0030170	0,000350904	pyridoxal phosphate binding
Δfst4	8-day	2636719	down	GO:0030170	0,000350904	pyridoxal phosphate binding
Δfst4	8-day	2663997	down	GO:0030170	0,000350904	pyridoxal phosphate binding
Δfst4	8-day	78628	down	GO:0020037	0,000672113	heme binding
Δfst4	8-day	1131542	down	GO:0020037	0,000672113	heme binding
Δfst4	8-day	1159597	down	GO:0020037	0,000672113	heme binding
Δfst4	8-day	1189794	down	GO:0020037	0,000672113	heme binding
Δfst4	8-day	2486680	down	GO:0020037	0,000672113	heme binding
Δfst4	8-day	2509716	down	GO:0020037	0,000672113	heme binding
Δfst4	8-day	2516208	down	GO:0020037	0,000672113	heme binding
Δfst4	8-day	2559987	down	GO:0020037	0,000672113	heme binding
Δfst4	8-day	2594763	down	GO:0020037	0,000672113	heme binding
Δfst4	8-day	2604735	down	GO:0020037	0,000672113	heme binding
Δfst4	8-day	2611771	down	GO:0020037	0,000672113	heme binding
Δfst4	8-day	2614692	down	GO:0020037	0,000672113	heme binding
Δfst4	8-day	2614998	down	GO:0020037	0,000672113	heme binding
Δfst4	8-day	2616590	down	GO:0020037	0,000672113	heme binding
Δfst4	8-day	2619674	down	GO:0020037	0,000672113	heme binding
Δfst4	8-day	2619910	down	GO:0020037	0,000672113	heme binding
Δfst4	8-day	2621855	down	GO:0020037	0,000672113	heme binding
Δfst4	8-day	2628312	down	GO:0020037	0,000672113	heme binding
Δfst4	8-day	2628651	down	GO:0020037	0,000672113	heme binding
Δfst4	8-day	2634186	down	GO:0020037	0,000672113	heme binding

Δfst4	8-day	2635487	down	GO:0020037	0,000672113	heme binding
Δfst4	8-day	2638355	down	GO:0020037	0,000672113	heme binding
Δfst4	8-day	2703965	down	GO:0020037	0,000672113	heme binding
Δfst4	8-day	2703966	down	GO:0020037	0,000672113	heme binding
Δfst4	8-day	2705258	down	GO:0020037	0,000672113	heme binding
Δfst4	8-day	70808	down	GO:0005634	0,001013596	nucleus
Δfst4	8-day	250298	down	GO:0005634	0,001013596	nucleus
Δfst4	8-day	1156706	down	GO:0005634	0,001013596	nucleus
Δfst4	8-day	2151391	down	GO:0005634	0,001013596	nucleus
Δfst4	8-day	2510481	down	GO:0005634	0,001013596	nucleus
Δfst4	8-day	2515078	down	GO:0005634	0,001013596	nucleus
Δfst4	8-day	2596857	down	GO:0005634	0,001013596	nucleus
Δfst4	8-day	2598013	down	GO:0005634	0,001013596	nucleus
Δfst4	8-day	2605931	down	GO:0005634	0,001013596	nucleus
Δfst4	8-day	2608180	down	GO:0005634	0,001013596	nucleus
Δfst4	8-day	2608348	down	GO:0005634	0,001013596	nucleus
Δfst4	8-day	2609231	down	GO:0005634	0,001013596	nucleus
Δfst4	8-day	2610202	down	GO:0005634	0,001013596	nucleus
Δfst4	8-day	2613385	down	GO:0005634	0,001013596	nucleus
Δfst4	8-day	2615561	down	GO:0005634	0,001013596	nucleus
Δfst4	8-day	2620175	down	GO:0005634	0,001013596	nucleus
Δfst4	8-day	2625496	down	GO:0005634	0,001013596	nucleus
Δfst4	8-day	2627369	down	GO:0005634	0,001013596	nucleus
Δfst4	8-day	2632395	down	GO:0005634	0,001013596	nucleus
Δfst4	8-day	2642040	down	GO:0005634	0,001013596	nucleus
Δfst4	8-day	2645939	down	GO:0005634	0,001013596	nucleus
Δfst4	8-day	2689617	down	GO:0005634	0,001013596	nucleus
Δfst4	8-day	2703742	down	GO:0005634	0,001013596	nucleus
Δfst4	8-day	2746123	down	GO:0005634	0,001013596	nucleus
Δfst4	8-day	1077503	down	GO:0006810	0,001159639	transport
Δfst4	8-day	1100962	down	GO:0006810	0,001159639	transport
Δfst4	8-day	1129508	down	GO:0006810	0,001159639	transport
Δfst4	8-day	1144263	down	GO:0006810	0,001159639	transport
Δfst4	8-day	1147635	down	GO:0006810	0,001159639	transport

Δfst4	8-day	1154281	down	GO:0006810	0,001159639	transport
Δfst4	8-day	1175357	down	GO:0006810	0,001159639	transport
Δfst4	8-day	1245995	down	GO:0006810	0,001159639	transport
Δfst4	8-day	1357952	down	GO:0006810	0,001159639	transport
Δfst4	8-day	2138287	down	GO:0006810	0,001159639	transport
Δfst4	8-day	2162840	down	GO:0006810	0,001159639	transport
Δfst4	8-day	2481653	down	GO:0006810	0,001159639	transport
Δfst4	8-day	2491665	down	GO:0006810	0,001159639	transport
Δfst4	8-day	2492878	down	GO:0006810	0,001159639	transport
Δfst4	8-day	2493264	down	GO:0006810	0,001159639	transport
Δfst4	8-day	2501978	down	GO:0006810	0,001159639	transport
Δfst4	8-day	2504863	down	GO:0006810	0,001159639	transport
Δfst4	8-day	2509716	down	GO:0006810	0,001159639	transport
Δfst4	8-day	2514012	down	GO:0006810	0,001159639	transport
Δfst4	8-day	2519917	down	GO:0006810	0,001159639	transport
Δfst4	8-day	2543225	down	GO:0006810	0,001159639	transport
Δfst4	8-day	2546372	down	GO:0006810	0,001159639	transport
Δfst4	8-day	2550918	down	GO:0006810	0,001159639	transport
Δfst4	8-day	2564524	down	GO:0006810	0,001159639	transport
Δfst4	8-day	2565471	down	GO:0006810	0,001159639	transport
Δfst4	8-day	2600551	down	GO:0006810	0,001159639	transport
Δfst4	8-day	2609987	down	GO:0006810	0,001159639	transport
Δfst4	8-day	2612313	down	GO:0006810	0,001159639	transport
Δfst4	8-day	2616189	down	GO:0006810	0,001159639	transport
Δfst4	8-day	2616602	down	GO:0006810	0,001159639	transport
Δfst4	8-day	2617636	down	GO:0006810	0,001159639	transport
Δfst4	8-day	2617876	down	GO:0006810	0,001159639	transport
Δfst4	8-day	2618539	down	GO:0006810	0,001159639	transport
Δfst4	8-day	2619010	down	GO:0006810	0,001159639	transport
Δfst4	8-day	2620649	down	GO:0006810	0,001159639	transport
Δfst4	8-day	2622598	down	GO:0006810	0,001159639	transport
Δfst4	8-day	2623043	down	GO:0006810	0,001159639	transport
Δfst4	8-day	2623492	down	GO:0006810	0,001159639	transport
Δfst4	8-day	2627701	down	GO:0006810	0,001159639	transport

Δfst4	8-day	2627703	down	GO:0006810	0,001159639	transport
Δfst4	8-day	2627883	down	GO:0006810	0,001159639	transport
Δfst4	8-day	2630802	down	GO:0006810	0,001159639	transport
Δfst4	8-day	2631566	down	GO:0006810	0,001159639	transport
Δfst4	8-day	2631822	down	GO:0006810	0,001159639	transport
Δfst4	8-day	2633216	down	GO:0006810	0,001159639	transport
Δfst4	8-day	2636597	down	GO:0006810	0,001159639	transport
Δfst4	8-day	2638711	down	GO:0006810	0,001159639	transport
Δfst4	8-day	2641735	down	GO:0006810	0,001159639	transport
Δfst4	8-day	2642364	down	GO:0006810	0,001159639	transport
Δfst4	8-day	2661224	down	GO:0006810	0,001159639	transport
Δfst4	8-day	2662176	down	GO:0006810	0,001159639	transport
Δfst4	8-day	2662585	down	GO:0006810	0,001159639	transport
Δfst4	8-day	2701571	down	GO:0006810	0,001159639	transport
Δfst4	8-day	2738153	down	GO:0006810	0,001159639	transport
Δfst4	8-day	2747523	down	GO:0006810	0,001159639	transport
Δfst4	8-day	2749926	down	GO:0006810	0,001159639	transport
Δfst4	8-day	1129508	down	GO:0005215	0,002197779	transporter activity
Δfst4	8-day	1144263	down	GO:0005215	0,002197779	transporter activity
Δfst4	8-day	1147635	down	GO:0005215	0,002197779	transporter activity
Δfst4	8-day	1154281	down	GO:0005215	0,002197779	transporter activity
Δfst4	8-day	1175357	down	GO:0005215	0,002197779	transporter activity
Δfst4	8-day	1245995	down	GO:0005215	0,002197779	transporter activity
Δfst4	8-day	1357952	down	GO:0005215	0,002197779	transporter activity
Δfst4	8-day	2138287	down	GO:0005215	0,002197779	transporter activity
Δfst4	8-day	2162840	down	GO:0005215	0,002197779	transporter activity
Δfst4	8-day	2481653	down	GO:0005215	0,002197779	transporter activity
Δfst4	8-day	2491665	down	GO:0005215	0,002197779	transporter activity
Δfst4	8-day	2492878	down	GO:0005215	0,002197779	transporter activity
Δfst4	8-day	2501978	down	GO:0005215	0,002197779	transporter activity
Δfst4	8-day	2504863	down	GO:0005215	0,002197779	transporter activity
Δfst4	8-day	2509716	down	GO:0005215	0,002197779	transporter activity
Δfst4	8-day	2543225	down	GO:0005215	0,002197779	transporter activity
Δfst4	8-day	2546372	down	GO:0005215	0,002197779	transporter activity

Δfst4	8-day	2550918	down	GO:0005215	0,002197779	transporter activity
Δfst4	8-day	2564524	down	GO:0005215	0,002197779	transporter activity
Δfst4	8-day	2600551	down	GO:0005215	0,002197779	transporter activity
Δfst4	8-day	2612313	down	GO:0005215	0,002197779	transporter activity
Δfst4	8-day	2616189	down	GO:0005215	0,002197779	transporter activity
Δfst4	8-day	2617636	down	GO:0005215	0,002197779	transporter activity
Δfst4	8-day	2618539	down	GO:0005215	0,002197779	transporter activity
Δfst4	8-day	2619010	down	GO:0005215	0,002197779	transporter activity
Δfst4	8-day	2622598	down	GO:0005215	0,002197779	transporter activity
Δfst4	8-day	2623043	down	GO:0005215	0,002197779	transporter activity
Δfst4	8-day	2623492	down	GO:0005215	0,002197779	transporter activity
Δfst4	8-day	2627703	down	GO:0005215	0,002197779	transporter activity
Δfst4	8-day	2627883	down	GO:0005215	0,002197779	transporter activity
Δfst4	8-day	2630802	down	GO:0005215	0,002197779	transporter activity
Δfst4	8-day	2633216	down	GO:0005215	0,002197779	transporter activity
Δfst4	8-day	2636597	down	GO:0005215	0,002197779	transporter activity
Δfst4	8-day	2641735	down	GO:0005215	0,002197779	transporter activity
Δfst4	8-day	2642553	down	GO:0005215	0,002197779	transporter activity
Δfst4	8-day	2661224	down	GO:0005215	0,002197779	transporter activity
Δfst4	8-day	2662176	down	GO:0005215	0,002197779	transporter activity
Δfst4	8-day	2701571	down	GO:0005215	0,002197779	transporter activity
Δfst4	8-day	2738153	down	GO:0005215	0,002197779	transporter activity
Δfst4	8-day	2747523	down	GO:0005215	0,002197779	transporter activity
Δfst4	8-day	2493870	down	GO:0004003	0,002425106	ATP-dependent DNA helicase activity
Δfst4	8-day	2625496	down	GO:0004003	0,002425106	ATP-dependent DNA helicase activity
Δfst4	8-day	2677580	down	GO:0004003	0,002425106	ATP-dependent DNA helicase activity
Δfst4	8-day	2512973	down	GO:0034061	0,002738888	DNA polymerase activity
Δfst4	8-day	2629497	down	GO:0005694	0,002738888	chromosome
Δfst4	8-day	2493870	down	GO:0042624	0,002738888	ATPase activity, uncoupled
Δfst4	8-day	2677580	down	GO:0042624	0,002738888	ATPase activity, uncoupled
Δfst4	8-day	2493870	down	GO:0042625	0,002738888	ATPase activity, coupled to transmembrane movement of ions
Δfst4	8-day	2677580	down	GO:0042625	0,002738888	ATPase activity, coupled to transmembrane movement of ions
Δfst4	8-day	2493870	down	GO:0042626	0,002738888	ATPase activity, coupled to transmembrane movement of substances

Δfst4	8-day	2565471	down	GO:0042626	0,002738888	ATPase activity, coupled to transmembrane movement of substances
Δfst4	8-day	2677580	down	GO:0042626	0,002738888	ATPase activity, coupled to transmembrane movement of substances
Δfst4	8-day	2493870	down	GO:0042623	0,002738888	ATPase activity, coupled
Δfst4	8-day	2677580	down	GO:0042623	0,002738888	ATPase activity, coupled
Δfst4	8-day	2493870	down	GO:0015462	0,002738888	protein-transmembrane transporting ATPase activity
Δfst4	8-day	2677580	down	GO:0015462	0,002738888	protein-transmembrane transporting ATPase activity
Δfst4	8-day	2493870	down	GO:0017116	0,002738888	single-stranded DNA-dependent ATP-dependent DNA helicase activity
Δfst4	8-day	2677580	down	GO:0017116	0,002738888	single-stranded DNA-dependent ATP-dependent DNA helicase activity
Δfst4	8-day	1192535	down	GO:0004339	0,002738888	glucan 1,4-alpha-glucosidase activity
Δfst4	8-day	2631634	down	GO:0004339	0,002738888	glucan 1,4-alpha-glucosidase activity
Δfst4	8-day	78628	down	GO:0006118	0,002738888	electron transport
Δfst4	8-day	257768	down	GO:0006118	0,002738888	electron transport
Δfst4	8-day	1084124	down	GO:0006118	0,002738888	electron transport
Δfst4	8-day	1131542	down	GO:0006118	0,002738888	electron transport
Δfst4	8-day	1159597	down	GO:0006118	0,002738888	electron transport
Δfst4	8-day	1189794	down	GO:0006118	0,002738888	electron transport
Δfst4	8-day	2247626	down	GO:0006118	0,002738888	electron transport
Δfst4	8-day	2486680	down	GO:0006118	0,002738888	electron transport
Δfst4	8-day	2503108	down	GO:0006118	0,002738888	electron transport
Δfst4	8-day	2509716	down	GO:0006118	0,002738888	electron transport
Δfst4	8-day	2515850	down	GO:0006118	0,002738888	electron transport
Δfst4	8-day	2516208	down	GO:0006118	0,002738888	electron transport
Δfst4	8-day	2532967	down	GO:0006118	0,002738888	electron transport
Δfst4	8-day	2533120	down	GO:0006118	0,002738888	electron transport
Δfst4	8-day	2534187	down	GO:0006118	0,002738888	electron transport
Δfst4	8-day	2559987	down	GO:0006118	0,002738888	electron transport
Δfst4	8-day	2594763	down	GO:0006118	0,002738888	electron transport
Δfst4	8-day	2603449	down	GO:0006118	0,002738888	electron transport
Δfst4	8-day	2604735	down	GO:0006118	0,002738888	electron transport
Δfst4	8-day	2611771	down	GO:0006118	0,002738888	electron transport
Δfst4	8-day	2614692	down	GO:0006118	0,002738888	electron transport

Δfst4	8-day	2614998	down	GO:0006118	0,002738888	electron transport
Δfst4	8-day	2615184	down	GO:0006118	0,002738888	electron transport
Δfst4	8-day	2616590	down	GO:0006118	0,002738888	electron transport
Δfst4	8-day	2619674	down	GO:0006118	0,002738888	electron transport
Δfst4	8-day	2619910	down	GO:0006118	0,002738888	electron transport
Δfst4	8-day	2621855	down	GO:0006118	0,002738888	electron transport
Δfst4	8-day	2622733	down	GO:0006118	0,002738888	electron transport
Δfst4	8-day	2625951	down	GO:0006118	0,002738888	electron transport
Δfst4	8-day	2628312	down	GO:0006118	0,002738888	electron transport
Δfst4	8-day	2628651	down	GO:0006118	0,002738888	electron transport
Δfst4	8-day	2633167	down	GO:0006118	0,002738888	electron transport
Δfst4	8-day	2634186	down	GO:0006118	0,002738888	electron transport
Δfst4	8-day	2635487	down	GO:0006118	0,002738888	electron transport
Δfst4	8-day	2637104	down	GO:0006118	0,002738888	electron transport
Δfst4	8-day	2638355	down	GO:0006118	0,002738888	electron transport
Δfst4	8-day	2662585	down	GO:0006118	0,002738888	electron transport
Δfst4	8-day	2671785	down	GO:0006118	0,002738888	electron transport
Δfst4	8-day	2703965	down	GO:0006118	0,002738888	electron transport
Δfst4	8-day	2703966	down	GO:0006118	0,002738888	electron transport
Δfst4	8-day	2705258	down	GO:0006118	0,002738888	electron transport
Δfst4	8-day	2493870	down	GO:0004004	0,002738888	ATP-dependent RNA helicase activity
Δfst4	8-day	2677580	down	GO:0004004	0,002738888	ATP-dependent RNA helicase activity
Δfst4	8-day	2493870	down	GO:0008186	0,002738888	RNA-dependent ATPase activity
Δfst4	8-day	2677580	down	GO:0008186	0,002738888	RNA-dependent ATPase activity
Δfst4	8-day	2512973	down	GO:0003887	0,002875782	DNA-directed DNA polymerase activity
Δfst4	8-day	2630033	down	GO:0016717	0,002875782	oxidoreductase activity, acting on paired donors
Δfst4	8-day	2493870	down	GO:0008094	0,003085382	DNA-dependent ATPase activity
Δfst4	8-day	2677580	down	GO:0008094	0,003085382	DNA-dependent ATPase activity
Δfst4	8-day	2369054	down	GO:0005199	0,00432484	structural constituent of cell wall
Δfst4	8-day	2746250	down	GO:0005199	0,00432484	structural constituent of cell wall
Δfst4	8-day	78628	down	GO:0005506	0,004403822	iron ion binding
Δfst4	8-day	1131542	down	GO:0005506	0,004403822	iron ion binding
Δfst4	8-day	1159597	down	GO:0005506	0,004403822	iron ion binding
Δfst4	8-day	1189794	down	GO:0005506	0,004403822	iron ion binding

Δfst4	8-day	1191999	down	GO:0005506	0,004403822	iron ion binding
Δfst4	8-day	2486680	down	GO:0005506	0,004403822	iron ion binding
Δfst4	8-day	2503108	down	GO:0005506	0,004403822	iron ion binding
Δfst4	8-day	2516208	down	GO:0005506	0,004403822	iron ion binding
Δfst4	8-day	2559987	down	GO:0005506	0,004403822	iron ion binding
Δfst4	8-day	2594763	down	GO:0005506	0,004403822	iron ion binding
Δfst4	8-day	2603449	down	GO:0005506	0,004403822	iron ion binding
Δfst4	8-day	2604735	down	GO:0005506	0,004403822	iron ion binding
Δfst4	8-day	2611771	down	GO:0005506	0,004403822	iron ion binding
Δfst4	8-day	2614692	down	GO:0005506	0,004403822	iron ion binding
Δfst4	8-day	2616590	down	GO:0005506	0,004403822	iron ion binding
Δfst4	8-day	2619674	down	GO:0005506	0,004403822	iron ion binding
Δfst4	8-day	2621855	down	GO:0005506	0,004403822	iron ion binding
Δfst4	8-day	2628312	down	GO:0005506	0,004403822	iron ion binding
Δfst4	8-day	2628651	down	GO:0005506	0,004403822	iron ion binding
Δfst4	8-day	2634186	down	GO:0005506	0,004403822	iron ion binding
Δfst4	8-day	2635487	down	GO:0005506	0,004403822	iron ion binding
Δfst4	8-day	2636420	down	GO:0005506	0,004403822	iron ion binding
Δfst4	8-day	2638355	down	GO:0005506	0,004403822	iron ion binding
Δfst4	8-day	2641761	down	GO:0005506	0,004403822	iron ion binding
Δfst4	8-day	2703965	down	GO:0005506	0,004403822	iron ion binding
Δfst4	8-day	2703966	down	GO:0005506	0,004403822	iron ion binding
Δfst4	8-day	2705258	down	GO:0005506	0,004403822	iron ion binding
Δfst4	8-day	1077503	down	GO:0016020	0,005288077	membrane
Δfst4	8-day	1093363	down	GO:0016020	0,005288077	membrane
Δfst4	8-day	1144263	down	GO:0016020	0,005288077	membrane
Δfst4	8-day	1147635	down	GO:0016020	0,005288077	membrane
Δfst4	8-day	1160309	down	GO:0016020	0,005288077	membrane
Δfst4	8-day	1175357	down	GO:0016020	0,005288077	membrane
Δfst4	8-day	1245995	down	GO:0016020	0,005288077	membrane
Δfst4	8-day	2138287	down	GO:0016020	0,005288077	membrane
Δfst4	8-day	2162840	down	GO:0016020	0,005288077	membrane
Δfst4	8-day	2493264	down	GO:0016020	0,005288077	membrane
Δfst4	8-day	2514012	down	GO:0016020	0,005288077	membrane

Δfst4	8-day	2550918	down	GO:0016020	0,005288077	membrane
Δfst4	8-day	2565471	down	GO:0016020	0,005288077	membrane
Δfst4	8-day	2600551	down	GO:0016020	0,005288077	membrane
Δfst4	8-day	2604751	down	GO:0016020	0,005288077	membrane
Δfst4	8-day	2609987	down	GO:0016020	0,005288077	membrane
Δfst4	8-day	2610450	down	GO:0016020	0,005288077	membrane
Δfst4	8-day	2616189	down	GO:0016020	0,005288077	membrane
Δfst4	8-day	2616602	down	GO:0016020	0,005288077	membrane
Δfst4	8-day	2618501	down	GO:0016020	0,005288077	membrane
Δfst4	8-day	2619010	down	GO:0016020	0,005288077	membrane
Δfst4	8-day	2620649	down	GO:0016020	0,005288077	membrane
Δfst4	8-day	2621162	down	GO:0016020	0,005288077	membrane
Δfst4	8-day	2622595	down	GO:0016020	0,005288077	membrane
Δfst4	8-day	2622598	down	GO:0016020	0,005288077	membrane
Δfst4	8-day	2623043	down	GO:0016020	0,005288077	membrane
Δfst4	8-day	2627701	down	GO:0016020	0,005288077	membrane
Δfst4	8-day	2627703	down	GO:0016020	0,005288077	membrane
Δfst4	8-day	2627883	down	GO:0016020	0,005288077	membrane
Δfst4	8-day	2627998	down	GO:0016020	0,005288077	membrane
Δfst4	8-day	2630033	down	GO:0016020	0,005288077	membrane
Δfst4	8-day	2630802	down	GO:0016020	0,005288077	membrane
Δfst4	8-day	2631566	down	GO:0016020	0,005288077	membrane
Δfst4	8-day	2631822	down	GO:0016020	0,005288077	membrane
Δfst4	8-day	2638711	down	GO:0016020	0,005288077	membrane
Δfst4	8-day	2640999	down	GO:0016020	0,005288077	membrane
Δfst4	8-day	2642364	down	GO:0016020	0,005288077	membrane
Δfst4	8-day	2642553	down	GO:0016020	0,005288077	membrane
Δfst4	8-day	2662176	down	GO:0016020	0,005288077	membrane
Δfst4	8-day	2668580	down	GO:0016020	0,005288077	membrane
Δfst4	8-day	2673672	down	GO:0016020	0,005288077	membrane
Δfst4	8-day	2675646	down	GO:0016020	0,005288077	membrane
Δfst4	8-day	2696647	down	GO:0016020	0,005288077	membrane
Δfst4	8-day	2701571	down	GO:0016020	0,005288077	membrane
Δfst4	8-day	2738153	down	GO:0016020	0,005288077	membrane

Δfst4	8-day	2749926	down	GO:0016020	0,005288077	membrane
Δfst4	8-day	2369054	down	GO:0005618	0,006188309	cell wall
Δfst4	8-day	1176082	down	GO:0005509	0,006188309	calcium ion binding
Δfst4	8-day	2520635	down	GO:0005509	0,006188309	calcium ion binding
Δfst4	8-day	2673672	down	GO:0005509	0,006188309	calcium ion binding
Δfst4	8-day	1131965	down	GO:0005488	0,006215447	binding
Δfst4	8-day	1152293	down	GO:0005488	0,006215447	binding
Δfst4	8-day	1193333	down	GO:0005488	0,006215447	binding
Δfst4	8-day	1357952	down	GO:0005488	0,006215447	binding
Δfst4	8-day	2072306	down	GO:0005488	0,006215447	binding
Δfst4	8-day	2110046	down	GO:0005488	0,006215447	binding
Δfst4	8-day	2135332	down	GO:0005488	0,006215447	binding
Δfst4	8-day	2333133	down	GO:0005488	0,006215447	binding
Δfst4	8-day	2373661	down	GO:0005488	0,006215447	binding
Δfst4	8-day	2492878	down	GO:0005488	0,006215447	binding
Δfst4	8-day	2495855	down	GO:0005488	0,006215447	binding
Δfst4	8-day	2510775	down	GO:0005488	0,006215447	binding
Δfst4	8-day	2511020	down	GO:0005488	0,006215447	binding
Δfst4	8-day	2546372	down	GO:0005488	0,006215447	binding
Δfst4	8-day	2572586	down	GO:0005488	0,006215447	binding
Δfst4	8-day	2575853	down	GO:0005488	0,006215447	binding
Δfst4	8-day	2587289	down	GO:0005488	0,006215447	binding
Δfst4	8-day	2591516	down	GO:0005488	0,006215447	binding
Δfst4	8-day	2595361	down	GO:0005488	0,006215447	binding
Δfst4	8-day	2604699	down	GO:0005488	0,006215447	binding
Δfst4	8-day	2609281	down	GO:0005488	0,006215447	binding
Δfst4	8-day	2610587	down	GO:0005488	0,006215447	binding
Δfst4	8-day	2610599	down	GO:0005488	0,006215447	binding
Δfst4	8-day	2612591	down	GO:0005488	0,006215447	binding
Δfst4	8-day	2619248	down	GO:0005488	0,006215447	binding
Δfst4	8-day	2620649	down	GO:0005488	0,006215447	binding
Δfst4	8-day	2625782	down	GO:0005488	0,006215447	binding
Δfst4	8-day	2627113	down	GO:0005488	0,006215447	binding
Δfst4	8-day	2627883	down	GO:0005488	0,006215447	binding

Δfst4	8-day	2629526	down	GO:0005488	0,006215447	binding
Δfst4	8-day	2630590	down	GO:0005488	0,006215447	binding
Δfst4	8-day	2630802	down	GO:0005488	0,006215447	binding
Δfst4	8-day	2631368	down	GO:0005488	0,006215447	binding
Δfst4	8-day	2631822	down	GO:0005488	0,006215447	binding
Δfst4	8-day	2632611	down	GO:0005488	0,006215447	binding
Δfst4	8-day	2633167	down	GO:0005488	0,006215447	binding
Δfst4	8-day	2635567	down	GO:0005488	0,006215447	binding
Δfst4	8-day	2637104	down	GO:0005488	0,006215447	binding
Δfst4	8-day	2637668	down	GO:0005488	0,006215447	binding
Δfst4	8-day	2641506	down	GO:0005488	0,006215447	binding
Δfst4	8-day	2641520	down	GO:0005488	0,006215447	binding
Δfst4	8-day	2661224	down	GO:0005488	0,006215447	binding
Δfst4	8-day	2681358	down	GO:0005488	0,006215447	binding
Δfst4	8-day	2693285	down	GO:0005488	0,006215447	binding
Δfst4	8-day	2749926	down	GO:0005488	0,006215447	binding
Δfst4	8-day	2614858	down	GO:0046914	0,006649913	transition metal ion binding
Δfst4	8-day	78628	down	GO:0004497	0,020642556	monooxygenase activity
Δfst4	8-day	1131542	down	GO:0004497	0,020642556	monooxygenase activity
Δfst4	8-day	1159597	down	GO:0004497	0,020642556	monooxygenase activity
Δfst4	8-day	1189794	down	GO:0004497	0,020642556	monooxygenase activity
Δfst4	8-day	2486680	down	GO:0004497	0,020642556	monooxygenase activity
Δfst4	8-day	2515850	down	GO:0004497	0,020642556	monooxygenase activity
Δfst4	8-day	2516208	down	GO:0004497	0,020642556	monooxygenase activity
Δfst4	8-day	2534187	down	GO:0004497	0,020642556	monooxygenase activity
Δfst4	8-day	2559987	down	GO:0004497	0,020642556	monooxygenase activity
Δfst4	8-day	2594763	down	GO:0004497	0,020642556	monooxygenase activity
Δfst4	8-day	2604735	down	GO:0004497	0,020642556	monooxygenase activity
Δfst4	8-day	2614692	down	GO:0004497	0,020642556	monooxygenase activity
Δfst4	8-day	2616590	down	GO:0004497	0,020642556	monooxygenase activity
Δfst4	8-day	2619674	down	GO:0004497	0,020642556	monooxygenase activity
Δfst4	8-day	2628312	down	GO:0004497	0,020642556	monooxygenase activity
Δfst4	8-day	2628651	down	GO:0004497	0,020642556	monooxygenase activity
Δfst4	8-day	2634186	down	GO:0004497	0,020642556	monooxygenase activity

Δfst4	8-day	2635487	down	GO:0004497	0,020642556	monooxygenase activity
Δfst4	8-day	2638355	down	GO:0004497	0,020642556	monooxygenase activity
Δfst4	8-day	2671785	down	GO:0004497	0,020642556	monooxygenase activity
Δfst4	8-day	2703965	down	GO:0004497	0,020642556	monooxygenase activity
Δfst4	8-day	2703966	down	GO:0004497	0,020642556	monooxygenase activity
Δfst4	8-day	2705258	down	GO:0004497	0,020642556	monooxygenase activity
Δfst4	8-day	2493870	down	GO:0008026	0,024148563	ATP-dependent helicase activity
Δfst4	8-day	2677580	down	GO:0008026	0,024148563	ATP-dependent helicase activity
Δfst4	8-day	2512030	down	GO:0009058	0,024744166	biosynthetic process
Δfst4	8-day	2512873	down	GO:0009058	0,024744166	biosynthetic process
Δfst4	8-day	2623956	down	GO:0009058	0,024744166	biosynthetic process
Δfst4	8-day	2632714	down	GO:0009058	0,024744166	biosynthetic process
Δfst4	8-day	2616602	down	GO:0005388	0,033524109	calcium-transporting ATPase activity
Δfst4	8-day	2534187	down	GO:0006520	0,033524109	amino acid metabolic process
Δgat1	8-day	2629603	up	GO:0005199	6,29E-09	structural constituent of cell wall
Δgat1	8-day	2614257	up	GO:0005618	7,44E-09	cell wall
Δgat1	8-day	2629603	up	GO:0005618	7,44E-09	cell wall
Δgat1	8-day	78628	up	GO:0004497	9,04E-05	monooxygenase activity
Δgat1	8-day	81631	up	GO:0004497	9,04E-05	monooxygenase activity
Δgat1	8-day	1139445	up	GO:0004497	9,04E-05	monooxygenase activity
Δgat1	8-day	1189794	up	GO:0004497	9,04E-05	monooxygenase activity
Δgat1	8-day	2510618	up	GO:0004497	9,04E-05	monooxygenase activity
Δgat1	8-day	2516208	up	GO:0004497	9,04E-05	monooxygenase activity
Δgat1	8-day	2604096	up	GO:0004497	9,04E-05	monooxygenase activity
Δgat1	8-day	2604458	up	GO:0004497	9,04E-05	monooxygenase activity
Δgat1	8-day	2604735	up	GO:0004497	9,04E-05	monooxygenase activity
Δgat1	8-day	2611234	up	GO:0004497	9,04E-05	monooxygenase activity
Δgat1	8-day	2616301	up	GO:0004497	9,04E-05	monooxygenase activity
Δgat1	8-day	2619840	up	GO:0004497	9,04E-05	monooxygenase activity
Δgat1	8-day	2623115	up	GO:0004497	9,04E-05	monooxygenase activity
Δgat1	8-day	2623122	up	GO:0004497	9,04E-05	monooxygenase activity
Δgat1	8-day	2628651	up	GO:0004497	9,04E-05	monooxygenase activity
Δgat1	8-day	2635487	up	GO:0004497	9,04E-05	monooxygenase activity
Δgat1	8-day	2636544	up	GO:0004497	9,04E-05	monooxygenase activity

Δgat1	8-day	2644430	up	GO:0004497	9,04E-05	monooxygenase activity
Δgat1	8-day	2703628	up	GO:0004497	9,04E-05	monooxygenase activity
Δgat1	8-day	2703966	up	GO:0004497	9,04E-05	monooxygenase activity
Δgat1	8-day	2735179	up	GO:0004497	9,04E-05	monooxygenase activity
Δgat1	8-day	1131965	up	GO:0008152	0,000350754	metabolic process
Δgat1	8-day	1156481	up	GO:0008152	0,000350754	metabolic process
Δgat1	8-day	1193333	up	GO:0008152	0,000350754	metabolic process
Δgat1	8-day	2135332	up	GO:0008152	0,000350754	metabolic process
Δgat1	8-day	2255031	up	GO:0008152	0,000350754	metabolic process
Δgat1	8-day	2333133	up	GO:0008152	0,000350754	metabolic process
Δgat1	8-day	2373661	up	GO:0008152	0,000350754	metabolic process
Δgat1	8-day	2577440	up	GO:0008152	0,000350754	metabolic process
Δgat1	8-day	2587289	up	GO:0008152	0,000350754	metabolic process
Δgat1	8-day	2609150	up	GO:0008152	0,000350754	metabolic process
Δgat1	8-day	2610599	up	GO:0008152	0,000350754	metabolic process
Δgat1	8-day	2612018	up	GO:0008152	0,000350754	metabolic process
Δgat1	8-day	2615598	up	GO:0008152	0,000350754	metabolic process
Δgat1	8-day	2616916	up	GO:0008152	0,000350754	metabolic process
Δgat1	8-day	2619840	up	GO:0008152	0,000350754	metabolic process
Δgat1	8-day	2621873	up	GO:0008152	0,000350754	metabolic process
Δgat1	8-day	2624073	up	GO:0008152	0,000350754	metabolic process
Δgat1	8-day	2624525	up	GO:0008152	0,000350754	metabolic process
Δgat1	8-day	2625782	up	GO:0008152	0,000350754	metabolic process
Δgat1	8-day	2626510	up	GO:0008152	0,000350754	metabolic process
Δgat1	8-day	2627257	up	GO:0008152	0,000350754	metabolic process
Δgat1	8-day	2629548	up	GO:0008152	0,000350754	metabolic process
Δgat1	8-day	2630599	up	GO:0008152	0,000350754	metabolic process
Δgat1	8-day	2632206	up	GO:0008152	0,000350754	metabolic process
Δgat1	8-day	2632611	up	GO:0008152	0,000350754	metabolic process
Δgat1	8-day	2633266	up	GO:0008152	0,000350754	metabolic process
Δgat1	8-day	2633632	up	GO:0008152	0,000350754	metabolic process
Δgat1	8-day	2634450	up	GO:0008152	0,000350754	metabolic process
Δgat1	8-day	2634903	up	GO:0008152	0,000350754	metabolic process
Δgat1	8-day	2637104	up	GO:0008152	0,000350754	metabolic process

Δgat1	8-day	2637643	up	GO:0008152	0,000350754	metabolic process
Δgat1	8-day	2637755	up	GO:0008152	0,000350754	metabolic process
Δgat1	8-day	2638484	up	GO:0008152	0,000350754	metabolic process
Δgat1	8-day	2640472	up	GO:0008152	0,000350754	metabolic process
Δgat1	8-day	2640484	up	GO:0008152	0,000350754	metabolic process
Δgat1	8-day	2641506	up	GO:0008152	0,000350754	metabolic process
Δgat1	8-day	2641520	up	GO:0008152	0,000350754	metabolic process
Δgat1	8-day	2644127	up	GO:0008152	0,000350754	metabolic process
Δgat1	8-day	2644330	up	GO:0008152	0,000350754	metabolic process
Δgat1	8-day	2644430	up	GO:0008152	0,000350754	metabolic process
Δgat1	8-day	2705421	up	GO:0008152	0,000350754	metabolic process
Δgat1	8-day	257768	up	GO:0016491	0,000363678	oxidoreductase activity
Δgat1	8-day	1131965	up	GO:0016491	0,000363678	oxidoreductase activity
Δgat1	8-day	1156481	up	GO:0016491	0,000363678	oxidoreductase activity
Δgat1	8-day	2255031	up	GO:0016491	0,000363678	oxidoreductase activity
Δgat1	8-day	2312801	up	GO:0016491	0,000363678	oxidoreductase activity
Δgat1	8-day	2333133	up	GO:0016491	0,000363678	oxidoreductase activity
Δgat1	8-day	2376858	up	GO:0016491	0,000363678	oxidoreductase activity
Δgat1	8-day	2498556	up	GO:0016491	0,000363678	oxidoreductase activity
Δgat1	8-day	2503108	up	GO:0016491	0,000363678	oxidoreductase activity
Δgat1	8-day	2587289	up	GO:0016491	0,000363678	oxidoreductase activity
Δgat1	8-day	2604174	up	GO:0016491	0,000363678	oxidoreductase activity
Δgat1	8-day	2609840	up	GO:0016491	0,000363678	oxidoreductase activity
Δgat1	8-day	2610599	up	GO:0016491	0,000363678	oxidoreductase activity
Δgat1	8-day	2616916	up	GO:0016491	0,000363678	oxidoreductase activity
Δgat1	8-day	2617060	up	GO:0016491	0,000363678	oxidoreductase activity
Δgat1	8-day	2619840	up	GO:0016491	0,000363678	oxidoreductase activity
Δgat1	8-day	2621873	up	GO:0016491	0,000363678	oxidoreductase activity
Δgat1	8-day	2624073	up	GO:0016491	0,000363678	oxidoreductase activity
Δgat1	8-day	2624407	up	GO:0016491	0,000363678	oxidoreductase activity
Δgat1	8-day	2630599	up	GO:0016491	0,000363678	oxidoreductase activity
Δgat1	8-day	2633266	up	GO:0016491	0,000363678	oxidoreductase activity
Δgat1	8-day	2633632	up	GO:0016491	0,000363678	oxidoreductase activity
Δgat1	8-day	2634450	up	GO:0016491	0,000363678	oxidoreductase activity

Δgat1	8-day	2634903	up	GO:0016491	0,000363678	oxidoreductase activity
Δgat1	8-day	2637104	up	GO:0016491	0,000363678	oxidoreductase activity
Δgat1	8-day	2637755	up	GO:0016491	0,000363678	oxidoreductase activity
Δgat1	8-day	2638484	up	GO:0016491	0,000363678	oxidoreductase activity
Δgat1	8-day	2639682	up	GO:0016491	0,000363678	oxidoreductase activity
Δgat1	8-day	2641506	up	GO:0016491	0,000363678	oxidoreductase activity
Δgat1	8-day	2641520	up	GO:0016491	0,000363678	oxidoreductase activity
Δgat1	8-day	2644127	up	GO:0016491	0,000363678	oxidoreductase activity
Δgat1	8-day	2644330	up	GO:0016491	0,000363678	oxidoreductase activity
Δgat1	8-day	2644430	up	GO:0016491	0,000363678	oxidoreductase activity
Δgat1	8-day	2688172	up	GO:0016491	0,000363678	oxidoreductase activity
Δgat1	8-day	2705421	up	GO:0016491	0,000363678	oxidoreductase activity
Δgat1	8-day	78628	up	GO:0020037	0,000562173	heme binding
Δgat1	8-day	81631	up	GO:0020037	0,000562173	heme binding
Δgat1	8-day	1139445	up	GO:0020037	0,000562173	heme binding
Δgat1	8-day	1189794	up	GO:0020037	0,000562173	heme binding
Δgat1	8-day	2376858	up	GO:0020037	0,000562173	heme binding
Δgat1	8-day	2510618	up	GO:0020037	0,000562173	heme binding
Δgat1	8-day	2516208	up	GO:0020037	0,000562173	heme binding
Δgat1	8-day	2604096	up	GO:0020037	0,000562173	heme binding
Δgat1	8-day	2604458	up	GO:0020037	0,000562173	heme binding
Δgat1	8-day	2604628	up	GO:0020037	0,000562173	heme binding
Δgat1	8-day	2604735	up	GO:0020037	0,000562173	heme binding
Δgat1	8-day	2609840	up	GO:0020037	0,000562173	heme binding
Δgat1	8-day	2611234	up	GO:0020037	0,000562173	heme binding
Δgat1	8-day	2614998	up	GO:0020037	0,000562173	heme binding
Δgat1	8-day	2616301	up	GO:0020037	0,000562173	heme binding
Δgat1	8-day	2621855	up	GO:0020037	0,000562173	heme binding
Δgat1	8-day	2623115	up	GO:0020037	0,000562173	heme binding
Δgat1	8-day	2623122	up	GO:0020037	0,000562173	heme binding
Δgat1	8-day	2628651	up	GO:0020037	0,000562173	heme binding
Δgat1	8-day	2635487	up	GO:0020037	0,000562173	heme binding
Δgat1	8-day	2636544	up	GO:0020037	0,000562173	heme binding
Δgat1	8-day	2703628	up	GO:0020037	0,000562173	heme binding

Δgat1	8-day	2703966	up	GO:0020037	0,000562173	heme binding
Δgat1	8-day	2735179	up	GO:0020037	0,000562173	heme binding
Δgat1	8-day	257768	up	GO:0050660	0,00157944	FAD binding
Δgat1	8-day	2503108	up	GO:0050660	0,00157944	FAD binding
Δgat1	8-day	2607677	up	GO:0050660	0,00157944	FAD binding
Δgat1	8-day	2610771	up	GO:0050660	0,00157944	FAD binding
Δgat1	8-day	2642607	up	GO:0050660	0,00157944	FAD binding
Δgat1	8-day	2688172	up	GO:0050660	0,00157944	FAD binding
Δgat1	8-day	2607677	up	GO:0016614	0,004880619	oxidoreductase activity, acting on CH-OH group of donors
Δgat1	8-day	2610771	up	GO:0016614	0,004880619	oxidoreductase activity, acting on CH-OH group of donors
Δgat1	8-day	2642607	up	GO:0016614	0,004880619	oxidoreductase activity, acting on CH-OH group of donors
Δgat1	8-day	78628	up	GO:0006118	0,005273466	electron transport
Δgat1	8-day	81631	up	GO:0006118	0,005273466	electron transport
Δgat1	8-day	257768	up	GO:0006118	0,005273466	electron transport
Δgat1	8-day	1139445	up	GO:0006118	0,005273466	electron transport
Δgat1	8-day	1189794	up	GO:0006118	0,005273466	electron transport
Δgat1	8-day	2498556	up	GO:0006118	0,005273466	electron transport
Δgat1	8-day	2503108	up	GO:0006118	0,005273466	electron transport
Δgat1	8-day	2510618	up	GO:0006118	0,005273466	electron transport
Δgat1	8-day	2516208	up	GO:0006118	0,005273466	electron transport
Δgat1	8-day	2604096	up	GO:0006118	0,005273466	electron transport
Δgat1	8-day	2604458	up	GO:0006118	0,005273466	electron transport
Δgat1	8-day	2604628	up	GO:0006118	0,005273466	electron transport
Δgat1	8-day	2604735	up	GO:0006118	0,005273466	electron transport
Δgat1	8-day	2609831	up	GO:0006118	0,005273466	electron transport
Δgat1	8-day	2609840	up	GO:0006118	0,005273466	electron transport
Δgat1	8-day	2611234	up	GO:0006118	0,005273466	electron transport
Δgat1	8-day	2614998	up	GO:0006118	0,005273466	electron transport
Δgat1	8-day	2616301	up	GO:0006118	0,005273466	electron transport
Δgat1	8-day	2619840	up	GO:0006118	0,005273466	electron transport
Δgat1	8-day	2620726	up	GO:0006118	0,005273466	electron transport
Δgat1	8-day	2621855	up	GO:0006118	0,005273466	electron transport
Δgat1	8-day	2623115	up	GO:0006118	0,005273466	electron transport
Δgat1	8-day	2623122	up	GO:0006118	0,005273466	electron transport

Δgat1	8-day	2628651	up	GO:0006118	0,005273466	electron transport
Δgat1	8-day	2635487	up	GO:0006118	0,005273466	electron transport
Δgat1	8-day	2636544	up	GO:0006118	0,005273466	electron transport
Δgat1	8-day	2637104	up	GO:0006118	0,005273466	electron transport
Δgat1	8-day	2639682	up	GO:0006118	0,005273466	electron transport
Δgat1	8-day	2644430	up	GO:0006118	0,005273466	electron transport
Δgat1	8-day	2662585	up	GO:0006118	0,005273466	electron transport
Δgat1	8-day	2703628	up	GO:0006118	0,005273466	electron transport
Δgat1	8-day	2703966	up	GO:0006118	0,005273466	electron transport
Δgat1	8-day	2735179	up	GO:0006118	0,005273466	electron transport
Δgat1	8-day	2609831	up	GO:0005507	0,006102775	copper ion binding
Δgat1	8-day	78628	up	GO:0050381	0,010486262	unspecific monooxygenase activity
Δgat1	8-day	81631	up	GO:0050381	0,010486262	unspecific monooxygenase activity
Δgat1	8-day	1139445	up	GO:0050381	0,010486262	unspecific monooxygenase activity
Δgat1	8-day	2510618	up	GO:0050381	0,010486262	unspecific monooxygenase activity
Δgat1	8-day	2604458	up	GO:0050381	0,010486262	unspecific monooxygenase activity
Δgat1	8-day	2628651	up	GO:0050381	0,010486262	unspecific monooxygenase activity
Δgat1	8-day	2636544	up	GO:0050381	0,010486262	unspecific monooxygenase activity
Δgat1	8-day	2703628	up	GO:0050381	0,010486262	unspecific monooxygenase activity
Δgat1	8-day	2703966	up	GO:0050381	0,010486262	unspecific monooxygenase activity
Δgat1	8-day	2735179	up	GO:0050381	0,010486262	unspecific monooxygenase activity
Δgat1	8-day	78628	up	GO:0005506	0,024343875	iron ion binding
Δgat1	8-day	81631	up	GO:0005506	0,024343875	iron ion binding
Δgat1	8-day	1139445	up	GO:0005506	0,024343875	iron ion binding
Δgat1	8-day	1189794	up	GO:0005506	0,024343875	iron ion binding
Δgat1	8-day	2312801	up	GO:0005506	0,024343875	iron ion binding
Δgat1	8-day	2376858	up	GO:0005506	0,024343875	iron ion binding
Δgat1	8-day	2503108	up	GO:0005506	0,024343875	iron ion binding
Δgat1	8-day	2510618	up	GO:0005506	0,024343875	iron ion binding
Δgat1	8-day	2516208	up	GO:0005506	0,024343875	iron ion binding
Δgat1	8-day	2604096	up	GO:0005506	0,024343875	iron ion binding
Δgat1	8-day	2604458	up	GO:0005506	0,024343875	iron ion binding
Δgat1	8-day	2604628	up	GO:0005506	0,024343875	iron ion binding
Δgat1	8-day	2604735	up	GO:0005506	0,024343875	iron ion binding

Δgat1	8-day	2611234	up	GO:0005506	0,024343875	iron ion binding
Δgat1	8-day	2616301	up	GO:0005506	0,024343875	iron ion binding
Δgat1	8-day	2617060	up	GO:0005506	0,024343875	iron ion binding
Δgat1	8-day	2621855	up	GO:0005506	0,024343875	iron ion binding
Δgat1	8-day	2623115	up	GO:0005506	0,024343875	iron ion binding
Δgat1	8-day	2623122	up	GO:0005506	0,024343875	iron ion binding
Δgat1	8-day	2628651	up	GO:0005506	0,024343875	iron ion binding
Δgat1	8-day	2635487	up	GO:0005506	0,024343875	iron ion binding
Δgat1	8-day	2636544	up	GO:0005506	0,024343875	iron ion binding
Δgat1	8-day	2703628	up	GO:0005506	0,024343875	iron ion binding
Δgat1	8-day	2703966	up	GO:0005506	0,024343875	iron ion binding
Δgat1	8-day	2735179	up	GO:0005506	0,024343875	iron ion binding
Δgat1	8-day	2577440	up	GO:0000162	0,035771354	tryptophan biosynthetic process
Δgat1	8-day	2619840	up	GO:0006725	0,035771354	aromatic compound metabolic process
Δgat1	8-day	2644430	up	GO:0006725	0,035771354	aromatic compound metabolic process
Δgat1	8-day	1087819	down	GO:0005975	8,18E-09	carbohydrate metabolic process
Δgat1	8-day	2514546	down	GO:0005975	8,18E-09	carbohydrate metabolic process
Δgat1	8-day	2613657	down	GO:0005975	8,18E-09	carbohydrate metabolic process
Δgat1	8-day	2626756	down	GO:0005975	8,18E-09	carbohydrate metabolic process
Δgat1	8-day	2636145	down	GO:0005975	8,18E-09	carbohydrate metabolic process
Δgat1	8-day	2641020	down	GO:0005975	8,18E-09	carbohydrate metabolic process
Δgat1	8-day	2670422	down	GO:0005975	8,18E-09	carbohydrate metabolic process
Δgat1	8-day	1083109	down	GO:0005506	1,22E-07	iron ion binding
Δgat1	8-day	1120318	down	GO:0005506	1,22E-07	iron ion binding
Δgat1	8-day	1358353	down	GO:0005506	1,22E-07	iron ion binding
Δgat1	8-day	2364606	down	GO:0005506	1,22E-07	iron ion binding
Δgat1	8-day	2491624	down	GO:0005506	1,22E-07	iron ion binding
Δgat1	8-day	2501258	down	GO:0005506	1,22E-07	iron ion binding
Δgat1	8-day	2514636	down	GO:0005506	1,22E-07	iron ion binding
Δgat1	8-day	2537529	down	GO:0005506	1,22E-07	iron ion binding
Δgat1	8-day	2559987	down	GO:0005506	1,22E-07	iron ion binding
Δgat1	8-day	2619716	down	GO:0005506	1,22E-07	iron ion binding
Δgat1	8-day	2622782	down	GO:0005506	1,22E-07	iron ion binding
Δgat1	8-day	2624973	down	GO:0005506	1,22E-07	iron ion binding

Δgat1	8-day	2635870	down	GO:0005506	1,22E-07	iron ion binding
Δgat1	8-day	2637467	down	GO:0005506	1,22E-07	iron ion binding
Δgat1	8-day	1083109	down	GO:0020037	1,67E-06	heme binding
Δgat1	8-day	1120318	down	GO:0020037	1,67E-06	heme binding
Δgat1	8-day	1358353	down	GO:0020037	1,67E-06	heme binding
Δgat1	8-day	2364606	down	GO:0020037	1,67E-06	heme binding
Δgat1	8-day	2491624	down	GO:0020037	1,67E-06	heme binding
Δgat1	8-day	2501258	down	GO:0020037	1,67E-06	heme binding
Δgat1	8-day	2514636	down	GO:0020037	1,67E-06	heme binding
Δgat1	8-day	2537529	down	GO:0020037	1,67E-06	heme binding
Δgat1	8-day	2559987	down	GO:0020037	1,67E-06	heme binding
Δgat1	8-day	2608091	down	GO:0020037	1,67E-06	heme binding
Δgat1	8-day	2619716	down	GO:0020037	1,67E-06	heme binding
Δgat1	8-day	2622782	down	GO:0020037	1,67E-06	heme binding
Δgat1	8-day	2622893	down	GO:0020037	1,67E-06	heme binding
Δgat1	8-day	2624973	down	GO:0020037	1,67E-06	heme binding
Δgat1	8-day	2635870	down	GO:0020037	1,67E-06	heme binding
Δgat1	8-day	2637467	down	GO:0020037	1,67E-06	heme binding
Δgat1	8-day	2640585	down	GO:0020037	1,67E-06	heme binding
Δgat1	8-day	2673121	down	GO:0020037	1,67E-06	heme binding
Δgat1	8-day	1120318	down	GO:0006118	1,85E-06	electron transport
Δgat1	8-day	1342670	down	GO:0006118	1,85E-06	electron transport
Δgat1	8-day	1358353	down	GO:0006118	1,85E-06	electron transport
Δgat1	8-day	2364606	down	GO:0006118	1,85E-06	electron transport
Δgat1	8-day	2490194	down	GO:0006118	1,85E-06	electron transport
Δgat1	8-day	2491624	down	GO:0006118	1,85E-06	electron transport
Δgat1	8-day	2501258	down	GO:0006118	1,85E-06	electron transport
Δgat1	8-day	2508276	down	GO:0006118	1,85E-06	electron transport
Δgat1	8-day	2514636	down	GO:0006118	1,85E-06	electron transport
Δgat1	8-day	2537529	down	GO:0006118	1,85E-06	electron transport
Δgat1	8-day	2559987	down	GO:0006118	1,85E-06	electron transport
Δgat1	8-day	2573393	down	GO:0006118	1,85E-06	electron transport
Δgat1	8-day	2573396	down	GO:0006118	1,85E-06	electron transport
Δgat1	8-day	2605889	down	GO:0006118	1,85E-06	electron transport

Δgat1	8-day	2619716	down	GO:0006118	1,85E-06	electron transport
Δgat1	8-day	2622782	down	GO:0006118	1,85E-06	electron transport
Δgat1	8-day	2624973	down	GO:0006118	1,85E-06	electron transport
Δgat1	8-day	2628120	down	GO:0006118	1,85E-06	electron transport
Δgat1	8-day	2635870	down	GO:0006118	1,85E-06	electron transport
Δgat1	8-day	2637467	down	GO:0006118	1,85E-06	electron transport
Δgat1	8-day	2638473	down	GO:0006118	1,85E-06	electron transport
Δgat1	8-day	2666009	down	GO:0006118	1,85E-06	electron transport
Δgat1	8-day	2703927	down	GO:0006118	1,85E-06	electron transport
Δgat1	8-day	1120318	down	GO:0004497	4,91E-06	monooxygenase activity
Δgat1	8-day	1342670	down	GO:0004497	4,91E-06	monooxygenase activity
Δgat1	8-day	1358353	down	GO:0004497	4,91E-06	monooxygenase activity
Δgat1	8-day	2364606	down	GO:0004497	4,91E-06	monooxygenase activity
Δgat1	8-day	2490194	down	GO:0004497	4,91E-06	monooxygenase activity
Δgat1	8-day	2491624	down	GO:0004497	4,91E-06	monooxygenase activity
Δgat1	8-day	2501258	down	GO:0004497	4,91E-06	monooxygenase activity
Δgat1	8-day	2514636	down	GO:0004497	4,91E-06	monooxygenase activity
Δgat1	8-day	2537529	down	GO:0004497	4,91E-06	monooxygenase activity
Δgat1	8-day	2559987	down	GO:0004497	4,91E-06	monooxygenase activity
Δgat1	8-day	2573393	down	GO:0004497	4,91E-06	monooxygenase activity
Δgat1	8-day	2573396	down	GO:0004497	4,91E-06	monooxygenase activity
Δgat1	8-day	2619716	down	GO:0004497	4,91E-06	monooxygenase activity
Δgat1	8-day	2622782	down	GO:0004497	4,91E-06	monooxygenase activity
Δgat1	8-day	2624973	down	GO:0004497	4,91E-06	monooxygenase activity
Δgat1	8-day	2635870	down	GO:0004497	4,91E-06	monooxygenase activity
Δgat1	8-day	2637467	down	GO:0004497	4,91E-06	monooxygenase activity
Δgat1	8-day	2703927	down	GO:0004497	4,91E-06	monooxygenase activity
Δgat1	8-day	1158800	down	GO:0005524	0,000112675	ATP binding
Δgat1	8-day	1172108	down	GO:0005524	0,000112675	ATP binding
Δgat1	8-day	1181112	down	GO:0005524	0,000112675	ATP binding
Δgat1	8-day	2484205	down	GO:0005524	0,000112675	ATP binding
Δgat1	8-day	2490823	down	GO:0005524	0,000112675	ATP binding
Δgat1	8-day	2510275	down	GO:0005524	0,000112675	ATP binding
Δgat1	8-day	2510445	down	GO:0005524	0,000112675	ATP binding

Δgat1	8-day	2510924	down	GO:0005524	0,000112675	ATP binding
Δgat1	8-day	2581797	down	GO:0005524	0,000112675	ATP binding
Δgat1	8-day	2621533	down	GO:0005524	0,000112675	ATP binding
Δgat1	8-day	2633620	down	GO:0005524	0,000112675	ATP binding
Δgat1	8-day	2640828	down	GO:0005524	0,000112675	ATP binding
Δgat1	8-day	2641678	down	GO:0005524	0,000112675	ATP binding
Δgat1	8-day	2661304	down	GO:0005524	0,000112675	ATP binding
Δgat1	8-day	2675859	down	GO:0005524	0,000112675	ATP binding
Δgat1	8-day	2636220	down	GO:0004316	0,000335515	3-oxoacyl-[acyl-carrier-protein] reductase activity
Δgat1	8-day	2636221	down	GO:0004316	0,000335515	3-oxoacyl-[acyl-carrier-protein] reductase activity
Δgat1	8-day	2613657	down	GO:0004553	0,000521035	hydrolase activity, hydrolyzing O-glycosyl compounds
Δgat1	8-day	2670422	down	GO:0004553	0,000521035	hydrolase activity, hydrolyzing O-glycosyl compounds
Δgat1	8-day	1120318	down	GO:0050381	0,001125957	unspecific monooxygenase activity
Δgat1	8-day	1358353	down	GO:0050381	0,001125957	unspecific monooxygenase activity
Δgat1	8-day	2491624	down	GO:0050381	0,001125957	unspecific monooxygenase activity
Δgat1	8-day	2514636	down	GO:0050381	0,001125957	unspecific monooxygenase activity
Δgat1	8-day	2559987	down	GO:0050381	0,001125957	unspecific monooxygenase activity
Δgat1	8-day	2619716	down	GO:0050381	0,001125957	unspecific monooxygenase activity
Δgat1	8-day	2624973	down	GO:0050381	0,001125957	unspecific monooxygenase activity
Δgat1	8-day	2637467	down	GO:0050381	0,001125957	unspecific monooxygenase activity
Δgat1	8-day	1171024	down	GO:0005215	0,003960324	transporter activity
Δgat1	8-day	2508421	down	GO:0005215	0,003960324	transporter activity
Δgat1	8-day	2533765	down	GO:0005215	0,003960324	transporter activity
Δgat1	8-day	2550496	down	GO:0005215	0,003960324	transporter activity
Δgat1	8-day	2608744	down	GO:0005215	0,003960324	transporter activity
Δgat1	8-day	2611816	down	GO:0005215	0,003960324	transporter activity
Δgat1	8-day	2617636	down	GO:0005215	0,003960324	transporter activity
Δgat1	8-day	2619010	down	GO:0005215	0,003960324	transporter activity
Δgat1	8-day	2628098	down	GO:0005215	0,003960324	transporter activity
Δgat1	8-day	2630257	down	GO:0005215	0,003960324	transporter activity
Δgat1	8-day	2631348	down	GO:0005215	0,003960324	transporter activity
Δgat1	8-day	2632105	down	GO:0005215	0,003960324	transporter activity
Δgat1	8-day	1083109	down	GO:0016491	0,012094047	oxidoreductase activity
Δgat1	8-day	1087819	down	GO:0016491	0,012094047	oxidoreductase activity

Δgat1	8-day	1194451	down	GO:0016491	0,012094047	oxidoreductase activity
Δgat1	8-day	1214326	down	GO:0016491	0,012094047	oxidoreductase activity
Δgat1	8-day	1342670	down	GO:0016491	0,012094047	oxidoreductase activity
Δgat1	8-day	2490194	down	GO:0016491	0,012094047	oxidoreductase activity
Δgat1	8-day	2501673	down	GO:0016491	0,012094047	oxidoreductase activity
Δgat1	8-day	2508276	down	GO:0016491	0,012094047	oxidoreductase activity
Δgat1	8-day	2516955	down	GO:0016491	0,012094047	oxidoreductase activity
Δgat1	8-day	2517867	down	GO:0016491	0,012094047	oxidoreductase activity
Δgat1	8-day	2573393	down	GO:0016491	0,012094047	oxidoreductase activity
Δgat1	8-day	2573396	down	GO:0016491	0,012094047	oxidoreductase activity
Δgat1	8-day	2606041	down	GO:0016491	0,012094047	oxidoreductase activity
Δgat1	8-day	2614722	down	GO:0016491	0,012094047	oxidoreductase activity
Δgat1	8-day	2625836	down	GO:0016491	0,012094047	oxidoreductase activity
Δgat1	8-day	2626155	down	GO:0016491	0,012094047	oxidoreductase activity
Δgat1	8-day	2627113	down	GO:0016491	0,012094047	oxidoreductase activity
Δgat1	8-day	2628120	down	GO:0016491	0,012094047	oxidoreductase activity
Δgat1	8-day	2630033	down	GO:0016491	0,012094047	oxidoreductase activity
Δgat1	8-day	2632193	down	GO:0016491	0,012094047	oxidoreductase activity
Δgat1	8-day	2634619	down	GO:0016491	0,012094047	oxidoreductase activity
Δgat1	8-day	2636220	down	GO:0016491	0,012094047	oxidoreductase activity
Δgat1	8-day	2636221	down	GO:0016491	0,012094047	oxidoreductase activity
Δgat1	8-day	2637838	down	GO:0016491	0,012094047	oxidoreductase activity
Δgat1	8-day	2638445	down	GO:0016491	0,012094047	oxidoreductase activity
Δgat1	8-day	2638473	down	GO:0016491	0,012094047	oxidoreductase activity
Δgat1	8-day	2638753	down	GO:0016491	0,012094047	oxidoreductase activity
Δgat1	8-day	2640123	down	GO:0016491	0,012094047	oxidoreductase activity
Δgat1	8-day	2644003	down	GO:0016491	0,012094047	oxidoreductase activity
Δgat1	8-day	2666009	down	GO:0016491	0,012094047	oxidoreductase activity
Δgat1	8-day	2681649	down	GO:0016491	0,012094047	oxidoreductase activity
Δgat1	8-day	2691273	down	GO:0016491	0,012094047	oxidoreductase activity
Δgat1	8-day	2698778	down	GO:0016491	0,012094047	oxidoreductase activity
Δgat1	8-day	2703927	down	GO:0016491	0,012094047	oxidoreductase activity
Δgat1	8-day	2713107	down	GO:0016491	0,012094047	oxidoreductase activity
Δgat1	8-day	2748659	down	GO:0016491	0,012094047	oxidoreductase activity

Δhom1	8-day	2364606	down	GO:0004497	0,001917923	monooxygenase activity
Δhom1	8-day	2635487	down	GO:0004497	0,001917923	monooxygenase activity
Δhom1	8-day	2635869	down	GO:0004497	0,001917923	monooxygenase activity
Δhom1	8-day	2638355	down	GO:0004497	0,001917923	monooxygenase activity
Δhom1	8-day	2703628	down	GO:0004497	0,001917923	monooxygenase activity
Δhom1	8-day	2502504	down	GO:0005975	0,001917923	carbohydrate metabolic process
Δhom1	8-day	2514546	down	GO:0005975	0,001917923	carbohydrate metabolic process
Δhom1	8-day	2605157	down	GO:0005975	0,001917923	carbohydrate metabolic process
Δhom1	8-day	2612719	down	GO:0005975	0,001917923	carbohydrate metabolic process
Δhom1	8-day	2613657	down	GO:0005975	0,001917923	carbohydrate metabolic process
Δhom1	8-day	2636145	down	GO:0005975	0,001917923	carbohydrate metabolic process
Δhom1	8-day	2645945	down	GO:0005975	0,001917923	carbohydrate metabolic process
Δhom1	8-day	2502504	down	GO:0004553	0,001917923	hydrolase activity, hydrolyzing O-glycosyl compounds
Δhom1	8-day	2605157	down	GO:0004553	0,001917923	hydrolase activity, hydrolyzing O-glycosyl compounds
Δhom1	8-day	2612719	down	GO:0004553	0,001917923	hydrolase activity, hydrolyzing O-glycosyl compounds
Δhom1	8-day	2613657	down	GO:0004553	0,001917923	hydrolase activity, hydrolyzing O-glycosyl compounds
Δhom1	8-day	1214326	down	GO:0016491	0,003228097	oxidoreductase activity
Δhom1	8-day	2511002	down	GO:0016491	0,003228097	oxidoreductase activity
Δhom1	8-day	2606041	down	GO:0016491	0,003228097	oxidoreductase activity
Δhom1	8-day	2614092	down	GO:0016491	0,003228097	oxidoreductase activity
Δhom1	8-day	2618220	down	GO:0016491	0,003228097	oxidoreductase activity
Δhom1	8-day	2623663	down	GO:0016491	0,003228097	oxidoreductase activity
Δhom1	8-day	2623957	down	GO:0016491	0,003228097	oxidoreductase activity
Δhom1	8-day	2630033	down	GO:0016491	0,003228097	oxidoreductase activity
Δhom1	8-day	2637838	down	GO:0016491	0,003228097	oxidoreductase activity
Δhom1	8-day	2638893	down	GO:0016491	0,003228097	oxidoreductase activity
Δhom1	8-day	2639682	down	GO:0016491	0,003228097	oxidoreductase activity
Δhom1	8-day	2698778	down	GO:0016491	0,003228097	oxidoreductase activity
Δhom1	8-day	2713107	down	GO:0016491	0,003228097	oxidoreductase activity
Δhom1	8-day	2364606	down	GO:0006118	0,005982409	electron transport
Δhom1	8-day	2614092	down	GO:0006118	0,005982409	electron transport
Δhom1	8-day	2635487	down	GO:0006118	0,005982409	electron transport
Δhom1	8-day	2635869	down	GO:0006118	0,005982409	electron transport
Δhom1	8-day	2638355	down	GO:0006118	0,005982409	electron transport

Δhom1	8-day	2639682	down	GO:0006118	0,005982409	electron transport
Δhom1	8-day	2703628	down	GO:0006118	0,005982409	electron transport
Δhom1	8-day	2364606	down	GO:0005506	0,04915661	iron ion binding
Δhom1	8-day	2511002	down	GO:0005506	0,04915661	iron ion binding
Δhom1	8-day	2618220	down	GO:0005506	0,04915661	iron ion binding
Δhom1	8-day	2635487	down	GO:0005506	0,04915661	iron ion binding
Δhom1	8-day	2635869	down	GO:0005506	0,04915661	iron ion binding
Δhom1	8-day	2636420	down	GO:0005506	0,04915661	iron ion binding
Δhom1	8-day	2638355	down	GO:0005506	0,04915661	iron ion binding
Δhom1	8-day	2703628	down	GO:0005506	0,04915661	iron ion binding
Δhom2	8-day	2514546	up	GO:0005975	1,33E-19	carbohydrate metabolic process
Δhom2	8-day	2611511	up	GO:0005975	1,33E-19	carbohydrate metabolic process
Δhom2	8-day	2613657	up	GO:0005975	1,33E-19	carbohydrate metabolic process
Δhom2	8-day	2626756	up	GO:0005975	1,33E-19	carbohydrate metabolic process
Δhom2	8-day	2633791	up	GO:0005975	1,33E-19	carbohydrate metabolic process
Δhom2	8-day	2641020	up	GO:0005975	1,33E-19	carbohydrate metabolic process
Δhom2	8-day	2643740	up	GO:0005975	1,33E-19	carbohydrate metabolic process
Δhom2	8-day	2645822	up	GO:0005975	1,33E-19	carbohydrate metabolic process
Δhom2	8-day	2670422	up	GO:0005975	1,33E-19	carbohydrate metabolic process
Δhom2	8-day	2704222	up	GO:0005975	1,33E-19	carbohydrate metabolic process
Δhom2	8-day	2706622	up	GO:0005975	1,33E-19	carbohydrate metabolic process
Δhom2	8-day	2611511	up	GO:0004553	3,41E-17	hydrolase activity, hydrolyzing O-glycosyl compounds
Δhom2	8-day	2613657	up	GO:0004553	3,41E-17	hydrolase activity, hydrolyzing O-glycosyl compounds
Δhom2	8-day	2643740	up	GO:0004553	3,41E-17	hydrolase activity, hydrolyzing O-glycosyl compounds
Δhom2	8-day	2645822	up	GO:0004553	3,41E-17	hydrolase activity, hydrolyzing O-glycosyl compounds
Δhom2	8-day	2670422	up	GO:0004553	3,41E-17	hydrolase activity, hydrolyzing O-glycosyl compounds
Δhom2	8-day	2704222	up	GO:0004553	3,41E-17	hydrolase activity, hydrolyzing O-glycosyl compounds
Δhom2	8-day	2706622	up	GO:0004553	3,41E-17	hydrolase activity, hydrolyzing O-glycosyl compounds
Δhom2	8-day	2643740	up	GO:0030246	2,52E-07	carbohydrate binding
Δhom2	8-day	2706622	up	GO:0030246	2,52E-07	carbohydrate binding
Δhom2	8-day	48473	up	GO:0003824	1,44E-06	catalytic activity
Δhom2	8-day	1150509	up	GO:0003824	1,44E-06	catalytic activity
Δhom2	8-day	2491365	up	GO:0003824	1,44E-06	catalytic activity
Δhom2	8-day	2502008	up	GO:0003824	1,44E-06	catalytic activity

Δhom2	8-day	2514546	up	GO:0003824	1,44E-06	catalytic activity
Δhom2	8-day	2516444	up	GO:0003824	1,44E-06	catalytic activity
Δhom2	8-day	2516959	up	GO:0003824	1,44E-06	catalytic activity
Δhom2	8-day	2592175	up	GO:0003824	1,44E-06	catalytic activity
Δhom2	8-day	2594114	up	GO:0003824	1,44E-06	catalytic activity
Δhom2	8-day	2614113	up	GO:0003824	1,44E-06	catalytic activity
Δhom2	8-day	2616512	up	GO:0003824	1,44E-06	catalytic activity
Δhom2	8-day	2617245	up	GO:0003824	1,44E-06	catalytic activity
Δhom2	8-day	2618252	up	GO:0003824	1,44E-06	catalytic activity
Δhom2	8-day	2623957	up	GO:0003824	1,44E-06	catalytic activity
Δhom2	8-day	2626155	up	GO:0003824	1,44E-06	catalytic activity
Δhom2	8-day	2626172	up	GO:0003824	1,44E-06	catalytic activity
Δhom2	8-day	2626756	up	GO:0003824	1,44E-06	catalytic activity
Δhom2	8-day	2627304	up	GO:0003824	1,44E-06	catalytic activity
Δhom2	8-day	2632193	up	GO:0003824	1,44E-06	catalytic activity
Δhom2	8-day	2634217	up	GO:0003824	1,44E-06	catalytic activity
Δhom2	8-day	2634903	up	GO:0003824	1,44E-06	catalytic activity
Δhom2	8-day	2635219	up	GO:0003824	1,44E-06	catalytic activity
Δhom2	8-day	2636220	up	GO:0003824	1,44E-06	catalytic activity
Δhom2	8-day	2638445	up	GO:0003824	1,44E-06	catalytic activity
Δhom2	8-day	2638893	up	GO:0003824	1,44E-06	catalytic activity
Δhom2	8-day	2640123	up	GO:0003824	1,44E-06	catalytic activity
Δhom2	8-day	2641020	up	GO:0003824	1,44E-06	catalytic activity
Δhom2	8-day	2641553	up	GO:0003824	1,44E-06	catalytic activity
Δhom2	8-day	2681649	up	GO:0003824	1,44E-06	catalytic activity
Δhom2	8-day	2682272	up	GO:0003824	1,44E-06	catalytic activity
Δhom2	8-day	2684249	up	GO:0003824	1,44E-06	catalytic activity
Δhom2	8-day	2748659	up	GO:0003824	1,44E-06	catalytic activity
Δhom2	8-day	1194451	up	GO:0016491	7,27E-06	oxidoreductase activity
Δhom2	8-day	1342670	up	GO:0016491	7,27E-06	oxidoreductase activity
Δhom2	8-day	2490194	up	GO:0016491	7,27E-06	oxidoreductase activity
Δhom2	8-day	2501673	up	GO:0016491	7,27E-06	oxidoreductase activity
Δhom2	8-day	2506612	up	GO:0016491	7,27E-06	oxidoreductase activity
Δhom2	8-day	2509814	up	GO:0016491	7,27E-06	oxidoreductase activity

Δhom2	8-day	2516444	up	GO:0016491	7,27E-06	oxidoreductase activity
Δhom2	8-day	2516959	up	GO:0016491	7,27E-06	oxidoreductase activity
Δhom2	8-day	2524219	up	GO:0016491	7,27E-06	oxidoreductase activity
Δhom2	8-day	2557571	up	GO:0016491	7,27E-06	oxidoreductase activity
Δhom2	8-day	2573393	up	GO:0016491	7,27E-06	oxidoreductase activity
Δhom2	8-day	2573396	up	GO:0016491	7,27E-06	oxidoreductase activity
Δhom2	8-day	2583399	up	GO:0016491	7,27E-06	oxidoreductase activity
Δhom2	8-day	2606041	up	GO:0016491	7,27E-06	oxidoreductase activity
Δhom2	8-day	2611287	up	GO:0016491	7,27E-06	oxidoreductase activity
Δhom2	8-day	2617060	up	GO:0016491	7,27E-06	oxidoreductase activity
Δhom2	8-day	2622733	up	GO:0016491	7,27E-06	oxidoreductase activity
Δhom2	8-day	2623957	up	GO:0016491	7,27E-06	oxidoreductase activity
Δhom2	8-day	2625241	up	GO:0016491	7,27E-06	oxidoreductase activity
Δhom2	8-day	2625836	up	GO:0016491	7,27E-06	oxidoreductase activity
Δhom2	8-day	2626155	up	GO:0016491	7,27E-06	oxidoreductase activity
Δhom2	8-day	2627304	up	GO:0016491	7,27E-06	oxidoreductase activity
Δhom2	8-day	2632193	up	GO:0016491	7,27E-06	oxidoreductase activity
Δhom2	8-day	2632403	up	GO:0016491	7,27E-06	oxidoreductase activity
Δhom2	8-day	2634217	up	GO:0016491	7,27E-06	oxidoreductase activity
Δhom2	8-day	2634619	up	GO:0016491	7,27E-06	oxidoreductase activity
Δhom2	8-day	2634903	up	GO:0016491	7,27E-06	oxidoreductase activity
Δhom2	8-day	2636220	up	GO:0016491	7,27E-06	oxidoreductase activity
Δhom2	8-day	2638445	up	GO:0016491	7,27E-06	oxidoreductase activity
Δhom2	8-day	2638473	up	GO:0016491	7,27E-06	oxidoreductase activity
Δhom2	8-day	2638753	up	GO:0016491	7,27E-06	oxidoreductase activity
Δhom2	8-day	2638893	up	GO:0016491	7,27E-06	oxidoreductase activity
Δhom2	8-day	2640123	up	GO:0016491	7,27E-06	oxidoreductase activity
Δhom2	8-day	2681649	up	GO:0016491	7,27E-06	oxidoreductase activity
Δhom2	8-day	2691273	up	GO:0016491	7,27E-06	oxidoreductase activity
Δhom2	8-day	2703927	up	GO:0016491	7,27E-06	oxidoreductase activity
Δhom2	8-day	2713107	up	GO:0016491	7,27E-06	oxidoreductase activity
Δhom2	8-day	2748659	up	GO:0016491	7,27E-06	oxidoreductase activity
Δhom2	8-day	1111500	up	GO:0005215	3,03E-05	transporter activity
Δhom2	8-day	1154281	up	GO:0005215	3,03E-05	transporter activity

Δhom2	8-day	1234296	up	GO:0005215	3,03E-05	transporter activity
Δhom2	8-day	2145491	up	GO:0005215	3,03E-05	transporter activity
Δhom2	8-day	2487072	up	GO:0005215	3,03E-05	transporter activity
Δhom2	8-day	2488479	up	GO:0005215	3,03E-05	transporter activity
Δhom2	8-day	2502284	up	GO:0005215	3,03E-05	transporter activity
Δhom2	8-day	2508421	up	GO:0005215	3,03E-05	transporter activity
Δhom2	8-day	2508697	up	GO:0005215	3,03E-05	transporter activity
Δhom2	8-day	2600551	up	GO:0005215	3,03E-05	transporter activity
Δhom2	8-day	2612313	up	GO:0005215	3,03E-05	transporter activity
Δhom2	8-day	2617636	up	GO:0005215	3,03E-05	transporter activity
Δhom2	8-day	2618842	up	GO:0005215	3,03E-05	transporter activity
Δhom2	8-day	2619010	up	GO:0005215	3,03E-05	transporter activity
Δhom2	8-day	2623043	up	GO:0005215	3,03E-05	transporter activity
Δhom2	8-day	2628098	up	GO:0005215	3,03E-05	transporter activity
Δhom2	8-day	2628982	up	GO:0005215	3,03E-05	transporter activity
Δhom2	8-day	2631348	up	GO:0005215	3,03E-05	transporter activity
Δhom2	8-day	2632105	up	GO:0005215	3,03E-05	transporter activity
Δhom2	8-day	2633216	up	GO:0005215	3,03E-05	transporter activity
Δhom2	8-day	2633874	up	GO:0005215	3,03E-05	transporter activity
Δhom2	8-day	2641735	up	GO:0005215	3,03E-05	transporter activity
Δhom2	8-day	2642894	up	GO:0005215	3,03E-05	transporter activity
Δhom2	8-day	2738153	up	GO:0005215	3,03E-05	transporter activity
Δhom2	8-day	48473	up	GO:0008152	7,83E-05	metabolic process
Δhom2	8-day	1099466	up	GO:0008152	7,83E-05	metabolic process
Δhom2	8-day	1150509	up	GO:0008152	7,83E-05	metabolic process
Δhom2	8-day	1202278	up	GO:0008152	7,83E-05	metabolic process
Δhom2	8-day	1279479	up	GO:0008152	7,83E-05	metabolic process
Δhom2	8-day	1342670	up	GO:0008152	7,83E-05	metabolic process
Δhom2	8-day	2490194	up	GO:0008152	7,83E-05	metabolic process
Δhom2	8-day	2491365	up	GO:0008152	7,83E-05	metabolic process
Δhom2	8-day	2491542	up	GO:0008152	7,83E-05	metabolic process
Δhom2	8-day	2501673	up	GO:0008152	7,83E-05	metabolic process
Δhom2	8-day	2502008	up	GO:0008152	7,83E-05	metabolic process
Δhom2	8-day	2516959	up	GO:0008152	7,83E-05	metabolic process

Δhom2	8-day	2557571	up	GO:0008152	7,83E-05	metabolic process
Δhom2	8-day	2573393	up	GO:0008152	7,83E-05	metabolic process
Δhom2	8-day	2573396	up	GO:0008152	7,83E-05	metabolic process
Δhom2	8-day	2583540	up	GO:0008152	7,83E-05	metabolic process
Δhom2	8-day	2589496	up	GO:0008152	7,83E-05	metabolic process
Δhom2	8-day	2594114	up	GO:0008152	7,83E-05	metabolic process
Δhom2	8-day	2606041	up	GO:0008152	7,83E-05	metabolic process
Δhom2	8-day	2616280	up	GO:0008152	7,83E-05	metabolic process
Δhom2	8-day	2616512	up	GO:0008152	7,83E-05	metabolic process
Δhom2	8-day	2617245	up	GO:0008152	7,83E-05	metabolic process
Δhom2	8-day	2622385	up	GO:0008152	7,83E-05	metabolic process
Δhom2	8-day	2623957	up	GO:0008152	7,83E-05	metabolic process
Δhom2	8-day	2624525	up	GO:0008152	7,83E-05	metabolic process
Δhom2	8-day	2624569	up	GO:0008152	7,83E-05	metabolic process
Δhom2	8-day	2625241	up	GO:0008152	7,83E-05	metabolic process
Δhom2	8-day	2625836	up	GO:0008152	7,83E-05	metabolic process
Δhom2	8-day	2626172	up	GO:0008152	7,83E-05	metabolic process
Δhom2	8-day	2627304	up	GO:0008152	7,83E-05	metabolic process
Δhom2	8-day	2628358	up	GO:0008152	7,83E-05	metabolic process
Δhom2	8-day	2632193	up	GO:0008152	7,83E-05	metabolic process
Δhom2	8-day	2634217	up	GO:0008152	7,83E-05	metabolic process
Δhom2	8-day	2634903	up	GO:0008152	7,83E-05	metabolic process
Δhom2	8-day	2636220	up	GO:0008152	7,83E-05	metabolic process
Δhom2	8-day	2638445	up	GO:0008152	7,83E-05	metabolic process
Δhom2	8-day	2638473	up	GO:0008152	7,83E-05	metabolic process
Δhom2	8-day	2641553	up	GO:0008152	7,83E-05	metabolic process
Δhom2	8-day	2668051	up	GO:0008152	7,83E-05	metabolic process
Δhom2	8-day	2681649	up	GO:0008152	7,83E-05	metabolic process
Δhom2	8-day	2682272	up	GO:0008152	7,83E-05	metabolic process
Δhom2	8-day	2696676	up	GO:0008152	7,83E-05	metabolic process
Δhom2	8-day	2703927	up	GO:0008152	7,83E-05	metabolic process
Δhom2	8-day	2713107	up	GO:0008152	7,83E-05	metabolic process
Δhom2	8-day	2726410	up	GO:0008152	7,83E-05	metabolic process
Δhom2	8-day	2749557	up	GO:0008152	7,83E-05	metabolic process

Δhom2	8-day	1133978	up	GO:0016021	9,97E-05	integral to membrane
Δhom2	8-day	1154281	up	GO:0016021	9,97E-05	integral to membrane
Δhom2	8-day	1234296	up	GO:0016021	9,97E-05	integral to membrane
Δhom2	8-day	2145491	up	GO:0016021	9,97E-05	integral to membrane
Δhom2	8-day	2279823	up	GO:0016021	9,97E-05	integral to membrane
Δhom2	8-day	2487072	up	GO:0016021	9,97E-05	integral to membrane
Δhom2	8-day	2488479	up	GO:0016021	9,97E-05	integral to membrane
Δhom2	8-day	2505704	up	GO:0016021	9,97E-05	integral to membrane
Δhom2	8-day	2508421	up	GO:0016021	9,97E-05	integral to membrane
Δhom2	8-day	2508697	up	GO:0016021	9,97E-05	integral to membrane
Δhom2	8-day	2612313	up	GO:0016021	9,97E-05	integral to membrane
Δhom2	8-day	2612912	up	GO:0016021	9,97E-05	integral to membrane
Δhom2	8-day	2617636	up	GO:0016021	9,97E-05	integral to membrane
Δhom2	8-day	2618842	up	GO:0016021	9,97E-05	integral to membrane
Δhom2	8-day	2619010	up	GO:0016021	9,97E-05	integral to membrane
Δhom2	8-day	2623043	up	GO:0016021	9,97E-05	integral to membrane
Δhom2	8-day	2628982	up	GO:0016021	9,97E-05	integral to membrane
Δhom2	8-day	2631348	up	GO:0016021	9,97E-05	integral to membrane
Δhom2	8-day	2632105	up	GO:0016021	9,97E-05	integral to membrane
Δhom2	8-day	2633216	up	GO:0016021	9,97E-05	integral to membrane
Δhom2	8-day	2633874	up	GO:0016021	9,97E-05	integral to membrane
Δhom2	8-day	2635198	up	GO:0016021	9,97E-05	integral to membrane
Δhom2	8-day	2638969	up	GO:0016021	9,97E-05	integral to membrane
Δhom2	8-day	2642116	up	GO:0016021	9,97E-05	integral to membrane
Δhom2	8-day	2642894	up	GO:0016021	9,97E-05	integral to membrane
Δhom2	8-day	2675859	up	GO:0016021	9,97E-05	integral to membrane
Δhom2	8-day	78628	up	GO:0006118	0,003343327	electron transport
Δhom2	8-day	1120318	up	GO:0006118	0,003343327	electron transport
Δhom2	8-day	1215660	up	GO:0006118	0,003343327	electron transport
Δhom2	8-day	1342670	up	GO:0006118	0,003343327	electron transport
Δhom2	8-day	2490194	up	GO:0006118	0,003343327	electron transport
Δhom2	8-day	2491624	up	GO:0006118	0,003343327	electron transport
Δhom2	8-day	2501258	up	GO:0006118	0,003343327	electron transport
Δhom2	8-day	2514636	up	GO:0006118	0,003343327	electron transport

Δhom2	8-day	2516208	up	GO:0006118	0,003343327	electron transport
Δhom2	8-day	2517480	up	GO:0006118	0,003343327	electron transport
Δhom2	8-day	2557571	up	GO:0006118	0,003343327	electron transport
Δhom2	8-day	2573393	up	GO:0006118	0,003343327	electron transport
Δhom2	8-day	2573396	up	GO:0006118	0,003343327	electron transport
Δhom2	8-day	2583399	up	GO:0006118	0,003343327	electron transport
Δhom2	8-day	2605889	up	GO:0006118	0,003343327	electron transport
Δhom2	8-day	2614998	up	GO:0006118	0,003343327	electron transport
Δhom2	8-day	2616590	up	GO:0006118	0,003343327	electron transport
Δhom2	8-day	2622733	up	GO:0006118	0,003343327	electron transport
Δhom2	8-day	2628651	up	GO:0006118	0,003343327	electron transport
Δhom2	8-day	2629864	up	GO:0006118	0,003343327	electron transport
Δhom2	8-day	2633776	up	GO:0006118	0,003343327	electron transport
Δhom2	8-day	2635870	up	GO:0006118	0,003343327	electron transport
Δhom2	8-day	2637467	up	GO:0006118	0,003343327	electron transport
Δhom2	8-day	2638282	up	GO:0006118	0,003343327	electron transport
Δhom2	8-day	2638350	up	GO:0006118	0,003343327	electron transport
Δhom2	8-day	2638355	up	GO:0006118	0,003343327	electron transport
Δhom2	8-day	2638473	up	GO:0006118	0,003343327	electron transport
Δhom2	8-day	2640550	up	GO:0006118	0,003343327	electron transport
Δhom2	8-day	2666440	up	GO:0006118	0,003343327	electron transport
Δhom2	8-day	2703927	up	GO:0006118	0,003343327	electron transport
Δhom2	8-day	2753029	up	GO:0006118	0,003343327	electron transport
Δhom2	8-day	1183234	up	GO:0030170	0,003343327	pyridoxal phosphate binding
Δhom2	8-day	2560780	up	GO:0030170	0,003343327	pyridoxal phosphate binding
Δhom2	8-day	2625520	up	GO:0030170	0,003343327	pyridoxal phosphate binding
Δhom2	8-day	2619010	up	GO:0008643	0,003777816	carbohydrate transport
Δhom2	8-day	2623043	up	GO:0008643	0,003777816	carbohydrate transport
Δhom2	8-day	1111500	up	GO:0006810	0,004083984	transport
Δhom2	8-day	1133978	up	GO:0006810	0,004083984	transport
Δhom2	8-day	1154281	up	GO:0006810	0,004083984	transport
Δhom2	8-day	1234296	up	GO:0006810	0,004083984	transport
Δhom2	8-day	2145491	up	GO:0006810	0,004083984	transport
Δhom2	8-day	2279823	up	GO:0006810	0,004083984	transport

Δhom2	8-day	2487072	up	GO:0006810	0,004083984	transport
Δhom2	8-day	2488479	up	GO:0006810	0,004083984	transport
Δhom2	8-day	2493264	up	GO:0006810	0,004083984	transport
Δhom2	8-day	2502284	up	GO:0006810	0,004083984	transport
Δhom2	8-day	2508421	up	GO:0006810	0,004083984	transport
Δhom2	8-day	2508697	up	GO:0006810	0,004083984	transport
Δhom2	8-day	2525886	up	GO:0006810	0,004083984	transport
Δhom2	8-day	2600551	up	GO:0006810	0,004083984	transport
Δhom2	8-day	2612313	up	GO:0006810	0,004083984	transport
Δhom2	8-day	2612912	up	GO:0006810	0,004083984	transport
Δhom2	8-day	2617458	up	GO:0006810	0,004083984	transport
Δhom2	8-day	2617636	up	GO:0006810	0,004083984	transport
Δhom2	8-day	2618842	up	GO:0006810	0,004083984	transport
Δhom2	8-day	2619010	up	GO:0006810	0,004083984	transport
Δhom2	8-day	2620257	up	GO:0006810	0,004083984	transport
Δhom2	8-day	2622049	up	GO:0006810	0,004083984	transport
Δhom2	8-day	2623043	up	GO:0006810	0,004083984	transport
Δhom2	8-day	2628098	up	GO:0006810	0,004083984	transport
Δhom2	8-day	2628982	up	GO:0006810	0,004083984	transport
Δhom2	8-day	2631348	up	GO:0006810	0,004083984	transport
Δhom2	8-day	2631822	up	GO:0006810	0,004083984	transport
Δhom2	8-day	2632105	up	GO:0006810	0,004083984	transport
Δhom2	8-day	2633216	up	GO:0006810	0,004083984	transport
Δhom2	8-day	2633874	up	GO:0006810	0,004083984	transport
Δhom2	8-day	2635519	up	GO:0006810	0,004083984	transport
Δhom2	8-day	2636140	up	GO:0006810	0,004083984	transport
Δhom2	8-day	2638969	up	GO:0006810	0,004083984	transport
Δhom2	8-day	2641735	up	GO:0006810	0,004083984	transport
Δhom2	8-day	2642116	up	GO:0006810	0,004083984	transport
Δhom2	8-day	2642364	up	GO:0006810	0,004083984	transport
Δhom2	8-day	2642894	up	GO:0006810	0,004083984	transport
Δhom2	8-day	2675859	up	GO:0006810	0,004083984	transport
Δhom2	8-day	2738153	up	GO:0006810	0,004083984	transport
Δhom2	8-day	2619010	up	GO:0008733	0,004335371	L-arabinose isomerase activity

Δhom2	8-day	2623043	up	GO:0008733	0,004335371	L-arabinose isomerase activity
Δhom2	8-day	2627902	up	GO:0005576	0,004965807	extracellular region
Δhom2	8-day	2627923	up	GO:0005576	0,004965807	extracellular region
Δhom2	8-day	2643740	up	GO:0005576	0,004965807	extracellular region
Δhom2	8-day	2706622	up	GO:0005576	0,004965807	extracellular region
Δhom2	8-day	2619010	up	GO:0005351	0,00660336	sugar:hydrogen symporter activity
Δhom2	8-day	2623043	up	GO:0005351	0,00660336	sugar:hydrogen symporter activity
Δhom2	8-day	2501673	up	GO:0006520	0,022257414	amino acid metabolic process
Δhom2	8-day	78628	up	GO:0005506	0,025481173	iron ion binding
Δhom2	8-day	1120318	up	GO:0005506	0,025481173	iron ion binding
Δhom2	8-day	2491624	up	GO:0005506	0,025481173	iron ion binding
Δhom2	8-day	2501258	up	GO:0005506	0,025481173	iron ion binding
Δhom2	8-day	2514636	up	GO:0005506	0,025481173	iron ion binding
Δhom2	8-day	2516208	up	GO:0005506	0,025481173	iron ion binding
Δhom2	8-day	2517480	up	GO:0005506	0,025481173	iron ion binding
Δhom2	8-day	2616590	up	GO:0005506	0,025481173	iron ion binding
Δhom2	8-day	2617060	up	GO:0005506	0,025481173	iron ion binding
Δhom2	8-day	2628651	up	GO:0005506	0,025481173	iron ion binding
Δhom2	8-day	2629864	up	GO:0005506	0,025481173	iron ion binding
Δhom2	8-day	2633776	up	GO:0005506	0,025481173	iron ion binding
Δhom2	8-day	2635870	up	GO:0005506	0,025481173	iron ion binding
Δhom2	8-day	2637467	up	GO:0005506	0,025481173	iron ion binding
Δhom2	8-day	2638282	up	GO:0005506	0,025481173	iron ion binding
Δhom2	8-day	2638350	up	GO:0005506	0,025481173	iron ion binding
Δhom2	8-day	2638355	up	GO:0005506	0,025481173	iron ion binding
Δhom2	8-day	2640550	up	GO:0005506	0,025481173	iron ion binding
Δhom2	8-day	2666440	up	GO:0005506	0,025481173	iron ion binding
Δhom2	8-day	2753029	up	GO:0005506	0,025481173	iron ion binding
Δhom2	8-day	2626756	up	GO:0016810	0,025481173	hydrolase activity, acting on carbon-nitrogen (but not peptide) bonds
Δhom2	8-day	2633738	up	GO:0016810	0,025481173	hydrolase activity, acting on carbon-nitrogen (but not peptide) bonds
Δhom2	8-day	2641020	up	GO:0016810	0,025481173	hydrolase activity, acting on carbon-nitrogen (but not peptide) bonds

Δhom2	8-day	78628	up	GO:0004497	0,025481173	monooxygenase activity
Δhom2	8-day	1120318	up	GO:0004497	0,025481173	monooxygenase activity
Δhom2	8-day	1342670	up	GO:0004497	0,025481173	monooxygenase activity
Δhom2	8-day	2490194	up	GO:0004497	0,025481173	monooxygenase activity
Δhom2	8-day	2491624	up	GO:0004497	0,025481173	monooxygenase activity
Δhom2	8-day	2501258	up	GO:0004497	0,025481173	monooxygenase activity
Δhom2	8-day	2514636	up	GO:0004497	0,025481173	monooxygenase activity
Δhom2	8-day	2516208	up	GO:0004497	0,025481173	monooxygenase activity
Δhom2	8-day	2517480	up	GO:0004497	0,025481173	monooxygenase activity
Δhom2	8-day	2557571	up	GO:0004497	0,025481173	monooxygenase activity
Δhom2	8-day	2573393	up	GO:0004497	0,025481173	monooxygenase activity
Δhom2	8-day	2573396	up	GO:0004497	0,025481173	monooxygenase activity
Δhom2	8-day	2616590	up	GO:0004497	0,025481173	monooxygenase activity
Δhom2	8-day	2628651	up	GO:0004497	0,025481173	monooxygenase activity
Δhom2	8-day	2629864	up	GO:0004497	0,025481173	monooxygenase activity
Δhom2	8-day	2633776	up	GO:0004497	0,025481173	monooxygenase activity
Δhom2	8-day	2635870	up	GO:0004497	0,025481173	monooxygenase activity
Δhom2	8-day	2637467	up	GO:0004497	0,025481173	monooxygenase activity
Δhom2	8-day	2638282	up	GO:0004497	0,025481173	monooxygenase activity
Δhom2	8-day	2638350	up	GO:0004497	0,025481173	monooxygenase activity
Δhom2	8-day	2638355	up	GO:0004497	0,025481173	monooxygenase activity
Δhom2	8-day	2640550	up	GO:0004497	0,025481173	monooxygenase activity
Δhom2	8-day	2666440	up	GO:0004497	0,025481173	monooxygenase activity
Δhom2	8-day	2703927	up	GO:0004497	0,025481173	monooxygenase activity
Δhom2	8-day	2753029	up	GO:0004497	0,025481173	monooxygenase activity
Δhom2	8-day	2343034	up	GO:0016614	0,025481173	oxidoreductase activity, acting on CH-OH group of donors
Δhom2	8-day	2607677	up	GO:0016614	0,025481173	oxidoreductase activity, acting on CH-OH group of donors
Δhom2	8-day	2638753	up	GO:0016614	0,025481173	oxidoreductase activity, acting on CH-OH group of donors
Δhom2	8-day	2643062	up	GO:0016614	0,025481173	oxidoreductase activity, acting on CH-OH group of donors
Δhom2	8-day	2663917	up	GO:0016614	0,025481173	oxidoreductase activity, acting on CH-OH group of donors
Δhom2	8-day	2666039	up	GO:0016614	0,025481173	oxidoreductase activity, acting on CH-OH group of donors
Δhom2	8-day	2693570	up	GO:0016614	0,025481173	oxidoreductase activity, acting on CH-OH group of donors
Δhom2	8-day	1131542	down	GO:0004497	5,37E-07	monooxygenase activity
Δhom2	8-day	1139445	down	GO:0004497	5,37E-07	monooxygenase activity

Δhom2	8-day	1189794	down	GO:0004497	5,37E-07	monooxygenase activity
Δhom2	8-day	2486680	down	GO:0004497	5,37E-07	monooxygenase activity
Δhom2	8-day	2503471	down	GO:0004497	5,37E-07	monooxygenase activity
Δhom2	8-day	2510618	down	GO:0004497	5,37E-07	monooxygenase activity
Δhom2	8-day	2604231	down	GO:0004497	5,37E-07	monooxygenase activity
Δhom2	8-day	2618455	down	GO:0004497	5,37E-07	monooxygenase activity
Δhom2	8-day	2635487	down	GO:0004497	5,37E-07	monooxygenase activity
Δhom2	8-day	2635869	down	GO:0004497	5,37E-07	monooxygenase activity
Δhom2	8-day	2644430	down	GO:0004497	5,37E-07	monooxygenase activity
Δhom2	8-day	2668568	down	GO:0004497	5,37E-07	monooxygenase activity
Δhom2	8-day	2705258	down	GO:0004497	5,37E-07	monooxygenase activity
Δhom2	8-day	256167	down	GO:0006810	2,38E-06	transport
Δhom2	8-day	1078940	down	GO:0006810	2,38E-06	transport
Δhom2	8-day	1129508	down	GO:0006810	2,38E-06	transport
Δhom2	8-day	1163017	down	GO:0006810	2,38E-06	transport
Δhom2	8-day	1171024	down	GO:0006810	2,38E-06	transport
Δhom2	8-day	1175357	down	GO:0006810	2,38E-06	transport
Δhom2	8-day	1179721	down	GO:0006810	2,38E-06	transport
Δhom2	8-day	1188719	down	GO:0006810	2,38E-06	transport
Δhom2	8-day	2510346	down	GO:0006810	2,38E-06	transport
Δhom2	8-day	2516374	down	GO:0006810	2,38E-06	transport
Δhom2	8-day	2543516	down	GO:0006810	2,38E-06	transport
Δhom2	8-day	2607662	down	GO:0006810	2,38E-06	transport
Δhom2	8-day	2608744	down	GO:0006810	2,38E-06	transport
Δhom2	8-day	2612474	down	GO:0006810	2,38E-06	transport
Δhom2	8-day	2613255	down	GO:0006810	2,38E-06	transport
Δhom2	8-day	2616961	down	GO:0006810	2,38E-06	transport
Δhom2	8-day	2623492	down	GO:0006810	2,38E-06	transport
Δhom2	8-day	2627703	down	GO:0006810	2,38E-06	transport
Δhom2	8-day	2636597	down	GO:0006810	2,38E-06	transport
Δhom2	8-day	2637509	down	GO:0006810	2,38E-06	transport
Δhom2	8-day	2641095	down	GO:0006810	2,38E-06	transport
Δhom2	8-day	2664286	down	GO:0006810	2,38E-06	transport
Δhom2	8-day	2669459	down	GO:0006810	2,38E-06	transport

Δhom2	8-day	2675831	down	GO:0006810	2,38E-06	transport
Δhom2	8-day	2701571	down	GO:0006810	2,38E-06	transport
Δhom2	8-day	2712060	down	GO:0006810	2,38E-06	transport
Δhom2	8-day	1131542	down	GO:0020037	0,000111408	heme binding
Δhom2	8-day	1139445	down	GO:0020037	0,000111408	heme binding
Δhom2	8-day	1189794	down	GO:0020037	0,000111408	heme binding
Δhom2	8-day	2486680	down	GO:0020037	0,000111408	heme binding
Δhom2	8-day	2497674	down	GO:0020037	0,000111408	heme binding
Δhom2	8-day	2510618	down	GO:0020037	0,000111408	heme binding
Δhom2	8-day	2604231	down	GO:0020037	0,000111408	heme binding
Δhom2	8-day	2609854	down	GO:0020037	0,000111408	heme binding
Δhom2	8-day	2635487	down	GO:0020037	0,000111408	heme binding
Δhom2	8-day	2635869	down	GO:0020037	0,000111408	heme binding
Δhom2	8-day	2668568	down	GO:0020037	0,000111408	heme binding
Δhom2	8-day	2705258	down	GO:0020037	0,000111408	heme binding
Δhom2	8-day	1131542	down	GO:0006118	0,000534277	electron transport
Δhom2	8-day	1139445	down	GO:0006118	0,000534277	electron transport
Δhom2	8-day	1189794	down	GO:0006118	0,000534277	electron transport
Δhom2	8-day	2486680	down	GO:0006118	0,000534277	electron transport
Δhom2	8-day	2490518	down	GO:0006118	0,000534277	electron transport
Δhom2	8-day	2497674	down	GO:0006118	0,000534277	electron transport
Δhom2	8-day	2499521	down	GO:0006118	0,000534277	electron transport
Δhom2	8-day	2503471	down	GO:0006118	0,000534277	electron transport
Δhom2	8-day	2510618	down	GO:0006118	0,000534277	electron transport
Δhom2	8-day	2604231	down	GO:0006118	0,000534277	electron transport
Δhom2	8-day	2609831	down	GO:0006118	0,000534277	electron transport
Δhom2	8-day	2609854	down	GO:0006118	0,000534277	electron transport
Δhom2	8-day	2613466	down	GO:0006118	0,000534277	electron transport
Δhom2	8-day	2613481	down	GO:0006118	0,000534277	electron transport
Δhom2	8-day	2618455	down	GO:0006118	0,000534277	electron transport
Δhom2	8-day	2620726	down	GO:0006118	0,000534277	electron transport
Δhom2	8-day	2633167	down	GO:0006118	0,000534277	electron transport
Δhom2	8-day	2635487	down	GO:0006118	0,000534277	electron transport
Δhom2	8-day	2635869	down	GO:0006118	0,000534277	electron transport

Δhom2	8-day	2637104	down	GO:0006118	0,000534277	electron transport
Δhom2	8-day	2639682	down	GO:0006118	0,000534277	electron transport
Δhom2	8-day	2644430	down	GO:0006118	0,000534277	electron transport
Δhom2	8-day	2668568	down	GO:0006118	0,000534277	electron transport
Δhom2	8-day	2705258	down	GO:0006118	0,000534277	electron transport
Δhom2	8-day	256167	down	GO:0005215	0,000650959	transporter activity
Δhom2	8-day	1129508	down	GO:0005215	0,000650959	transporter activity
Δhom2	8-day	1171024	down	GO:0005215	0,000650959	transporter activity
Δhom2	8-day	1175357	down	GO:0005215	0,000650959	transporter activity
Δhom2	8-day	1179721	down	GO:0005215	0,000650959	transporter activity
Δhom2	8-day	1188719	down	GO:0005215	0,000650959	transporter activity
Δhom2	8-day	2510346	down	GO:0005215	0,000650959	transporter activity
Δhom2	8-day	2516374	down	GO:0005215	0,000650959	transporter activity
Δhom2	8-day	2543516	down	GO:0005215	0,000650959	transporter activity
Δhom2	8-day	2607662	down	GO:0005215	0,000650959	transporter activity
Δhom2	8-day	2608744	down	GO:0005215	0,000650959	transporter activity
Δhom2	8-day	2612474	down	GO:0005215	0,000650959	transporter activity
Δhom2	8-day	2613255	down	GO:0005215	0,000650959	transporter activity
Δhom2	8-day	2616961	down	GO:0005215	0,000650959	transporter activity
Δhom2	8-day	2623492	down	GO:0005215	0,000650959	transporter activity
Δhom2	8-day	2627703	down	GO:0005215	0,000650959	transporter activity
Δhom2	8-day	2636597	down	GO:0005215	0,000650959	transporter activity
Δhom2	8-day	2637509	down	GO:0005215	0,000650959	transporter activity
Δhom2	8-day	2641095	down	GO:0005215	0,000650959	transporter activity
Δhom2	8-day	2664286	down	GO:0005215	0,000650959	transporter activity
Δhom2	8-day	2669459	down	GO:0005215	0,000650959	transporter activity
Δhom2	8-day	2701571	down	GO:0005215	0,000650959	transporter activity
Δhom2	8-day	2712060	down	GO:0005215	0,000650959	transporter activity
Δhom2	8-day	1139445	down	GO:0050381	0,001012262	unspecific monooxygenase activity
Δhom2	8-day	2486680	down	GO:0050381	0,001012262	unspecific monooxygenase activity
Δhom2	8-day	2510618	down	GO:0050381	0,001012262	unspecific monooxygenase activity
Δhom2	8-day	2635869	down	GO:0050381	0,001012262	unspecific monooxygenase activity
Δhom2	8-day	2705258	down	GO:0050381	0,001012262	unspecific monooxygenase activity
Δhom2	8-day	1131542	down	GO:0005506	0,001012262	iron ion binding

Δhom2	8-day	1139445	down	GO:0005506	0,001012262	iron ion binding
Δhom2	8-day	1189794	down	GO:0005506	0,001012262	iron ion binding
Δhom2	8-day	2312801	down	GO:0005506	0,001012262	iron ion binding
Δhom2	8-day	2486680	down	GO:0005506	0,001012262	iron ion binding
Δhom2	8-day	2497674	down	GO:0005506	0,001012262	iron ion binding
Δhom2	8-day	2510618	down	GO:0005506	0,001012262	iron ion binding
Δhom2	8-day	2604231	down	GO:0005506	0,001012262	iron ion binding
Δhom2	8-day	2618220	down	GO:0005506	0,001012262	iron ion binding
Δhom2	8-day	2628448	down	GO:0005506	0,001012262	iron ion binding
Δhom2	8-day	2635487	down	GO:0005506	0,001012262	iron ion binding
Δhom2	8-day	2635869	down	GO:0005506	0,001012262	iron ion binding
Δhom2	8-day	2668568	down	GO:0005506	0,001012262	iron ion binding
Δhom2	8-day	2705258	down	GO:0005506	0,001012262	iron ion binding
Δhom2	8-day	2675831	down	GO:0006865	0,002614182	amino acid transport
Δhom2	8-day	2675831	down	GO:0015171	0,002614182	amino acid transmembrane transporter activity
Δhom2	8-day	2592894	down	GO:0016614	0,003051928	oxidoreductase activity, acting on CH-OH group of donors
Δhom2	8-day	2614510	down	GO:0016614	0,003051928	oxidoreductase activity, acting on CH-OH group of donors
Δhom2	8-day	2642438	down	GO:0016614	0,003051928	oxidoreductase activity, acting on CH-OH group of donors
Δhom2	8-day	2642607	down	GO:0016614	0,003051928	oxidoreductase activity, acting on CH-OH group of donors
Δhom2	8-day	2643025	down	GO:0016614	0,003051928	oxidoreductase activity, acting on CH-OH group of donors
Δhom2	8-day	2540746	down	GO:0050660	0,003600851	FAD binding
Δhom2	8-day	2592894	down	GO:0050660	0,003600851	FAD binding
Δhom2	8-day	2614510	down	GO:0050660	0,003600851	FAD binding
Δhom2	8-day	2623663	down	GO:0050660	0,003600851	FAD binding
Δhom2	8-day	2642438	down	GO:0050660	0,003600851	FAD binding
Δhom2	8-day	2642607	down	GO:0050660	0,003600851	FAD binding
Δhom2	8-day	2643025	down	GO:0050660	0,003600851	FAD binding
Δhom2	8-day	2614029	down	GO:0005618	0,004956468	cell wall
Δhom2	8-day	1175357	down	GO:0016020	0,006783157	membrane
Δhom2	8-day	2516374	down	GO:0016020	0,006783157	membrane
Δhom2	8-day	2543516	down	GO:0016020	0,006783157	membrane
Δhom2	8-day	2608744	down	GO:0016020	0,006783157	membrane
Δhom2	8-day	2612474	down	GO:0016020	0,006783157	membrane
Δhom2	8-day	2613255	down	GO:0016020	0,006783157	membrane

Δhom2	8-day	2627703	down	GO:0016020	0,006783157	membrane
Δhom2	8-day	2629358	down	GO:0016020	0,006783157	membrane
Δhom2	8-day	2640999	down	GO:0016020	0,006783157	membrane
Δhom2	8-day	2641095	down	GO:0016020	0,006783157	membrane
Δhom2	8-day	2675646	down	GO:0016020	0,006783157	membrane
Δhom2	8-day	2675831	down	GO:0016020	0,006783157	membrane
Δhom2	8-day	2701571	down	GO:0016020	0,006783157	membrane
Δhom2	8-day	1156481	down	GO:0016491	0,016084515	oxidoreductase activity
Δhom2	8-day	1189573	down	GO:0016491	0,016084515	oxidoreductase activity
Δhom2	8-day	2312801	down	GO:0016491	0,016084515	oxidoreductase activity
Δhom2	8-day	2490518	down	GO:0016491	0,016084515	oxidoreductase activity
Δhom2	8-day	2495855	down	GO:0016491	0,016084515	oxidoreductase activity
Δhom2	8-day	2497674	down	GO:0016491	0,016084515	oxidoreductase activity
Δhom2	8-day	2499521	down	GO:0016491	0,016084515	oxidoreductase activity
Δhom2	8-day	2503471	down	GO:0016491	0,016084515	oxidoreductase activity
Δhom2	8-day	2507164	down	GO:0016491	0,016084515	oxidoreductase activity
Δhom2	8-day	2515739	down	GO:0016491	0,016084515	oxidoreductase activity
Δhom2	8-day	2515964	down	GO:0016491	0,016084515	oxidoreductase activity
Δhom2	8-day	2518082	down	GO:0016491	0,016084515	oxidoreductase activity
Δhom2	8-day	2538948	down	GO:0016491	0,016084515	oxidoreductase activity
Δhom2	8-day	2540746	down	GO:0016491	0,016084515	oxidoreductase activity
Δhom2	8-day	2591516	down	GO:0016491	0,016084515	oxidoreductase activity
Δhom2	8-day	2601704	down	GO:0016491	0,016084515	oxidoreductase activity
Δhom2	8-day	2606428	down	GO:0016491	0,016084515	oxidoreductase activity
Δhom2	8-day	2606988	down	GO:0016491	0,016084515	oxidoreductase activity
Δhom2	8-day	2608048	down	GO:0016491	0,016084515	oxidoreductase activity
Δhom2	8-day	2609854	down	GO:0016491	0,016084515	oxidoreductase activity
Δhom2	8-day	2611538	down	GO:0016491	0,016084515	oxidoreductase activity
Δhom2	8-day	2613466	down	GO:0016491	0,016084515	oxidoreductase activity
Δhom2	8-day	2613481	down	GO:0016491	0,016084515	oxidoreductase activity
Δhom2	8-day	2615160	down	GO:0016491	0,016084515	oxidoreductase activity
Δhom2	8-day	2616880	down	GO:0016491	0,016084515	oxidoreductase activity
Δhom2	8-day	2618220	down	GO:0016491	0,016084515	oxidoreductase activity
Δhom2	8-day	2618455	down	GO:0016491	0,016084515	oxidoreductase activity

Δhom2	8-day	2621873	down	GO:0016491	0,016084515	oxidoreductase activity
Δhom2	8-day	2623663	down	GO:0016491	0,016084515	oxidoreductase activity
Δhom2	8-day	2625917	down	GO:0016491	0,016084515	oxidoreductase activity
Δhom2	8-day	2629507	down	GO:0016491	0,016084515	oxidoreductase activity
Δhom2	8-day	2629526	down	GO:0016491	0,016084515	oxidoreductase activity
Δhom2	8-day	2633167	down	GO:0016491	0,016084515	oxidoreductase activity
Δhom2	8-day	2634450	down	GO:0016491	0,016084515	oxidoreductase activity
Δhom2	8-day	2637104	down	GO:0016491	0,016084515	oxidoreductase activity
Δhom2	8-day	2637755	down	GO:0016491	0,016084515	oxidoreductase activity
Δhom2	8-day	2639682	down	GO:0016491	0,016084515	oxidoreductase activity
Δhom2	8-day	2641506	down	GO:0016491	0,016084515	oxidoreductase activity
Δhom2	8-day	2642438	down	GO:0016491	0,016084515	oxidoreductase activity
Δhom2	8-day	2644430	down	GO:0016491	0,016084515	oxidoreductase activity
Δhom2	8-day	2685505	down	GO:0016491	0,016084515	oxidoreductase activity
Δhom2	8-day	2614510	down	GO:0006066	0,019346511	alcohol metabolic process
Δhom2	8-day	2642607	down	GO:0006066	0,019346511	alcohol metabolic process
Δhom2	8-day	2643025	down	GO:0006066	0,019346511	alcohol metabolic process
Δhom2	8-day	2614510	down	GO:0008812	0,019346511	choline dehydrogenase activity
Δhom2	8-day	2642607	down	GO:0008812	0,019346511	choline dehydrogenase activity
Δhom2	8-day	2643025	down	GO:0008812	0,019346511	choline dehydrogenase activity
Δwc1	8-day	2643740	up	GO:0004553	0,000587315	hydrolase activity, hydrolyzing O-glycosyl compounds
Δwc1	8-day	2607659	up	GO:0008733	0,000587315	L-arabinose isomerase activity
Δwc1	8-day	2514546	up	GO:0005975	0,000587315	carbohydrate metabolic process
Δwc1	8-day	2609013	up	GO:0005975	0,000587315	carbohydrate metabolic process
Δwc1	8-day	2620185	up	GO:0005975	0,000587315	carbohydrate metabolic process
Δwc1	8-day	2643740	up	GO:0005975	0,000587315	carbohydrate metabolic process
Δwc1	8-day	2645945	up	GO:0005975	0,000587315	carbohydrate metabolic process
Δwc1	8-day	2607659	up	GO:0008643	0,000587315	carbohydrate transport
Δwc1	8-day	2607659	up	GO:0005351	0,000785667	sugar:hydrogen symporter activity
Δwc1	8-day	1120318	up	GO:0005506	0,000785667	iron ion binding
Δwc1	8-day	2364606	up	GO:0005506	0,000785667	iron ion binding
Δwc1	8-day	2491624	up	GO:0005506	0,000785667	iron ion binding
Δwc1	8-day	2501258	up	GO:0005506	0,000785667	iron ion binding
Δwc1	8-day	2616590	up	GO:0005506	0,000785667	iron ion binding

Δwc1	8-day	2617060	up	GO:0005506	0,000785667	iron ion binding
Δwc1	8-day	2623122	up	GO:0005506	0,000785667	iron ion binding
Δwc1	8-day	2633776	up	GO:0005506	0,000785667	iron ion binding
Δwc1	8-day	2635870	up	GO:0005506	0,000785667	iron ion binding
Δwc1	8-day	2666440	up	GO:0005506	0,000785667	iron ion binding
Δwc1	8-day	2753029	up	GO:0005506	0,000785667	iron ion binding
Δwc1	8-day	1120318	up	GO:0006118	0,002748685	electron transport
Δwc1	8-day	1215660	up	GO:0006118	0,002748685	electron transport
Δwc1	8-day	1342670	up	GO:0006118	0,002748685	electron transport
Δwc1	8-day	2364606	up	GO:0006118	0,002748685	electron transport
Δwc1	8-day	2490194	up	GO:0006118	0,002748685	electron transport
Δwc1	8-day	2491624	up	GO:0006118	0,002748685	electron transport
Δwc1	8-day	2501258	up	GO:0006118	0,002748685	electron transport
Δwc1	8-day	2573393	up	GO:0006118	0,002748685	electron transport
Δwc1	8-day	2573396	up	GO:0006118	0,002748685	electron transport
Δwc1	8-day	2613444	up	GO:0006118	0,002748685	electron transport
Δwc1	8-day	2616557	up	GO:0006118	0,002748685	electron transport
Δwc1	8-day	2616590	up	GO:0006118	0,002748685	electron transport
Δwc1	8-day	2623122	up	GO:0006118	0,002748685	electron transport
Δwc1	8-day	2625730	up	GO:0006118	0,002748685	electron transport
Δwc1	8-day	2633776	up	GO:0006118	0,002748685	electron transport
Δwc1	8-day	2635870	up	GO:0006118	0,002748685	electron transport
Δwc1	8-day	2666440	up	GO:0006118	0,002748685	electron transport
Δwc1	8-day	2671376	up	GO:0006118	0,002748685	electron transport
Δwc1	8-day	2703927	up	GO:0006118	0,002748685	electron transport
Δwc1	8-day	2753029	up	GO:0006118	0,002748685	electron transport
Δwc1	8-day	2638753	up	GO:0016614	0,002748685	oxidoreductase activity, acting on CH-OH group of donors
Δwc1	8-day	2643062	up	GO:0016614	0,002748685	oxidoreductase activity, acting on CH-OH group of donors
Δwc1	8-day	2663917	up	GO:0016614	0,002748685	oxidoreductase activity, acting on CH-OH group of donors
Δwc1	8-day	2693570	up	GO:0016614	0,002748685	oxidoreductase activity, acting on CH-OH group of donors
Δwc1	8-day	2643062	up	GO:0006066	0,003509835	alcohol metabolic process
Δwc1	8-day	2693570	up	GO:0006066	0,003509835	alcohol metabolic process
Δwc1	8-day	2643062	up	GO:0008812	0,003509835	choline dehydrogenase activity
Δwc1	8-day	2693570	up	GO:0008812	0,003509835	choline dehydrogenase activity

Δwc1	8-day	1120318	up	GO:0050381	0,003882954	unspecific monooxygenase activity
Δwc1	8-day	2491624	up	GO:0050381	0,003882954	unspecific monooxygenase activity
Δwc1	8-day	2633776	up	GO:0050381	0,003882954	unspecific monooxygenase activity
Δwc1	8-day	2666440	up	GO:0050381	0,003882954	unspecific monooxygenase activity
Δwc1	8-day	2753029	up	GO:0050381	0,003882954	unspecific monooxygenase activity
Δwc1	8-day	1120318	up	GO:0004497	0,007651462	monooxygenase activity
Δwc1	8-day	1342670	up	GO:0004497	0,007651462	monooxygenase activity
Δwc1	8-day	2364606	up	GO:0004497	0,007651462	monooxygenase activity
Δwc1	8-day	2490194	up	GO:0004497	0,007651462	monooxygenase activity
Δwc1	8-day	2491624	up	GO:0004497	0,007651462	monooxygenase activity
Δwc1	8-day	2501258	up	GO:0004497	0,007651462	monooxygenase activity
Δwc1	8-day	2573393	up	GO:0004497	0,007651462	monooxygenase activity
Δwc1	8-day	2573396	up	GO:0004497	0,007651462	monooxygenase activity
Δwc1	8-day	2616590	up	GO:0004497	0,007651462	monooxygenase activity
Δwc1	8-day	2623122	up	GO:0004497	0,007651462	monooxygenase activity
Δwc1	8-day	2633776	up	GO:0004497	0,007651462	monooxygenase activity
Δwc1	8-day	2635870	up	GO:0004497	0,007651462	monooxygenase activity
Δwc1	8-day	2666440	up	GO:0004497	0,007651462	monooxygenase activity
Δwc1	8-day	2703927	up	GO:0004497	0,007651462	monooxygenase activity
Δwc1	8-day	2753029	up	GO:0004497	0,007651462	monooxygenase activity
Δwc1	8-day	1194451	up	GO:0016491	0,007795772	oxidoreductase activity
Δwc1	8-day	1342670	up	GO:0016491	0,007795772	oxidoreductase activity
Δwc1	8-day	2487936	up	GO:0016491	0,007795772	oxidoreductase activity
Δwc1	8-day	2490194	up	GO:0016491	0,007795772	oxidoreductase activity
Δwc1	8-day	2501673	up	GO:0016491	0,007795772	oxidoreductase activity
Δwc1	8-day	2506447	up	GO:0016491	0,007795772	oxidoreductase activity
Δwc1	8-day	2506612	up	GO:0016491	0,007795772	oxidoreductase activity
Δwc1	8-day	2524219	up	GO:0016491	0,007795772	oxidoreductase activity
Δwc1	8-day	2573393	up	GO:0016491	0,007795772	oxidoreductase activity
Δwc1	8-day	2573396	up	GO:0016491	0,007795772	oxidoreductase activity
Δwc1	8-day	2606041	up	GO:0016491	0,007795772	oxidoreductase activity
Δwc1	8-day	2611263	up	GO:0016491	0,007795772	oxidoreductase activity
Δwc1	8-day	2613444	up	GO:0016491	0,007795772	oxidoreductase activity
Δwc1	8-day	2614455	up	GO:0016491	0,007795772	oxidoreductase activity

Δwc1	8-day	2616557	up	GO:0016491	0,007795772	oxidoreductase activity
Δwc1	8-day	2617060	up	GO:0016491	0,007795772	oxidoreductase activity
Δwc1	8-day	2623957	up	GO:0016491	0,007795772	oxidoreductase activity
Δwc1	8-day	2625836	up	GO:0016491	0,007795772	oxidoreductase activity
Δwc1	8-day	2630033	up	GO:0016491	0,007795772	oxidoreductase activity
Δwc1	8-day	2632193	up	GO:0016491	0,007795772	oxidoreductase activity
Δwc1	8-day	2634619	up	GO:0016491	0,007795772	oxidoreductase activity
Δwc1	8-day	2636220	up	GO:0016491	0,007795772	oxidoreductase activity
Δwc1	8-day	2636221	up	GO:0016491	0,007795772	oxidoreductase activity
Δwc1	8-day	2638445	up	GO:0016491	0,007795772	oxidoreductase activity
Δwc1	8-day	2638753	up	GO:0016491	0,007795772	oxidoreductase activity
Δwc1	8-day	2640123	up	GO:0016491	0,007795772	oxidoreductase activity
Δwc1	8-day	2671376	up	GO:0016491	0,007795772	oxidoreductase activity
Δwc1	8-day	2681649	up	GO:0016491	0,007795772	oxidoreductase activity
Δwc1	8-day	2691273	up	GO:0016491	0,007795772	oxidoreductase activity
Δwc1	8-day	2703927	up	GO:0016491	0,007795772	oxidoreductase activity
Δwc1	8-day	2713107	up	GO:0016491	0,007795772	oxidoreductase activity
Δwc1	8-day	2748659	up	GO:0016491	0,007795772	oxidoreductase activity
Δwc1	8-day	2508421	up	GO:0005215	0,007795772	transporter activity
Δwc1	8-day	2607659	up	GO:0005215	0,007795772	transporter activity
Δwc1	8-day	2616961	up	GO:0005215	0,007795772	transporter activity
Δwc1	8-day	2617636	up	GO:0005215	0,007795772	transporter activity
Δwc1	8-day	2618842	up	GO:0005215	0,007795772	transporter activity
Δwc1	8-day	2620170	up	GO:0005215	0,007795772	transporter activity
Δwc1	8-day	2622931	up	GO:0005215	0,007795772	transporter activity
Δwc1	8-day	2628098	up	GO:0005215	0,007795772	transporter activity
Δwc1	8-day	2628982	up	GO:0005215	0,007795772	transporter activity
Δwc1	8-day	2631348	up	GO:0005215	0,007795772	transporter activity
Δwc1	8-day	2632105	up	GO:0005215	0,007795772	transporter activity
Δwc1	8-day	2637652	up	GO:0005215	0,007795772	transporter activity
Δwc1	8-day	1120318	up	GO:0020037	0,009216473	heme binding
Δwc1	8-day	2364606	up	GO:0020037	0,009216473	heme binding
Δwc1	8-day	2491624	up	GO:0020037	0,009216473	heme binding
Δwc1	8-day	2501258	up	GO:0020037	0,009216473	heme binding

Δwc1	8-day	2608091	up	GO:0020037	0,009216473	heme binding
Δwc1	8-day	2616590	up	GO:0020037	0,009216473	heme binding
Δwc1	8-day	2623122	up	GO:0020037	0,009216473	heme binding
Δwc1	8-day	2633776	up	GO:0020037	0,009216473	heme binding
Δwc1	8-day	2635870	up	GO:0020037	0,009216473	heme binding
Δwc1	8-day	2666440	up	GO:0020037	0,009216473	heme binding
Δwc1	8-day	2673121	up	GO:0020037	0,009216473	heme binding
Δwc1	8-day	2753029	up	GO:0020037	0,009216473	heme binding
Δwc1	8-day	2620185	up	GO:0016810	0,012144163	hydrolase activity, acting on carbon-nitrogen (but not peptide) bonds
Δwc1	8-day	2633738	up	GO:0016810	0,012144163	hydrolase activity, acting on carbon-nitrogen (but not peptide) bonds
Δwc1	8-day	48473	up	GO:0003824	0,013358637	catalytic activity
Δwc1	8-day	2368049	up	GO:0003824	0,013358637	catalytic activity
Δwc1	8-day	2502008	up	GO:0003824	0,013358637	catalytic activity
Δwc1	8-day	2506635	up	GO:0003824	0,013358637	catalytic activity
Δwc1	8-day	2514546	up	GO:0003824	0,013358637	catalytic activity
Δwc1	8-day	2594114	up	GO:0003824	0,013358637	catalytic activity
Δwc1	8-day	2606333	up	GO:0003824	0,013358637	catalytic activity
Δwc1	8-day	2614455	up	GO:0003824	0,013358637	catalytic activity
Δwc1	8-day	2616512	up	GO:0003824	0,013358637	catalytic activity
Δwc1	8-day	2620185	up	GO:0003824	0,013358637	catalytic activity
Δwc1	8-day	2621626	up	GO:0003824	0,013358637	catalytic activity
Δwc1	8-day	2623957	up	GO:0003824	0,013358637	catalytic activity
Δwc1	8-day	2626172	up	GO:0003824	0,013358637	catalytic activity
Δwc1	8-day	2626529	up	GO:0003824	0,013358637	catalytic activity
Δwc1	8-day	2631920	up	GO:0003824	0,013358637	catalytic activity
Δwc1	8-day	2632193	up	GO:0003824	0,013358637	catalytic activity
Δwc1	8-day	2636220	up	GO:0003824	0,013358637	catalytic activity
Δwc1	8-day	2636221	up	GO:0003824	0,013358637	catalytic activity
Δwc1	8-day	2637388	up	GO:0003824	0,013358637	catalytic activity
Δwc1	8-day	2638445	up	GO:0003824	0,013358637	catalytic activity
Δwc1	8-day	2638446	up	GO:0003824	0,013358637	catalytic activity
Δwc1	8-day	2640123	up	GO:0003824	0,013358637	catalytic activity

Δwc1	8-day	2681649	up	GO:0003824	0,013358637	catalytic activity
Δwc1	8-day	2682272	up	GO:0003824	0,013358637	catalytic activity
Δwc1	8-day	2748659	up	GO:0003824	0,013358637	catalytic activity
Δwc1	8-day	1174869	up	GO:0005524	0,018073482	ATP binding
Δwc1	8-day	2279823	up	GO:0005524	0,018073482	ATP binding
Δwc1	8-day	2621626	up	GO:0005524	0,018073482	ATP binding
Δwc1	8-day	2623809	up	GO:0005524	0,018073482	ATP binding
Δwc1	8-day	2645675	up	GO:0005524	0,018073482	ATP binding
Δwc1	8-day	2675859	up	GO:0005524	0,018073482	ATP binding
Δwc1	8-day	2693043	up	GO:0005524	0,018073482	ATP binding
Δwc1	8-day	2693940	up	GO:0005524	0,018073482	ATP binding
Δwc1	8-day	2727986	up	GO:0005524	0,018073482	ATP binding
Δwc1	8-day	2638753	up	GO:0050660	0,027550831	FAD binding
Δwc1	8-day	2640123	up	GO:0050660	0,027550831	FAD binding
Δwc1	8-day	2643062	up	GO:0050660	0,027550831	FAD binding
Δwc1	8-day	2663917	up	GO:0050660	0,027550831	FAD binding
Δwc1	8-day	2693570	up	GO:0050660	0,027550831	FAD binding
Δwc1	8-day	2748659	up	GO:0050660	0,027550831	FAD binding
Δwc1	8-day	2560780	up	GO:0030170	0,027550831	pyridoxal phosphate binding
Δwc1	8-day	2606037	up	GO:0030170	0,027550831	pyridoxal phosphate binding
Δwc1	8-day	2632714	up	GO:0030170	0,027550831	pyridoxal phosphate binding
Δwc1	8-day	1189573	down	GO:0016491	0,00694302	oxidoreductase activity
Δwc1	8-day	2312801	down	GO:0016491	0,00694302	oxidoreductase activity
Δwc1	8-day	2483752	down	GO:0016491	0,00694302	oxidoreductase activity
Δwc1	8-day	2490518	down	GO:0016491	0,00694302	oxidoreductase activity
Δwc1	8-day	2497674	down	GO:0016491	0,00694302	oxidoreductase activity
Δwc1	8-day	2499521	down	GO:0016491	0,00694302	oxidoreductase activity
Δwc1	8-day	2507164	down	GO:0016491	0,00694302	oxidoreductase activity
Δwc1	8-day	2515739	down	GO:0016491	0,00694302	oxidoreductase activity
Δwc1	8-day	2516444	down	GO:0016491	0,00694302	oxidoreductase activity
Δwc1	8-day	2571870	down	GO:0016491	0,00694302	oxidoreductase activity
Δwc1	8-day	2591516	down	GO:0016491	0,00694302	oxidoreductase activity
Δwc1	8-day	2606988	down	GO:0016491	0,00694302	oxidoreductase activity
Δwc1	8-day	2608048	down	GO:0016491	0,00694302	oxidoreductase activity

Δwc1	8-day	2611538	down	GO:0016491	0,00694302	oxidoreductase activity
Δwc1	8-day	2615160	down	GO:0016491	0,00694302	oxidoreductase activity
Δwc1	8-day	2616880	down	GO:0016491	0,00694302	oxidoreductase activity
Δwc1	8-day	2616916	down	GO:0016491	0,00694302	oxidoreductase activity
Δwc1	8-day	2618220	down	GO:0016491	0,00694302	oxidoreductase activity
Δwc1	8-day	2623663	down	GO:0016491	0,00694302	oxidoreductase activity
Δwc1	8-day	2625917	down	GO:0016491	0,00694302	oxidoreductase activity
Δwc1	8-day	2629507	down	GO:0016491	0,00694302	oxidoreductase activity
Δwc1	8-day	2629850	down	GO:0016491	0,00694302	oxidoreductase activity
Δwc1	8-day	2630590	down	GO:0016491	0,00694302	oxidoreductase activity
Δwc1	8-day	2633167	down	GO:0016491	0,00694302	oxidoreductase activity
Δwc1	8-day	2637755	down	GO:0016491	0,00694302	oxidoreductase activity
Δwc1	8-day	2639682	down	GO:0016491	0,00694302	oxidoreductase activity
Δwc1	8-day	2641520	down	GO:0016491	0,00694302	oxidoreductase activity
Δwc1	8-day	2661224	down	GO:0016491	0,00694302	oxidoreductase activity
Δwc1	8-day	2705421	down	GO:0016491	0,00694302	oxidoreductase activity
Δwc1	8-day	1131542	down	GO:0004497	0,00694302	monooxygenase activity
Δwc1	8-day	1193647	down	GO:0004497	0,00694302	monooxygenase activity
Δwc1	8-day	2616301	down	GO:0004497	0,00694302	monooxygenase activity
Δwc1	8-day	2620407	down	GO:0004497	0,00694302	monooxygenase activity
Δwc1	8-day	2629864	down	GO:0004497	0,00694302	monooxygenase activity
Δwc1	8-day	2632925	down	GO:0004497	0,00694302	monooxygenase activity
Δwc1	8-day	2635487	down	GO:0004497	0,00694302	monooxygenase activity
Δwc1	8-day	2636544	down	GO:0004497	0,00694302	monooxygenase activity
Δwc1	8-day	2638355	down	GO:0004497	0,00694302	monooxygenase activity
Δwc1	8-day	2668568	down	GO:0004497	0,00694302	monooxygenase activity
Δwc1	8-day	2703966	down	GO:0004497	0,00694302	monooxygenase activity
Δwc1	8-day	2705258	down	GO:0004497	0,00694302	monooxygenase activity
Δwc1	8-day	2735179	down	GO:0004497	0,00694302	monooxygenase activity
Δwc1	8-day	2675831	down	GO:0015171	0,009265003	amino acid transmembrane transporter activity
Δwc1	8-day	2675831	down	GO:0006865	0,009265003	amino acid transport
Δwc1	8-day	2629603	down	GO:0005199	0,018915599	structural constituent of cell wall
Δwc1	8-day	2629603	down	GO:0005618	0,021849425	cell wall
Δwc1	8-day	2577992	down	GO:0004194	0,023576008	pepsin A activity

Δwc1	8-day	68300	down	GO:0006810	0,023576008	transport
Δwc1	8-day	2283957	down	GO:0006810	0,023576008	transport
Δwc1	8-day	2515031	down	GO:0006810	0,023576008	transport
Δwc1	8-day	2516374	down	GO:0006810	0,023576008	transport
Δwc1	8-day	2543516	down	GO:0006810	0,023576008	transport
Δwc1	8-day	2550918	down	GO:0006810	0,023576008	transport
Δwc1	8-day	2611816	down	GO:0006810	0,023576008	transport
Δwc1	8-day	2613255	down	GO:0006810	0,023576008	transport
Δwc1	8-day	2617729	down	GO:0006810	0,023576008	transport
Δwc1	8-day	2623492	down	GO:0006810	0,023576008	transport
Δwc1	8-day	2627703	down	GO:0006810	0,023576008	transport
Δwc1	8-day	2636597	down	GO:0006810	0,023576008	transport
Δwc1	8-day	2661224	down	GO:0006810	0,023576008	transport
Δwc1	8-day	2669459	down	GO:0006810	0,023576008	transport
Δwc1	8-day	2675831	down	GO:0006810	0,023576008	transport
Δwc1	8-day	2701571	down	GO:0006810	0,023576008	transport
Δwc1	8-day	2712060	down	GO:0006810	0,023576008	transport
Δwc1	8-day	1131542	down	GO:0006118	0,028913489	electron transport
Δwc1	8-day	1193647	down	GO:0006118	0,028913489	electron transport
Δwc1	8-day	2490518	down	GO:0006118	0,028913489	electron transport
Δwc1	8-day	2497674	down	GO:0006118	0,028913489	electron transport
Δwc1	8-day	2499521	down	GO:0006118	0,028913489	electron transport
Δwc1	8-day	2507628	down	GO:0006118	0,028913489	electron transport
Δwc1	8-day	2571870	down	GO:0006118	0,028913489	electron transport
Δwc1	8-day	2616301	down	GO:0006118	0,028913489	electron transport
Δwc1	8-day	2620407	down	GO:0006118	0,028913489	electron transport
Δwc1	8-day	2620726	down	GO:0006118	0,028913489	electron transport
Δwc1	8-day	2629850	down	GO:0006118	0,028913489	electron transport
Δwc1	8-day	2629864	down	GO:0006118	0,028913489	electron transport
Δwc1	8-day	2632925	down	GO:0006118	0,028913489	electron transport
Δwc1	8-day	2633167	down	GO:0006118	0,028913489	electron transport
Δwc1	8-day	2635487	down	GO:0006118	0,028913489	electron transport
Δwc1	8-day	2636544	down	GO:0006118	0,028913489	electron transport
Δwc1	8-day	2638355	down	GO:0006118	0,028913489	electron transport

Δwc1	8-day	2639682	down	GO:0006118	0,028913489	electron transport
Δwc1	8-day	2668568	down	GO:0006118	0,028913489	electron transport
Δwc1	8-day	2703966	down	GO:0006118	0,028913489	electron transport
Δwc1	8-day	2705258	down	GO:0006118	0,028913489	electron transport
Δwc1	8-day	2735179	down	GO:0006118	0,028913489	electron transport
Δwc2	8-day	66483	up	GO:0005975	1,23E-07	carbohydrate metabolic process
Δwc2	8-day	85210	up	GO:0005975	1,23E-07	carbohydrate metabolic process
Δwc2	8-day	2493241	up	GO:0005975	1,23E-07	carbohydrate metabolic process
Δwc2	8-day	2514546	up	GO:0005975	1,23E-07	carbohydrate metabolic process
Δwc2	8-day	2607718	up	GO:0005975	1,23E-07	carbohydrate metabolic process
Δwc2	8-day	2609013	up	GO:0005975	1,23E-07	carbohydrate metabolic process
Δwc2	8-day	2620185	up	GO:0005975	1,23E-07	carbohydrate metabolic process
Δwc2	8-day	2633791	up	GO:0005975	1,23E-07	carbohydrate metabolic process
Δwc2	8-day	2642958	up	GO:0005975	1,23E-07	carbohydrate metabolic process
Δwc2	8-day	2643740	up	GO:0005975	1,23E-07	carbohydrate metabolic process
Δwc2	8-day	2645945	up	GO:0005975	1,23E-07	carbohydrate metabolic process
Δwc2	8-day	2704222	up	GO:0005975	1,23E-07	carbohydrate metabolic process
Δwc2	8-day	2706622	up	GO:0005975	1,23E-07	carbohydrate metabolic process
Δwc2	8-day	85210	up	GO:0004553	2,82E-07	hydrolase activity, hydrolyzing O-glycosyl compounds
Δwc2	8-day	2642958	up	GO:0004553	2,82E-07	hydrolase activity, hydrolyzing O-glycosyl compounds
Δwc2	8-day	2643740	up	GO:0004553	2,82E-07	hydrolase activity, hydrolyzing O-glycosyl compounds
Δwc2	8-day	2704222	up	GO:0004553	2,82E-07	hydrolase activity, hydrolyzing O-glycosyl compounds
Δwc2	8-day	2706622	up	GO:0004553	2,82E-07	hydrolase activity, hydrolyzing O-glycosyl compounds
Δwc2	8-day	1120318	up	GO:0005506	1,41E-05	iron ion binding
Δwc2	8-day	2364606	up	GO:0005506	1,41E-05	iron ion binding
Δwc2	8-day	2491624	up	GO:0005506	1,41E-05	iron ion binding
Δwc2	8-day	2501258	up	GO:0005506	1,41E-05	iron ion binding
Δwc2	8-day	2604628	up	GO:0005506	1,41E-05	iron ion binding
Δwc2	8-day	2616590	up	GO:0005506	1,41E-05	iron ion binding
Δwc2	8-day	2617060	up	GO:0005506	1,41E-05	iron ion binding
Δwc2	8-day	2623189	up	GO:0005506	1,41E-05	iron ion binding
Δwc2	8-day	2635870	up	GO:0005506	1,41E-05	iron ion binding
Δwc2	8-day	2637467	up	GO:0005506	1,41E-05	iron ion binding
Δwc2	8-day	2638282	up	GO:0005506	1,41E-05	iron ion binding

Δwc2	8-day	2666440	up	GO:0005506	1,41E-05	iron ion binding
Δwc2	8-day	2753029	up	GO:0005506	1,41E-05	iron ion binding
Δwc2	8-day	1120318	up	GO:0004497	0,000384306	monooxygenase activity
Δwc2	8-day	1342670	up	GO:0004497	0,000384306	monooxygenase activity
Δwc2	8-day	2364606	up	GO:0004497	0,000384306	monooxygenase activity
Δwc2	8-day	2490194	up	GO:0004497	0,000384306	monooxygenase activity
Δwc2	8-day	2491624	up	GO:0004497	0,000384306	monooxygenase activity
Δwc2	8-day	2501258	up	GO:0004497	0,000384306	monooxygenase activity
Δwc2	8-day	2557571	up	GO:0004497	0,000384306	monooxygenase activity
Δwc2	8-day	2573393	up	GO:0004497	0,000384306	monooxygenase activity
Δwc2	8-day	2573396	up	GO:0004497	0,000384306	monooxygenase activity
Δwc2	8-day	2616590	up	GO:0004497	0,000384306	monooxygenase activity
Δwc2	8-day	2635870	up	GO:0004497	0,000384306	monooxygenase activity
Δwc2	8-day	2637467	up	GO:0004497	0,000384306	monooxygenase activity
Δwc2	8-day	2638282	up	GO:0004497	0,000384306	monooxygenase activity
Δwc2	8-day	2666440	up	GO:0004497	0,000384306	monooxygenase activity
Δwc2	8-day	2703927	up	GO:0004497	0,000384306	monooxygenase activity
Δwc2	8-day	2753029	up	GO:0004497	0,000384306	monooxygenase activity
Δwc2	8-day	1120318	up	GO:0020037	0,000529648	heme binding
Δwc2	8-day	2364606	up	GO:0020037	0,000529648	heme binding
Δwc2	8-day	2491624	up	GO:0020037	0,000529648	heme binding
Δwc2	8-day	2501258	up	GO:0020037	0,000529648	heme binding
Δwc2	8-day	2604628	up	GO:0020037	0,000529648	heme binding
Δwc2	8-day	2608091	up	GO:0020037	0,000529648	heme binding
Δwc2	8-day	2616590	up	GO:0020037	0,000529648	heme binding
Δwc2	8-day	2635870	up	GO:0020037	0,000529648	heme binding
Δwc2	8-day	2637467	up	GO:0020037	0,000529648	heme binding
Δwc2	8-day	2638282	up	GO:0020037	0,000529648	heme binding
Δwc2	8-day	2666440	up	GO:0020037	0,000529648	heme binding
Δwc2	8-day	2673121	up	GO:0020037	0,000529648	heme binding
Δwc2	8-day	2753029	up	GO:0020037	0,000529648	heme binding
Δwc2	8-day	1120318	up	GO:0050381	0,000529648	unspecific monooxygenase activity
Δwc2	8-day	2491624	up	GO:0050381	0,000529648	unspecific monooxygenase activity
Δwc2	8-day	2637467	up	GO:0050381	0,000529648	unspecific monooxygenase activity

Δwc2	8-day	2666440	up	GO:0050381	0,000529648	unspecific monooxygenase activity
Δwc2	8-day	2753029	up	GO:0050381	0,000529648	unspecific monooxygenase activity
Δwc2	8-day	2638753	up	GO:0016614	0,001502273	oxidoreductase activity, acting on CH-OH group of donors
Δwc2	8-day	2643062	up	GO:0016614	0,001502273	oxidoreductase activity, acting on CH-OH group of donors
Δwc2	8-day	2663917	up	GO:0016614	0,001502273	oxidoreductase activity, acting on CH-OH group of donors
Δwc2	8-day	2693570	up	GO:0016614	0,001502273	oxidoreductase activity, acting on CH-OH group of donors
Δwc2	8-day	2643062	up	GO:0008812	0,002417551	choline dehydrogenase activity
Δwc2	8-day	2693570	up	GO:0008812	0,002417551	choline dehydrogenase activity
Δwc2	8-day	2643062	up	GO:0006066	0,002417551	alcohol metabolic process
Δwc2	8-day	2693570	up	GO:0006066	0,002417551	alcohol metabolic process
Δwc2	8-day	1120318	up	GO:0006118	0,004482871	electron transport
Δwc2	8-day	1215660	up	GO:0006118	0,004482871	electron transport
Δwc2	8-day	1342670	up	GO:0006118	0,004482871	electron transport
Δwc2	8-day	2364606	up	GO:0006118	0,004482871	electron transport
Δwc2	8-day	2490194	up	GO:0006118	0,004482871	electron transport
Δwc2	8-day	2491624	up	GO:0006118	0,004482871	electron transport
Δwc2	8-day	2501258	up	GO:0006118	0,004482871	electron transport
Δwc2	8-day	2557571	up	GO:0006118	0,004482871	electron transport
Δwc2	8-day	2573393	up	GO:0006118	0,004482871	electron transport
Δwc2	8-day	2573396	up	GO:0006118	0,004482871	electron transport
Δwc2	8-day	2604628	up	GO:0006118	0,004482871	electron transport
Δwc2	8-day	2605889	up	GO:0006118	0,004482871	electron transport
Δwc2	8-day	2616557	up	GO:0006118	0,004482871	electron transport
Δwc2	8-day	2616590	up	GO:0006118	0,004482871	electron transport
Δwc2	8-day	2625730	up	GO:0006118	0,004482871	electron transport
Δwc2	8-day	2635870	up	GO:0006118	0,004482871	electron transport
Δwc2	8-day	2637467	up	GO:0006118	0,004482871	electron transport
Δwc2	8-day	2638282	up	GO:0006118	0,004482871	electron transport
Δwc2	8-day	2666440	up	GO:0006118	0,004482871	electron transport
Δwc2	8-day	2671376	up	GO:0006118	0,004482871	electron transport
Δwc2	8-day	2703927	up	GO:0006118	0,004482871	electron transport
Δwc2	8-day	2753029	up	GO:0006118	0,004482871	electron transport
Δwc2	8-day	2607659	up	GO:0008643	0,006556977	carbohydrate transport
Δwc2	8-day	2641095	up	GO:0008643	0,006556977	carbohydrate transport

Δwc2	8-day	2607659	up	GO:0008733	0,008412206	L-arabinose isomerase activity
Δwc2	8-day	2641095	up	GO:0008733	0,008412206	L-arabinose isomerase activity
Δwc2	8-day	2607659	up	GO:0005351	0,01236846	sugar:hydrogen symporter activity
Δwc2	8-day	2641095	up	GO:0005351	0,01236846	sugar:hydrogen symporter activity
Δwc2	8-day	2638753	up	GO:0050660	0,018381024	FAD binding
Δwc2	8-day	2640123	up	GO:0050660	0,018381024	FAD binding
Δwc2	8-day	2643062	up	GO:0050660	0,018381024	FAD binding
Δwc2	8-day	2663917	up	GO:0050660	0,018381024	FAD binding
Δwc2	8-day	2693570	up	GO:0050660	0,018381024	FAD binding
Δwc2	8-day	2748659	up	GO:0050660	0,018381024	FAD binding
Δwc2	8-day	2279823	up	GO:0005524	0,018381024	ATP binding
Δwc2	8-day	2529670	up	GO:0005524	0,018381024	ATP binding
Δwc2	8-day	2623809	up	GO:0005524	0,018381024	ATP binding
Δwc2	8-day	2645675	up	GO:0005524	0,018381024	ATP binding
Δwc2	8-day	2675859	up	GO:0005524	0,018381024	ATP binding
Δwc2	8-day	2693043	up	GO:0005524	0,018381024	ATP binding
Δwc2	8-day	2693940	up	GO:0005524	0,018381024	ATP binding
Δwc2	8-day	2727986	up	GO:0005524	0,018381024	ATP binding
Δwc2	8-day	2643740	up	GO:0006032	0,022255079	chitin catabolic process
Δwc2	8-day	2706622	up	GO:0006032	0,022255079	chitin catabolic process
Δwc2	8-day	2632766	up	GO:0004197	0,0334914	cysteine-type endopeptidase activity
Δwc2	8-day	85210	up	GO:0004568	0,04192666	chitinase activity
Δwc2	8-day	2643740	up	GO:0004568	0,04192666	chitinase activity
Δwc2	8-day	2706622	up	GO:0004568	0,04192666	chitinase activity
Δwc2	8-day	85210	up	GO:0008843	0,04192666	endochitinase activity
Δwc2	8-day	2643740	up	GO:0008843	0,04192666	endochitinase activity
Δwc2	8-day	2706622	up	GO:0008843	0,04192666	endochitinase activity
Δwc2	8-day	1189573	down	GO:0016491	1,71E-05	oxidoreductase activity
Δwc2	8-day	2312801	down	GO:0016491	1,71E-05	oxidoreductase activity
Δwc2	8-day	2483752	down	GO:0016491	1,71E-05	oxidoreductase activity
Δwc2	8-day	2490518	down	GO:0016491	1,71E-05	oxidoreductase activity
Δwc2	8-day	2497674	down	GO:0016491	1,71E-05	oxidoreductase activity
Δwc2	8-day	2499521	down	GO:0016491	1,71E-05	oxidoreductase activity
Δwc2	8-day	2507164	down	GO:0016491	1,71E-05	oxidoreductase activity

Δwc2	8-day	2515739	down	GO:0016491	1,71E-05	oxidoreductase activity
Δwc2	8-day	2516444	down	GO:0016491	1,71E-05	oxidoreductase activity
Δwc2	8-day	2606988	down	GO:0016491	1,71E-05	oxidoreductase activity
Δwc2	8-day	2608048	down	GO:0016491	1,71E-05	oxidoreductase activity
Δwc2	8-day	2615160	down	GO:0016491	1,71E-05	oxidoreductase activity
Δwc2	8-day	2616880	down	GO:0016491	1,71E-05	oxidoreductase activity
Δwc2	8-day	2616916	down	GO:0016491	1,71E-05	oxidoreductase activity
Δwc2	8-day	2618220	down	GO:0016491	1,71E-05	oxidoreductase activity
Δwc2	8-day	2623663	down	GO:0016491	1,71E-05	oxidoreductase activity
Δwc2	8-day	2625917	down	GO:0016491	1,71E-05	oxidoreductase activity
Δwc2	8-day	2629507	down	GO:0016491	1,71E-05	oxidoreductase activity
Δwc2	8-day	2629850	down	GO:0016491	1,71E-05	oxidoreductase activity
Δwc2	8-day	2637755	down	GO:0016491	1,71E-05	oxidoreductase activity
Δwc2	8-day	2639682	down	GO:0016491	1,71E-05	oxidoreductase activity
Δwc2	8-day	2641520	down	GO:0016491	1,71E-05	oxidoreductase activity
Δwc2	8-day	2661224	down	GO:0016491	1,71E-05	oxidoreductase activity
Δwc2	8-day	2705421	down	GO:0016491	1,71E-05	oxidoreductase activity
Δwc2	8-day	2629603	down	GO:0005199	0,004542905	structural constituent of cell wall
Δwc2	8-day	73881	down	GO:0008152	0,004542905	metabolic process
Δwc2	8-day	1092260	down	GO:0008152	0,004542905	metabolic process
Δwc2	8-day	1189573	down	GO:0008152	0,004542905	metabolic process
Δwc2	8-day	1325481	down	GO:0008152	0,004542905	metabolic process
Δwc2	8-day	2161304	down	GO:0008152	0,004542905	metabolic process
Δwc2	8-day	2495032	down	GO:0008152	0,004542905	metabolic process
Δwc2	8-day	2507164	down	GO:0008152	0,004542905	metabolic process
Δwc2	8-day	2511095	down	GO:0008152	0,004542905	metabolic process
Δwc2	8-day	2540071	down	GO:0008152	0,004542905	metabolic process
Δwc2	8-day	2606988	down	GO:0008152	0,004542905	metabolic process
Δwc2	8-day	2608048	down	GO:0008152	0,004542905	metabolic process
Δwc2	8-day	2612826	down	GO:0008152	0,004542905	metabolic process
Δwc2	8-day	2616880	down	GO:0008152	0,004542905	metabolic process
Δwc2	8-day	2616916	down	GO:0008152	0,004542905	metabolic process
Δwc2	8-day	2618904	down	GO:0008152	0,004542905	metabolic process
Δwc2	8-day	2626867	down	GO:0008152	0,004542905	metabolic process

Δwc2	8-day	2627172	down	GO:0008152	0,004542905	metabolic process
Δwc2	8-day	2629850	down	GO:0008152	0,004542905	metabolic process
Δwc2	8-day	2631112	down	GO:0008152	0,004542905	metabolic process
Δwc2	8-day	2631237	down	GO:0008152	0,004542905	metabolic process
Δwc2	8-day	2637643	down	GO:0008152	0,004542905	metabolic process
Δwc2	8-day	2637755	down	GO:0008152	0,004542905	metabolic process
Δwc2	8-day	2641520	down	GO:0008152	0,004542905	metabolic process
Δwc2	8-day	2705421	down	GO:0008152	0,004542905	metabolic process
Δwc2	8-day	2629603	down	GO:0005618	0,004542905	cell wall
Δwc2	8-day	1131542	down	GO:0004497	0,004542905	monooxygenase activity
Δwc2	8-day	1193647	down	GO:0004497	0,004542905	monooxygenase activity
Δwc2	8-day	2583618	down	GO:0004497	0,004542905	monooxygenase activity
Δwc2	8-day	2616301	down	GO:0004497	0,004542905	monooxygenase activity
Δwc2	8-day	2620407	down	GO:0004497	0,004542905	monooxygenase activity
Δwc2	8-day	2629864	down	GO:0004497	0,004542905	monooxygenase activity
Δwc2	8-day	2632925	down	GO:0004497	0,004542905	monooxygenase activity
Δwc2	8-day	2635487	down	GO:0004497	0,004542905	monooxygenase activity
Δwc2	8-day	2636544	down	GO:0004497	0,004542905	monooxygenase activity
Δwc2	8-day	2638355	down	GO:0004497	0,004542905	monooxygenase activity
Δwc2	8-day	2668568	down	GO:0004497	0,004542905	monooxygenase activity
Δwc2	8-day	2703965	down	GO:0004497	0,004542905	monooxygenase activity
Δwc2	8-day	2703966	down	GO:0004497	0,004542905	monooxygenase activity
Δwc2	8-day	2705258	down	GO:0004497	0,004542905	monooxygenase activity
Δwc2	8-day	2735179	down	GO:0004497	0,004542905	monooxygenase activity
Δwc2	8-day	2675831	down	GO:0015171	0,009934347	amino acid transmembrane transporter activity
Δwc2	8-day	2675831	down	GO:0006865	0,009934347	amino acid transport
Δwc2	8-day	2641520	down	GO:0004316	0,019508114	3-oxoacyl-[acyl-carrier-protein] reductase activity
Δwc2	8-day	2705421	down	GO:0004316	0,019508114	3-oxoacyl-[acyl-carrier-protein] reductase activity
Δwc2	8-day	68300	down	GO:0006810	0,023640689	transport
Δwc2	8-day	1163017	down	GO:0006810	0,023640689	transport
Δwc2	8-day	1171024	down	GO:0006810	0,023640689	transport
Δwc2	8-day	2283957	down	GO:0006810	0,023640689	transport
Δwc2	8-day	2516374	down	GO:0006810	0,023640689	transport
Δwc2	8-day	2519917	down	GO:0006810	0,023640689	transport

Δwc2	8-day	2543516	down	GO:0006810	0,023640689	transport
Δwc2	8-day	2550918	down	GO:0006810	0,023640689	transport
Δwc2	8-day	2617729	down	GO:0006810	0,023640689	transport
Δwc2	8-day	2623492	down	GO:0006810	0,023640689	transport
Δwc2	8-day	2627703	down	GO:0006810	0,023640689	transport
Δwc2	8-day	2661224	down	GO:0006810	0,023640689	transport
Δwc2	8-day	2675831	down	GO:0006810	0,023640689	transport
Δwc2	8-day	2701571	down	GO:0006810	0,023640689	transport
Δwc2	8-day	1131542	down	GO:0006118	0,028824383	electron transport
Δwc2	8-day	1193647	down	GO:0006118	0,028824383	electron transport
Δwc2	8-day	2490518	down	GO:0006118	0,028824383	electron transport
Δwc2	8-day	2497674	down	GO:0006118	0,028824383	electron transport
Δwc2	8-day	2499521	down	GO:0006118	0,028824383	electron transport
Δwc2	8-day	2583618	down	GO:0006118	0,028824383	electron transport
Δwc2	8-day	2616301	down	GO:0006118	0,028824383	electron transport
Δwc2	8-day	2620407	down	GO:0006118	0,028824383	electron transport
Δwc2	8-day	2629850	down	GO:0006118	0,028824383	electron transport
Δwc2	8-day	2629864	down	GO:0006118	0,028824383	electron transport
Δwc2	8-day	2632925	down	GO:0006118	0,028824383	electron transport
Δwc2	8-day	2635487	down	GO:0006118	0,028824383	electron transport
Δwc2	8-day	2636544	down	GO:0006118	0,028824383	electron transport
Δwc2	8-day	2638355	down	GO:0006118	0,028824383	electron transport
Δwc2	8-day	2639682	down	GO:0006118	0,028824383	electron transport
Δwc2	8-day	2668568	down	GO:0006118	0,028824383	electron transport
Δwc2	8-day	2703965	down	GO:0006118	0,028824383	electron transport
Δwc2	8-day	2703966	down	GO:0006118	0,028824383	electron transport
Δwc2	8-day	2705258	down	GO:0006118	0,028824383	electron transport
Δwc2	8-day	2735179	down	GO:0006118	0,028824383	electron transport
Δwc2	8-day	2577992	down	GO:0004194	0,028824383	pepsin A activity
Δbri1	12-day	2502504	down	GO:0005975	0,000249884	carbohydrate metabolic process
Δbri1	12-day	2612719	down	GO:0005975	0,000249884	carbohydrate metabolic process
Δbri1	12-day	2645945	down	GO:0005975	0,000249884	carbohydrate metabolic process
Δbri1	12-day	2502504	down	GO:0004553	0,000308535	hydrolase activity, hydrolyzing O-glycosyl compounds
Δbri1	12-day	2612719	down	GO:0004553	0,000308535	hydrolase activity, hydrolyzing O-glycosyl compounds

Δbri1	12-day	1194451	down	GO:0016491	0,000308535	oxidoreductase activity
Δbri1	12-day	2516444	down	GO:0016491	0,000308535	oxidoreductase activity
Δbri1	12-day	2524219	down	GO:0016491	0,000308535	oxidoreductase activity
Δbri1	12-day	2577740	down	GO:0016491	0,000308535	oxidoreductase activity
Δbri1	12-day	2606988	down	GO:0016491	0,000308535	oxidoreductase activity
Δbri1	12-day	2615160	down	GO:0016491	0,000308535	oxidoreductase activity
Δbri1	12-day	2625561	down	GO:0016491	0,000308535	oxidoreductase activity
Δbri1	12-day	2627304	down	GO:0016491	0,000308535	oxidoreductase activity
Δbri1	12-day	2637755	down	GO:0016491	0,000308535	oxidoreductase activity
Δbri1	12-day	2639682	down	GO:0016491	0,000308535	oxidoreductase activity
Δbri1	12-day	2671376	down	GO:0016491	0,000308535	oxidoreductase activity
Δbri1	12-day	2697968	down	GO:0016491	0,000308535	oxidoreductase activity
Δbri1	12-day	2705421	down	GO:0016491	0,000308535	oxidoreductase activity
Δbri1	12-day	2629603	down	GO:0005199	0,000508187	structural constituent of cell wall
Δbri1	12-day	2629603	down	GO:0005618	0,000589421	cell wall
Δbri1	12-day	2368049	down	GO:0003824	0,000589421	catalytic activity
Δbri1	12-day	2484205	down	GO:0003824	0,000589421	catalytic activity
Δbri1	12-day	2488410	down	GO:0003824	0,000589421	catalytic activity
Δbri1	12-day	2501669	down	GO:0003824	0,000589421	catalytic activity
Δbri1	12-day	2516444	down	GO:0003824	0,000589421	catalytic activity
Δbri1	12-day	2543117	down	GO:0003824	0,000589421	catalytic activity
Δbri1	12-day	2577740	down	GO:0003824	0,000589421	catalytic activity
Δbri1	12-day	2577781	down	GO:0003824	0,000589421	catalytic activity
Δbri1	12-day	2606988	down	GO:0003824	0,000589421	catalytic activity
Δbri1	12-day	2618904	down	GO:0003824	0,000589421	catalytic activity
Δbri1	12-day	2625561	down	GO:0003824	0,000589421	catalytic activity
Δbri1	12-day	2627304	down	GO:0003824	0,000589421	catalytic activity
Δbri1	12-day	2629622	down	GO:0003824	0,000589421	catalytic activity
Δbri1	12-day	2660684	down	GO:0003824	0,000589421	catalytic activity
Δbri1	12-day	2701028	down	GO:0003824	0,000589421	catalytic activity
Δbri1	12-day	2705421	down	GO:0003824	0,000589421	catalytic activity
Δbri1	12-day	2484205	down	GO:0008152	0,001731762	metabolic process
Δbri1	12-day	2488410	down	GO:0008152	0,001731762	metabolic process
Δbri1	12-day	2501669	down	GO:0008152	0,001731762	metabolic process

Δbri1	12-day	2512248	down	GO:0008152	0,001731762	metabolic process
Δbri1	12-day	2543117	down	GO:0008152	0,001731762	metabolic process
Δbri1	12-day	2577740	down	GO:0008152	0,001731762	metabolic process
Δbri1	12-day	2577781	down	GO:0008152	0,001731762	metabolic process
Δbri1	12-day	2601905	down	GO:0008152	0,001731762	metabolic process
Δbri1	12-day	2606988	down	GO:0008152	0,001731762	metabolic process
Δbri1	12-day	2618904	down	GO:0008152	0,001731762	metabolic process
Δbri1	12-day	2624525	down	GO:0008152	0,001731762	metabolic process
Δbri1	12-day	2625561	down	GO:0008152	0,001731762	metabolic process
Δbri1	12-day	2627304	down	GO:0008152	0,001731762	metabolic process
Δbri1	12-day	2629622	down	GO:0008152	0,001731762	metabolic process
Δbri1	12-day	2637708	down	GO:0008152	0,001731762	metabolic process
Δbri1	12-day	2637755	down	GO:0008152	0,001731762	metabolic process
Δbri1	12-day	2660684	down	GO:0008152	0,001731762	metabolic process
Δbri1	12-day	2701028	down	GO:0008152	0,001731762	metabolic process
Δbri1	12-day	2705421	down	GO:0008152	0,001731762	metabolic process
Δbri1	12-day	2726410	down	GO:0008152	0,001731762	metabolic process
Δbri1	12-day	2481387	down	GO:0005488	0,008212127	binding
Δbri1	12-day	2488410	down	GO:0005488	0,008212127	binding
Δbri1	12-day	2520877	down	GO:0005488	0,008212127	binding
Δbri1	12-day	2577740	down	GO:0005488	0,008212127	binding
Δbri1	12-day	2606988	down	GO:0005488	0,008212127	binding
Δbri1	12-day	2618904	down	GO:0005488	0,008212127	binding
Δbri1	12-day	2625561	down	GO:0005488	0,008212127	binding
Δbri1	12-day	2627304	down	GO:0005488	0,008212127	binding
Δbri1	12-day	2628751	down	GO:0005488	0,008212127	binding
Δbri1	12-day	2660684	down	GO:0005488	0,008212127	binding
Δbri1	12-day	2705421	down	GO:0005488	0,008212127	binding
Δbri1	12-day	2520877	down	GO:0005215	0,03108854	transporter activity
Δbri1	12-day	2628751	down	GO:0005215	0,03108854	transporter activity
Δbri1	12-day	2637509	down	GO:0005215	0,03108854	transporter activity
Δc2h2	12-day	1300522	up	GO:0005506	1,65E-10	iron ion binding
Δc2h2	12-day	2364606	up	GO:0005506	1,65E-10	iron ion binding
Δc2h2	12-day	2493935	up	GO:0005506	1,65E-10	iron ion binding

Δc2h2	12-day	2635870	up	GO:0005506	1,65E-10	iron ion binding
Δc2h2	12-day	1300522	up	GO:0020037	3,21E-10	heme binding
Δc2h2	12-day	2364606	up	GO:0020037	3,21E-10	heme binding
Δc2h2	12-day	2635870	up	GO:0020037	3,21E-10	heme binding
Δc2h2	12-day	2640585	up	GO:0020037	3,21E-10	heme binding
Δc2h2	12-day	2673121	up	GO:0020037	3,21E-10	heme binding
Δc2h2	12-day	1300522	up	GO:0004497	4,46E-08	monooxygenase activity
Δc2h2	12-day	2213880	up	GO:0004497	4,46E-08	monooxygenase activity
Δc2h2	12-day	2364606	up	GO:0004497	4,46E-08	monooxygenase activity
Δc2h2	12-day	2635870	up	GO:0004497	4,46E-08	monooxygenase activity
Δc2h2	12-day	1300522	up	GO:0006118	1,24E-07	electron transport
Δc2h2	12-day	2213880	up	GO:0006118	1,24E-07	electron transport
Δc2h2	12-day	2364606	up	GO:0006118	1,24E-07	electron transport
Δc2h2	12-day	2605889	up	GO:0006118	1,24E-07	electron transport
Δc2h2	12-day	2625730	up	GO:0006118	1,24E-07	electron transport
Δc2h2	12-day	2635870	up	GO:0006118	1,24E-07	electron transport
Δc2h2	12-day	1300522	up	GO:0050381	0,005879196	unspecific monooxygenase activity
Δc2h2	12-day	234329	up	GO:0004553	0,007319074	hydrolase activity, hydrolyzing O-glycosyl compounds
Δc2h2	12-day	2496051	up	GO:0004553	0,007319074	hydrolase activity, hydrolyzing O-glycosyl compounds
Δc2h2	12-day	2611511	up	GO:0004553	0,007319074	hydrolase activity, hydrolyzing O-glycosyl compounds
Δc2h2	12-day	2635003	up	GO:0004553	0,007319074	hydrolase activity, hydrolyzing O-glycosyl compounds
Δc2h2	12-day	2637065	up	GO:0004553	0,007319074	hydrolase activity, hydrolyzing O-glycosyl compounds
Δc2h2	12-day	2643740	up	GO:0004553	0,007319074	hydrolase activity, hydrolyzing O-glycosyl compounds
Δc2h2	12-day	234329	up	GO:0005975	0,00961807	carbohydrate metabolic process
Δc2h2	12-day	2496051	up	GO:0005975	0,00961807	carbohydrate metabolic process
Δc2h2	12-day	2514546	up	GO:0005975	0,00961807	carbohydrate metabolic process
Δc2h2	12-day	2611511	up	GO:0005975	0,00961807	carbohydrate metabolic process
Δc2h2	12-day	2635003	up	GO:0005975	0,00961807	carbohydrate metabolic process
Δc2h2	12-day	2637065	up	GO:0005975	0,00961807	carbohydrate metabolic process
Δc2h2	12-day	2643740	up	GO:0005975	0,00961807	carbohydrate metabolic process
Δc2h2	12-day	2643740	up	GO:0006032	0,017311507	chitin catabolic process
Δc2h2	12-day	2643740	up	GO:0008843	0,037967169	endochitinase activity
Δc2h2	12-day	2643740	up	GO:0004568	0,037967169	chitinase activity
Δc2h2	12-day	85278	down	GO:0003824	2,70E-05	catalytic activity

Δc2h2	12-day	2594114	down	GO:0003824	2,70E-05	catalytic activity
Δc2h2	12-day	2606988	down	GO:0003824	2,70E-05	catalytic activity
Δc2h2	12-day	2607837	down	GO:0003824	2,70E-05	catalytic activity
Δc2h2	12-day	2613213	down	GO:0003824	2,70E-05	catalytic activity
Δc2h2	12-day	2615598	down	GO:0003824	2,70E-05	catalytic activity
Δc2h2	12-day	2625561	down	GO:0003824	2,70E-05	catalytic activity
Δc2h2	12-day	2625782	down	GO:0003824	2,70E-05	catalytic activity
Δc2h2	12-day	2629548	down	GO:0003824	2,70E-05	catalytic activity
Δc2h2	12-day	2629646	down	GO:0003824	2,70E-05	catalytic activity
Δc2h2	12-day	2630634	down	GO:0003824	2,70E-05	catalytic activity
Δc2h2	12-day	2631112	down	GO:0003824	2,70E-05	catalytic activity
Δc2h2	12-day	2633144	down	GO:0003824	2,70E-05	catalytic activity
Δc2h2	12-day	2633167	down	GO:0003824	2,70E-05	catalytic activity
Δc2h2	12-day	2635216	down	GO:0003824	2,70E-05	catalytic activity
Δc2h2	12-day	2637668	down	GO:0003824	2,70E-05	catalytic activity
Δc2h2	12-day	2641506	down	GO:0003824	2,70E-05	catalytic activity
Δc2h2	12-day	2688172	down	GO:0003824	2,70E-05	catalytic activity
Δc2h2	12-day	1202278	down	GO:0008152	2,70E-05	metabolic process
Δc2h2	12-day	2508441	down	GO:0008152	2,70E-05	metabolic process
Δc2h2	12-day	2557571	down	GO:0008152	2,70E-05	metabolic process
Δc2h2	12-day	2594114	down	GO:0008152	2,70E-05	metabolic process
Δc2h2	12-day	2604130	down	GO:0008152	2,70E-05	metabolic process
Δc2h2	12-day	2606988	down	GO:0008152	2,70E-05	metabolic process
Δc2h2	12-day	2607837	down	GO:0008152	2,70E-05	metabolic process
Δc2h2	12-day	2613213	down	GO:0008152	2,70E-05	metabolic process
Δc2h2	12-day	2615598	down	GO:0008152	2,70E-05	metabolic process
Δc2h2	12-day	2623956	down	GO:0008152	2,70E-05	metabolic process
Δc2h2	12-day	2624525	down	GO:0008152	2,70E-05	metabolic process
Δc2h2	12-day	2625561	down	GO:0008152	2,70E-05	metabolic process
Δc2h2	12-day	2625782	down	GO:0008152	2,70E-05	metabolic process
Δc2h2	12-day	2629548	down	GO:0008152	2,70E-05	metabolic process
Δc2h2	12-day	2629646	down	GO:0008152	2,70E-05	metabolic process
Δc2h2	12-day	2631112	down	GO:0008152	2,70E-05	metabolic process
Δc2h2	12-day	2633167	down	GO:0008152	2,70E-05	metabolic process

Δc2h2	12-day	2637668	down	GO:0008152	2,70E-05	metabolic process
Δc2h2	12-day	2637708	down	GO:0008152	2,70E-05	metabolic process
Δc2h2	12-day	2641506	down	GO:0008152	2,70E-05	metabolic process
Δc2h2	12-day	2623956	down	GO:0030170	0,000488024	pyridoxal phosphate binding
Δc2h2	12-day	2641506	down	GO:0004316	0,004883594	3-oxoacyl-[acyl-carrier-protein] reductase activity
Δc2h2	12-day	2703935	down	GO:0005199	0,005530036	structural constituent of cell wall
Δc2h2	12-day	2614029	down	GO:0005618	0,006419692	cell wall
Δc2h2	12-day	2703935	down	GO:0005618	0,006419692	cell wall
Δc2h2	12-day	2594114	down	GO:0005488	0,044636306	binding
Δc2h2	12-day	2603669	down	GO:0005488	0,044636306	binding
Δc2h2	12-day	2606988	down	GO:0005488	0,044636306	binding
Δc2h2	12-day	2613213	down	GO:0005488	0,044636306	binding
Δc2h2	12-day	2625561	down	GO:0005488	0,044636306	binding
Δc2h2	12-day	2625782	down	GO:0005488	0,044636306	binding
Δc2h2	12-day	2628751	down	GO:0005488	0,044636306	binding
Δc2h2	12-day	2633167	down	GO:0005488	0,044636306	binding
Δc2h2	12-day	2637668	down	GO:0005488	0,044636306	binding
Δc2h2	12-day	2641506	down	GO:0005488	0,044636306	binding
Δfst3	12-day	2172097	down	GO:0004553	0,002489186	hydrolase activity, hydrolyzing O-glycosyl compounds
Δfst3	12-day	2496051	down	GO:0004553	0,002489186	hydrolase activity, hydrolyzing O-glycosyl compounds
Δfst3	12-day	2612719	down	GO:0004553	0,002489186	hydrolase activity, hydrolyzing O-glycosyl compounds
Δfst3	12-day	2547619	down	GO:0005199	0,00355692	structural constituent of cell wall
Δfst3	12-day	2547619	down	GO:0005618	0,003903108	cell wall
Δfst3	12-day	2617562	down	GO:0030246	0,009912079	carbohydrate binding
Δfst3	12-day	78628	down	GO:0050381	0,039618458	unspecific monooxygenase activity
Δfst3	12-day	2635869	down	GO:0050381	0,039618458	unspecific monooxygenase activity
Δfst3	12-day	2172097	down	GO:0005975	0,042413126	carbohydrate metabolic process
Δfst3	12-day	2496051	down	GO:0005975	0,042413126	carbohydrate metabolic process
Δfst3	12-day	2507164	down	GO:0005975	0,042413126	carbohydrate metabolic process
Δfst3	12-day	2612719	down	GO:0005975	0,042413126	carbohydrate metabolic process
Δfst3	12-day	2631687	down	GO:0005975	0,042413126	carbohydrate metabolic process
Δfst4	12-day	66483	up	GO:0005975	3,93E-07	carbohydrate metabolic process
Δfst4	12-day	256167	up	GO:0005975	3,93E-07	carbohydrate metabolic process
Δfst4	12-day	2501021	up	GO:0005975	3,93E-07	carbohydrate metabolic process

Δfst4	12-day	2557656	up	GO:0005975	3,93E-07	carbohydrate metabolic process
Δfst4	12-day	2570936	up	GO:0005975	3,93E-07	carbohydrate metabolic process
Δfst4	12-day	2605157	up	GO:0005975	3,93E-07	carbohydrate metabolic process
Δfst4	12-day	2613657	up	GO:0005975	3,93E-07	carbohydrate metabolic process
Δfst4	12-day	2621806	up	GO:0005975	3,93E-07	carbohydrate metabolic process
Δfst4	12-day	2622563	up	GO:0005975	3,93E-07	carbohydrate metabolic process
Δfst4	12-day	2624823	up	GO:0005975	3,93E-07	carbohydrate metabolic process
Δfst4	12-day	2626756	up	GO:0005975	3,93E-07	carbohydrate metabolic process
Δfst4	12-day	2641020	up	GO:0005975	3,93E-07	carbohydrate metabolic process
Δfst4	12-day	2641022	up	GO:0005975	3,93E-07	carbohydrate metabolic process
Δfst4	12-day	2645822	up	GO:0005975	3,93E-07	carbohydrate metabolic process
Δfst4	12-day	2670422	up	GO:0005975	3,93E-07	carbohydrate metabolic process
Δfst4	12-day	2686544	up	GO:0005975	3,93E-07	carbohydrate metabolic process
Δfst4	12-day	2706631	up	GO:0005975	3,93E-07	carbohydrate metabolic process
Δfst4	12-day	13059	up	GO:0006508	0,000103152	proteolysis
Δfst4	12-day	54219	up	GO:0006508	0,000103152	proteolysis
Δfst4	12-day	75642	up	GO:0006508	0,000103152	proteolysis
Δfst4	12-day	1190859	up	GO:0006508	0,000103152	proteolysis
Δfst4	12-day	2494345	up	GO:0006508	0,000103152	proteolysis
Δfst4	12-day	2495563	up	GO:0006508	0,000103152	proteolysis
Δfst4	12-day	2497944	up	GO:0006508	0,000103152	proteolysis
Δfst4	12-day	2501086	up	GO:0006508	0,000103152	proteolysis
Δfst4	12-day	2508594	up	GO:0006508	0,000103152	proteolysis
Δfst4	12-day	2521109	up	GO:0006508	0,000103152	proteolysis
Δfst4	12-day	2525660	up	GO:0006508	0,000103152	proteolysis
Δfst4	12-day	2611979	up	GO:0006508	0,000103152	proteolysis
Δfst4	12-day	2611989	up	GO:0006508	0,000103152	proteolysis
Δfst4	12-day	2616091	up	GO:0006508	0,000103152	proteolysis
Δfst4	12-day	2625721	up	GO:0006508	0,000103152	proteolysis
Δfst4	12-day	2627176	up	GO:0006508	0,000103152	proteolysis
Δfst4	12-day	2630895	up	GO:0006508	0,000103152	proteolysis
Δfst4	12-day	2635416	up	GO:0006508	0,000103152	proteolysis
Δfst4	12-day	2636358	up	GO:0006508	0,000103152	proteolysis
Δfst4	12-day	2637463	up	GO:0006508	0,000103152	proteolysis

Δfst4	12-day	2639300	up	GO:0006508	0,000103152	proteolysis
Δfst4	12-day	2668049	up	GO:0006508	0,000103152	proteolysis
Δfst4	12-day	2703529	up	GO:0006508	0,000103152	proteolysis
Δfst4	12-day	1083109	up	GO:0005506	0,000397283	iron ion binding
Δfst4	12-day	1120318	up	GO:0005506	0,000397283	iron ion binding
Δfst4	12-day	1147397	up	GO:0005506	0,000397283	iron ion binding
Δfst4	12-day	1173144	up	GO:0005506	0,000397283	iron ion binding
Δfst4	12-day	1188813	up	GO:0005506	0,000397283	iron ion binding
Δfst4	12-day	2364606	up	GO:0005506	0,000397283	iron ion binding
Δfst4	12-day	2491624	up	GO:0005506	0,000397283	iron ion binding
Δfst4	12-day	2493935	up	GO:0005506	0,000397283	iron ion binding
Δfst4	12-day	2537529	up	GO:0005506	0,000397283	iron ion binding
Δfst4	12-day	2559987	up	GO:0005506	0,000397283	iron ion binding
Δfst4	12-day	2614692	up	GO:0005506	0,000397283	iron ion binding
Δfst4	12-day	2618220	up	GO:0005506	0,000397283	iron ion binding
Δfst4	12-day	2619716	up	GO:0005506	0,000397283	iron ion binding
Δfst4	12-day	2624973	up	GO:0005506	0,000397283	iron ion binding
Δfst4	12-day	2625023	up	GO:0005506	0,000397283	iron ion binding
Δfst4	12-day	2626362	up	GO:0005506	0,000397283	iron ion binding
Δfst4	12-day	2628300	up	GO:0005506	0,000397283	iron ion binding
Δfst4	12-day	2629864	up	GO:0005506	0,000397283	iron ion binding
Δfst4	12-day	2635870	up	GO:0005506	0,000397283	iron ion binding
Δfst4	12-day	2635949	up	GO:0005506	0,000397283	iron ion binding
Δfst4	12-day	2637467	up	GO:0005506	0,000397283	iron ion binding
Δfst4	12-day	2638282	up	GO:0005506	0,000397283	iron ion binding
Δfst4	12-day	2640550	up	GO:0005506	0,000397283	iron ion binding
Δfst4	12-day	2666440	up	GO:0005506	0,000397283	iron ion binding
Δfst4	12-day	2695849	up	GO:0005506	0,000397283	iron ion binding
Δfst4	12-day	2703965	up	GO:0005506	0,000397283	iron ion binding
Δfst4	12-day	256167	up	GO:0004553	0,000874663	hydrolase activity, hydrolyzing O-glycosyl compounds
Δfst4	12-day	2605157	up	GO:0004553	0,000874663	hydrolase activity, hydrolyzing O-glycosyl compounds
Δfst4	12-day	2613657	up	GO:0004553	0,000874663	hydrolase activity, hydrolyzing O-glycosyl compounds
Δfst4	12-day	2621806	up	GO:0004553	0,000874663	hydrolase activity, hydrolyzing O-glycosyl compounds
Δfst4	12-day	2622563	up	GO:0004553	0,000874663	hydrolase activity, hydrolyzing O-glycosyl compounds

Δfst4	12-day	2645822	up	GO:0004553	0,000874663	hydrolase activity, hydrolyzing O-glycosyl compounds
Δfst4	12-day	2670422	up	GO:0004553	0,000874663	hydrolase activity, hydrolyzing O-glycosyl compounds
Δfst4	12-day	2686544	up	GO:0004553	0,000874663	hydrolase activity, hydrolyzing O-glycosyl compounds
Δfst4	12-day	2706631	up	GO:0004553	0,000874663	hydrolase activity, hydrolyzing O-glycosyl compounds
Δfst4	12-day	1083109	up	GO:0020037	0,000874663	heme binding
Δfst4	12-day	1120318	up	GO:0020037	0,000874663	heme binding
Δfst4	12-day	1147397	up	GO:0020037	0,000874663	heme binding
Δfst4	12-day	1173144	up	GO:0020037	0,000874663	heme binding
Δfst4	12-day	1188813	up	GO:0020037	0,000874663	heme binding
Δfst4	12-day	2364606	up	GO:0020037	0,000874663	heme binding
Δfst4	12-day	2491624	up	GO:0020037	0,000874663	heme binding
Δfst4	12-day	2537529	up	GO:0020037	0,000874663	heme binding
Δfst4	12-day	2559987	up	GO:0020037	0,000874663	heme binding
Δfst4	12-day	2608091	up	GO:0020037	0,000874663	heme binding
Δfst4	12-day	2614692	up	GO:0020037	0,000874663	heme binding
Δfst4	12-day	2619716	up	GO:0020037	0,000874663	heme binding
Δfst4	12-day	2622893	up	GO:0020037	0,000874663	heme binding
Δfst4	12-day	2624973	up	GO:0020037	0,000874663	heme binding
Δfst4	12-day	2625023	up	GO:0020037	0,000874663	heme binding
Δfst4	12-day	2626362	up	GO:0020037	0,000874663	heme binding
Δfst4	12-day	2628300	up	GO:0020037	0,000874663	heme binding
Δfst4	12-day	2629864	up	GO:0020037	0,000874663	heme binding
Δfst4	12-day	2635870	up	GO:0020037	0,000874663	heme binding
Δfst4	12-day	2635949	up	GO:0020037	0,000874663	heme binding
Δfst4	12-day	2637467	up	GO:0020037	0,000874663	heme binding
Δfst4	12-day	2638282	up	GO:0020037	0,000874663	heme binding
Δfst4	12-day	2640550	up	GO:0020037	0,000874663	heme binding
Δfst4	12-day	2640585	up	GO:0020037	0,000874663	heme binding
Δfst4	12-day	2643274	up	GO:0020037	0,000874663	heme binding
Δfst4	12-day	2666440	up	GO:0020037	0,000874663	heme binding
Δfst4	12-day	2673121	up	GO:0020037	0,000874663	heme binding
Δfst4	12-day	2695849	up	GO:0020037	0,000874663	heme binding
Δfst4	12-day	2703965	up	GO:0020037	0,000874663	heme binding
Δfst4	12-day	256167	up	GO:0005215	0,000874663	transporter activity

Δfst4	12-day	1147635	up	GO:0005215	0,000874663	transporter activity
Δfst4	12-day	1196424	up	GO:0005215	0,000874663	transporter activity
Δfst4	12-day	1234296	up	GO:0005215	0,000874663	transporter activity
Δfst4	12-day	1296620	up	GO:0005215	0,000874663	transporter activity
Δfst4	12-day	2481323	up	GO:0005215	0,000874663	transporter activity
Δfst4	12-day	2490525	up	GO:0005215	0,000874663	transporter activity
Δfst4	12-day	2496627	up	GO:0005215	0,000874663	transporter activity
Δfst4	12-day	2499364	up	GO:0005215	0,000874663	transporter activity
Δfst4	12-day	2499677	up	GO:0005215	0,000874663	transporter activity
Δfst4	12-day	2502284	up	GO:0005215	0,000874663	transporter activity
Δfst4	12-day	2508421	up	GO:0005215	0,000874663	transporter activity
Δfst4	12-day	2515031	up	GO:0005215	0,000874663	transporter activity
Δfst4	12-day	2543516	up	GO:0005215	0,000874663	transporter activity
Δfst4	12-day	2564524	up	GO:0005215	0,000874663	transporter activity
Δfst4	12-day	2605636	up	GO:0005215	0,000874663	transporter activity
Δfst4	12-day	2611816	up	GO:0005215	0,000874663	transporter activity
Δfst4	12-day	2615463	up	GO:0005215	0,000874663	transporter activity
Δfst4	12-day	2616961	up	GO:0005215	0,000874663	transporter activity
Δfst4	12-day	2617636	up	GO:0005215	0,000874663	transporter activity
Δfst4	12-day	2618842	up	GO:0005215	0,000874663	transporter activity
Δfst4	12-day	2619010	up	GO:0005215	0,000874663	transporter activity
Δfst4	12-day	2627072	up	GO:0005215	0,000874663	transporter activity
Δfst4	12-day	2628098	up	GO:0005215	0,000874663	transporter activity
Δfst4	12-day	2630257	up	GO:0005215	0,000874663	transporter activity
Δfst4	12-day	2631348	up	GO:0005215	0,000874663	transporter activity
Δfst4	12-day	2632105	up	GO:0005215	0,000874663	transporter activity
Δfst4	12-day	2633206	up	GO:0005215	0,000874663	transporter activity
Δfst4	12-day	2633212	up	GO:0005215	0,000874663	transporter activity
Δfst4	12-day	2633596	up	GO:0005215	0,000874663	transporter activity
Δfst4	12-day	2637652	up	GO:0005215	0,000874663	transporter activity
Δfst4	12-day	2660764	up	GO:0005215	0,000874663	transporter activity
Δfst4	12-day	2663452	up	GO:0005215	0,000874663	transporter activity
Δfst4	12-day	2698170	up	GO:0005215	0,000874663	transporter activity
Δfst4	12-day	2704067	up	GO:0005215	0,000874663	transporter activity

Δfst4	12-day	2749899	up	GO:0005215	0,000874663	transporter activity
Δfst4	12-day	1120318	up	GO:0050381	0,000874663	unspecific monooxygenase activity
Δfst4	12-day	1188813	up	GO:0050381	0,000874663	unspecific monooxygenase activity
Δfst4	12-day	2491624	up	GO:0050381	0,000874663	unspecific monooxygenase activity
Δfst4	12-day	2559987	up	GO:0050381	0,000874663	unspecific monooxygenase activity
Δfst4	12-day	2614692	up	GO:0050381	0,000874663	unspecific monooxygenase activity
Δfst4	12-day	2619716	up	GO:0050381	0,000874663	unspecific monooxygenase activity
Δfst4	12-day	2624973	up	GO:0050381	0,000874663	unspecific monooxygenase activity
Δfst4	12-day	2625023	up	GO:0050381	0,000874663	unspecific monooxygenase activity
Δfst4	12-day	2637467	up	GO:0050381	0,000874663	unspecific monooxygenase activity
Δfst4	12-day	2666440	up	GO:0050381	0,000874663	unspecific monooxygenase activity
Δfst4	12-day	2703965	up	GO:0050381	0,000874663	unspecific monooxygenase activity
Δfst4	12-day	1120318	up	GO:0004497	0,001709546	monooxygenase activity
Δfst4	12-day	1147397	up	GO:0004497	0,001709546	monooxygenase activity
Δfst4	12-day	1173144	up	GO:0004497	0,001709546	monooxygenase activity
Δfst4	12-day	1188813	up	GO:0004497	0,001709546	monooxygenase activity
Δfst4	12-day	1342670	up	GO:0004497	0,001709546	monooxygenase activity
Δfst4	12-day	2213880	up	GO:0004497	0,001709546	monooxygenase activity
Δfst4	12-day	2364606	up	GO:0004497	0,001709546	monooxygenase activity
Δfst4	12-day	2490194	up	GO:0004497	0,001709546	monooxygenase activity
Δfst4	12-day	2491624	up	GO:0004497	0,001709546	monooxygenase activity
Δfst4	12-day	2537529	up	GO:0004497	0,001709546	monooxygenase activity
Δfst4	12-day	2559987	up	GO:0004497	0,001709546	monooxygenase activity
Δfst4	12-day	2573393	up	GO:0004497	0,001709546	monooxygenase activity
Δfst4	12-day	2573396	up	GO:0004497	0,001709546	monooxygenase activity
Δfst4	12-day	2614692	up	GO:0004497	0,001709546	monooxygenase activity
Δfst4	12-day	2619716	up	GO:0004497	0,001709546	monooxygenase activity
Δfst4	12-day	2619840	up	GO:0004497	0,001709546	monooxygenase activity
Δfst4	12-day	2624973	up	GO:0004497	0,001709546	monooxygenase activity
Δfst4	12-day	2625023	up	GO:0004497	0,001709546	monooxygenase activity
Δfst4	12-day	2626362	up	GO:0004497	0,001709546	monooxygenase activity
Δfst4	12-day	2628300	up	GO:0004497	0,001709546	monooxygenase activity
Δfst4	12-day	2629864	up	GO:0004497	0,001709546	monooxygenase activity
Δfst4	12-day	2635870	up	GO:0004497	0,001709546	monooxygenase activity

Δfst4	12-day	2635949	up	GO:0004497	0,001709546	monooxygenase activity
Δfst4	12-day	2637467	up	GO:0004497	0,001709546	monooxygenase activity
Δfst4	12-day	2638282	up	GO:0004497	0,001709546	monooxygenase activity
Δfst4	12-day	2640550	up	GO:0004497	0,001709546	monooxygenase activity
Δfst4	12-day	2666440	up	GO:0004497	0,001709546	monooxygenase activity
Δfst4	12-day	2695849	up	GO:0004497	0,001709546	monooxygenase activity
Δfst4	12-day	2703927	up	GO:0004497	0,001709546	monooxygenase activity
Δfst4	12-day	2703965	up	GO:0004497	0,001709546	monooxygenase activity
Δfst4	12-day	1120318	up	GO:0006118	0,006467074	electron transport
Δfst4	12-day	1147397	up	GO:0006118	0,006467074	electron transport
Δfst4	12-day	1173144	up	GO:0006118	0,006467074	electron transport
Δfst4	12-day	1188813	up	GO:0006118	0,006467074	electron transport
Δfst4	12-day	1215660	up	GO:0006118	0,006467074	electron transport
Δfst4	12-day	1342670	up	GO:0006118	0,006467074	electron transport
Δfst4	12-day	2213880	up	GO:0006118	0,006467074	electron transport
Δfst4	12-day	2364606	up	GO:0006118	0,006467074	electron transport
Δfst4	12-day	2457440	up	GO:0006118	0,006467074	electron transport
Δfst4	12-day	2490194	up	GO:0006118	0,006467074	electron transport
Δfst4	12-day	2490518	up	GO:0006118	0,006467074	electron transport
Δfst4	12-day	2491624	up	GO:0006118	0,006467074	electron transport
Δfst4	12-day	2499521	up	GO:0006118	0,006467074	electron transport
Δfst4	12-day	2505277	up	GO:0006118	0,006467074	electron transport
Δfst4	12-day	2507628	up	GO:0006118	0,006467074	electron transport
Δfst4	12-day	2525011	up	GO:0006118	0,006467074	electron transport
Δfst4	12-day	2537529	up	GO:0006118	0,006467074	electron transport
Δfst4	12-day	2559987	up	GO:0006118	0,006467074	electron transport
Δfst4	12-day	2571870	up	GO:0006118	0,006467074	electron transport
Δfst4	12-day	2573393	up	GO:0006118	0,006467074	electron transport
Δfst4	12-day	2573396	up	GO:0006118	0,006467074	electron transport
Δfst4	12-day	2576903	up	GO:0006118	0,006467074	electron transport
Δfst4	12-day	2594205	up	GO:0006118	0,006467074	electron transport
Δfst4	12-day	2605889	up	GO:0006118	0,006467074	electron transport
Δfst4	12-day	2611761	up	GO:0006118	0,006467074	electron transport
Δfst4	12-day	2612998	up	GO:0006118	0,006467074	electron transport

Δfst4	12-day	2614692	up	GO:0006118	0,006467074	electron transport
Δfst4	12-day	2615184	up	GO:0006118	0,006467074	electron transport
Δfst4	12-day	2619716	up	GO:0006118	0,006467074	electron transport
Δfst4	12-day	2619840	up	GO:0006118	0,006467074	electron transport
Δfst4	12-day	2620726	up	GO:0006118	0,006467074	electron transport
Δfst4	12-day	2622259	up	GO:0006118	0,006467074	electron transport
Δfst4	12-day	2622733	up	GO:0006118	0,006467074	electron transport
Δfst4	12-day	2622740	up	GO:0006118	0,006467074	electron transport
Δfst4	12-day	2624973	up	GO:0006118	0,006467074	electron transport
Δfst4	12-day	2625023	up	GO:0006118	0,006467074	electron transport
Δfst4	12-day	2626362	up	GO:0006118	0,006467074	electron transport
Δfst4	12-day	2628120	up	GO:0006118	0,006467074	electron transport
Δfst4	12-day	2628300	up	GO:0006118	0,006467074	electron transport
Δfst4	12-day	2629864	up	GO:0006118	0,006467074	electron transport
Δfst4	12-day	2635870	up	GO:0006118	0,006467074	electron transport
Δfst4	12-day	2635949	up	GO:0006118	0,006467074	electron transport
Δfst4	12-day	2637467	up	GO:0006118	0,006467074	electron transport
Δfst4	12-day	2638282	up	GO:0006118	0,006467074	electron transport
Δfst4	12-day	2638473	up	GO:0006118	0,006467074	electron transport
Δfst4	12-day	2640550	up	GO:0006118	0,006467074	electron transport
Δfst4	12-day	2643274	up	GO:0006118	0,006467074	electron transport
Δfst4	12-day	2666009	up	GO:0006118	0,006467074	electron transport
Δfst4	12-day	2666440	up	GO:0006118	0,006467074	electron transport
Δfst4	12-day	2667289	up	GO:0006118	0,006467074	electron transport
Δfst4	12-day	2695849	up	GO:0006118	0,006467074	electron transport
Δfst4	12-day	2698211	up	GO:0006118	0,006467074	electron transport
Δfst4	12-day	2703927	up	GO:0006118	0,006467074	electron transport
Δfst4	12-day	2703965	up	GO:0006118	0,006467074	electron transport
Δfst4	12-day	1147635	up	GO:0005351	0,008205461	sugar:hydrogen symporter activity
Δfst4	12-day	2499677	up	GO:0005351	0,008205461	sugar:hydrogen symporter activity
Δfst4	12-day	2515031	up	GO:0005351	0,008205461	sugar:hydrogen symporter activity
Δfst4	12-day	2610180	up	GO:0005351	0,008205461	sugar:hydrogen symporter activity
Δfst4	12-day	2619010	up	GO:0005351	0,008205461	sugar:hydrogen symporter activity
Δfst4	12-day	2704067	up	GO:0005351	0,008205461	sugar:hydrogen symporter activity

Δfst4	12-day	2749899	up	GO:0005351	0,008205461	sugar:hydrogen symporter activity
Δfst4	12-day	2521109	up	GO:0008236	0,008205461	serine-type peptidase activity
Δfst4	12-day	2616091	up	GO:0008236	0,008205461	serine-type peptidase activity
Δfst4	12-day	2627176	up	GO:0008236	0,008205461	serine-type peptidase activity
Δfst4	12-day	1147635	up	GO:0008643	0,010579492	carbohydrate transport
Δfst4	12-day	2499677	up	GO:0008643	0,010579492	carbohydrate transport
Δfst4	12-day	2515031	up	GO:0008643	0,010579492	carbohydrate transport
Δfst4	12-day	2619010	up	GO:0008643	0,010579492	carbohydrate transport
Δfst4	12-day	2704067	up	GO:0008643	0,010579492	carbohydrate transport
Δfst4	12-day	2749899	up	GO:0008643	0,010579492	carbohydrate transport
Δfst4	12-day	2703529	up	GO:0004222	0,015918997	metalloendopeptidase activity
Δfst4	12-day	1147635	up	GO:0008733	0,015918997	L-arabinose isomerase activity
Δfst4	12-day	2499677	up	GO:0008733	0,015918997	L-arabinose isomerase activity
Δfst4	12-day	2515031	up	GO:0008733	0,015918997	L-arabinose isomerase activity
Δfst4	12-day	2564524	up	GO:0008733	0,015918997	L-arabinose isomerase activity
Δfst4	12-day	2619010	up	GO:0008733	0,015918997	L-arabinose isomerase activity
Δfst4	12-day	2704067	up	GO:0008733	0,015918997	L-arabinose isomerase activity
Δfst4	12-day	2749899	up	GO:0008733	0,015918997	L-arabinose isomerase activity
Δfst4	12-day	256167	up	GO:0016021	0,026975913	integral to membrane
Δfst4	12-day	1147635	up	GO:0016021	0,026975913	integral to membrane
Δfst4	12-day	1163017	up	GO:0016021	0,026975913	integral to membrane
Δfst4	12-day	1181112	up	GO:0016021	0,026975913	integral to membrane
Δfst4	12-day	1234296	up	GO:0016021	0,026975913	integral to membrane
Δfst4	12-day	1296620	up	GO:0016021	0,026975913	integral to membrane
Δfst4	12-day	2481323	up	GO:0016021	0,026975913	integral to membrane
Δfst4	12-day	2491032	up	GO:0016021	0,026975913	integral to membrane
Δfst4	12-day	2493556	up	GO:0016021	0,026975913	integral to membrane
Δfst4	12-day	2496627	up	GO:0016021	0,026975913	integral to membrane
Δfst4	12-day	2499364	up	GO:0016021	0,026975913	integral to membrane
Δfst4	12-day	2499677	up	GO:0016021	0,026975913	integral to membrane
Δfst4	12-day	2508421	up	GO:0016021	0,026975913	integral to membrane
Δfst4	12-day	2515031	up	GO:0016021	0,026975913	integral to membrane
Δfst4	12-day	2540007	up	GO:0016021	0,026975913	integral to membrane
Δfst4	12-day	2543516	up	GO:0016021	0,026975913	integral to membrane

Δfst4	12-day	2559621	up	GO:0016021	0,026975913	integral to membrane
Δfst4	12-day	2564524	up	GO:0016021	0,026975913	integral to membrane
Δfst4	12-day	2566280	up	GO:0016021	0,026975913	integral to membrane
Δfst4	12-day	2604210	up	GO:0016021	0,026975913	integral to membrane
Δfst4	12-day	2605636	up	GO:0016021	0,026975913	integral to membrane
Δfst4	12-day	2611816	up	GO:0016021	0,026975913	integral to membrane
Δfst4	12-day	2616961	up	GO:0016021	0,026975913	integral to membrane
Δfst4	12-day	2617636	up	GO:0016021	0,026975913	integral to membrane
Δfst4	12-day	2618122	up	GO:0016021	0,026975913	integral to membrane
Δfst4	12-day	2618842	up	GO:0016021	0,026975913	integral to membrane
Δfst4	12-day	2618885	up	GO:0016021	0,026975913	integral to membrane
Δfst4	12-day	2619010	up	GO:0016021	0,026975913	integral to membrane
Δfst4	12-day	2621209	up	GO:0016021	0,026975913	integral to membrane
Δfst4	12-day	2621533	up	GO:0016021	0,026975913	integral to membrane
Δfst4	12-day	2621626	up	GO:0016021	0,026975913	integral to membrane
Δfst4	12-day	2622310	up	GO:0016021	0,026975913	integral to membrane
Δfst4	12-day	2625385	up	GO:0016021	0,026975913	integral to membrane
Δfst4	12-day	2626360	up	GO:0016021	0,026975913	integral to membrane
Δfst4	12-day	2626581	up	GO:0016021	0,026975913	integral to membrane
Δfst4	12-day	2630257	up	GO:0016021	0,026975913	integral to membrane
Δfst4	12-day	2631348	up	GO:0016021	0,026975913	integral to membrane
Δfst4	12-day	2632105	up	GO:0016021	0,026975913	integral to membrane
Δfst4	12-day	2632855	up	GO:0016021	0,026975913	integral to membrane
Δfst4	12-day	2633206	up	GO:0016021	0,026975913	integral to membrane
Δfst4	12-day	2633212	up	GO:0016021	0,026975913	integral to membrane
Δfst4	12-day	2635198	up	GO:0016021	0,026975913	integral to membrane
Δfst4	12-day	2635448	up	GO:0016021	0,026975913	integral to membrane
Δfst4	12-day	2637652	up	GO:0016021	0,026975913	integral to membrane
Δfst4	12-day	2642542	up	GO:0016021	0,026975913	integral to membrane
Δfst4	12-day	2642967	up	GO:0016021	0,026975913	integral to membrane
Δfst4	12-day	2660764	up	GO:0016021	0,026975913	integral to membrane
Δfst4	12-day	2663452	up	GO:0016021	0,026975913	integral to membrane
Δfst4	12-day	2675859	up	GO:0016021	0,026975913	integral to membrane
Δfst4	12-day	2698170	up	GO:0016021	0,026975913	integral to membrane

Δfst4	12-day	2704067	up	GO:0016021	0,026975913	integral to membrane
Δfst4	12-day	2733418	up	GO:0016021	0,026975913	integral to membrane
Δfst4	12-day	2749899	up	GO:0016021	0,026975913	integral to membrane
Δfst4	12-day	2750441	up	GO:0016021	0,026975913	integral to membrane
Δfst4	12-day	2642810	up	GO:0004555	0,039301358	alpha,alpha-trehalase activity
Δfst4	12-day	2634281	up	GO:0008678	0,039301358	2-deoxy-D-gluconate 3-dehydrogenase activity
Δfst4	12-day	2642810	up	GO:0005991	0,039301358	trehalose metabolic process
Δfst4	12-day	237513	down	GO:0016491	5,46E-11	oxidoreductase activity
Δfst4	12-day	257768	down	GO:0016491	5,46E-11	oxidoreductase activity
Δfst4	12-day	1152293	down	GO:0016491	5,46E-11	oxidoreductase activity
Δfst4	12-day	2255031	down	GO:0016491	5,46E-11	oxidoreductase activity
Δfst4	12-day	2333133	down	GO:0016491	5,46E-11	oxidoreductase activity
Δfst4	12-day	2495855	down	GO:0016491	5,46E-11	oxidoreductase activity
Δfst4	12-day	2503108	down	GO:0016491	5,46E-11	oxidoreductase activity
Δfst4	12-day	2504654	down	GO:0016491	5,46E-11	oxidoreductase activity
Δfst4	12-day	2507164	down	GO:0016491	5,46E-11	oxidoreductase activity
Δfst4	12-day	2516444	down	GO:0016491	5,46E-11	oxidoreductase activity
Δfst4	12-day	2524219	down	GO:0016491	5,46E-11	oxidoreductase activity
Δfst4	12-day	2532967	down	GO:0016491	5,46E-11	oxidoreductase activity
Δfst4	12-day	2538948	down	GO:0016491	5,46E-11	oxidoreductase activity
Δfst4	12-day	2604174	down	GO:0016491	5,46E-11	oxidoreductase activity
Δfst4	12-day	2604326	down	GO:0016491	5,46E-11	oxidoreductase activity
Δfst4	12-day	2605752	down	GO:0016491	5,46E-11	oxidoreductase activity
Δfst4	12-day	2605787	down	GO:0016491	5,46E-11	oxidoreductase activity
Δfst4	12-day	2606988	down	GO:0016491	5,46E-11	oxidoreductase activity
Δfst4	12-day	2608048	down	GO:0016491	5,46E-11	oxidoreductase activity
Δfst4	12-day	2609840	down	GO:0016491	5,46E-11	oxidoreductase activity
Δfst4	12-day	2610587	down	GO:0016491	5,46E-11	oxidoreductase activity
Δfst4	12-day	2610599	down	GO:0016491	5,46E-11	oxidoreductase activity
Δfst4	12-day	2612591	down	GO:0016491	5,46E-11	oxidoreductase activity
Δfst4	12-day	2614759	down	GO:0016491	5,46E-11	oxidoreductase activity
Δfst4	12-day	2616880	down	GO:0016491	5,46E-11	oxidoreductase activity
Δfst4	12-day	2616916	down	GO:0016491	5,46E-11	oxidoreductase activity
Δfst4	12-day	2618455	down	GO:0016491	5,46E-11	oxidoreductase activity

Δfst4	12-day	2621873	down	GO:0016491	5,46E-11	oxidoreductase activity
Δfst4	12-day	2622063	down	GO:0016491	5,46E-11	oxidoreductase activity
Δfst4	12-day	2622616	down	GO:0016491	5,46E-11	oxidoreductase activity
Δfst4	12-day	2623301	down	GO:0016491	5,46E-11	oxidoreductase activity
Δfst4	12-day	2625561	down	GO:0016491	5,46E-11	oxidoreductase activity
Δfst4	12-day	2629526	down	GO:0016491	5,46E-11	oxidoreductase activity
Δfst4	12-day	2629948	down	GO:0016491	5,46E-11	oxidoreductase activity
Δfst4	12-day	2630637	down	GO:0016491	5,46E-11	oxidoreductase activity
Δfst4	12-day	2631774	down	GO:0016491	5,46E-11	oxidoreductase activity
Δfst4	12-day	2633167	down	GO:0016491	5,46E-11	oxidoreductase activity
Δfst4	12-day	2633632	down	GO:0016491	5,46E-11	oxidoreductase activity
Δfst4	12-day	2634450	down	GO:0016491	5,46E-11	oxidoreductase activity
Δfst4	12-day	2637104	down	GO:0016491	5,46E-11	oxidoreductase activity
Δfst4	12-day	2638484	down	GO:0016491	5,46E-11	oxidoreductase activity
Δfst4	12-day	2641506	down	GO:0016491	5,46E-11	oxidoreductase activity
Δfst4	12-day	2641520	down	GO:0016491	5,46E-11	oxidoreductase activity
Δfst4	12-day	2643891	down	GO:0016491	5,46E-11	oxidoreductase activity
Δfst4	12-day	2644330	down	GO:0016491	5,46E-11	oxidoreductase activity
Δfst4	12-day	2644430	down	GO:0016491	5,46E-11	oxidoreductase activity
Δfst4	12-day	2661224	down	GO:0016491	5,46E-11	oxidoreductase activity
Δfst4	12-day	2663372	down	GO:0016491	5,46E-11	oxidoreductase activity
Δfst4	12-day	2671376	down	GO:0016491	5,46E-11	oxidoreductase activity
Δfst4	12-day	2688622	down	GO:0016491	5,46E-11	oxidoreductase activity
Δfst4	12-day	2697968	down	GO:0016491	5,46E-11	oxidoreductase activity
Δfst4	12-day	2705421	down	GO:0016491	5,46E-11	oxidoreductase activity
Δfst4	12-day	1152293	down	GO:0008152	9,00E-08	metabolic process
Δfst4	12-day	1200798	down	GO:0008152	9,00E-08	metabolic process
Δfst4	12-day	2255031	down	GO:0008152	9,00E-08	metabolic process
Δfst4	12-day	2333133	down	GO:0008152	9,00E-08	metabolic process
Δfst4	12-day	2485727	down	GO:0008152	9,00E-08	metabolic process
Δfst4	12-day	2492878	down	GO:0008152	9,00E-08	metabolic process
Δfst4	12-day	2495855	down	GO:0008152	9,00E-08	metabolic process
Δfst4	12-day	2507164	down	GO:0008152	9,00E-08	metabolic process
Δfst4	12-day	2511020	down	GO:0008152	9,00E-08	metabolic process

Δfst4	12-day	2533120	down	GO:0008152	9,00E-08	metabolic process
Δfst4	12-day	2553610	down	GO:0008152	9,00E-08	metabolic process
Δfst4	12-day	2605752	down	GO:0008152	9,00E-08	metabolic process
Δfst4	12-day	2605787	down	GO:0008152	9,00E-08	metabolic process
Δfst4	12-day	2606988	down	GO:0008152	9,00E-08	metabolic process
Δfst4	12-day	2608048	down	GO:0008152	9,00E-08	metabolic process
Δfst4	12-day	2609150	down	GO:0008152	9,00E-08	metabolic process
Δfst4	12-day	2610587	down	GO:0008152	9,00E-08	metabolic process
Δfst4	12-day	2610599	down	GO:0008152	9,00E-08	metabolic process
Δfst4	12-day	2611306	down	GO:0008152	9,00E-08	metabolic process
Δfst4	12-day	2612591	down	GO:0008152	9,00E-08	metabolic process
Δfst4	12-day	2614759	down	GO:0008152	9,00E-08	metabolic process
Δfst4	12-day	2615598	down	GO:0008152	9,00E-08	metabolic process
Δfst4	12-day	2616602	down	GO:0008152	9,00E-08	metabolic process
Δfst4	12-day	2616880	down	GO:0008152	9,00E-08	metabolic process
Δfst4	12-day	2616916	down	GO:0008152	9,00E-08	metabolic process
Δfst4	12-day	2618455	down	GO:0008152	9,00E-08	metabolic process
Δfst4	12-day	2618904	down	GO:0008152	9,00E-08	metabolic process
Δfst4	12-day	2619714	down	GO:0008152	9,00E-08	metabolic process
Δfst4	12-day	2621873	down	GO:0008152	9,00E-08	metabolic process
Δfst4	12-day	2622063	down	GO:0008152	9,00E-08	metabolic process
Δfst4	12-day	2623062	down	GO:0008152	9,00E-08	metabolic process
Δfst4	12-day	2623068	down	GO:0008152	9,00E-08	metabolic process
Δfst4	12-day	2623301	down	GO:0008152	9,00E-08	metabolic process
Δfst4	12-day	2624525	down	GO:0008152	9,00E-08	metabolic process
Δfst4	12-day	2624675	down	GO:0008152	9,00E-08	metabolic process
Δfst4	12-day	2625561	down	GO:0008152	9,00E-08	metabolic process
Δfst4	12-day	2625706	down	GO:0008152	9,00E-08	metabolic process
Δfst4	12-day	2625782	down	GO:0008152	9,00E-08	metabolic process
Δfst4	12-day	2626510	down	GO:0008152	9,00E-08	metabolic process
Δfst4	12-day	2629526	down	GO:0008152	9,00E-08	metabolic process
Δfst4	12-day	2629548	down	GO:0008152	9,00E-08	metabolic process
Δfst4	12-day	2631774	down	GO:0008152	9,00E-08	metabolic process
Δfst4	12-day	2632206	down	GO:0008152	9,00E-08	metabolic process

Δfst4	12-day	2632611	down	GO:0008152	9,00E-08	metabolic process
Δfst4	12-day	2633167	down	GO:0008152	9,00E-08	metabolic process
Δfst4	12-day	2633632	down	GO:0008152	9,00E-08	metabolic process
Δfst4	12-day	2633644	down	GO:0008152	9,00E-08	metabolic process
Δfst4	12-day	2634450	down	GO:0008152	9,00E-08	metabolic process
Δfst4	12-day	2637088	down	GO:0008152	9,00E-08	metabolic process
Δfst4	12-day	2637104	down	GO:0008152	9,00E-08	metabolic process
Δfst4	12-day	2637643	down	GO:0008152	9,00E-08	metabolic process
Δfst4	12-day	2637668	down	GO:0008152	9,00E-08	metabolic process
Δfst4	12-day	2638484	down	GO:0008152	9,00E-08	metabolic process
Δfst4	12-day	2640484	down	GO:0008152	9,00E-08	metabolic process
Δfst4	12-day	2641506	down	GO:0008152	9,00E-08	metabolic process
Δfst4	12-day	2641520	down	GO:0008152	9,00E-08	metabolic process
Δfst4	12-day	2643891	down	GO:0008152	9,00E-08	metabolic process
Δfst4	12-day	2644330	down	GO:0008152	9,00E-08	metabolic process
Δfst4	12-day	2644430	down	GO:0008152	9,00E-08	metabolic process
Δfst4	12-day	2645207	down	GO:0008152	9,00E-08	metabolic process
Δfst4	12-day	2665516	down	GO:0008152	9,00E-08	metabolic process
Δfst4	12-day	2669046	down	GO:0008152	9,00E-08	metabolic process
Δfst4	12-day	2705421	down	GO:0008152	9,00E-08	metabolic process
Δfst4	12-day	2711060	down	GO:0008152	9,00E-08	metabolic process
Δfst4	12-day	2726410	down	GO:0008152	9,00E-08	metabolic process
Δfst4	12-day	85278	down	GO:0003824	8,68E-07	catalytic activity
Δfst4	12-day	237513	down	GO:0003824	8,68E-07	catalytic activity
Δfst4	12-day	1152293	down	GO:0003824	8,68E-07	catalytic activity
Δfst4	12-day	1184723	down	GO:0003824	8,68E-07	catalytic activity
Δfst4	12-day	1191999	down	GO:0003824	8,68E-07	catalytic activity
Δfst4	12-day	1200798	down	GO:0003824	8,68E-07	catalytic activity
Δfst4	12-day	2055308	down	GO:0003824	8,68E-07	catalytic activity
Δfst4	12-day	2255031	down	GO:0003824	8,68E-07	catalytic activity
Δfst4	12-day	2333133	down	GO:0003824	8,68E-07	catalytic activity
Δfst4	12-day	2368049	down	GO:0003824	8,68E-07	catalytic activity
Δfst4	12-day	2484815	down	GO:0003824	8,68E-07	catalytic activity
Δfst4	12-day	2492878	down	GO:0003824	8,68E-07	catalytic activity

Δfst4	12-day	2495855	down	GO:0003824	8,68E-07	catalytic activity
Δfst4	12-day	2511020	down	GO:0003824	8,68E-07	catalytic activity
Δfst4	12-day	2513424	down	GO:0003824	8,68E-07	catalytic activity
Δfst4	12-day	2516444	down	GO:0003824	8,68E-07	catalytic activity
Δfst4	12-day	2598960	down	GO:0003824	8,68E-07	catalytic activity
Δfst4	12-day	2605122	down	GO:0003824	8,68E-07	catalytic activity
Δfst4	12-day	2605752	down	GO:0003824	8,68E-07	catalytic activity
Δfst4	12-day	2605787	down	GO:0003824	8,68E-07	catalytic activity
Δfst4	12-day	2606988	down	GO:0003824	8,68E-07	catalytic activity
Δfst4	12-day	2608048	down	GO:0003824	8,68E-07	catalytic activity
Δfst4	12-day	2610587	down	GO:0003824	8,68E-07	catalytic activity
Δfst4	12-day	2610599	down	GO:0003824	8,68E-07	catalytic activity
Δfst4	12-day	2612591	down	GO:0003824	8,68E-07	catalytic activity
Δfst4	12-day	2614269	down	GO:0003824	8,68E-07	catalytic activity
Δfst4	12-day	2614759	down	GO:0003824	8,68E-07	catalytic activity
Δfst4	12-day	2615598	down	GO:0003824	8,68E-07	catalytic activity
Δfst4	12-day	2616491	down	GO:0003824	8,68E-07	catalytic activity
Δfst4	12-day	2616602	down	GO:0003824	8,68E-07	catalytic activity
Δfst4	12-day	2616880	down	GO:0003824	8,68E-07	catalytic activity
Δfst4	12-day	2616916	down	GO:0003824	8,68E-07	catalytic activity
Δfst4	12-day	2618904	down	GO:0003824	8,68E-07	catalytic activity
Δfst4	12-day	2618997	down	GO:0003824	8,68E-07	catalytic activity
Δfst4	12-day	2619714	down	GO:0003824	8,68E-07	catalytic activity
Δfst4	12-day	2621873	down	GO:0003824	8,68E-07	catalytic activity
Δfst4	12-day	2622063	down	GO:0003824	8,68E-07	catalytic activity
Δfst4	12-day	2623062	down	GO:0003824	8,68E-07	catalytic activity
Δfst4	12-day	2623068	down	GO:0003824	8,68E-07	catalytic activity
Δfst4	12-day	2623301	down	GO:0003824	8,68E-07	catalytic activity
Δfst4	12-day	2624675	down	GO:0003824	8,68E-07	catalytic activity
Δfst4	12-day	2625554	down	GO:0003824	8,68E-07	catalytic activity
Δfst4	12-day	2625561	down	GO:0003824	8,68E-07	catalytic activity
Δfst4	12-day	2625782	down	GO:0003824	8,68E-07	catalytic activity
Δfst4	12-day	2626510	down	GO:0003824	8,68E-07	catalytic activity
Δfst4	12-day	2628678	down	GO:0003824	8,68E-07	catalytic activity

Δfst4	12-day	2629526	down	GO:0003824	8,68E-07	catalytic activity
Δfst4	12-day	2629548	down	GO:0003824	8,68E-07	catalytic activity
Δfst4	12-day	2630634	down	GO:0003824	8,68E-07	catalytic activity
Δfst4	12-day	2631920	down	GO:0003824	8,68E-07	catalytic activity
Δfst4	12-day	2632206	down	GO:0003824	8,68E-07	catalytic activity
Δfst4	12-day	2632611	down	GO:0003824	8,68E-07	catalytic activity
Δfst4	12-day	2633144	down	GO:0003824	8,68E-07	catalytic activity
Δfst4	12-day	2633162	down	GO:0003824	8,68E-07	catalytic activity
Δfst4	12-day	2633167	down	GO:0003824	8,68E-07	catalytic activity
Δfst4	12-day	2633632	down	GO:0003824	8,68E-07	catalytic activity
Δfst4	12-day	2634450	down	GO:0003824	8,68E-07	catalytic activity
Δfst4	12-day	2634918	down	GO:0003824	8,68E-07	catalytic activity
Δfst4	12-day	2635216	down	GO:0003824	8,68E-07	catalytic activity
Δfst4	12-day	2635219	down	GO:0003824	8,68E-07	catalytic activity
Δfst4	12-day	2637088	down	GO:0003824	8,68E-07	catalytic activity
Δfst4	12-day	2637104	down	GO:0003824	8,68E-07	catalytic activity
Δfst4	12-day	2637668	down	GO:0003824	8,68E-07	catalytic activity
Δfst4	12-day	2637783	down	GO:0003824	8,68E-07	catalytic activity
Δfst4	12-day	2637845	down	GO:0003824	8,68E-07	catalytic activity
Δfst4	12-day	2638484	down	GO:0003824	8,68E-07	catalytic activity
Δfst4	12-day	2638783	down	GO:0003824	8,68E-07	catalytic activity
Δfst4	12-day	2640484	down	GO:0003824	8,68E-07	catalytic activity
Δfst4	12-day	2641506	down	GO:0003824	8,68E-07	catalytic activity
Δfst4	12-day	2641520	down	GO:0003824	8,68E-07	catalytic activity
Δfst4	12-day	2643891	down	GO:0003824	8,68E-07	catalytic activity
Δfst4	12-day	2643902	down	GO:0003824	8,68E-07	catalytic activity
Δfst4	12-day	2644330	down	GO:0003824	8,68E-07	catalytic activity
Δfst4	12-day	2645207	down	GO:0003824	8,68E-07	catalytic activity
Δfst4	12-day	2682976	down	GO:0003824	8,68E-07	catalytic activity
Δfst4	12-day	2699217	down	GO:0003824	8,68E-07	catalytic activity
Δfst4	12-day	2705421	down	GO:0003824	8,68E-07	catalytic activity
Δfst4	12-day	2711060	down	GO:0003824	8,68E-07	catalytic activity
Δfst4	12-day	2606344	down	GO:0000786	1,52E-05	nucleosome
Δfst4	12-day	2630193	down	GO:0000786	1,52E-05	nucleosome

Δfst4	12-day	2068434	down	GO:0030170	1,86E-05	pyridoxal phosphate binding
Δfst4	12-day	2553610	down	GO:0030170	1,86E-05	pyridoxal phosphate binding
Δfst4	12-day	2606344	down	GO:0006334	4,51E-05	nucleosome assembly
Δfst4	12-day	2630193	down	GO:0006334	4,51E-05	nucleosome assembly
Δfst4	12-day	78628	down	GO:0006118	5,21E-05	electron transport
Δfst4	12-day	81631	down	GO:0006118	5,21E-05	electron transport
Δfst4	12-day	257768	down	GO:0006118	5,21E-05	electron transport
Δfst4	12-day	1084300	down	GO:0006118	5,21E-05	electron transport
Δfst4	12-day	1131542	down	GO:0006118	5,21E-05	electron transport
Δfst4	12-day	1139445	down	GO:0006118	5,21E-05	electron transport
Δfst4	12-day	1159597	down	GO:0006118	5,21E-05	electron transport
Δfst4	12-day	1189794	down	GO:0006118	5,21E-05	electron transport
Δfst4	12-day	2327903	down	GO:0006118	5,21E-05	electron transport
Δfst4	12-day	2486680	down	GO:0006118	5,21E-05	electron transport
Δfst4	12-day	2503108	down	GO:0006118	5,21E-05	electron transport
Δfst4	12-day	2509716	down	GO:0006118	5,21E-05	electron transport
Δfst4	12-day	2512276	down	GO:0006118	5,21E-05	electron transport
Δfst4	12-day	2516208	down	GO:0006118	5,21E-05	electron transport
Δfst4	12-day	2532967	down	GO:0006118	5,21E-05	electron transport
Δfst4	12-day	2533120	down	GO:0006118	5,21E-05	electron transport
Δfst4	12-day	2604628	down	GO:0006118	5,21E-05	electron transport
Δfst4	12-day	2604735	down	GO:0006118	5,21E-05	electron transport
Δfst4	12-day	2609840	down	GO:0006118	5,21E-05	electron transport
Δfst4	12-day	2611234	down	GO:0006118	5,21E-05	electron transport
Δfst4	12-day	2616301	down	GO:0006118	5,21E-05	electron transport
Δfst4	12-day	2618455	down	GO:0006118	5,21E-05	electron transport
Δfst4	12-day	2619674	down	GO:0006118	5,21E-05	electron transport
Δfst4	12-day	2621855	down	GO:0006118	5,21E-05	electron transport
Δfst4	12-day	2623196	down	GO:0006118	5,21E-05	electron transport
Δfst4	12-day	2628312	down	GO:0006118	5,21E-05	electron transport
Δfst4	12-day	2628651	down	GO:0006118	5,21E-05	electron transport
Δfst4	12-day	2633167	down	GO:0006118	5,21E-05	electron transport
Δfst4	12-day	2635487	down	GO:0006118	5,21E-05	electron transport
Δfst4	12-day	2635869	down	GO:0006118	5,21E-05	electron transport

Δfst4	12-day	2637104	down	GO:0006118	5,21E-05	electron transport
Δfst4	12-day	2637130	down	GO:0006118	5,21E-05	electron transport
Δfst4	12-day	2638355	down	GO:0006118	5,21E-05	electron transport
Δfst4	12-day	2639762	down	GO:0006118	5,21E-05	electron transport
Δfst4	12-day	2644430	down	GO:0006118	5,21E-05	electron transport
Δfst4	12-day	2662585	down	GO:0006118	5,21E-05	electron transport
Δfst4	12-day	2663372	down	GO:0006118	5,21E-05	electron transport
Δfst4	12-day	2668568	down	GO:0006118	5,21E-05	electron transport
Δfst4	12-day	2671376	down	GO:0006118	5,21E-05	electron transport
Δfst4	12-day	2688622	down	GO:0006118	5,21E-05	electron transport
Δfst4	12-day	2703628	down	GO:0006118	5,21E-05	electron transport
Δfst4	12-day	2753029	down	GO:0006118	5,21E-05	electron transport
Δfst4	12-day	2746250	down	GO:0005199	0,000295281	structural constituent of cell wall
Δfst4	12-day	2614257	down	GO:0005618	0,000523114	cell wall
Δfst4	12-day	78628	down	GO:0004497	0,000892414	monooxygenase activity
Δfst4	12-day	81631	down	GO:0004497	0,000892414	monooxygenase activity
Δfst4	12-day	1131542	down	GO:0004497	0,000892414	monooxygenase activity
Δfst4	12-day	1139445	down	GO:0004497	0,000892414	monooxygenase activity
Δfst4	12-day	1159597	down	GO:0004497	0,000892414	monooxygenase activity
Δfst4	12-day	1189794	down	GO:0004497	0,000892414	monooxygenase activity
Δfst4	12-day	2327903	down	GO:0004497	0,000892414	monooxygenase activity
Δfst4	12-day	2486680	down	GO:0004497	0,000892414	monooxygenase activity
Δfst4	12-day	2512276	down	GO:0004497	0,000892414	monooxygenase activity
Δfst4	12-day	2516208	down	GO:0004497	0,000892414	monooxygenase activity
Δfst4	12-day	2604735	down	GO:0004497	0,000892414	monooxygenase activity
Δfst4	12-day	2611234	down	GO:0004497	0,000892414	monooxygenase activity
Δfst4	12-day	2616301	down	GO:0004497	0,000892414	monooxygenase activity
Δfst4	12-day	2618455	down	GO:0004497	0,000892414	monooxygenase activity
Δfst4	12-day	2619674	down	GO:0004497	0,000892414	monooxygenase activity
Δfst4	12-day	2623196	down	GO:0004497	0,000892414	monooxygenase activity
Δfst4	12-day	2628312	down	GO:0004497	0,000892414	monooxygenase activity
Δfst4	12-day	2628651	down	GO:0004497	0,000892414	monooxygenase activity
Δfst4	12-day	2635487	down	GO:0004497	0,000892414	monooxygenase activity
Δfst4	12-day	2635869	down	GO:0004497	0,000892414	monooxygenase activity

Δfst4	12-day	2637130	down	GO:0004497	0,000892414	monooxygenase activity
Δfst4	12-day	2638355	down	GO:0004497	0,000892414	monooxygenase activity
Δfst4	12-day	2644430	down	GO:0004497	0,000892414	monooxygenase activity
Δfst4	12-day	2668568	down	GO:0004497	0,000892414	monooxygenase activity
Δfst4	12-day	2703628	down	GO:0004497	0,000892414	monooxygenase activity
Δfst4	12-day	2753029	down	GO:0004497	0,000892414	monooxygenase activity
Δfst4	12-day	1093619	down	GO:0006810	0,003047456	transport
Δfst4	12-day	1144263	down	GO:0006810	0,003047456	transport
Δfst4	12-day	1154281	down	GO:0006810	0,003047456	transport
Δfst4	12-day	1166919	down	GO:0006810	0,003047456	transport
Δfst4	12-day	2145491	down	GO:0006810	0,003047456	transport
Δfst4	12-day	2162840	down	GO:0006810	0,003047456	transport
Δfst4	12-day	2481653	down	GO:0006810	0,003047456	transport
Δfst4	12-day	2485970	down	GO:0006810	0,003047456	transport
Δfst4	12-day	2492878	down	GO:0006810	0,003047456	transport
Δfst4	12-day	2501677	down	GO:0006810	0,003047456	transport
Δfst4	12-day	2504863	down	GO:0006810	0,003047456	transport
Δfst4	12-day	2509716	down	GO:0006810	0,003047456	transport
Δfst4	12-day	2516374	down	GO:0006810	0,003047456	transport
Δfst4	12-day	2520877	down	GO:0006810	0,003047456	transport
Δfst4	12-day	2525305	down	GO:0006810	0,003047456	transport
Δfst4	12-day	2543225	down	GO:0006810	0,003047456	transport
Δfst4	12-day	2550918	down	GO:0006810	0,003047456	transport
Δfst4	12-day	2607025	down	GO:0006810	0,003047456	transport
Δfst4	12-day	2613255	down	GO:0006810	0,003047456	transport
Δfst4	12-day	2616189	down	GO:0006810	0,003047456	transport
Δfst4	12-day	2616602	down	GO:0006810	0,003047456	transport
Δfst4	12-day	2617458	down	GO:0006810	0,003047456	transport
Δfst4	12-day	2621148	down	GO:0006810	0,003047456	transport
Δfst4	12-day	2622598	down	GO:0006810	0,003047456	transport
Δfst4	12-day	2623492	down	GO:0006810	0,003047456	transport
Δfst4	12-day	2626453	down	GO:0006810	0,003047456	transport
Δfst4	12-day	2626803	down	GO:0006810	0,003047456	transport
Δfst4	12-day	2627703	down	GO:0006810	0,003047456	transport

Δfst4	12-day	2628751	down	GO:0006810	0,003047456	transport
Δfst4	12-day	2629091	down	GO:0006810	0,003047456	transport
Δfst4	12-day	2633216	down	GO:0006810	0,003047456	transport
Δfst4	12-day	2636597	down	GO:0006810	0,003047456	transport
Δfst4	12-day	2637509	down	GO:0006810	0,003047456	transport
Δfst4	12-day	2661224	down	GO:0006810	0,003047456	transport
Δfst4	12-day	2662585	down	GO:0006810	0,003047456	transport
Δfst4	12-day	2681288	down	GO:0006810	0,003047456	transport
Δfst4	12-day	2701571	down	GO:0006810	0,003047456	transport
Δfst4	12-day	2712060	down	GO:0006810	0,003047456	transport
Δfst4	12-day	1093619	down	GO:0005215	0,005405783	transporter activity
Δfst4	12-day	1144263	down	GO:0005215	0,005405783	transporter activity
Δfst4	12-day	1154281	down	GO:0005215	0,005405783	transporter activity
Δfst4	12-day	2145491	down	GO:0005215	0,005405783	transporter activity
Δfst4	12-day	2162840	down	GO:0005215	0,005405783	transporter activity
Δfst4	12-day	2481653	down	GO:0005215	0,005405783	transporter activity
Δfst4	12-day	2485970	down	GO:0005215	0,005405783	transporter activity
Δfst4	12-day	2492878	down	GO:0005215	0,005405783	transporter activity
Δfst4	12-day	2501677	down	GO:0005215	0,005405783	transporter activity
Δfst4	12-day	2504863	down	GO:0005215	0,005405783	transporter activity
Δfst4	12-day	2509716	down	GO:0005215	0,005405783	transporter activity
Δfst4	12-day	2516374	down	GO:0005215	0,005405783	transporter activity
Δfst4	12-day	2520877	down	GO:0005215	0,005405783	transporter activity
Δfst4	12-day	2525305	down	GO:0005215	0,005405783	transporter activity
Δfst4	12-day	2543225	down	GO:0005215	0,005405783	transporter activity
Δfst4	12-day	2550918	down	GO:0005215	0,005405783	transporter activity
Δfst4	12-day	2607025	down	GO:0005215	0,005405783	transporter activity
Δfst4	12-day	2613255	down	GO:0005215	0,005405783	transporter activity
Δfst4	12-day	2616189	down	GO:0005215	0,005405783	transporter activity
Δfst4	12-day	2621148	down	GO:0005215	0,005405783	transporter activity
Δfst4	12-day	2622598	down	GO:0005215	0,005405783	transporter activity
Δfst4	12-day	2623492	down	GO:0005215	0,005405783	transporter activity
Δfst4	12-day	2626453	down	GO:0005215	0,005405783	transporter activity
Δfst4	12-day	2626803	down	GO:0005215	0,005405783	transporter activity

Δfst4	12-day	2627703	down	GO:0005215	0,005405783	transporter activity
Δfst4	12-day	2628751	down	GO:0005215	0,005405783	transporter activity
Δfst4	12-day	2629091	down	GO:0005215	0,005405783	transporter activity
Δfst4	12-day	2633216	down	GO:0005215	0,005405783	transporter activity
Δfst4	12-day	2636597	down	GO:0005215	0,005405783	transporter activity
Δfst4	12-day	2637509	down	GO:0005215	0,005405783	transporter activity
Δfst4	12-day	2642553	down	GO:0005215	0,005405783	transporter activity
Δfst4	12-day	2661224	down	GO:0005215	0,005405783	transporter activity
Δfst4	12-day	2701571	down	GO:0005215	0,005405783	transporter activity
Δfst4	12-day	2712060	down	GO:0005215	0,005405783	transporter activity
Δfst4	12-day	1138917	down	GO:0005488	0,006446617	binding
Δfst4	12-day	1152293	down	GO:0005488	0,006446617	binding
Δfst4	12-day	2085050	down	GO:0005488	0,006446617	binding
Δfst4	12-day	2255031	down	GO:0005488	0,006446617	binding
Δfst4	12-day	2333133	down	GO:0005488	0,006446617	binding
Δfst4	12-day	2492878	down	GO:0005488	0,006446617	binding
Δfst4	12-day	2495855	down	GO:0005488	0,006446617	binding
Δfst4	12-day	2511020	down	GO:0005488	0,006446617	binding
Δfst4	12-day	2520877	down	GO:0005488	0,006446617	binding
Δfst4	12-day	2562034	down	GO:0005488	0,006446617	binding
Δfst4	12-day	2605752	down	GO:0005488	0,006446617	binding
Δfst4	12-day	2605787	down	GO:0005488	0,006446617	binding
Δfst4	12-day	2606988	down	GO:0005488	0,006446617	binding
Δfst4	12-day	2608048	down	GO:0005488	0,006446617	binding
Δfst4	12-day	2610587	down	GO:0005488	0,006446617	binding
Δfst4	12-day	2610599	down	GO:0005488	0,006446617	binding
Δfst4	12-day	2612591	down	GO:0005488	0,006446617	binding
Δfst4	12-day	2614759	down	GO:0005488	0,006446617	binding
Δfst4	12-day	2616880	down	GO:0005488	0,006446617	binding
Δfst4	12-day	2616916	down	GO:0005488	0,006446617	binding
Δfst4	12-day	2618904	down	GO:0005488	0,006446617	binding
Δfst4	12-day	2619248	down	GO:0005488	0,006446617	binding
Δfst4	12-day	2619714	down	GO:0005488	0,006446617	binding
Δfst4	12-day	2621873	down	GO:0005488	0,006446617	binding

Δfst4	12-day	2622063	down	GO:0005488	0,006446617	binding
Δfst4	12-day	2623301	down	GO:0005488	0,006446617	binding
Δfst4	12-day	2624675	down	GO:0005488	0,006446617	binding
Δfst4	12-day	2625561	down	GO:0005488	0,006446617	binding
Δfst4	12-day	2625782	down	GO:0005488	0,006446617	binding
Δfst4	12-day	2626510	down	GO:0005488	0,006446617	binding
Δfst4	12-day	2628751	down	GO:0005488	0,006446617	binding
Δfst4	12-day	2629526	down	GO:0005488	0,006446617	binding
Δfst4	12-day	2632611	down	GO:0005488	0,006446617	binding
Δfst4	12-day	2633167	down	GO:0005488	0,006446617	binding
Δfst4	12-day	2633632	down	GO:0005488	0,006446617	binding
Δfst4	12-day	2634450	down	GO:0005488	0,006446617	binding
Δfst4	12-day	2637088	down	GO:0005488	0,006446617	binding
Δfst4	12-day	2637104	down	GO:0005488	0,006446617	binding
Δfst4	12-day	2637668	down	GO:0005488	0,006446617	binding
Δfst4	12-day	2638484	down	GO:0005488	0,006446617	binding
Δfst4	12-day	2641506	down	GO:0005488	0,006446617	binding
Δfst4	12-day	2641520	down	GO:0005488	0,006446617	binding
Δfst4	12-day	2644330	down	GO:0005488	0,006446617	binding
Δfst4	12-day	2661224	down	GO:0005488	0,006446617	binding
Δfst4	12-day	2661491	down	GO:0005488	0,006446617	binding
Δfst4	12-day	2673954	down	GO:0005488	0,006446617	binding
Δfst4	12-day	2705421	down	GO:0005488	0,006446617	binding
Δfst4	12-day	2711060	down	GO:0005488	0,006446617	binding
Δfst4	12-day	2633632	down	GO:0004316	0,015926484	3-oxoacyl-[acyl-carrier-protein] reductase activity
Δfst4	12-day	2634450	down	GO:0004316	0,015926484	3-oxoacyl-[acyl-carrier-protein] reductase activity
Δfst4	12-day	2641506	down	GO:0004316	0,015926484	3-oxoacyl-[acyl-carrier-protein] reductase activity
Δfst4	12-day	2641520	down	GO:0004316	0,015926484	3-oxoacyl-[acyl-carrier-protein] reductase activity
Δfst4	12-day	2705421	down	GO:0004316	0,015926484	3-oxoacyl-[acyl-carrier-protein] reductase activity
Δfst4	12-day	1036421	down	GO:0016021	0,030484489	integral to membrane
Δfst4	12-day	1091573	down	GO:0016021	0,030484489	integral to membrane
Δfst4	12-day	1093619	down	GO:0016021	0,030484489	integral to membrane
Δfst4	12-day	1144263	down	GO:0016021	0,030484489	integral to membrane
Δfst4	12-day	1154281	down	GO:0016021	0,030484489	integral to membrane

Δfst4	12-day	1166919	down	GO:0016021	0,030484489	integral to membrane
Δfst4	12-day	2145491	down	GO:0016021	0,030484489	integral to membrane
Δfst4	12-day	2481653	down	GO:0016021	0,030484489	integral to membrane
Δfst4	12-day	2483267	down	GO:0016021	0,030484489	integral to membrane
Δfst4	12-day	2485970	down	GO:0016021	0,030484489	integral to membrane
Δfst4	12-day	2503108	down	GO:0016021	0,030484489	integral to membrane
Δfst4	12-day	2504863	down	GO:0016021	0,030484489	integral to membrane
Δfst4	12-day	2509716	down	GO:0016021	0,030484489	integral to membrane
Δfst4	12-day	2516374	down	GO:0016021	0,030484489	integral to membrane
Δfst4	12-day	2524870	down	GO:0016021	0,030484489	integral to membrane
Δfst4	12-day	2525305	down	GO:0016021	0,030484489	integral to membrane
Δfst4	12-day	2538948	down	GO:0016021	0,030484489	integral to membrane
Δfst4	12-day	2543225	down	GO:0016021	0,030484489	integral to membrane
Δfst4	12-day	2550918	down	GO:0016021	0,030484489	integral to membrane
Δfst4	12-day	2558791	down	GO:0016021	0,030484489	integral to membrane
Δfst4	12-day	2592432	down	GO:0016021	0,030484489	integral to membrane
Δfst4	12-day	2604174	down	GO:0016021	0,030484489	integral to membrane
Δfst4	12-day	2604984	down	GO:0016021	0,030484489	integral to membrane
Δfst4	12-day	2607025	down	GO:0016021	0,030484489	integral to membrane
Δfst4	12-day	2613255	down	GO:0016021	0,030484489	integral to membrane
Δfst4	12-day	2616189	down	GO:0016021	0,030484489	integral to membrane
Δfst4	12-day	2616602	down	GO:0016021	0,030484489	integral to membrane
Δfst4	12-day	2621148	down	GO:0016021	0,030484489	integral to membrane
Δfst4	12-day	2622598	down	GO:0016021	0,030484489	integral to membrane
Δfst4	12-day	2623492	down	GO:0016021	0,030484489	integral to membrane
Δfst4	12-day	2626453	down	GO:0016021	0,030484489	integral to membrane
Δfst4	12-day	2626803	down	GO:0016021	0,030484489	integral to membrane
Δfst4	12-day	2627703	down	GO:0016021	0,030484489	integral to membrane
Δfst4	12-day	2629091	down	GO:0016021	0,030484489	integral to membrane
Δfst4	12-day	2633216	down	GO:0016021	0,030484489	integral to membrane
Δfst4	12-day	2636597	down	GO:0016021	0,030484489	integral to membrane
Δfst4	12-day	2637509	down	GO:0016021	0,030484489	integral to membrane
Δfst4	12-day	2638261	down	GO:0016021	0,030484489	integral to membrane
Δfst4	12-day	2641709	down	GO:0016021	0,030484489	integral to membrane

Δfst4	12-day	2663372	down	GO:0016021	0,030484489	integral to membrane
Δfst4	12-day	2667661	down	GO:0016021	0,030484489	integral to membrane
Δfst4	12-day	2681288	down	GO:0016021	0,030484489	integral to membrane
Δfst4	12-day	2686473	down	GO:0016021	0,030484489	integral to membrane
Δfst4	12-day	2691538	down	GO:0016021	0,030484489	integral to membrane
Δfst4	12-day	2701571	down	GO:0016021	0,030484489	integral to membrane
Δfst4	12-day	2704867	down	GO:0016021	0,030484489	integral to membrane
Δfst4	12-day	2712060	down	GO:0016021	0,030484489	integral to membrane
Δfst4	12-day	78628	down	GO:0020037	0,041944195	heme binding
Δfst4	12-day	81631	down	GO:0020037	0,041944195	heme binding
Δfst4	12-day	1131542	down	GO:0020037	0,041944195	heme binding
Δfst4	12-day	1139445	down	GO:0020037	0,041944195	heme binding
Δfst4	12-day	1159597	down	GO:0020037	0,041944195	heme binding
Δfst4	12-day	1189794	down	GO:0020037	0,041944195	heme binding
Δfst4	12-day	2327903	down	GO:0020037	0,041944195	heme binding
Δfst4	12-day	2486680	down	GO:0020037	0,041944195	heme binding
Δfst4	12-day	2509716	down	GO:0020037	0,041944195	heme binding
Δfst4	12-day	2512276	down	GO:0020037	0,041944195	heme binding
Δfst4	12-day	2516208	down	GO:0020037	0,041944195	heme binding
Δfst4	12-day	2604628	down	GO:0020037	0,041944195	heme binding
Δfst4	12-day	2604735	down	GO:0020037	0,041944195	heme binding
Δfst4	12-day	2609840	down	GO:0020037	0,041944195	heme binding
Δfst4	12-day	2611234	down	GO:0020037	0,041944195	heme binding
Δfst4	12-day	2616301	down	GO:0020037	0,041944195	heme binding
Δfst4	12-day	2619674	down	GO:0020037	0,041944195	heme binding
Δfst4	12-day	2621855	down	GO:0020037	0,041944195	heme binding
Δfst4	12-day	2623196	down	GO:0020037	0,041944195	heme binding
Δfst4	12-day	2628312	down	GO:0020037	0,041944195	heme binding
Δfst4	12-day	2628651	down	GO:0020037	0,041944195	heme binding
Δfst4	12-day	2635487	down	GO:0020037	0,041944195	heme binding
Δfst4	12-day	2635869	down	GO:0020037	0,041944195	heme binding
Δfst4	12-day	2637130	down	GO:0020037	0,041944195	heme binding
Δfst4	12-day	2638355	down	GO:0020037	0,041944195	heme binding
Δfst4	12-day	2639762	down	GO:0020037	0,041944195	heme binding

Δfst4	12-day	2668568	down	GO:0020037	0,041944195	heme binding
Δfst4	12-day	2703628	down	GO:0020037	0,041944195	heme binding
Δfst4	12-day	2704129	down	GO:0020037	0,041944195	heme binding
Δfst4	12-day	2753029	down	GO:0020037	0,041944195	heme binding
Δfst4	12-day	237513	down	GO:0050660	0,04567865	FAD binding
Δfst4	12-day	257768	down	GO:0050660	0,04567865	FAD binding
Δfst4	12-day	2343034	down	GO:0050660	0,04567865	FAD binding
Δfst4	12-day	2503108	down	GO:0050660	0,04567865	FAD binding
Δfst4	12-day	2516444	down	GO:0050660	0,04567865	FAD binding
Δfst4	12-day	2607677	down	GO:0050660	0,04567865	FAD binding
Δfst4	12-day	2610771	down	GO:0050660	0,04567865	FAD binding
Δfst4	12-day	2642607	down	GO:0050660	0,04567865	FAD binding
Δfst4	12-day	2663372	down	GO:0050660	0,04567865	FAD binding
Δfst4	12-day	2553610	down	GO:0016769	0,04567865	transferase activity, transferring nitrogenous groups
Δfst4	12-day	2520460	down	GO:0006520	0,04567865	amino acid metabolic process
Δfst4	12-day	1144263	down	GO:0016020	0,048918805	membrane
Δfst4	12-day	2088105	down	GO:0016020	0,048918805	membrane
Δfst4	12-day	2162840	down	GO:0016020	0,048918805	membrane
Δfst4	12-day	2501677	down	GO:0016020	0,048918805	membrane
Δfst4	12-day	2503590	down	GO:0016020	0,048918805	membrane
Δfst4	12-day	2516374	down	GO:0016020	0,048918805	membrane
Δfst4	12-day	2525305	down	GO:0016020	0,048918805	membrane
Δfst4	12-day	2541638	down	GO:0016020	0,048918805	membrane
Δfst4	12-day	2550918	down	GO:0016020	0,048918805	membrane
Δfst4	12-day	2600584	down	GO:0016020	0,048918805	membrane
Δfst4	12-day	2610450	down	GO:0016020	0,048918805	membrane
Δfst4	12-day	2613255	down	GO:0016020	0,048918805	membrane
Δfst4	12-day	2616189	down	GO:0016020	0,048918805	membrane
Δfst4	12-day	2616602	down	GO:0016020	0,048918805	membrane
Δfst4	12-day	2617458	down	GO:0016020	0,048918805	membrane
Δfst4	12-day	2618895	down	GO:0016020	0,048918805	membrane
Δfst4	12-day	2618950	down	GO:0016020	0,048918805	membrane
Δfst4	12-day	2622595	down	GO:0016020	0,048918805	membrane
Δfst4	12-day	2622598	down	GO:0016020	0,048918805	membrane

Δfst4	12-day	2627703	down	GO:0016020	0,048918805	membrane
Δfst4	12-day	2627998	down	GO:0016020	0,048918805	membrane
Δfst4	12-day	2629091	down	GO:0016020	0,048918805	membrane
Δfst4	12-day	2629948	down	GO:0016020	0,048918805	membrane
Δfst4	12-day	2631182	down	GO:0016020	0,048918805	membrane
Δfst4	12-day	2631590	down	GO:0016020	0,048918805	membrane
Δfst4	12-day	2638261	down	GO:0016020	0,048918805	membrane
Δfst4	12-day	2640999	down	GO:0016020	0,048918805	membrane
Δfst4	12-day	2642553	down	GO:0016020	0,048918805	membrane
Δfst4	12-day	2663372	down	GO:0016020	0,048918805	membrane
Δfst4	12-day	2670534	down	GO:0016020	0,048918805	membrane
Δfst4	12-day	2675646	down	GO:0016020	0,048918805	membrane
Δfst4	12-day	2701571	down	GO:0016020	0,048918805	membrane
Δgat1	12-day	2335105	up	GO:0004308	0,029344967	exo-alpha-sialidase activity
Δgat1	12-day	2629381	up	GO:0004308	0,029344967	exo-alpha-sialidase activity
Δgat1	12-day	2619340	up	GO:0004194	0,048447606	pepsin A activity
Δgat1	12-day	2520592	up	GO:0004339	0,048447606	glucan 1,4-alpha-glucosidase activity
Δgat1	12-day	2683413	down	GO:0005840	2,35E-19	ribosome
Δgat1	12-day	2689752	down	GO:0005840	2,35E-19	ribosome
Δgat1	12-day	2683413	down	GO:0003735	3,26E-19	structural constituent of ribosome
Δgat1	12-day	2689752	down	GO:0003735	3,26E-19	structural constituent of ribosome
Δgat1	12-day	2620969	down	GO:0006412	1,08E-17	translation
Δgat1	12-day	2683413	down	GO:0006412	1,08E-17	translation
Δgat1	12-day	2689752	down	GO:0006412	1,08E-17	translation
Δgat1	12-day	1163017	down	GO:0005622	5,40E-07	intracellular
Δgat1	12-day	1168422	down	GO:0005622	5,40E-07	intracellular
Δgat1	12-day	2511560	down	GO:0005622	5,40E-07	intracellular
Δgat1	12-day	2520583	down	GO:0005622	5,40E-07	intracellular
Δgat1	12-day	2526629	down	GO:0005622	5,40E-07	intracellular
Δgat1	12-day	2533198	down	GO:0005622	5,40E-07	intracellular
Δgat1	12-day	2545061	down	GO:0005622	5,40E-07	intracellular
Δgat1	12-day	2563894	down	GO:0005622	5,40E-07	intracellular
Δgat1	12-day	2594118	down	GO:0005622	5,40E-07	intracellular
Δgat1	12-day	2604883	down	GO:0005622	5,40E-07	intracellular

Δgat1	12-day	2606711	down	GO:0005622	5,40E-07	intracellular
Δgat1	12-day	2624237	down	GO:0005622	5,40E-07	intracellular
Δgat1	12-day	2629777	down	GO:0005622	5,40E-07	intracellular
Δgat1	12-day	2667924	down	GO:0005622	5,40E-07	intracellular
Δgat1	12-day	2668143	down	GO:0005622	5,40E-07	intracellular
Δgat1	12-day	2683413	down	GO:0005622	5,40E-07	intracellular
Δgat1	12-day	2686265	down	GO:0005622	5,40E-07	intracellular
Δgat1	12-day	2687474	down	GO:0005622	5,40E-07	intracellular
Δgat1	12-day	2689752	down	GO:0005622	5,40E-07	intracellular
Δgat1	12-day	2690968	down	GO:0005622	5,40E-07	intracellular
Δgat1	12-day	2743676	down	GO:0005622	5,40E-07	intracellular
Δgat1	12-day	2075136	down	GO:0006118	0,000245849	electron transport
Δgat1	12-day	2501258	down	GO:0006118	0,000245849	electron transport
Δgat1	12-day	2503108	down	GO:0006118	0,000245849	electron transport
Δgat1	12-day	2516208	down	GO:0006118	0,000245849	electron transport
Δgat1	12-day	2527674	down	GO:0006118	0,000245849	electron transport
Δgat1	12-day	2537529	down	GO:0006118	0,000245849	electron transport
Δgat1	12-day	2594205	down	GO:0006118	0,000245849	electron transport
Δgat1	12-day	2603449	down	GO:0006118	0,000245849	electron transport
Δgat1	12-day	2620407	down	GO:0006118	0,000245849	electron transport
Δgat1	12-day	2620726	down	GO:0006118	0,000245849	electron transport
Δgat1	12-day	2643965	down	GO:0006118	0,000245849	electron transport
Δgat1	12-day	2666440	down	GO:0006118	0,000245849	electron transport
Δgat1	12-day	2695849	down	GO:0006118	0,000245849	electron transport
Δgat1	12-day	2703927	down	GO:0006118	0,000245849	electron transport
Δgat1	12-day	2075136	down	GO:0005506	0,000363471	iron ion binding
Δgat1	12-day	2501258	down	GO:0005506	0,000363471	iron ion binding
Δgat1	12-day	2503108	down	GO:0005506	0,000363471	iron ion binding
Δgat1	12-day	2516208	down	GO:0005506	0,000363471	iron ion binding
Δgat1	12-day	2537529	down	GO:0005506	0,000363471	iron ion binding
Δgat1	12-day	2603449	down	GO:0005506	0,000363471	iron ion binding
Δgat1	12-day	2618220	down	GO:0005506	0,000363471	iron ion binding
Δgat1	12-day	2620407	down	GO:0005506	0,000363471	iron ion binding
Δgat1	12-day	2622144	down	GO:0005506	0,000363471	iron ion binding

Δgat1	12-day	2643965	down	GO:0005506	0,000363471	iron ion binding
Δgat1	12-day	2666440	down	GO:0005506	0,000363471	iron ion binding
Δgat1	12-day	2695849	down	GO:0005506	0,000363471	iron ion binding
Δgat1	12-day	2075136	down	GO:0020037	0,000363471	heme binding
Δgat1	12-day	2501258	down	GO:0020037	0,000363471	heme binding
Δgat1	12-day	2516208	down	GO:0020037	0,000363471	heme binding
Δgat1	12-day	2537529	down	GO:0020037	0,000363471	heme binding
Δgat1	12-day	2620407	down	GO:0020037	0,000363471	heme binding
Δgat1	12-day	2666440	down	GO:0020037	0,000363471	heme binding
Δgat1	12-day	2695849	down	GO:0020037	0,000363471	heme binding
Δgat1	12-day	2501258	down	GO:0004497	0,00037448	monooxygenase activity
Δgat1	12-day	2516208	down	GO:0004497	0,00037448	monooxygenase activity
Δgat1	12-day	2537529	down	GO:0004497	0,00037448	monooxygenase activity
Δgat1	12-day	2620407	down	GO:0004497	0,00037448	monooxygenase activity
Δgat1	12-day	2666440	down	GO:0004497	0,00037448	monooxygenase activity
Δgat1	12-day	2695849	down	GO:0004497	0,00037448	monooxygenase activity
Δgat1	12-day	2703927	down	GO:0004497	0,00037448	monooxygenase activity
Δgat1	12-day	1214326	down	GO:0008152	0,00037448	metabolic process
Δgat1	12-day	2039576	down	GO:0008152	0,00037448	metabolic process
Δgat1	12-day	2508441	down	GO:0008152	0,00037448	metabolic process
Δgat1	12-day	2512248	down	GO:0008152	0,00037448	metabolic process
Δgat1	12-day	2579143	down	GO:0008152	0,00037448	metabolic process
Δgat1	12-day	2606988	down	GO:0008152	0,00037448	metabolic process
Δgat1	12-day	2610398	down	GO:0008152	0,00037448	metabolic process
Δgat1	12-day	2613213	down	GO:0008152	0,00037448	metabolic process
Δgat1	12-day	2614800	down	GO:0008152	0,00037448	metabolic process
Δgat1	12-day	2623038	down	GO:0008152	0,00037448	metabolic process
Δgat1	12-day	2643306	down	GO:0008152	0,00037448	metabolic process
Δgat1	12-day	2670535	down	GO:0008152	0,00037448	metabolic process
Δgat1	12-day	2681649	down	GO:0008152	0,00037448	metabolic process
Δgat1	12-day	2683413	down	GO:0008152	0,00037448	metabolic process
Δgat1	12-day	2703927	down	GO:0008152	0,00037448	metabolic process
Δgat1	12-day	2705669	down	GO:0008152	0,00037448	metabolic process
Δgat1	12-day	2629420	down	GO:0005199	0,00037448	structural constituent of cell wall

Δgat1	12-day	2703935	down	GO:0005199	0,00037448	structural constituent of cell wall
Δgat1	12-day	2629420	down	GO:0005618	0,000446778	cell wall
Δgat1	12-day	2703935	down	GO:0005618	0,000446778	cell wall
Δgat1	12-day	1160309	down	GO:0016491	0,000852952	oxidoreductase activity
Δgat1	12-day	1174574	down	GO:0016491	0,000852952	oxidoreductase activity
Δgat1	12-day	1214326	down	GO:0016491	0,000852952	oxidoreductase activity
Δgat1	12-day	2039576	down	GO:0016491	0,000852952	oxidoreductase activity
Δgat1	12-day	2503108	down	GO:0016491	0,000852952	oxidoreductase activity
Δgat1	12-day	2527674	down	GO:0016491	0,000852952	oxidoreductase activity
Δgat1	12-day	2593711	down	GO:0016491	0,000852952	oxidoreductase activity
Δgat1	12-day	2603449	down	GO:0016491	0,000852952	oxidoreductase activity
Δgat1	12-day	2606988	down	GO:0016491	0,000852952	oxidoreductase activity
Δgat1	12-day	2618220	down	GO:0016491	0,000852952	oxidoreductase activity
Δgat1	12-day	2622144	down	GO:0016491	0,000852952	oxidoreductase activity
Δgat1	12-day	2643306	down	GO:0016491	0,000852952	oxidoreductase activity
Δgat1	12-day	2643965	down	GO:0016491	0,000852952	oxidoreductase activity
Δgat1	12-day	2681649	down	GO:0016491	0,000852952	oxidoreductase activity
Δgat1	12-day	2685505	down	GO:0016491	0,000852952	oxidoreductase activity
Δgat1	12-day	2703927	down	GO:0016491	0,000852952	oxidoreductase activity
Δgat1	12-day	1097910	down	GO:0003824	0,001914723	catalytic activity
Δgat1	12-day	1160309	down	GO:0003824	0,001914723	catalytic activity
Δgat1	12-day	1354877	down	GO:0003824	0,001914723	catalytic activity
Δgat1	12-day	2501021	down	GO:0003824	0,001914723	catalytic activity
Δgat1	12-day	2557656	down	GO:0003824	0,001914723	catalytic activity
Δgat1	12-day	2568997	down	GO:0003824	0,001914723	catalytic activity
Δgat1	12-day	2570936	down	GO:0003824	0,001914723	catalytic activity
Δgat1	12-day	2598145	down	GO:0003824	0,001914723	catalytic activity
Δgat1	12-day	2606988	down	GO:0003824	0,001914723	catalytic activity
Δgat1	12-day	2613213	down	GO:0003824	0,001914723	catalytic activity
Δgat1	12-day	2626643	down	GO:0003824	0,001914723	catalytic activity
Δgat1	12-day	2628008	down	GO:0003824	0,001914723	catalytic activity
Δgat1	12-day	2643306	down	GO:0003824	0,001914723	catalytic activity
Δgat1	12-day	2643776	down	GO:0003824	0,001914723	catalytic activity
Δgat1	12-day	2645588	down	GO:0003824	0,001914723	catalytic activity

Δgat1	12-day	2670535	down	GO:0003824	0,001914723	catalytic activity
Δgat1	12-day	2681649	down	GO:0003824	0,001914723	catalytic activity
Δgat1	12-day	2683413	down	GO:0003824	0,001914723	catalytic activity
Δgat1	12-day	2699217	down	GO:0003824	0,001914723	catalytic activity
Δgat1	12-day	1165990	down	GO:0030170	0,003409341	pyridoxal phosphate binding
Δgat1	12-day	2579143	down	GO:0030170	0,003409341	pyridoxal phosphate binding
Δgat1	12-day	2610398	down	GO:0030170	0,003409341	pyridoxal phosphate binding
Δgat1	12-day	2620407	down	GO:0050381	0,004644759	unspecific monooxygenase activity
Δgat1	12-day	2666440	down	GO:0050381	0,004644759	unspecific monooxygenase activity
Δgat1	12-day	73246	down	GO:0005975	0,010410984	carbohydrate metabolic process
Δgat1	12-day	2501021	down	GO:0005975	0,010410984	carbohydrate metabolic process
Δgat1	12-day	2538080	down	GO:0005975	0,010410984	carbohydrate metabolic process
Δgat1	12-day	2557656	down	GO:0005975	0,010410984	carbohydrate metabolic process
Δgat1	12-day	2570936	down	GO:0005975	0,010410984	carbohydrate metabolic process
Δgat1	12-day	2613657	down	GO:0005975	0,010410984	carbohydrate metabolic process
Δgat1	12-day	2621806	down	GO:0005975	0,010410984	carbohydrate metabolic process
Δgat1	12-day	2628008	down	GO:0005975	0,010410984	carbohydrate metabolic process
Δgat1	12-day	2645945	down	GO:0005975	0,010410984	carbohydrate metabolic process
Δgat1	12-day	2686544	down	GO:0005975	0,010410984	carbohydrate metabolic process
Δgat1	12-day	2620969	down	GO:0006418	0,016476396	tRNA aminoacylation for protein translation
Δgat1	12-day	2620969	down	GO:0005737	0,026640154	cytoplasm
Δgat1	12-day	2645217	down	GO:0005737	0,026640154	cytoplasm
Δgat1	12-day	2607525	down	GO:0006511	0,026640154	ubiquitin-dependent protein catabolic process
Δgat1	12-day	2620969	down	GO:0004812	0,030671141	aminoacyl-tRNA ligase activity
Δhom1	12-day	1097910	down	GO:0003824	0,000112704	catalytic activity
Δhom1	12-day	1160309	down	GO:0003824	0,000112704	catalytic activity
Δhom1	12-day	2557656	down	GO:0003824	0,000112704	catalytic activity
Δhom1	12-day	2570936	down	GO:0003824	0,000112704	catalytic activity
Δhom1	12-day	2598145	down	GO:0003824	0,000112704	catalytic activity
Δhom1	12-day	2613213	down	GO:0003824	0,000112704	catalytic activity
Δhom1	12-day	2626643	down	GO:0003824	0,000112704	catalytic activity
Δhom1	12-day	2631112	down	GO:0003824	0,000112704	catalytic activity
Δhom1	12-day	2645588	down	GO:0003824	0,000112704	catalytic activity
Δhom1	12-day	2698029	down	GO:0003824	0,000112704	catalytic activity

Δhom1	12-day	1160309	down	GO:0016491	0,00026419	oxidoreductase activity
Δhom1	12-day	1174574	down	GO:0016491	0,00026419	oxidoreductase activity
Δhom1	12-day	2481224	down	GO:0016491	0,00026419	oxidoreductase activity
Δhom1	12-day	2618220	down	GO:0016491	0,00026419	oxidoreductase activity
Δhom1	12-day	2508441	down	GO:0008152	0,002845935	metabolic process
Δhom1	12-day	2613213	down	GO:0008152	0,002845935	metabolic process
Δhom1	12-day	2631112	down	GO:0008152	0,002845935	metabolic process
Δhom1	12-day	2698029	down	GO:0008152	0,002845935	metabolic process
Δhom1	12-day	2705669	down	GO:0008152	0,002845935	metabolic process
Δhom1	12-day	1091573	down	GO:0016021	0,007675283	integral to membrane
Δhom1	12-day	1136736	down	GO:0016021	0,007675283	integral to membrane
Δhom1	12-day	1179721	down	GO:0016021	0,007675283	integral to membrane
Δhom1	12-day	2491032	down	GO:0016021	0,007675283	integral to membrane
Δhom1	12-day	2493556	down	GO:0016021	0,007675283	integral to membrane
Δhom1	12-day	2499364	down	GO:0016021	0,007675283	integral to membrane
Δhom1	12-day	2563487	down	GO:0016021	0,007675283	integral to membrane
Δhom1	12-day	2566280	down	GO:0016021	0,007675283	integral to membrane
Δhom1	12-day	2607662	down	GO:0016021	0,007675283	integral to membrane
Δhom1	12-day	2625385	down	GO:0016021	0,007675283	integral to membrane
Δhom1	12-day	2626581	down	GO:0016021	0,007675283	integral to membrane
Δhom1	12-day	2642967	down	GO:0016021	0,007675283	integral to membrane
Δhom1	12-day	2695671	down	GO:0016021	0,007675283	integral to membrane
Δhom1	12-day	1136736	down	GO:0005215	0,020827849	transporter activity
Δhom1	12-day	1179721	down	GO:0005215	0,020827849	transporter activity
Δhom1	12-day	2499364	down	GO:0005215	0,020827849	transporter activity
Δhom1	12-day	2607662	down	GO:0005215	0,020827849	transporter activity
Δhom1	12-day	2538080	down	GO:0005975	0,031693201	carbohydrate metabolic process
Δhom1	12-day	2557656	down	GO:0005975	0,031693201	carbohydrate metabolic process
Δhom1	12-day	2570936	down	GO:0005975	0,031693201	carbohydrate metabolic process
Δhom1	12-day	2645945	down	GO:0005975	0,031693201	carbohydrate metabolic process
Δhom2	12-day	2515885	up	GO:0003735	4,60E-31	structural constituent of ribosome
Δhom2	12-day	2644288	up	GO:0003735	4,60E-31	structural constituent of ribosome
Δhom2	12-day	2689752	up	GO:0003735	4,60E-31	structural constituent of ribosome
Δhom2	12-day	2515885	up	GO:0005840	5,28E-30	ribosome

Δhom2	12-day	2644288	up	GO:0005840	5,28E-30	ribosome
Δhom2	12-day	2689752	up	GO:0005840	5,28E-30	ribosome
Δhom2	12-day	1196424	up	GO:0006412	1,40E-21	translation
Δhom2	12-day	2515885	up	GO:0006412	1,40E-21	translation
Δhom2	12-day	2620969	up	GO:0006412	1,40E-21	translation
Δhom2	12-day	2644288	up	GO:0006412	1,40E-21	translation
Δhom2	12-day	2689752	up	GO:0006412	1,40E-21	translation
Δhom2	12-day	2730106	up	GO:0006412	1,40E-21	translation
Δhom2	12-day	66483	up	GO:0005975	1,47E-10	carbohydrate metabolic process
Δhom2	12-day	85210	up	GO:0005975	1,47E-10	carbohydrate metabolic process
Δhom2	12-day	234329	up	GO:0005975	1,47E-10	carbohydrate metabolic process
Δhom2	12-day	2486953	up	GO:0005975	1,47E-10	carbohydrate metabolic process
Δhom2	12-day	2493241	up	GO:0005975	1,47E-10	carbohydrate metabolic process
Δhom2	12-day	2501021	up	GO:0005975	1,47E-10	carbohydrate metabolic process
Δhom2	12-day	2514546	up	GO:0005975	1,47E-10	carbohydrate metabolic process
Δhom2	12-day	2570936	up	GO:0005975	1,47E-10	carbohydrate metabolic process
Δhom2	12-day	2611511	up	GO:0005975	1,47E-10	carbohydrate metabolic process
Δhom2	12-day	2613657	up	GO:0005975	1,47E-10	carbohydrate metabolic process
Δhom2	12-day	2621806	up	GO:0005975	1,47E-10	carbohydrate metabolic process
Δhom2	12-day	2622563	up	GO:0005975	1,47E-10	carbohydrate metabolic process
Δhom2	12-day	2624588	up	GO:0005975	1,47E-10	carbohydrate metabolic process
Δhom2	12-day	2624823	up	GO:0005975	1,47E-10	carbohydrate metabolic process
Δhom2	12-day	2626756	up	GO:0005975	1,47E-10	carbohydrate metabolic process
Δhom2	12-day	2631634	up	GO:0005975	1,47E-10	carbohydrate metabolic process
Δhom2	12-day	2633791	up	GO:0005975	1,47E-10	carbohydrate metabolic process
Δhom2	12-day	2641020	up	GO:0005975	1,47E-10	carbohydrate metabolic process
Δhom2	12-day	2642958	up	GO:0005975	1,47E-10	carbohydrate metabolic process
Δhom2	12-day	2643740	up	GO:0005975	1,47E-10	carbohydrate metabolic process
Δhom2	12-day	2644666	up	GO:0005975	1,47E-10	carbohydrate metabolic process
Δhom2	12-day	2645822	up	GO:0005975	1,47E-10	carbohydrate metabolic process
Δhom2	12-day	2670422	up	GO:0005975	1,47E-10	carbohydrate metabolic process
Δhom2	12-day	2676633	up	GO:0005975	1,47E-10	carbohydrate metabolic process
Δhom2	12-day	2686544	up	GO:0005975	1,47E-10	carbohydrate metabolic process
Δhom2	12-day	1083109	up	GO:0005506	5,62E-10	iron ion binding

Δhom2	12-day	1120318	up	GO:0005506	5,62E-10	iron ion binding
Δhom2	12-day	1147397	up	GO:0005506	5,62E-10	iron ion binding
Δhom2	12-day	1173144	up	GO:0005506	5,62E-10	iron ion binding
Δhom2	12-day	2364606	up	GO:0005506	5,62E-10	iron ion binding
Δhom2	12-day	2491624	up	GO:0005506	5,62E-10	iron ion binding
Δhom2	12-day	2501258	up	GO:0005506	5,62E-10	iron ion binding
Δhom2	12-day	2514636	up	GO:0005506	5,62E-10	iron ion binding
Δhom2	12-day	2516208	up	GO:0005506	5,62E-10	iron ion binding
Δhom2	12-day	2517480	up	GO:0005506	5,62E-10	iron ion binding
Δhom2	12-day	2537529	up	GO:0005506	5,62E-10	iron ion binding
Δhom2	12-day	2559987	up	GO:0005506	5,62E-10	iron ion binding
Δhom2	12-day	2616590	up	GO:0005506	5,62E-10	iron ion binding
Δhom2	12-day	2625023	up	GO:0005506	5,62E-10	iron ion binding
Δhom2	12-day	2626841	up	GO:0005506	5,62E-10	iron ion binding
Δhom2	12-day	2628300	up	GO:0005506	5,62E-10	iron ion binding
Δhom2	12-day	2629308	up	GO:0005506	5,62E-10	iron ion binding
Δhom2	12-day	2633776	up	GO:0005506	5,62E-10	iron ion binding
Δhom2	12-day	2635949	up	GO:0005506	5,62E-10	iron ion binding
Δhom2	12-day	2637467	up	GO:0005506	5,62E-10	iron ion binding
Δhom2	12-day	2638282	up	GO:0005506	5,62E-10	iron ion binding
Δhom2	12-day	2638350	up	GO:0005506	5,62E-10	iron ion binding
Δhom2	12-day	2638355	up	GO:0005506	5,62E-10	iron ion binding
Δhom2	12-day	2640550	up	GO:0005506	5,62E-10	iron ion binding
Δhom2	12-day	2666440	up	GO:0005506	5,62E-10	iron ion binding
Δhom2	12-day	2695849	up	GO:0005506	5,62E-10	iron ion binding
Δhom2	12-day	2703965	up	GO:0005506	5,62E-10	iron ion binding
Δhom2	12-day	1083109	up	GO:0020037	1,12E-09	heme binding
Δhom2	12-day	1120318	up	GO:0020037	1,12E-09	heme binding
Δhom2	12-day	1147397	up	GO:0020037	1,12E-09	heme binding
Δhom2	12-day	1166490	up	GO:0020037	1,12E-09	heme binding
Δhom2	12-day	1173144	up	GO:0020037	1,12E-09	heme binding
Δhom2	12-day	2364606	up	GO:0020037	1,12E-09	heme binding
Δhom2	12-day	2491624	up	GO:0020037	1,12E-09	heme binding
Δhom2	12-day	2501258	up	GO:0020037	1,12E-09	heme binding

Δhom2	12-day	2514636	up	GO:0020037	1,12E-09	heme binding
Δhom2	12-day	2516208	up	GO:0020037	1,12E-09	heme binding
Δhom2	12-day	2517480	up	GO:0020037	1,12E-09	heme binding
Δhom2	12-day	2537529	up	GO:0020037	1,12E-09	heme binding
Δhom2	12-day	2559987	up	GO:0020037	1,12E-09	heme binding
Δhom2	12-day	2583399	up	GO:0020037	1,12E-09	heme binding
Δhom2	12-day	2608091	up	GO:0020037	1,12E-09	heme binding
Δhom2	12-day	2616590	up	GO:0020037	1,12E-09	heme binding
Δhom2	12-day	2625023	up	GO:0020037	1,12E-09	heme binding
Δhom2	12-day	2626841	up	GO:0020037	1,12E-09	heme binding
Δhom2	12-day	2628300	up	GO:0020037	1,12E-09	heme binding
Δhom2	12-day	2629308	up	GO:0020037	1,12E-09	heme binding
Δhom2	12-day	2633776	up	GO:0020037	1,12E-09	heme binding
Δhom2	12-day	2635949	up	GO:0020037	1,12E-09	heme binding
Δhom2	12-day	2637467	up	GO:0020037	1,12E-09	heme binding
Δhom2	12-day	2638282	up	GO:0020037	1,12E-09	heme binding
Δhom2	12-day	2638350	up	GO:0020037	1,12E-09	heme binding
Δhom2	12-day	2638355	up	GO:0020037	1,12E-09	heme binding
Δhom2	12-day	2640550	up	GO:0020037	1,12E-09	heme binding
Δhom2	12-day	2640585	up	GO:0020037	1,12E-09	heme binding
Δhom2	12-day	2643274	up	GO:0020037	1,12E-09	heme binding
Δhom2	12-day	2666440	up	GO:0020037	1,12E-09	heme binding
Δhom2	12-day	2673121	up	GO:0020037	1,12E-09	heme binding
Δhom2	12-day	2695849	up	GO:0020037	1,12E-09	heme binding
Δhom2	12-day	2703965	up	GO:0020037	1,12E-09	heme binding
Δhom2	12-day	1120318	up	GO:0006118	1,74E-08	electron transport
Δhom2	12-day	1147397	up	GO:0006118	1,74E-08	electron transport
Δhom2	12-day	1166490	up	GO:0006118	1,74E-08	electron transport
Δhom2	12-day	1168026	up	GO:0006118	1,74E-08	electron transport
Δhom2	12-day	1173144	up	GO:0006118	1,74E-08	electron transport
Δhom2	12-day	1342670	up	GO:0006118	1,74E-08	electron transport
Δhom2	12-day	2213880	up	GO:0006118	1,74E-08	electron transport
Δhom2	12-day	2364606	up	GO:0006118	1,74E-08	electron transport
Δhom2	12-day	2490194	up	GO:0006118	1,74E-08	electron transport

Δhom2	12-day	2491624	up	GO:0006118	1,74E-08	electron transport
Δhom2	12-day	2501258	up	GO:0006118	1,74E-08	electron transport
Δhom2	12-day	2505277	up	GO:0006118	1,74E-08	electron transport
Δhom2	12-day	2507628	up	GO:0006118	1,74E-08	electron transport
Δhom2	12-day	2514636	up	GO:0006118	1,74E-08	electron transport
Δhom2	12-day	2515251	up	GO:0006118	1,74E-08	electron transport
Δhom2	12-day	2516208	up	GO:0006118	1,74E-08	electron transport
Δhom2	12-day	2517480	up	GO:0006118	1,74E-08	electron transport
Δhom2	12-day	2537529	up	GO:0006118	1,74E-08	electron transport
Δhom2	12-day	2559987	up	GO:0006118	1,74E-08	electron transport
Δhom2	12-day	2573393	up	GO:0006118	1,74E-08	electron transport
Δhom2	12-day	2573396	up	GO:0006118	1,74E-08	electron transport
Δhom2	12-day	2576903	up	GO:0006118	1,74E-08	electron transport
Δhom2	12-day	2583399	up	GO:0006118	1,74E-08	electron transport
Δhom2	12-day	2594205	up	GO:0006118	1,74E-08	electron transport
Δhom2	12-day	2605889	up	GO:0006118	1,74E-08	electron transport
Δhom2	12-day	2615184	up	GO:0006118	1,74E-08	electron transport
Δhom2	12-day	2616557	up	GO:0006118	1,74E-08	electron transport
Δhom2	12-day	2616590	up	GO:0006118	1,74E-08	electron transport
Δhom2	12-day	2622733	up	GO:0006118	1,74E-08	electron transport
Δhom2	12-day	2622740	up	GO:0006118	1,74E-08	electron transport
Δhom2	12-day	2625023	up	GO:0006118	1,74E-08	electron transport
Δhom2	12-day	2625730	up	GO:0006118	1,74E-08	electron transport
Δhom2	12-day	2626841	up	GO:0006118	1,74E-08	electron transport
Δhom2	12-day	2628120	up	GO:0006118	1,74E-08	electron transport
Δhom2	12-day	2628300	up	GO:0006118	1,74E-08	electron transport
Δhom2	12-day	2629308	up	GO:0006118	1,74E-08	electron transport
Δhom2	12-day	2633776	up	GO:0006118	1,74E-08	electron transport
Δhom2	12-day	2635949	up	GO:0006118	1,74E-08	electron transport
Δhom2	12-day	2637467	up	GO:0006118	1,74E-08	electron transport
Δhom2	12-day	2638282	up	GO:0006118	1,74E-08	electron transport
Δhom2	12-day	2638350	up	GO:0006118	1,74E-08	electron transport
Δhom2	12-day	2638355	up	GO:0006118	1,74E-08	electron transport
Δhom2	12-day	2638473	up	GO:0006118	1,74E-08	electron transport

Δhom2	12-day	2640550	up	GO:0006118	1,74E-08	electron transport
Δhom2	12-day	2643274	up	GO:0006118	1,74E-08	electron transport
Δhom2	12-day	2645843	up	GO:0006118	1,74E-08	electron transport
Δhom2	12-day	2666009	up	GO:0006118	1,74E-08	electron transport
Δhom2	12-day	2666440	up	GO:0006118	1,74E-08	electron transport
Δhom2	12-day	2667289	up	GO:0006118	1,74E-08	electron transport
Δhom2	12-day	2695849	up	GO:0006118	1,74E-08	electron transport
Δhom2	12-day	2703927	up	GO:0006118	1,74E-08	electron transport
Δhom2	12-day	2703965	up	GO:0006118	1,74E-08	electron transport
Δhom2	12-day	2750685	up	GO:0006118	1,74E-08	electron transport
Δhom2	12-day	1194000	up	GO:0005622	1,74E-08	intracellular
Δhom2	12-day	1293032	up	GO:0005622	1,74E-08	intracellular
Δhom2	12-day	2509453	up	GO:0005622	1,74E-08	intracellular
Δhom2	12-day	2511582	up	GO:0005622	1,74E-08	intracellular
Δhom2	12-day	2513276	up	GO:0005622	1,74E-08	intracellular
Δhom2	12-day	2515885	up	GO:0005622	1,74E-08	intracellular
Δhom2	12-day	2520583	up	GO:0005622	1,74E-08	intracellular
Δhom2	12-day	2563894	up	GO:0005622	1,74E-08	intracellular
Δhom2	12-day	2570476	up	GO:0005622	1,74E-08	intracellular
Δhom2	12-day	2580737	up	GO:0005622	1,74E-08	intracellular
Δhom2	12-day	2594118	up	GO:0005622	1,74E-08	intracellular
Δhom2	12-day	2601101	up	GO:0005622	1,74E-08	intracellular
Δhom2	12-day	2604369	up	GO:0005622	1,74E-08	intracellular
Δhom2	12-day	2608014	up	GO:0005622	1,74E-08	intracellular
Δhom2	12-day	2609438	up	GO:0005622	1,74E-08	intracellular
Δhom2	12-day	2615107	up	GO:0005622	1,74E-08	intracellular
Δhom2	12-day	2621972	up	GO:0005622	1,74E-08	intracellular
Δhom2	12-day	2624237	up	GO:0005622	1,74E-08	intracellular
Δhom2	12-day	2630358	up	GO:0005622	1,74E-08	intracellular
Δhom2	12-day	2635886	up	GO:0005622	1,74E-08	intracellular
Δhom2	12-day	2643497	up	GO:0005622	1,74E-08	intracellular
Δhom2	12-day	2644288	up	GO:0005622	1,74E-08	intracellular
Δhom2	12-day	2666500	up	GO:0005622	1,74E-08	intracellular
Δhom2	12-day	2667924	up	GO:0005622	1,74E-08	intracellular

Δhom2	12-day	2689752	up	GO:0005622	1,74E-08	intracellular
Δhom2	12-day	2703923	up	GO:0005622	1,74E-08	intracellular
Δhom2	12-day	2737267	up	GO:0005622	1,74E-08	intracellular
Δhom2	12-day	85210	up	GO:0004553	1,83E-08	hydrolase activity, hydrolyzing O-glycosyl compounds
Δhom2	12-day	234329	up	GO:0004553	1,83E-08	hydrolase activity, hydrolyzing O-glycosyl compounds
Δhom2	12-day	2486953	up	GO:0004553	1,83E-08	hydrolase activity, hydrolyzing O-glycosyl compounds
Δhom2	12-day	2611511	up	GO:0004553	1,83E-08	hydrolase activity, hydrolyzing O-glycosyl compounds
Δhom2	12-day	2613657	up	GO:0004553	1,83E-08	hydrolase activity, hydrolyzing O-glycosyl compounds
Δhom2	12-day	2621806	up	GO:0004553	1,83E-08	hydrolase activity, hydrolyzing O-glycosyl compounds
Δhom2	12-day	2622563	up	GO:0004553	1,83E-08	hydrolase activity, hydrolyzing O-glycosyl compounds
Δhom2	12-day	2642958	up	GO:0004553	1,83E-08	hydrolase activity, hydrolyzing O-glycosyl compounds
Δhom2	12-day	2643740	up	GO:0004553	1,83E-08	hydrolase activity, hydrolyzing O-glycosyl compounds
Δhom2	12-day	2644666	up	GO:0004553	1,83E-08	hydrolase activity, hydrolyzing O-glycosyl compounds
Δhom2	12-day	2645822	up	GO:0004553	1,83E-08	hydrolase activity, hydrolyzing O-glycosyl compounds
Δhom2	12-day	2670422	up	GO:0004553	1,83E-08	hydrolase activity, hydrolyzing O-glycosyl compounds
Δhom2	12-day	2686544	up	GO:0004553	1,83E-08	hydrolase activity, hydrolyzing O-glycosyl compounds
Δhom2	12-day	1120318	up	GO:0004497	5,15E-08	monooxygenase activity
Δhom2	12-day	1147397	up	GO:0004497	5,15E-08	monooxygenase activity
Δhom2	12-day	1168026	up	GO:0004497	5,15E-08	monooxygenase activity
Δhom2	12-day	1173144	up	GO:0004497	5,15E-08	monooxygenase activity
Δhom2	12-day	1342670	up	GO:0004497	5,15E-08	monooxygenase activity
Δhom2	12-day	2213880	up	GO:0004497	5,15E-08	monooxygenase activity
Δhom2	12-day	2364606	up	GO:0004497	5,15E-08	monooxygenase activity
Δhom2	12-day	2490194	up	GO:0004497	5,15E-08	monooxygenase activity
Δhom2	12-day	2491624	up	GO:0004497	5,15E-08	monooxygenase activity
Δhom2	12-day	2501258	up	GO:0004497	5,15E-08	monooxygenase activity
Δhom2	12-day	2514636	up	GO:0004497	5,15E-08	monooxygenase activity
Δhom2	12-day	2516208	up	GO:0004497	5,15E-08	monooxygenase activity
Δhom2	12-day	2517480	up	GO:0004497	5,15E-08	monooxygenase activity
Δhom2	12-day	2537529	up	GO:0004497	5,15E-08	monooxygenase activity
Δhom2	12-day	2559987	up	GO:0004497	5,15E-08	monooxygenase activity
Δhom2	12-day	2573393	up	GO:0004497	5,15E-08	monooxygenase activity
Δhom2	12-day	2573396	up	GO:0004497	5,15E-08	monooxygenase activity
Δhom2	12-day	2616590	up	GO:0004497	5,15E-08	monooxygenase activity

Δhom2	12-day	2625023	up	GO:0004497	5,15E-08	monooxygenase activity
Δhom2	12-day	2626841	up	GO:0004497	5,15E-08	monooxygenase activity
Δhom2	12-day	2628300	up	GO:0004497	5,15E-08	monooxygenase activity
Δhom2	12-day	2629308	up	GO:0004497	5,15E-08	monooxygenase activity
Δhom2	12-day	2633776	up	GO:0004497	5,15E-08	monooxygenase activity
Δhom2	12-day	2635949	up	GO:0004497	5,15E-08	monooxygenase activity
Δhom2	12-day	2637467	up	GO:0004497	5,15E-08	monooxygenase activity
Δhom2	12-day	2638282	up	GO:0004497	5,15E-08	monooxygenase activity
Δhom2	12-day	2638350	up	GO:0004497	5,15E-08	monooxygenase activity
Δhom2	12-day	2638355	up	GO:0004497	5,15E-08	monooxygenase activity
Δhom2	12-day	2640550	up	GO:0004497	5,15E-08	monooxygenase activity
Δhom2	12-day	2666440	up	GO:0004497	5,15E-08	monooxygenase activity
Δhom2	12-day	2695849	up	GO:0004497	5,15E-08	monooxygenase activity
Δhom2	12-day	2703927	up	GO:0004497	5,15E-08	monooxygenase activity
Δhom2	12-day	2703965	up	GO:0004497	5,15E-08	monooxygenase activity
Δhom2	12-day	2750685	up	GO:0004497	5,15E-08	monooxygenase activity
Δhom2	12-day	1120318	up	GO:0050381	2,55E-07	unspecific monooxygenase activity
Δhom2	12-day	2491624	up	GO:0050381	2,55E-07	unspecific monooxygenase activity
Δhom2	12-day	2514636	up	GO:0050381	2,55E-07	unspecific monooxygenase activity
Δhom2	12-day	2559987	up	GO:0050381	2,55E-07	unspecific monooxygenase activity
Δhom2	12-day	2625023	up	GO:0050381	2,55E-07	unspecific monooxygenase activity
Δhom2	12-day	2633776	up	GO:0050381	2,55E-07	unspecific monooxygenase activity
Δhom2	12-day	2637467	up	GO:0050381	2,55E-07	unspecific monooxygenase activity
Δhom2	12-day	2638350	up	GO:0050381	2,55E-07	unspecific monooxygenase activity
Δhom2	12-day	2638355	up	GO:0050381	2,55E-07	unspecific monooxygenase activity
Δhom2	12-day	2666440	up	GO:0050381	2,55E-07	unspecific monooxygenase activity
Δhom2	12-day	2703965	up	GO:0050381	2,55E-07	unspecific monooxygenase activity
Δhom2	12-day	1111500	up	GO:0005215	0,002394941	transporter activity
Δhom2	12-day	1136736	up	GO:0005215	0,002394941	transporter activity
Δhom2	12-day	1147635	up	GO:0005215	0,002394941	transporter activity
Δhom2	12-day	1196424	up	GO:0005215	0,002394941	transporter activity
Δhom2	12-day	1234296	up	GO:0005215	0,002394941	transporter activity
Δhom2	12-day	2145491	up	GO:0005215	0,002394941	transporter activity
Δhom2	12-day	2481323	up	GO:0005215	0,002394941	transporter activity

Δhom2	12-day	2487072	up	GO:0005215	0,002394941	transporter activity
Δhom2	12-day	2488479	up	GO:0005215	0,002394941	transporter activity
Δhom2	12-day	2490525	up	GO:0005215	0,002394941	transporter activity
Δhom2	12-day	2499364	up	GO:0005215	0,002394941	transporter activity
Δhom2	12-day	2499677	up	GO:0005215	0,002394941	transporter activity
Δhom2	12-day	2501978	up	GO:0005215	0,002394941	transporter activity
Δhom2	12-day	2502284	up	GO:0005215	0,002394941	transporter activity
Δhom2	12-day	2508421	up	GO:0005215	0,002394941	transporter activity
Δhom2	12-day	2515031	up	GO:0005215	0,002394941	transporter activity
Δhom2	12-day	2533765	up	GO:0005215	0,002394941	transporter activity
Δhom2	12-day	2546372	up	GO:0005215	0,002394941	transporter activity
Δhom2	12-day	2550918	up	GO:0005215	0,002394941	transporter activity
Δhom2	12-day	2564524	up	GO:0005215	0,002394941	transporter activity
Δhom2	12-day	2611816	up	GO:0005215	0,002394941	transporter activity
Δhom2	12-day	2612313	up	GO:0005215	0,002394941	transporter activity
Δhom2	12-day	2617636	up	GO:0005215	0,002394941	transporter activity
Δhom2	12-day	2618842	up	GO:0005215	0,002394941	transporter activity
Δhom2	12-day	2619010	up	GO:0005215	0,002394941	transporter activity
Δhom2	12-day	2623043	up	GO:0005215	0,002394941	transporter activity
Δhom2	12-day	2623613	up	GO:0005215	0,002394941	transporter activity
Δhom2	12-day	2625268	up	GO:0005215	0,002394941	transporter activity
Δhom2	12-day	2627824	up	GO:0005215	0,002394941	transporter activity
Δhom2	12-day	2627883	up	GO:0005215	0,002394941	transporter activity
Δhom2	12-day	2628098	up	GO:0005215	0,002394941	transporter activity
Δhom2	12-day	2628982	up	GO:0005215	0,002394941	transporter activity
Δhom2	12-day	2630257	up	GO:0005215	0,002394941	transporter activity
Δhom2	12-day	2630802	up	GO:0005215	0,002394941	transporter activity
Δhom2	12-day	2631348	up	GO:0005215	0,002394941	transporter activity
Δhom2	12-day	2632105	up	GO:0005215	0,002394941	transporter activity
Δhom2	12-day	2633206	up	GO:0005215	0,002394941	transporter activity
Δhom2	12-day	2633212	up	GO:0005215	0,002394941	transporter activity
Δhom2	12-day	2633216	up	GO:0005215	0,002394941	transporter activity
Δhom2	12-day	2633596	up	GO:0005215	0,002394941	transporter activity
Δhom2	12-day	2633874	up	GO:0005215	0,002394941	transporter activity

Δhom2	12-day	2637652	up	GO:0005215	0,002394941	transporter activity
Δhom2	12-day	2641735	up	GO:0005215	0,002394941	transporter activity
Δhom2	12-day	2642894	up	GO:0005215	0,002394941	transporter activity
Δhom2	12-day	2660764	up	GO:0005215	0,002394941	transporter activity
Δhom2	12-day	2663452	up	GO:0005215	0,002394941	transporter activity
Δhom2	12-day	2680674	up	GO:0005215	0,002394941	transporter activity
Δhom2	12-day	2698170	up	GO:0005215	0,002394941	transporter activity
Δhom2	12-day	2698544	up	GO:0005215	0,002394941	transporter activity
Δhom2	12-day	2704067	up	GO:0005215	0,002394941	transporter activity
Δhom2	12-day	2738153	up	GO:0005215	0,002394941	transporter activity
Δhom2	12-day	2749899	up	GO:0005215	0,002394941	transporter activity
Δhom2	12-day	2621224	up	GO:0006071	0,007460932	glycerol metabolic process
Δhom2	12-day	48375	up	GO:0003824	0,01579114	catalytic activity
Δhom2	12-day	48473	up	GO:0003824	0,01579114	catalytic activity
Δhom2	12-day	62301	up	GO:0003824	0,01579114	catalytic activity
Δhom2	12-day	75401	up	GO:0003824	0,01579114	catalytic activity
Δhom2	12-day	1097910	up	GO:0003824	0,01579114	catalytic activity
Δhom2	12-day	1110309	up	GO:0003824	0,01579114	catalytic activity
Δhom2	12-day	1131413	up	GO:0003824	0,01579114	catalytic activity
Δhom2	12-day	1143694	up	GO:0003824	0,01579114	catalytic activity
Δhom2	12-day	1147022	up	GO:0003824	0,01579114	catalytic activity
Δhom2	12-day	1150509	up	GO:0003824	0,01579114	catalytic activity
Δhom2	12-day	1158760	up	GO:0003824	0,01579114	catalytic activity
Δhom2	12-day	1160309	up	GO:0003824	0,01579114	catalytic activity
Δhom2	12-day	1212612	up	GO:0003824	0,01579114	catalytic activity
Δhom2	12-day	2006874	up	GO:0003824	0,01579114	catalytic activity
Δhom2	12-day	2052819	up	GO:0003824	0,01579114	catalytic activity
Δhom2	12-day	2190164	up	GO:0003824	0,01579114	catalytic activity
Δhom2	12-day	2485902	up	GO:0003824	0,01579114	catalytic activity
Δhom2	12-day	2491365	up	GO:0003824	0,01579114	catalytic activity
Δhom2	12-day	2499736	up	GO:0003824	0,01579114	catalytic activity
Δhom2	12-day	2501021	up	GO:0003824	0,01579114	catalytic activity
Δhom2	12-day	2502008	up	GO:0003824	0,01579114	catalytic activity
Δhom2	12-day	2506635	up	GO:0003824	0,01579114	catalytic activity

Δhom2	12-day	2509648	up	GO:0003824	0,01579114	catalytic activity
Δhom2	12-day	2513478	up	GO:0003824	0,01579114	catalytic activity
Δhom2	12-day	2514546	up	GO:0003824	0,01579114	catalytic activity
Δhom2	12-day	2515044	up	GO:0003824	0,01579114	catalytic activity
Δhom2	12-day	2516959	up	GO:0003824	0,01579114	catalytic activity
Δhom2	12-day	2525914	up	GO:0003824	0,01579114	catalytic activity
Δhom2	12-day	2536732	up	GO:0003824	0,01579114	catalytic activity
Δhom2	12-day	2540875	up	GO:0003824	0,01579114	catalytic activity
Δhom2	12-day	2543052	up	GO:0003824	0,01579114	catalytic activity
Δhom2	12-day	2556971	up	GO:0003824	0,01579114	catalytic activity
Δhom2	12-day	2568997	up	GO:0003824	0,01579114	catalytic activity
Δhom2	12-day	2570476	up	GO:0003824	0,01579114	catalytic activity
Δhom2	12-day	2570936	up	GO:0003824	0,01579114	catalytic activity
Δhom2	12-day	2570968	up	GO:0003824	0,01579114	catalytic activity
Δhom2	12-day	2580436	up	GO:0003824	0,01579114	catalytic activity
Δhom2	12-day	2592175	up	GO:0003824	0,01579114	catalytic activity
Δhom2	12-day	2594114	up	GO:0003824	0,01579114	catalytic activity
Δhom2	12-day	2603999	up	GO:0003824	0,01579114	catalytic activity
Δhom2	12-day	2605850	up	GO:0003824	0,01579114	catalytic activity
Δhom2	12-day	2606113	up	GO:0003824	0,01579114	catalytic activity
Δhom2	12-day	2606333	up	GO:0003824	0,01579114	catalytic activity
Δhom2	12-day	2606680	up	GO:0003824	0,01579114	catalytic activity
Δhom2	12-day	2606982	up	GO:0003824	0,01579114	catalytic activity
Δhom2	12-day	2607741	up	GO:0003824	0,01579114	catalytic activity
Δhom2	12-day	2607760	up	GO:0003824	0,01579114	catalytic activity
Δhom2	12-day	2608014	up	GO:0003824	0,01579114	catalytic activity
Δhom2	12-day	2609281	up	GO:0003824	0,01579114	catalytic activity
Δhom2	12-day	2609923	up	GO:0003824	0,01579114	catalytic activity
Δhom2	12-day	2610555	up	GO:0003824	0,01579114	catalytic activity
Δhom2	12-day	2611066	up	GO:0003824	0,01579114	catalytic activity
Δhom2	12-day	2612608	up	GO:0003824	0,01579114	catalytic activity
Δhom2	12-day	2613213	up	GO:0003824	0,01579114	catalytic activity
Δhom2	12-day	2613634	up	GO:0003824	0,01579114	catalytic activity
Δhom2	12-day	2614269	up	GO:0003824	0,01579114	catalytic activity

Δhom2	12-day	2614455	up	GO:0003824	0,01579114	catalytic activity
Δhom2	12-day	2614716	up	GO:0003824	0,01579114	catalytic activity
Δhom2	12-day	2616512	up	GO:0003824	0,01579114	catalytic activity
Δhom2	12-day	2616873	up	GO:0003824	0,01579114	catalytic activity
Δhom2	12-day	2617245	up	GO:0003824	0,01579114	catalytic activity
Δhom2	12-day	2617669	up	GO:0003824	0,01579114	catalytic activity
Δhom2	12-day	2618038	up	GO:0003824	0,01579114	catalytic activity
Δhom2	12-day	2618228	up	GO:0003824	0,01579114	catalytic activity
Δhom2	12-day	2619775	up	GO:0003824	0,01579114	catalytic activity
Δhom2	12-day	2620112	up	GO:0003824	0,01579114	catalytic activity
Δhom2	12-day	2620314	up	GO:0003824	0,01579114	catalytic activity
Δhom2	12-day	2620801	up	GO:0003824	0,01579114	catalytic activity
Δhom2	12-day	2622563	up	GO:0003824	0,01579114	catalytic activity
Δhom2	12-day	2623663	up	GO:0003824	0,01579114	catalytic activity
Δhom2	12-day	2623957	up	GO:0003824	0,01579114	catalytic activity
Δhom2	12-day	2624672	up	GO:0003824	0,01579114	catalytic activity
Δhom2	12-day	2626155	up	GO:0003824	0,01579114	catalytic activity
Δhom2	12-day	2626643	up	GO:0003824	0,01579114	catalytic activity
Δhom2	12-day	2626749	up	GO:0003824	0,01579114	catalytic activity
Δhom2	12-day	2626756	up	GO:0003824	0,01579114	catalytic activity
Δhom2	12-day	2627113	up	GO:0003824	0,01579114	catalytic activity
Δhom2	12-day	2628120	up	GO:0003824	0,01579114	catalytic activity
Δhom2	12-day	2629514	up	GO:0003824	0,01579114	catalytic activity
Δhom2	12-day	2630740	up	GO:0003824	0,01579114	catalytic activity
Δhom2	12-day	2631112	up	GO:0003824	0,01579114	catalytic activity
Δhom2	12-day	2631634	up	GO:0003824	0,01579114	catalytic activity
Δhom2	12-day	2632193	up	GO:0003824	0,01579114	catalytic activity
Δhom2	12-day	2634217	up	GO:0003824	0,01579114	catalytic activity
Δhom2	12-day	2634281	up	GO:0003824	0,01579114	catalytic activity
Δhom2	12-day	2636220	up	GO:0003824	0,01579114	catalytic activity
Δhom2	12-day	2636221	up	GO:0003824	0,01579114	catalytic activity
Δhom2	12-day	2636417	up	GO:0003824	0,01579114	catalytic activity
Δhom2	12-day	2636705	up	GO:0003824	0,01579114	catalytic activity
Δhom2	12-day	2637838	up	GO:0003824	0,01579114	catalytic activity

Δhom2	12-day	2638445	up	GO:0003824	0,01579114	catalytic activity
Δhom2	12-day	2638446	up	GO:0003824	0,01579114	catalytic activity
Δhom2	12-day	2638893	up	GO:0003824	0,01579114	catalytic activity
Δhom2	12-day	2640123	up	GO:0003824	0,01579114	catalytic activity
Δhom2	12-day	2641020	up	GO:0003824	0,01579114	catalytic activity
Δhom2	12-day	2641553	up	GO:0003824	0,01579114	catalytic activity
Δhom2	12-day	2642124	up	GO:0003824	0,01579114	catalytic activity
Δhom2	12-day	2642810	up	GO:0003824	0,01579114	catalytic activity
Δhom2	12-day	2643306	up	GO:0003824	0,01579114	catalytic activity
Δhom2	12-day	2643776	up	GO:0003824	0,01579114	catalytic activity
Δhom2	12-day	2644309	up	GO:0003824	0,01579114	catalytic activity
Δhom2	12-day	2644333	up	GO:0003824	0,01579114	catalytic activity
Δhom2	12-day	2644610	up	GO:0003824	0,01579114	catalytic activity
Δhom2	12-day	2661978	up	GO:0003824	0,01579114	catalytic activity
Δhom2	12-day	2670535	up	GO:0003824	0,01579114	catalytic activity
Δhom2	12-day	2670536	up	GO:0003824	0,01579114	catalytic activity
Δhom2	12-day	2676633	up	GO:0003824	0,01579114	catalytic activity
Δhom2	12-day	2681649	up	GO:0003824	0,01579114	catalytic activity
Δhom2	12-day	2682272	up	GO:0003824	0,01579114	catalytic activity
Δhom2	12-day	2684249	up	GO:0003824	0,01579114	catalytic activity
Δhom2	12-day	2689521	up	GO:0003824	0,01579114	catalytic activity
Δhom2	12-day	2690641	up	GO:0003824	0,01579114	catalytic activity
Δhom2	12-day	2694468	up	GO:0003824	0,01579114	catalytic activity
Δhom2	12-day	2698029	up	GO:0003824	0,01579114	catalytic activity
Δhom2	12-day	2698778	up	GO:0003824	0,01579114	catalytic activity
Δhom2	12-day	2737267	up	GO:0003824	0,01579114	catalytic activity
Δhom2	12-day	2748659	up	GO:0003824	0,01579114	catalytic activity
Δhom2	12-day	2750120	up	GO:0003824	0,01579114	catalytic activity
Δhom2	12-day	2750165	up	GO:0003824	0,01579114	catalytic activity
Δhom2	12-day	75401	up	GO:0005515	0,03955685	protein binding
Δhom2	12-day	1174869	up	GO:0005515	0,03955685	protein binding
Δhom2	12-day	1215992	up	GO:0005515	0,03955685	protein binding
Δhom2	12-day	2335105	up	GO:0005515	0,03955685	protein binding
Δhom2	12-day	2485674	up	GO:0005515	0,03955685	protein binding

Δhom2	12-day	2488527	up	GO:0005515	0,03955685	protein binding
Δhom2	12-day	2495727	up	GO:0005515	0,03955685	protein binding
Δhom2	12-day	2533371	up	GO:0005515	0,03955685	protein binding
Δhom2	12-day	2535716	up	GO:0005515	0,03955685	protein binding
Δhom2	12-day	2550939	up	GO:0005515	0,03955685	protein binding
Δhom2	12-day	2563016	up	GO:0005515	0,03955685	protein binding
Δhom2	12-day	2563200	up	GO:0005515	0,03955685	protein binding
Δhom2	12-day	2568997	up	GO:0005515	0,03955685	protein binding
Δhom2	12-day	2585708	up	GO:0005515	0,03955685	protein binding
Δhom2	12-day	2589194	up	GO:0005515	0,03955685	protein binding
Δhom2	12-day	2604369	up	GO:0005515	0,03955685	protein binding
Δhom2	12-day	2605152	up	GO:0005515	0,03955685	protein binding
Δhom2	12-day	2608117	up	GO:0005515	0,03955685	protein binding
Δhom2	12-day	2610180	up	GO:0005515	0,03955685	protein binding
Δhom2	12-day	2610321	up	GO:0005515	0,03955685	protein binding
Δhom2	12-day	2614036	up	GO:0005515	0,03955685	protein binding
Δhom2	12-day	2614667	up	GO:0005515	0,03955685	protein binding
Δhom2	12-day	2615956	up	GO:0005515	0,03955685	protein binding
Δhom2	12-day	2630911	up	GO:0005515	0,03955685	protein binding
Δhom2	12-day	2631130	up	GO:0005515	0,03955685	protein binding
Δhom2	12-day	2636606	up	GO:0005515	0,03955685	protein binding
Δhom2	12-day	2644666	up	GO:0005515	0,03955685	protein binding
Δhom2	12-day	2669239	up	GO:0005515	0,03955685	protein binding
Δhom2	12-day	2684588	up	GO:0005515	0,03955685	protein binding
Δhom2	12-day	2710797	up	GO:0005515	0,03955685	protein binding
Δhom2	12-day	1147635	up	GO:0005351	0,048490429	sugar:hydrogen symporter activity
Δhom2	12-day	2499677	up	GO:0005351	0,048490429	sugar:hydrogen symporter activity
Δhom2	12-day	2515031	up	GO:0005351	0,048490429	sugar:hydrogen symporter activity
Δhom2	12-day	2550918	up	GO:0005351	0,048490429	sugar:hydrogen symporter activity
Δhom2	12-day	2610180	up	GO:0005351	0,048490429	sugar:hydrogen symporter activity
Δhom2	12-day	2619010	up	GO:0005351	0,048490429	sugar:hydrogen symporter activity
Δhom2	12-day	2623043	up	GO:0005351	0,048490429	sugar:hydrogen symporter activity
Δhom2	12-day	2627824	up	GO:0005351	0,048490429	sugar:hydrogen symporter activity
Δhom2	12-day	2627883	up	GO:0005351	0,048490429	sugar:hydrogen symporter activity

Δhom2	12-day	2704067	up	GO:0005351	0,048490429	sugar:hydrogen symporter activity
Δhom2	12-day	2749899	up	GO:0005351	0,048490429	sugar:hydrogen symporter activity
Δhom2	12-day	57429	up	GO:0005524	0,048490429	ATP binding
Δhom2	12-day	1129062	up	GO:0005524	0,048490429	ATP binding
Δhom2	12-day	1158800	up	GO:0005524	0,048490429	ATP binding
Δhom2	12-day	1172108	up	GO:0005524	0,048490429	ATP binding
Δhom2	12-day	1174869	up	GO:0005524	0,048490429	ATP binding
Δhom2	12-day	1178759	up	GO:0005524	0,048490429	ATP binding
Δhom2	12-day	1181112	up	GO:0005524	0,048490429	ATP binding
Δhom2	12-day	1186310	up	GO:0005524	0,048490429	ATP binding
Δhom2	12-day	1196424	up	GO:0005524	0,048490429	ATP binding
Δhom2	12-day	2253810	up	GO:0005524	0,048490429	ATP binding
Δhom2	12-day	2279823	up	GO:0005524	0,048490429	ATP binding
Δhom2	12-day	2481145	up	GO:0005524	0,048490429	ATP binding
Δhom2	12-day	2484841	up	GO:0005524	0,048490429	ATP binding
Δhom2	12-day	2486797	up	GO:0005524	0,048490429	ATP binding
Δhom2	12-day	2487176	up	GO:0005524	0,048490429	ATP binding
Δhom2	12-day	2490823	up	GO:0005524	0,048490429	ATP binding
Δhom2	12-day	2493556	up	GO:0005524	0,048490429	ATP binding
Δhom2	12-day	2495301	up	GO:0005524	0,048490429	ATP binding
Δhom2	12-day	2503832	up	GO:0005524	0,048490429	ATP binding
Δhom2	12-day	2510275	up	GO:0005524	0,048490429	ATP binding
Δhom2	12-day	2511561	up	GO:0005524	0,048490429	ATP binding
Δhom2	12-day	2514780	up	GO:0005524	0,048490429	ATP binding
Δhom2	12-day	2520046	up	GO:0005524	0,048490429	ATP binding
Δhom2	12-day	2527752	up	GO:0005524	0,048490429	ATP binding
Δhom2	12-day	2530387	up	GO:0005524	0,048490429	ATP binding
Δhom2	12-day	2533371	up	GO:0005524	0,048490429	ATP binding
Δhom2	12-day	2535079	up	GO:0005524	0,048490429	ATP binding
Δhom2	12-day	2535527	up	GO:0005524	0,048490429	ATP binding
Δhom2	12-day	2544151	up	GO:0005524	0,048490429	ATP binding
Δhom2	12-day	2550266	up	GO:0005524	0,048490429	ATP binding
Δhom2	12-day	2550939	up	GO:0005524	0,048490429	ATP binding
Δhom2	12-day	2554917	up	GO:0005524	0,048490429	ATP binding

Δhom2	12-day	2563653	up	GO:0005524	0,048490429	ATP binding
Δhom2	12-day	2565471	up	GO:0005524	0,048490429	ATP binding
Δhom2	12-day	2569377	up	GO:0005524	0,048490429	ATP binding
Δhom2	12-day	2585708	up	GO:0005524	0,048490429	ATP binding
Δhom2	12-day	2604369	up	GO:0005524	0,048490429	ATP binding
Δhom2	12-day	2605230	up	GO:0005524	0,048490429	ATP binding
Δhom2	12-day	2606202	up	GO:0005524	0,048490429	ATP binding
Δhom2	12-day	2610180	up	GO:0005524	0,048490429	ATP binding
Δhom2	12-day	2612206	up	GO:0005524	0,048490429	ATP binding
Δhom2	12-day	2612608	up	GO:0005524	0,048490429	ATP binding
Δhom2	12-day	2614625	up	GO:0005524	0,048490429	ATP binding
Δhom2	12-day	2614761	up	GO:0005524	0,048490429	ATP binding
Δhom2	12-day	2620364	up	GO:0005524	0,048490429	ATP binding
Δhom2	12-day	2620969	up	GO:0005524	0,048490429	ATP binding
Δhom2	12-day	2621533	up	GO:0005524	0,048490429	ATP binding
Δhom2	12-day	2621741	up	GO:0005524	0,048490429	ATP binding
Δhom2	12-day	2622087	up	GO:0005524	0,048490429	ATP binding
Δhom2	12-day	2623685	up	GO:0005524	0,048490429	ATP binding
Δhom2	12-day	2629497	up	GO:0005524	0,048490429	ATP binding
Δhom2	12-day	2632313	up	GO:0005524	0,048490429	ATP binding
Δhom2	12-day	2632374	up	GO:0005524	0,048490429	ATP binding
Δhom2	12-day	2632524	up	GO:0005524	0,048490429	ATP binding
Δhom2	12-day	2633620	up	GO:0005524	0,048490429	ATP binding
Δhom2	12-day	2636368	up	GO:0005524	0,048490429	ATP binding
Δhom2	12-day	2638986	up	GO:0005524	0,048490429	ATP binding
Δhom2	12-day	2641678	up	GO:0005524	0,048490429	ATP binding
Δhom2	12-day	2645675	up	GO:0005524	0,048490429	ATP binding
Δhom2	12-day	2661304	up	GO:0005524	0,048490429	ATP binding
Δhom2	12-day	2662880	up	GO:0005524	0,048490429	ATP binding
Δhom2	12-day	2672807	up	GO:0005524	0,048490429	ATP binding
Δhom2	12-day	2675859	up	GO:0005524	0,048490429	ATP binding
Δhom2	12-day	2684163	up	GO:0005524	0,048490429	ATP binding
Δhom2	12-day	2691288	up	GO:0005524	0,048490429	ATP binding
Δhom2	12-day	2693043	up	GO:0005524	0,048490429	ATP binding

Δhom2	12-day	2693940	up	GO:0005524	0,048490429	ATP binding
Δhom2	12-day	2706463	up	GO:0005524	0,048490429	ATP binding
Δhom2	12-day	2725125	up	GO:0005524	0,048490429	ATP binding
Δhom2	12-day	1078940	down	GO:0006810	6,33E-14	transport
Δhom2	12-day	1144263	down	GO:0006810	6,33E-14	transport
Δhom2	12-day	1153136	down	GO:0006810	6,33E-14	transport
Δhom2	12-day	1188719	down	GO:0006810	6,33E-14	transport
Δhom2	12-day	1267603	down	GO:0006810	6,33E-14	transport
Δhom2	12-day	2122325	down	GO:0006810	6,33E-14	transport
Δhom2	12-day	2162840	down	GO:0006810	6,33E-14	transport
Δhom2	12-day	2481653	down	GO:0006810	6,33E-14	transport
Δhom2	12-day	2488637	down	GO:0006810	6,33E-14	transport
Δhom2	12-day	2492878	down	GO:0006810	6,33E-14	transport
Δhom2	12-day	2494144	down	GO:0006810	6,33E-14	transport
Δhom2	12-day	2494727	down	GO:0006810	6,33E-14	transport
Δhom2	12-day	2504863	down	GO:0006810	6,33E-14	transport
Δhom2	12-day	2509716	down	GO:0006810	6,33E-14	transport
Δhom2	12-day	2516374	down	GO:0006810	6,33E-14	transport
Δhom2	12-day	2520877	down	GO:0006810	6,33E-14	transport
Δhom2	12-day	2525305	down	GO:0006810	6,33E-14	transport
Δhom2	12-day	2607025	down	GO:0006810	6,33E-14	transport
Δhom2	12-day	2607891	down	GO:0006810	6,33E-14	transport
Δhom2	12-day	2612474	down	GO:0006810	6,33E-14	transport
Δhom2	12-day	2613255	down	GO:0006810	6,33E-14	transport
Δhom2	12-day	2616961	down	GO:0006810	6,33E-14	transport
Δhom2	12-day	2619645	down	GO:0006810	6,33E-14	transport
Δhom2	12-day	2620170	down	GO:0006810	6,33E-14	transport
Δhom2	12-day	2620830	down	GO:0006810	6,33E-14	transport
Δhom2	12-day	2622598	down	GO:0006810	6,33E-14	transport
Δhom2	12-day	2622931	down	GO:0006810	6,33E-14	transport
Δhom2	12-day	2623492	down	GO:0006810	6,33E-14	transport
Δhom2	12-day	2626803	down	GO:0006810	6,33E-14	transport
Δhom2	12-day	2627190	down	GO:0006810	6,33E-14	transport
Δhom2	12-day	2627703	down	GO:0006810	6,33E-14	transport

Δhom2	12-day	2628751	down	GO:0006810	6,33E-14	transport
Δhom2	12-day	2629091	down	GO:0006810	6,33E-14	transport
Δhom2	12-day	2629455	down	GO:0006810	6,33E-14	transport
Δhom2	12-day	2636597	down	GO:0006810	6,33E-14	transport
Δhom2	12-day	2637190	down	GO:0006810	6,33E-14	transport
Δhom2	12-day	2637509	down	GO:0006810	6,33E-14	transport
Δhom2	12-day	2645850	down	GO:0006810	6,33E-14	transport
Δhom2	12-day	2660934	down	GO:0006810	6,33E-14	transport
Δhom2	12-day	2662585	down	GO:0006810	6,33E-14	transport
Δhom2	12-day	2675831	down	GO:0006810	6,33E-14	transport
Δhom2	12-day	2681288	down	GO:0006810	6,33E-14	transport
Δhom2	12-day	2701571	down	GO:0006810	6,33E-14	transport
Δhom2	12-day	2712060	down	GO:0006810	6,33E-14	transport
Δhom2	12-day	1144263	down	GO:0005215	1,33E-08	transporter activity
Δhom2	12-day	1153136	down	GO:0005215	1,33E-08	transporter activity
Δhom2	12-day	1188719	down	GO:0005215	1,33E-08	transporter activity
Δhom2	12-day	2122325	down	GO:0005215	1,33E-08	transporter activity
Δhom2	12-day	2162840	down	GO:0005215	1,33E-08	transporter activity
Δhom2	12-day	2481653	down	GO:0005215	1,33E-08	transporter activity
Δhom2	12-day	2488637	down	GO:0005215	1,33E-08	transporter activity
Δhom2	12-day	2492878	down	GO:0005215	1,33E-08	transporter activity
Δhom2	12-day	2494144	down	GO:0005215	1,33E-08	transporter activity
Δhom2	12-day	2504863	down	GO:0005215	1,33E-08	transporter activity
Δhom2	12-day	2509716	down	GO:0005215	1,33E-08	transporter activity
Δhom2	12-day	2516374	down	GO:0005215	1,33E-08	transporter activity
Δhom2	12-day	2520877	down	GO:0005215	1,33E-08	transporter activity
Δhom2	12-day	2525305	down	GO:0005215	1,33E-08	transporter activity
Δhom2	12-day	2607025	down	GO:0005215	1,33E-08	transporter activity
Δhom2	12-day	2607891	down	GO:0005215	1,33E-08	transporter activity
Δhom2	12-day	2612474	down	GO:0005215	1,33E-08	transporter activity
Δhom2	12-day	2613255	down	GO:0005215	1,33E-08	transporter activity
Δhom2	12-day	2616961	down	GO:0005215	1,33E-08	transporter activity
Δhom2	12-day	2619645	down	GO:0005215	1,33E-08	transporter activity
Δhom2	12-day	2620170	down	GO:0005215	1,33E-08	transporter activity

Δhom2	12-day	2620830	down	GO:0005215	1,33E-08	transporter activity
Δhom2	12-day	2622598	down	GO:0005215	1,33E-08	transporter activity
Δhom2	12-day	2622931	down	GO:0005215	1,33E-08	transporter activity
Δhom2	12-day	2623492	down	GO:0005215	1,33E-08	transporter activity
Δhom2	12-day	2626803	down	GO:0005215	1,33E-08	transporter activity
Δhom2	12-day	2627703	down	GO:0005215	1,33E-08	transporter activity
Δhom2	12-day	2628751	down	GO:0005215	1,33E-08	transporter activity
Δhom2	12-day	2629091	down	GO:0005215	1,33E-08	transporter activity
Δhom2	12-day	2629455	down	GO:0005215	1,33E-08	transporter activity
Δhom2	12-day	2636597	down	GO:0005215	1,33E-08	transporter activity
Δhom2	12-day	2637509	down	GO:0005215	1,33E-08	transporter activity
Δhom2	12-day	2660934	down	GO:0005215	1,33E-08	transporter activity
Δhom2	12-day	2701571	down	GO:0005215	1,33E-08	transporter activity
Δhom2	12-day	2712060	down	GO:0005215	1,33E-08	transporter activity
Δhom2	12-day	67604	down	GO:0016020	7,65E-07	membrane
Δhom2	12-day	1121912	down	GO:0016020	7,65E-07	membrane
Δhom2	12-day	1144263	down	GO:0016020	7,65E-07	membrane
Δhom2	12-day	1153136	down	GO:0016020	7,65E-07	membrane
Δhom2	12-day	1176319	down	GO:0016020	7,65E-07	membrane
Δhom2	12-day	1267603	down	GO:0016020	7,65E-07	membrane
Δhom2	12-day	2162840	down	GO:0016020	7,65E-07	membrane
Δhom2	12-day	2234784	down	GO:0016020	7,65E-07	membrane
Δhom2	12-day	2376858	down	GO:0016020	7,65E-07	membrane
Δhom2	12-day	2494727	down	GO:0016020	7,65E-07	membrane
Δhom2	12-day	2516374	down	GO:0016020	7,65E-07	membrane
Δhom2	12-day	2525305	down	GO:0016020	7,65E-07	membrane
Δhom2	12-day	2600584	down	GO:0016020	7,65E-07	membrane
Δhom2	12-day	2605277	down	GO:0016020	7,65E-07	membrane
Δhom2	12-day	2607008	down	GO:0016020	7,65E-07	membrane
Δhom2	12-day	2610450	down	GO:0016020	7,65E-07	membrane
Δhom2	12-day	2612474	down	GO:0016020	7,65E-07	membrane
Δhom2	12-day	2612886	down	GO:0016020	7,65E-07	membrane
Δhom2	12-day	2613255	down	GO:0016020	7,65E-07	membrane
Δhom2	12-day	2618425	down	GO:0016020	7,65E-07	membrane

Δhom2	12-day	2618479	down	GO:0016020	7,65E-07	membrane
Δhom2	12-day	2618895	down	GO:0016020	7,65E-07	membrane
Δhom2	12-day	2622595	down	GO:0016020	7,65E-07	membrane
Δhom2	12-day	2622598	down	GO:0016020	7,65E-07	membrane
Δhom2	12-day	2626051	down	GO:0016020	7,65E-07	membrane
Δhom2	12-day	2627703	down	GO:0016020	7,65E-07	membrane
Δhom2	12-day	2629091	down	GO:0016020	7,65E-07	membrane
Δhom2	12-day	2629744	down	GO:0016020	7,65E-07	membrane
Δhom2	12-day	2631182	down	GO:0016020	7,65E-07	membrane
Δhom2	12-day	2631590	down	GO:0016020	7,65E-07	membrane
Δhom2	12-day	2636160	down	GO:0016020	7,65E-07	membrane
Δhom2	12-day	2639525	down	GO:0016020	7,65E-07	membrane
Δhom2	12-day	2639805	down	GO:0016020	7,65E-07	membrane
Δhom2	12-day	2640999	down	GO:0016020	7,65E-07	membrane
Δhom2	12-day	2644280	down	GO:0016020	7,65E-07	membrane
Δhom2	12-day	2645850	down	GO:0016020	7,65E-07	membrane
Δhom2	12-day	2663372	down	GO:0016020	7,65E-07	membrane
Δhom2	12-day	2675831	down	GO:0016020	7,65E-07	membrane
Δhom2	12-day	2686995	down	GO:0016020	7,65E-07	membrane
Δhom2	12-day	2701571	down	GO:0016020	7,65E-07	membrane
Δhom2	12-day	237513	down	GO:0016491	2,45E-06	oxidoreductase activity
Δhom2	12-day	1152293	down	GO:0016491	2,45E-06	oxidoreductase activity
Δhom2	12-day	1156481	down	GO:0016491	2,45E-06	oxidoreductase activity
Δhom2	12-day	2255031	down	GO:0016491	2,45E-06	oxidoreductase activity
Δhom2	12-day	2312801	down	GO:0016491	2,45E-06	oxidoreductase activity
Δhom2	12-day	2333133	down	GO:0016491	2,45E-06	oxidoreductase activity
Δhom2	12-day	2376858	down	GO:0016491	2,45E-06	oxidoreductase activity
Δhom2	12-day	2490518	down	GO:0016491	2,45E-06	oxidoreductase activity
Δhom2	12-day	2492848	down	GO:0016491	2,45E-06	oxidoreductase activity
Δhom2	12-day	2497674	down	GO:0016491	2,45E-06	oxidoreductase activity
Δhom2	12-day	2499521	down	GO:0016491	2,45E-06	oxidoreductase activity
Δhom2	12-day	2503471	down	GO:0016491	2,45E-06	oxidoreductase activity
Δhom2	12-day	2507164	down	GO:0016491	2,45E-06	oxidoreductase activity
Δhom2	12-day	2515739	down	GO:0016491	2,45E-06	oxidoreductase activity

Δhom2	12-day	2515964	down	GO:0016491	2,45E-06	oxidoreductase activity
Δhom2	12-day	2518082	down	GO:0016491	2,45E-06	oxidoreductase activity
Δhom2	12-day	2538948	down	GO:0016491	2,45E-06	oxidoreductase activity
Δhom2	12-day	2540746	down	GO:0016491	2,45E-06	oxidoreductase activity
Δhom2	12-day	2543454	down	GO:0016491	2,45E-06	oxidoreductase activity
Δhom2	12-day	2571870	down	GO:0016491	2,45E-06	oxidoreductase activity
Δhom2	12-day	2605574	down	GO:0016491	2,45E-06	oxidoreductase activity
Δhom2	12-day	2605752	down	GO:0016491	2,45E-06	oxidoreductase activity
Δhom2	12-day	2606988	down	GO:0016491	2,45E-06	oxidoreductase activity
Δhom2	12-day	2609840	down	GO:0016491	2,45E-06	oxidoreductase activity
Δhom2	12-day	2609854	down	GO:0016491	2,45E-06	oxidoreductase activity
Δhom2	12-day	2610372	down	GO:0016491	2,45E-06	oxidoreductase activity
Δhom2	12-day	2611538	down	GO:0016491	2,45E-06	oxidoreductase activity
Δhom2	12-day	2615160	down	GO:0016491	2,45E-06	oxidoreductase activity
Δhom2	12-day	2616880	down	GO:0016491	2,45E-06	oxidoreductase activity
Δhom2	12-day	2616916	down	GO:0016491	2,45E-06	oxidoreductase activity
Δhom2	12-day	2618220	down	GO:0016491	2,45E-06	oxidoreductase activity
Δhom2	12-day	2618455	down	GO:0016491	2,45E-06	oxidoreductase activity
Δhom2	12-day	2621873	down	GO:0016491	2,45E-06	oxidoreductase activity
Δhom2	12-day	2623189	down	GO:0016491	2,45E-06	oxidoreductase activity
Δhom2	12-day	2623301	down	GO:0016491	2,45E-06	oxidoreductase activity
Δhom2	12-day	2625667	down	GO:0016491	2,45E-06	oxidoreductase activity
Δhom2	12-day	2625917	down	GO:0016491	2,45E-06	oxidoreductase activity
Δhom2	12-day	2627407	down	GO:0016491	2,45E-06	oxidoreductase activity
Δhom2	12-day	2629507	down	GO:0016491	2,45E-06	oxidoreductase activity
Δhom2	12-day	2630637	down	GO:0016491	2,45E-06	oxidoreductase activity
Δhom2	12-day	2633167	down	GO:0016491	2,45E-06	oxidoreductase activity
Δhom2	12-day	2633266	down	GO:0016491	2,45E-06	oxidoreductase activity
Δhom2	12-day	2633632	down	GO:0016491	2,45E-06	oxidoreductase activity
Δhom2	12-day	2634450	down	GO:0016491	2,45E-06	oxidoreductase activity
Δhom2	12-day	2636160	down	GO:0016491	2,45E-06	oxidoreductase activity
Δhom2	12-day	2637104	down	GO:0016491	2,45E-06	oxidoreductase activity
Δhom2	12-day	2637755	down	GO:0016491	2,45E-06	oxidoreductase activity
Δhom2	12-day	2638484	down	GO:0016491	2,45E-06	oxidoreductase activity

Δhom2	12-day	2639682	down	GO:0016491	2,45E-06	oxidoreductase activity
Δhom2	12-day	2641506	down	GO:0016491	2,45E-06	oxidoreductase activity
Δhom2	12-day	2644330	down	GO:0016491	2,45E-06	oxidoreductase activity
Δhom2	12-day	2644422	down	GO:0016491	2,45E-06	oxidoreductase activity
Δhom2	12-day	2644430	down	GO:0016491	2,45E-06	oxidoreductase activity
Δhom2	12-day	2645118	down	GO:0016491	2,45E-06	oxidoreductase activity
Δhom2	12-day	2646148	down	GO:0016491	2,45E-06	oxidoreductase activity
Δhom2	12-day	2663372	down	GO:0016491	2,45E-06	oxidoreductase activity
Δhom2	12-day	2671376	down	GO:0016491	2,45E-06	oxidoreductase activity
Δhom2	12-day	2685505	down	GO:0016491	2,45E-06	oxidoreductase activity
Δhom2	12-day	2688172	down	GO:0016491	2,45E-06	oxidoreductase activity
Δhom2	12-day	2697968	down	GO:0016491	2,45E-06	oxidoreductase activity
Δhom2	12-day	2705421	down	GO:0016491	2,45E-06	oxidoreductase activity
Δhom2	12-day	73881	down	GO:0008152	2,45E-06	metabolic process
Δhom2	12-day	1092260	down	GO:0008152	2,45E-06	metabolic process
Δhom2	12-day	1134402	down	GO:0008152	2,45E-06	metabolic process
Δhom2	12-day	1152293	down	GO:0008152	2,45E-06	metabolic process
Δhom2	12-day	1156481	down	GO:0008152	2,45E-06	metabolic process
Δhom2	12-day	1200798	down	GO:0008152	2,45E-06	metabolic process
Δhom2	12-day	2194185	down	GO:0008152	2,45E-06	metabolic process
Δhom2	12-day	2255031	down	GO:0008152	2,45E-06	metabolic process
Δhom2	12-day	2333133	down	GO:0008152	2,45E-06	metabolic process
Δhom2	12-day	2373661	down	GO:0008152	2,45E-06	metabolic process
Δhom2	12-day	2492848	down	GO:0008152	2,45E-06	metabolic process
Δhom2	12-day	2492878	down	GO:0008152	2,45E-06	metabolic process
Δhom2	12-day	2495032	down	GO:0008152	2,45E-06	metabolic process
Δhom2	12-day	2503471	down	GO:0008152	2,45E-06	metabolic process
Δhom2	12-day	2507164	down	GO:0008152	2,45E-06	metabolic process
Δhom2	12-day	2511020	down	GO:0008152	2,45E-06	metabolic process
Δhom2	12-day	2541321	down	GO:0008152	2,45E-06	metabolic process
Δhom2	12-day	2553610	down	GO:0008152	2,45E-06	metabolic process
Δhom2	12-day	2558552	down	GO:0008152	2,45E-06	metabolic process
Δhom2	12-day	2577440	down	GO:0008152	2,45E-06	metabolic process
Δhom2	12-day	2579143	down	GO:0008152	2,45E-06	metabolic process

Δhom2	12-day	2595361	down	GO:0008152	2,45E-06	metabolic process
Δhom2	12-day	2605752	down	GO:0008152	2,45E-06	metabolic process
Δhom2	12-day	2606988	down	GO:0008152	2,45E-06	metabolic process
Δhom2	12-day	2610372	down	GO:0008152	2,45E-06	metabolic process
Δhom2	12-day	2615598	down	GO:0008152	2,45E-06	metabolic process
Δhom2	12-day	2616880	down	GO:0008152	2,45E-06	metabolic process
Δhom2	12-day	2616916	down	GO:0008152	2,45E-06	metabolic process
Δhom2	12-day	2618455	down	GO:0008152	2,45E-06	metabolic process
Δhom2	12-day	2618904	down	GO:0008152	2,45E-06	metabolic process
Δhom2	12-day	2619714	down	GO:0008152	2,45E-06	metabolic process
Δhom2	12-day	2621873	down	GO:0008152	2,45E-06	metabolic process
Δhom2	12-day	2622287	down	GO:0008152	2,45E-06	metabolic process
Δhom2	12-day	2622365	down	GO:0008152	2,45E-06	metabolic process
Δhom2	12-day	2623301	down	GO:0008152	2,45E-06	metabolic process
Δhom2	12-day	2623956	down	GO:0008152	2,45E-06	metabolic process
Δhom2	12-day	2624525	down	GO:0008152	2,45E-06	metabolic process
Δhom2	12-day	2624675	down	GO:0008152	2,45E-06	metabolic process
Δhom2	12-day	2624698	down	GO:0008152	2,45E-06	metabolic process
Δhom2	12-day	2625667	down	GO:0008152	2,45E-06	metabolic process
Δhom2	12-day	2625706	down	GO:0008152	2,45E-06	metabolic process
Δhom2	12-day	2625782	down	GO:0008152	2,45E-06	metabolic process
Δhom2	12-day	2626510	down	GO:0008152	2,45E-06	metabolic process
Δhom2	12-day	2626867	down	GO:0008152	2,45E-06	metabolic process
Δhom2	12-day	2627172	down	GO:0008152	2,45E-06	metabolic process
Δhom2	12-day	2627449	down	GO:0008152	2,45E-06	metabolic process
Δhom2	12-day	2628730	down	GO:0008152	2,45E-06	metabolic process
Δhom2	12-day	2629646	down	GO:0008152	2,45E-06	metabolic process
Δhom2	12-day	2631237	down	GO:0008152	2,45E-06	metabolic process
Δhom2	12-day	2631275	down	GO:0008152	2,45E-06	metabolic process
Δhom2	12-day	2632206	down	GO:0008152	2,45E-06	metabolic process
Δhom2	12-day	2632253	down	GO:0008152	2,45E-06	metabolic process
Δhom2	12-day	2632611	down	GO:0008152	2,45E-06	metabolic process
Δhom2	12-day	2633167	down	GO:0008152	2,45E-06	metabolic process
Δhom2	12-day	2633266	down	GO:0008152	2,45E-06	metabolic process

Δhom2	12-day	2633632	down	GO:0008152	2,45E-06	metabolic process
Δhom2	12-day	2634450	down	GO:0008152	2,45E-06	metabolic process
Δhom2	12-day	2636100	down	GO:0008152	2,45E-06	metabolic process
Δhom2	12-day	2637088	down	GO:0008152	2,45E-06	metabolic process
Δhom2	12-day	2637104	down	GO:0008152	2,45E-06	metabolic process
Δhom2	12-day	2637643	down	GO:0008152	2,45E-06	metabolic process
Δhom2	12-day	2637755	down	GO:0008152	2,45E-06	metabolic process
Δhom2	12-day	2638484	down	GO:0008152	2,45E-06	metabolic process
Δhom2	12-day	2638921	down	GO:0008152	2,45E-06	metabolic process
Δhom2	12-day	2639805	down	GO:0008152	2,45E-06	metabolic process
Δhom2	12-day	2640472	down	GO:0008152	2,45E-06	metabolic process
Δhom2	12-day	2640484	down	GO:0008152	2,45E-06	metabolic process
Δhom2	12-day	2640992	down	GO:0008152	2,45E-06	metabolic process
Δhom2	12-day	2641506	down	GO:0008152	2,45E-06	metabolic process
Δhom2	12-day	2643650	down	GO:0008152	2,45E-06	metabolic process
Δhom2	12-day	2644281	down	GO:0008152	2,45E-06	metabolic process
Δhom2	12-day	2644330	down	GO:0008152	2,45E-06	metabolic process
Δhom2	12-day	2644422	down	GO:0008152	2,45E-06	metabolic process
Δhom2	12-day	2644430	down	GO:0008152	2,45E-06	metabolic process
Δhom2	12-day	2645207	down	GO:0008152	2,45E-06	metabolic process
Δhom2	12-day	2645668	down	GO:0008152	2,45E-06	metabolic process
Δhom2	12-day	2646148	down	GO:0008152	2,45E-06	metabolic process
Δhom2	12-day	2665516	down	GO:0008152	2,45E-06	metabolic process
Δhom2	12-day	2673857	down	GO:0008152	2,45E-06	metabolic process
Δhom2	12-day	2686995	down	GO:0008152	2,45E-06	metabolic process
Δhom2	12-day	2705421	down	GO:0008152	2,45E-06	metabolic process
Δhom2	12-day	2711060	down	GO:0008152	2,45E-06	metabolic process
Δhom2	12-day	2728826	down	GO:0008152	2,45E-06	metabolic process
Δhom2	12-day	73881	down	GO:0003824	2,45E-06	catalytic activity
Δhom2	12-day	85278	down	GO:0003824	2,45E-06	catalytic activity
Δhom2	12-day	237513	down	GO:0003824	2,45E-06	catalytic activity
Δhom2	12-day	1092260	down	GO:0003824	2,45E-06	catalytic activity
Δhom2	12-day	1134402	down	GO:0003824	2,45E-06	catalytic activity
Δhom2	12-day	1152293	down	GO:0003824	2,45E-06	catalytic activity

Δhom2	12-day	1184723	down	GO:0003824	2,45E-06	catalytic activity
Δhom2	12-day	1200798	down	GO:0003824	2,45E-06	catalytic activity
Δhom2	12-day	2023525	down	GO:0003824	2,45E-06	catalytic activity
Δhom2	12-day	2107183	down	GO:0003824	2,45E-06	catalytic activity
Δhom2	12-day	2194185	down	GO:0003824	2,45E-06	catalytic activity
Δhom2	12-day	2255031	down	GO:0003824	2,45E-06	catalytic activity
Δhom2	12-day	2333133	down	GO:0003824	2,45E-06	catalytic activity
Δhom2	12-day	2335629	down	GO:0003824	2,45E-06	catalytic activity
Δhom2	12-day	2368049	down	GO:0003824	2,45E-06	catalytic activity
Δhom2	12-day	2373661	down	GO:0003824	2,45E-06	catalytic activity
Δhom2	12-day	2492848	down	GO:0003824	2,45E-06	catalytic activity
Δhom2	12-day	2492878	down	GO:0003824	2,45E-06	catalytic activity
Δhom2	12-day	2495032	down	GO:0003824	2,45E-06	catalytic activity
Δhom2	12-day	2502088	down	GO:0003824	2,45E-06	catalytic activity
Δhom2	12-day	2511020	down	GO:0003824	2,45E-06	catalytic activity
Δhom2	12-day	2540746	down	GO:0003824	2,45E-06	catalytic activity
Δhom2	12-day	2541321	down	GO:0003824	2,45E-06	catalytic activity
Δhom2	12-day	2558552	down	GO:0003824	2,45E-06	catalytic activity
Δhom2	12-day	2595361	down	GO:0003824	2,45E-06	catalytic activity
Δhom2	12-day	2598960	down	GO:0003824	2,45E-06	catalytic activity
Δhom2	12-day	2605122	down	GO:0003824	2,45E-06	catalytic activity
Δhom2	12-day	2605752	down	GO:0003824	2,45E-06	catalytic activity
Δhom2	12-day	2606988	down	GO:0003824	2,45E-06	catalytic activity
Δhom2	12-day	2607034	down	GO:0003824	2,45E-06	catalytic activity
Δhom2	12-day	2610372	down	GO:0003824	2,45E-06	catalytic activity
Δhom2	12-day	2614194	down	GO:0003824	2,45E-06	catalytic activity
Δhom2	12-day	2615598	down	GO:0003824	2,45E-06	catalytic activity
Δhom2	12-day	2616880	down	GO:0003824	2,45E-06	catalytic activity
Δhom2	12-day	2616916	down	GO:0003824	2,45E-06	catalytic activity
Δhom2	12-day	2618425	down	GO:0003824	2,45E-06	catalytic activity
Δhom2	12-day	2618479	down	GO:0003824	2,45E-06	catalytic activity
Δhom2	12-day	2618904	down	GO:0003824	2,45E-06	catalytic activity
Δhom2	12-day	2618997	down	GO:0003824	2,45E-06	catalytic activity
Δhom2	12-day	2619714	down	GO:0003824	2,45E-06	catalytic activity

Δhom2	12-day	2620388	down	GO:0003824	2,45E-06	catalytic activity
Δhom2	12-day	2621873	down	GO:0003824	2,45E-06	catalytic activity
Δhom2	12-day	2622287	down	GO:0003824	2,45E-06	catalytic activity
Δhom2	12-day	2623301	down	GO:0003824	2,45E-06	catalytic activity
Δhom2	12-day	2624675	down	GO:0003824	2,45E-06	catalytic activity
Δhom2	12-day	2624698	down	GO:0003824	2,45E-06	catalytic activity
Δhom2	12-day	2625205	down	GO:0003824	2,45E-06	catalytic activity
Δhom2	12-day	2625667	down	GO:0003824	2,45E-06	catalytic activity
Δhom2	12-day	2625782	down	GO:0003824	2,45E-06	catalytic activity
Δhom2	12-day	2625818	down	GO:0003824	2,45E-06	catalytic activity
Δhom2	12-day	2626051	down	GO:0003824	2,45E-06	catalytic activity
Δhom2	12-day	2626510	down	GO:0003824	2,45E-06	catalytic activity
Δhom2	12-day	2627449	down	GO:0003824	2,45E-06	catalytic activity
Δhom2	12-day	2628448	down	GO:0003824	2,45E-06	catalytic activity
Δhom2	12-day	2628678	down	GO:0003824	2,45E-06	catalytic activity
Δhom2	12-day	2628730	down	GO:0003824	2,45E-06	catalytic activity
Δhom2	12-day	2629646	down	GO:0003824	2,45E-06	catalytic activity
Δhom2	12-day	2631237	down	GO:0003824	2,45E-06	catalytic activity
Δhom2	12-day	2632206	down	GO:0003824	2,45E-06	catalytic activity
Δhom2	12-day	2632253	down	GO:0003824	2,45E-06	catalytic activity
Δhom2	12-day	2632611	down	GO:0003824	2,45E-06	catalytic activity
Δhom2	12-day	2633144	down	GO:0003824	2,45E-06	catalytic activity
Δhom2	12-day	2633167	down	GO:0003824	2,45E-06	catalytic activity
Δhom2	12-day	2633266	down	GO:0003824	2,45E-06	catalytic activity
Δhom2	12-day	2633632	down	GO:0003824	2,45E-06	catalytic activity
Δhom2	12-day	2634450	down	GO:0003824	2,45E-06	catalytic activity
Δhom2	12-day	2634918	down	GO:0003824	2,45E-06	catalytic activity
Δhom2	12-day	2635216	down	GO:0003824	2,45E-06	catalytic activity
Δhom2	12-day	2637088	down	GO:0003824	2,45E-06	catalytic activity
Δhom2	12-day	2637104	down	GO:0003824	2,45E-06	catalytic activity
Δhom2	12-day	2637783	down	GO:0003824	2,45E-06	catalytic activity
Δhom2	12-day	2638484	down	GO:0003824	2,45E-06	catalytic activity
Δhom2	12-day	2638783	down	GO:0003824	2,45E-06	catalytic activity
Δhom2	12-day	2640484	down	GO:0003824	2,45E-06	catalytic activity

Δhom2	12-day	2640992	down	GO:0003824	2,45E-06	catalytic activity
Δhom2	12-day	2641022	down	GO:0003824	2,45E-06	catalytic activity
Δhom2	12-day	2641506	down	GO:0003824	2,45E-06	catalytic activity
Δhom2	12-day	2643650	down	GO:0003824	2,45E-06	catalytic activity
Δhom2	12-day	2643902	down	GO:0003824	2,45E-06	catalytic activity
Δhom2	12-day	2644281	down	GO:0003824	2,45E-06	catalytic activity
Δhom2	12-day	2644330	down	GO:0003824	2,45E-06	catalytic activity
Δhom2	12-day	2645207	down	GO:0003824	2,45E-06	catalytic activity
Δhom2	12-day	2645668	down	GO:0003824	2,45E-06	catalytic activity
Δhom2	12-day	2673857	down	GO:0003824	2,45E-06	catalytic activity
Δhom2	12-day	2686995	down	GO:0003824	2,45E-06	catalytic activity
Δhom2	12-day	2688172	down	GO:0003824	2,45E-06	catalytic activity
Δhom2	12-day	2699217	down	GO:0003824	2,45E-06	catalytic activity
Δhom2	12-day	2705421	down	GO:0003824	2,45E-06	catalytic activity
Δhom2	12-day	2711060	down	GO:0003824	2,45E-06	catalytic activity
Δhom2	12-day	1036421	down	GO:0016021	2,45E-06	integral to membrane
Δhom2	12-day	1144263	down	GO:0016021	2,45E-06	integral to membrane
Δhom2	12-day	1153136	down	GO:0016021	2,45E-06	integral to membrane
Δhom2	12-day	1188719	down	GO:0016021	2,45E-06	integral to membrane
Δhom2	12-day	2122325	down	GO:0016021	2,45E-06	integral to membrane
Δhom2	12-day	2481653	down	GO:0016021	2,45E-06	integral to membrane
Δhom2	12-day	2488637	down	GO:0016021	2,45E-06	integral to membrane
Δhom2	12-day	2494144	down	GO:0016021	2,45E-06	integral to membrane
Δhom2	12-day	2504863	down	GO:0016021	2,45E-06	integral to membrane
Δhom2	12-day	2509716	down	GO:0016021	2,45E-06	integral to membrane
Δhom2	12-day	2516374	down	GO:0016021	2,45E-06	integral to membrane
Δhom2	12-day	2524870	down	GO:0016021	2,45E-06	integral to membrane
Δhom2	12-day	2525305	down	GO:0016021	2,45E-06	integral to membrane
Δhom2	12-day	2538948	down	GO:0016021	2,45E-06	integral to membrane
Δhom2	12-day	2602395	down	GO:0016021	2,45E-06	integral to membrane
Δhom2	12-day	2604984	down	GO:0016021	2,45E-06	integral to membrane
Δhom2	12-day	2607025	down	GO:0016021	2,45E-06	integral to membrane
Δhom2	12-day	2607891	down	GO:0016021	2,45E-06	integral to membrane
Δhom2	12-day	2612474	down	GO:0016021	2,45E-06	integral to membrane

Δhom2	12-day	2613255	down	GO:0016021	2,45E-06	integral to membrane
Δhom2	12-day	2616961	down	GO:0016021	2,45E-06	integral to membrane
Δhom2	12-day	2619090	down	GO:0016021	2,45E-06	integral to membrane
Δhom2	12-day	2619645	down	GO:0016021	2,45E-06	integral to membrane
Δhom2	12-day	2620170	down	GO:0016021	2,45E-06	integral to membrane
Δhom2	12-day	2620830	down	GO:0016021	2,45E-06	integral to membrane
Δhom2	12-day	2622598	down	GO:0016021	2,45E-06	integral to membrane
Δhom2	12-day	2622931	down	GO:0016021	2,45E-06	integral to membrane
Δhom2	12-day	2623492	down	GO:0016021	2,45E-06	integral to membrane
Δhom2	12-day	2626803	down	GO:0016021	2,45E-06	integral to membrane
Δhom2	12-day	2627190	down	GO:0016021	2,45E-06	integral to membrane
Δhom2	12-day	2627703	down	GO:0016021	2,45E-06	integral to membrane
Δhom2	12-day	2629091	down	GO:0016021	2,45E-06	integral to membrane
Δhom2	12-day	2629455	down	GO:0016021	2,45E-06	integral to membrane
Δhom2	12-day	2636597	down	GO:0016021	2,45E-06	integral to membrane
Δhom2	12-day	2637190	down	GO:0016021	2,45E-06	integral to membrane
Δhom2	12-day	2637509	down	GO:0016021	2,45E-06	integral to membrane
Δhom2	12-day	2641709	down	GO:0016021	2,45E-06	integral to membrane
Δhom2	12-day	2645659	down	GO:0016021	2,45E-06	integral to membrane
Δhom2	12-day	2660934	down	GO:0016021	2,45E-06	integral to membrane
Δhom2	12-day	2663372	down	GO:0016021	2,45E-06	integral to membrane
Δhom2	12-day	2681288	down	GO:0016021	2,45E-06	integral to membrane
Δhom2	12-day	2686473	down	GO:0016021	2,45E-06	integral to membrane
Δhom2	12-day	2701571	down	GO:0016021	2,45E-06	integral to membrane
Δhom2	12-day	2704867	down	GO:0016021	2,45E-06	integral to membrane
Δhom2	12-day	2712060	down	GO:0016021	2,45E-06	integral to membrane
Δhom2	12-day	2675831	down	GO:0006865	9,53E-06	amino acid transport
Δhom2	12-day	2675831	down	GO:0015171	9,53E-06	amino acid transmembrane transporter activity
Δhom2	12-day	78628	down	GO:0004497	4,29E-05	monooxygenase activity
Δhom2	12-day	81631	down	GO:0004497	4,29E-05	monooxygenase activity
Δhom2	12-day	1131542	down	GO:0004497	4,29E-05	monooxygenase activity
Δhom2	12-day	1189794	down	GO:0004497	4,29E-05	monooxygenase activity
Δhom2	12-day	1193647	down	GO:0004497	4,29E-05	monooxygenase activity
Δhom2	12-day	2327903	down	GO:0004497	4,29E-05	monooxygenase activity

Δhom2	12-day	2484868	down	GO:0004497	4,29E-05	monooxygenase activity
Δhom2	12-day	2486680	down	GO:0004497	4,29E-05	monooxygenase activity
Δhom2	12-day	2503471	down	GO:0004497	4,29E-05	monooxygenase activity
Δhom2	12-day	2510618	down	GO:0004497	4,29E-05	monooxygenase activity
Δhom2	12-day	2512276	down	GO:0004497	4,29E-05	monooxygenase activity
Δhom2	12-day	2583618	down	GO:0004497	4,29E-05	monooxygenase activity
Δhom2	12-day	2604096	down	GO:0004497	4,29E-05	monooxygenase activity
Δhom2	12-day	2604231	down	GO:0004497	4,29E-05	monooxygenase activity
Δhom2	12-day	2604735	down	GO:0004497	4,29E-05	monooxygenase activity
Δhom2	12-day	2611234	down	GO:0004497	4,29E-05	monooxygenase activity
Δhom2	12-day	2618455	down	GO:0004497	4,29E-05	monooxygenase activity
Δhom2	12-day	2619674	down	GO:0004497	4,29E-05	monooxygenase activity
Δhom2	12-day	2623115	down	GO:0004497	4,29E-05	monooxygenase activity
Δhom2	12-day	2623122	down	GO:0004497	4,29E-05	monooxygenase activity
Δhom2	12-day	2623196	down	GO:0004497	4,29E-05	monooxygenase activity
Δhom2	12-day	2628312	down	GO:0004497	4,29E-05	monooxygenase activity
Δhom2	12-day	2628645	down	GO:0004497	4,29E-05	monooxygenase activity
Δhom2	12-day	2628651	down	GO:0004497	4,29E-05	monooxygenase activity
Δhom2	12-day	2632925	down	GO:0004497	4,29E-05	monooxygenase activity
Δhom2	12-day	2634283	down	GO:0004497	4,29E-05	monooxygenase activity
Δhom2	12-day	2635487	down	GO:0004497	4,29E-05	monooxygenase activity
Δhom2	12-day	2635869	down	GO:0004497	4,29E-05	monooxygenase activity
Δhom2	12-day	2636544	down	GO:0004497	4,29E-05	monooxygenase activity
Δhom2	12-day	2644422	down	GO:0004497	4,29E-05	monooxygenase activity
Δhom2	12-day	2644430	down	GO:0004497	4,29E-05	monooxygenase activity
Δhom2	12-day	2668568	down	GO:0004497	4,29E-05	monooxygenase activity
Δhom2	12-day	2690522	down	GO:0004497	4,29E-05	monooxygenase activity
Δhom2	12-day	2703628	down	GO:0004497	4,29E-05	monooxygenase activity
Δhom2	12-day	2703966	down	GO:0004497	4,29E-05	monooxygenase activity
Δhom2	12-day	2735179	down	GO:0004497	4,29E-05	monooxygenase activity
Δhom2	12-day	78628	down	GO:0006118	0,000820624	electron transport
Δhom2	12-day	81631	down	GO:0006118	0,000820624	electron transport
Δhom2	12-day	1084300	down	GO:0006118	0,000820624	electron transport
Δhom2	12-day	1131542	down	GO:0006118	0,000820624	electron transport

Δhom2	12-day	1189794	down	GO:0006118	0,000820624	electron transport
Δhom2	12-day	1193647	down	GO:0006118	0,000820624	electron transport
Δhom2	12-day	2327903	down	GO:0006118	0,000820624	electron transport
Δhom2	12-day	2450442	down	GO:0006118	0,000820624	electron transport
Δhom2	12-day	2484868	down	GO:0006118	0,000820624	electron transport
Δhom2	12-day	2486680	down	GO:0006118	0,000820624	electron transport
Δhom2	12-day	2490518	down	GO:0006118	0,000820624	electron transport
Δhom2	12-day	2497674	down	GO:0006118	0,000820624	electron transport
Δhom2	12-day	2499521	down	GO:0006118	0,000820624	electron transport
Δhom2	12-day	2503471	down	GO:0006118	0,000820624	electron transport
Δhom2	12-day	2509716	down	GO:0006118	0,000820624	electron transport
Δhom2	12-day	2510618	down	GO:0006118	0,000820624	electron transport
Δhom2	12-day	2512276	down	GO:0006118	0,000820624	electron transport
Δhom2	12-day	2571870	down	GO:0006118	0,000820624	electron transport
Δhom2	12-day	2583618	down	GO:0006118	0,000820624	electron transport
Δhom2	12-day	2604096	down	GO:0006118	0,000820624	electron transport
Δhom2	12-day	2604231	down	GO:0006118	0,000820624	electron transport
Δhom2	12-day	2604628	down	GO:0006118	0,000820624	electron transport
Δhom2	12-day	2604735	down	GO:0006118	0,000820624	electron transport
Δhom2	12-day	2607911	down	GO:0006118	0,000820624	electron transport
Δhom2	12-day	2609831	down	GO:0006118	0,000820624	electron transport
Δhom2	12-day	2609840	down	GO:0006118	0,000820624	electron transport
Δhom2	12-day	2609854	down	GO:0006118	0,000820624	electron transport
Δhom2	12-day	2611234	down	GO:0006118	0,000820624	electron transport
Δhom2	12-day	2611753	down	GO:0006118	0,000820624	electron transport
Δhom2	12-day	2618455	down	GO:0006118	0,000820624	electron transport
Δhom2	12-day	2619674	down	GO:0006118	0,000820624	electron transport
Δhom2	12-day	2621855	down	GO:0006118	0,000820624	electron transport
Δhom2	12-day	2623115	down	GO:0006118	0,000820624	electron transport
Δhom2	12-day	2623122	down	GO:0006118	0,000820624	electron transport
Δhom2	12-day	2623196	down	GO:0006118	0,000820624	electron transport
Δhom2	12-day	2625667	down	GO:0006118	0,000820624	electron transport
Δhom2	12-day	2625951	down	GO:0006118	0,000820624	electron transport
Δhom2	12-day	2628312	down	GO:0006118	0,000820624	electron transport

Δhom2	12-day	2628645	down	GO:0006118	0,000820624	electron transport
Δhom2	12-day	2628651	down	GO:0006118	0,000820624	electron transport
Δhom2	12-day	2632925	down	GO:0006118	0,000820624	electron transport
Δhom2	12-day	2633167	down	GO:0006118	0,000820624	electron transport
Δhom2	12-day	2634283	down	GO:0006118	0,000820624	electron transport
Δhom2	12-day	2635487	down	GO:0006118	0,000820624	electron transport
Δhom2	12-day	2635869	down	GO:0006118	0,000820624	electron transport
Δhom2	12-day	2636544	down	GO:0006118	0,000820624	electron transport
Δhom2	12-day	2637104	down	GO:0006118	0,000820624	electron transport
Δhom2	12-day	2637190	down	GO:0006118	0,000820624	electron transport
Δhom2	12-day	2639682	down	GO:0006118	0,000820624	electron transport
Δhom2	12-day	2644422	down	GO:0006118	0,000820624	electron transport
Δhom2	12-day	2644430	down	GO:0006118	0,000820624	electron transport
Δhom2	12-day	2662585	down	GO:0006118	0,000820624	electron transport
Δhom2	12-day	2663372	down	GO:0006118	0,000820624	electron transport
Δhom2	12-day	2668568	down	GO:0006118	0,000820624	electron transport
Δhom2	12-day	2671376	down	GO:0006118	0,000820624	electron transport
Δhom2	12-day	2690522	down	GO:0006118	0,000820624	electron transport
Δhom2	12-day	2703628	down	GO:0006118	0,000820624	electron transport
Δhom2	12-day	2703966	down	GO:0006118	0,000820624	electron transport
Δhom2	12-day	2735179	down	GO:0006118	0,000820624	electron transport
Δhom2	12-day	1134402	down	GO:0005488	0,002534434	binding
Δhom2	12-day	1152293	down	GO:0005488	0,002534434	binding
Δhom2	12-day	1267603	down	GO:0005488	0,002534434	binding
Δhom2	12-day	2085050	down	GO:0005488	0,002534434	binding
Δhom2	12-day	2194185	down	GO:0005488	0,002534434	binding
Δhom2	12-day	2255031	down	GO:0005488	0,002534434	binding
Δhom2	12-day	2333133	down	GO:0005488	0,002534434	binding
Δhom2	12-day	2373661	down	GO:0005488	0,002534434	binding
Δhom2	12-day	2492848	down	GO:0005488	0,002534434	binding
Δhom2	12-day	2492878	down	GO:0005488	0,002534434	binding
Δhom2	12-day	2493904	down	GO:0005488	0,002534434	binding
Δhom2	12-day	2494727	down	GO:0005488	0,002534434	binding
Δhom2	12-day	2511020	down	GO:0005488	0,002534434	binding

Δhom2	12-day	2520877	down	GO:0005488	0,002534434	binding
Δhom2	12-day	2541321	down	GO:0005488	0,002534434	binding
Δhom2	12-day	2558552	down	GO:0005488	0,002534434	binding
Δhom2	12-day	2595361	down	GO:0005488	0,002534434	binding
Δhom2	12-day	2605752	down	GO:0005488	0,002534434	binding
Δhom2	12-day	2606988	down	GO:0005488	0,002534434	binding
Δhom2	12-day	2610372	down	GO:0005488	0,002534434	binding
Δhom2	12-day	2616880	down	GO:0005488	0,002534434	binding
Δhom2	12-day	2616916	down	GO:0005488	0,002534434	binding
Δhom2	12-day	2618904	down	GO:0005488	0,002534434	binding
Δhom2	12-day	2619714	down	GO:0005488	0,002534434	binding
Δhom2	12-day	2621873	down	GO:0005488	0,002534434	binding
Δhom2	12-day	2623301	down	GO:0005488	0,002534434	binding
Δhom2	12-day	2624675	down	GO:0005488	0,002534434	binding
Δhom2	12-day	2624698	down	GO:0005488	0,002534434	binding
Δhom2	12-day	2625667	down	GO:0005488	0,002534434	binding
Δhom2	12-day	2625782	down	GO:0005488	0,002534434	binding
Δhom2	12-day	2626510	down	GO:0005488	0,002534434	binding
Δhom2	12-day	2628751	down	GO:0005488	0,002534434	binding
Δhom2	12-day	2631237	down	GO:0005488	0,002534434	binding
Δhom2	12-day	2632611	down	GO:0005488	0,002534434	binding
Δhom2	12-day	2633167	down	GO:0005488	0,002534434	binding
Δhom2	12-day	2633266	down	GO:0005488	0,002534434	binding
Δhom2	12-day	2633632	down	GO:0005488	0,002534434	binding
Δhom2	12-day	2634450	down	GO:0005488	0,002534434	binding
Δhom2	12-day	2637088	down	GO:0005488	0,002534434	binding
Δhom2	12-day	2637104	down	GO:0005488	0,002534434	binding
Δhom2	12-day	2638484	down	GO:0005488	0,002534434	binding
Δhom2	12-day	2641506	down	GO:0005488	0,002534434	binding
Δhom2	12-day	2644281	down	GO:0005488	0,002534434	binding
Δhom2	12-day	2644330	down	GO:0005488	0,002534434	binding
Δhom2	12-day	2645850	down	GO:0005488	0,002534434	binding
Δhom2	12-day	2661491	down	GO:0005488	0,002534434	binding
Δhom2	12-day	2673857	down	GO:0005488	0,002534434	binding

Δhom2	12-day	2705421	down	GO:0005488	0,002534434	binding
Δhom2	12-day	2711060	down	GO:0005488	0,002534434	binding
Δhom2	12-day	2628225	down	GO:0005199	0,004589545	structural constituent of cell wall
Δhom2	12-day	2629603	down	GO:0005199	0,004589545	structural constituent of cell wall
Δhom2	12-day	2746250	down	GO:0005199	0,004589545	structural constituent of cell wall
Δhom2	12-day	237513	down	GO:0050660	0,004933165	FAD binding
Δhom2	12-day	2508435	down	GO:0050660	0,004933165	FAD binding
Δhom2	12-day	2540746	down	GO:0050660	0,004933165	FAD binding
Δhom2	12-day	2592894	down	GO:0050660	0,004933165	FAD binding
Δhom2	12-day	2625667	down	GO:0050660	0,004933165	FAD binding
Δhom2	12-day	2642607	down	GO:0050660	0,004933165	FAD binding
Δhom2	12-day	2643025	down	GO:0050660	0,004933165	FAD binding
Δhom2	12-day	2663372	down	GO:0050660	0,004933165	FAD binding
Δhom2	12-day	2688172	down	GO:0050660	0,004933165	FAD binding
Δhom2	12-day	2630193	down	GO:0000786	0,004933165	nucleosome
Δhom2	12-day	78628	down	GO:0020037	0,004933165	heme binding
Δhom2	12-day	81631	down	GO:0020037	0,004933165	heme binding
Δhom2	12-day	1131542	down	GO:0020037	0,004933165	heme binding
Δhom2	12-day	1189794	down	GO:0020037	0,004933165	heme binding
Δhom2	12-day	1193647	down	GO:0020037	0,004933165	heme binding
Δhom2	12-day	2327903	down	GO:0020037	0,004933165	heme binding
Δhom2	12-day	2376858	down	GO:0020037	0,004933165	heme binding
Δhom2	12-day	2484868	down	GO:0020037	0,004933165	heme binding
Δhom2	12-day	2486680	down	GO:0020037	0,004933165	heme binding
Δhom2	12-day	2497674	down	GO:0020037	0,004933165	heme binding
Δhom2	12-day	2509716	down	GO:0020037	0,004933165	heme binding
Δhom2	12-day	2510618	down	GO:0020037	0,004933165	heme binding
Δhom2	12-day	2512276	down	GO:0020037	0,004933165	heme binding
Δhom2	12-day	2583618	down	GO:0020037	0,004933165	heme binding
Δhom2	12-day	2604096	down	GO:0020037	0,004933165	heme binding
Δhom2	12-day	2604231	down	GO:0020037	0,004933165	heme binding
Δhom2	12-day	2604628	down	GO:0020037	0,004933165	heme binding
Δhom2	12-day	2604735	down	GO:0020037	0,004933165	heme binding
Δhom2	12-day	2607911	down	GO:0020037	0,004933165	heme binding

Δhom2	12-day	2609840	down	GO:0020037	0,004933165	heme binding
Δhom2	12-day	2609854	down	GO:0020037	0,004933165	heme binding
Δhom2	12-day	2611234	down	GO:0020037	0,004933165	heme binding
Δhom2	12-day	2619674	down	GO:0020037	0,004933165	heme binding
Δhom2	12-day	2621855	down	GO:0020037	0,004933165	heme binding
Δhom2	12-day	2623115	down	GO:0020037	0,004933165	heme binding
Δhom2	12-day	2623122	down	GO:0020037	0,004933165	heme binding
Δhom2	12-day	2623196	down	GO:0020037	0,004933165	heme binding
Δhom2	12-day	2624504	down	GO:0020037	0,004933165	heme binding
Δhom2	12-day	2628312	down	GO:0020037	0,004933165	heme binding
Δhom2	12-day	2628645	down	GO:0020037	0,004933165	heme binding
Δhom2	12-day	2628651	down	GO:0020037	0,004933165	heme binding
Δhom2	12-day	2632925	down	GO:0020037	0,004933165	heme binding
Δhom2	12-day	2634283	down	GO:0020037	0,004933165	heme binding
Δhom2	12-day	2635487	down	GO:0020037	0,004933165	heme binding
Δhom2	12-day	2635869	down	GO:0020037	0,004933165	heme binding
Δhom2	12-day	2636160	down	GO:0020037	0,004933165	heme binding
Δhom2	12-day	2636544	down	GO:0020037	0,004933165	heme binding
Δhom2	12-day	2637190	down	GO:0020037	0,004933165	heme binding
Δhom2	12-day	2668568	down	GO:0020037	0,004933165	heme binding
Δhom2	12-day	2690522	down	GO:0020037	0,004933165	heme binding
Δhom2	12-day	2703628	down	GO:0020037	0,004933165	heme binding
Δhom2	12-day	2703966	down	GO:0020037	0,004933165	heme binding
Δhom2	12-day	2735179	down	GO:0020037	0,004933165	heme binding
Δhom2	12-day	2614257	down	GO:0005618	0,006109402	cell wall
Δhom2	12-day	2628225	down	GO:0005618	0,006109402	cell wall
Δhom2	12-day	2629603	down	GO:0005618	0,006109402	cell wall
Δhom2	12-day	2630193	down	GO:0006334	0,010571833	nucleosome assembly
Δhom2	12-day	2637783	down	GO:0016831	0,01844007	carboxy-lyase activity
Δhom2	12-day	1144263	down	GO:0008733	0,024265206	L-arabinose isomerase activity
Δhom2	12-day	1153136	down	GO:0008733	0,024265206	L-arabinose isomerase activity
Δhom2	12-day	2509716	down	GO:0008733	0,024265206	L-arabinose isomerase activity
Δhom2	12-day	2516374	down	GO:0008733	0,024265206	L-arabinose isomerase activity
Δhom2	12-day	2613255	down	GO:0008733	0,024265206	L-arabinose isomerase activity

Δhom2	12-day	2622598	down	GO:0008733	0,024265206	L-arabinose isomerase activity
Δhom2	12-day	2627703	down	GO:0008733	0,024265206	L-arabinose isomerase activity
Δhom2	12-day	2629091	down	GO:0008733	0,024265206	L-arabinose isomerase activity
Δhom2	12-day	2701571	down	GO:0008733	0,024265206	L-arabinose isomerase activity
Δhom2	12-day	59565	down	GO:0008843	0,026460585	endochitinase activity
Δhom2	12-day	2623305	down	GO:0008843	0,026460585	endochitinase activity
Δhom2	12-day	2633726	down	GO:0008843	0,026460585	endochitinase activity
Δhom2	12-day	59565	down	GO:0004568	0,026460585	chitinase activity
Δhom2	12-day	2623305	down	GO:0004568	0,026460585	chitinase activity
Δhom2	12-day	2633726	down	GO:0004568	0,026460585	chitinase activity
Δhom2	12-day	238601	down	GO:0005351	0,042665445	sugar:hydrogen symporter activity
Δhom2	12-day	1144263	down	GO:0005351	0,042665445	sugar:hydrogen symporter activity
Δhom2	12-day	2516374	down	GO:0005351	0,042665445	sugar:hydrogen symporter activity
Δhom2	12-day	2604984	down	GO:0005351	0,042665445	sugar:hydrogen symporter activity
Δhom2	12-day	2613255	down	GO:0005351	0,042665445	sugar:hydrogen symporter activity
Δhom2	12-day	2622598	down	GO:0005351	0,042665445	sugar:hydrogen symporter activity
Δhom2	12-day	2627703	down	GO:0005351	0,042665445	sugar:hydrogen symporter activity
Δhom2	12-day	2629091	down	GO:0005351	0,042665445	sugar:hydrogen symporter activity
Δhom2	12-day	2662585	down	GO:0005351	0,042665445	sugar:hydrogen symporter activity
Δhom2	12-day	2701571	down	GO:0005351	0,042665445	sugar:hydrogen symporter activity
Δwc1	12-day	1083109	up	GO:0005506	1,08E-12	iron ion binding
Δwc1	12-day	1120318	up	GO:0005506	1,08E-12	iron ion binding
Δwc1	12-day	1147397	up	GO:0005506	1,08E-12	iron ion binding
Δwc1	12-day	1173144	up	GO:0005506	1,08E-12	iron ion binding
Δwc1	12-day	2364606	up	GO:0005506	1,08E-12	iron ion binding
Δwc1	12-day	2491624	up	GO:0005506	1,08E-12	iron ion binding
Δwc1	12-day	2493935	up	GO:0005506	1,08E-12	iron ion binding
Δwc1	12-day	2501258	up	GO:0005506	1,08E-12	iron ion binding
Δwc1	12-day	2514636	up	GO:0005506	1,08E-12	iron ion binding
Δwc1	12-day	2537529	up	GO:0005506	1,08E-12	iron ion binding
Δwc1	12-day	2547557	up	GO:0005506	1,08E-12	iron ion binding
Δwc1	12-day	2559987	up	GO:0005506	1,08E-12	iron ion binding
Δwc1	12-day	2619716	up	GO:0005506	1,08E-12	iron ion binding
Δwc1	12-day	2620407	up	GO:0005506	1,08E-12	iron ion binding

Δwc1	12-day	2625023	up	GO:0005506	1,08E-12	iron ion binding
Δwc1	12-day	2626841	up	GO:0005506	1,08E-12	iron ion binding
Δwc1	12-day	2628300	up	GO:0005506	1,08E-12	iron ion binding
Δwc1	12-day	2629308	up	GO:0005506	1,08E-12	iron ion binding
Δwc1	12-day	2635949	up	GO:0005506	1,08E-12	iron ion binding
Δwc1	12-day	2637467	up	GO:0005506	1,08E-12	iron ion binding
Δwc1	12-day	2638350	up	GO:0005506	1,08E-12	iron ion binding
Δwc1	12-day	2640550	up	GO:0005506	1,08E-12	iron ion binding
Δwc1	12-day	2666440	up	GO:0005506	1,08E-12	iron ion binding
Δwc1	12-day	2695849	up	GO:0005506	1,08E-12	iron ion binding
Δwc1	12-day	2703965	up	GO:0005506	1,08E-12	iron ion binding
Δwc1	12-day	1083109	up	GO:0020037	3,49E-11	heme binding
Δwc1	12-day	1120318	up	GO:0020037	3,49E-11	heme binding
Δwc1	12-day	1147397	up	GO:0020037	3,49E-11	heme binding
Δwc1	12-day	1173144	up	GO:0020037	3,49E-11	heme binding
Δwc1	12-day	2364606	up	GO:0020037	3,49E-11	heme binding
Δwc1	12-day	2491624	up	GO:0020037	3,49E-11	heme binding
Δwc1	12-day	2501258	up	GO:0020037	3,49E-11	heme binding
Δwc1	12-day	2514636	up	GO:0020037	3,49E-11	heme binding
Δwc1	12-day	2537529	up	GO:0020037	3,49E-11	heme binding
Δwc1	12-day	2547557	up	GO:0020037	3,49E-11	heme binding
Δwc1	12-day	2559987	up	GO:0020037	3,49E-11	heme binding
Δwc1	12-day	2583399	up	GO:0020037	3,49E-11	heme binding
Δwc1	12-day	2608091	up	GO:0020037	3,49E-11	heme binding
Δwc1	12-day	2619716	up	GO:0020037	3,49E-11	heme binding
Δwc1	12-day	2620407	up	GO:0020037	3,49E-11	heme binding
Δwc1	12-day	2625023	up	GO:0020037	3,49E-11	heme binding
Δwc1	12-day	2626841	up	GO:0020037	3,49E-11	heme binding
Δwc1	12-day	2628300	up	GO:0020037	3,49E-11	heme binding
Δwc1	12-day	2629308	up	GO:0020037	3,49E-11	heme binding
Δwc1	12-day	2635949	up	GO:0020037	3,49E-11	heme binding
Δwc1	12-day	2637467	up	GO:0020037	3,49E-11	heme binding
Δwc1	12-day	2638350	up	GO:0020037	3,49E-11	heme binding
Δwc1	12-day	2640550	up	GO:0020037	3,49E-11	heme binding

Δwc1	12-day	2640585	up	GO:0020037	3,49E-11	heme binding
Δwc1	12-day	2643274	up	GO:0020037	3,49E-11	heme binding
Δwc1	12-day	2666440	up	GO:0020037	3,49E-11	heme binding
Δwc1	12-day	2673121	up	GO:0020037	3,49E-11	heme binding
Δwc1	12-day	2695849	up	GO:0020037	3,49E-11	heme binding
Δwc1	12-day	2703965	up	GO:0020037	3,49E-11	heme binding
Δwc1	12-day	1120318	up	GO:0004497	5,17E-10	monooxygenase activity
Δwc1	12-day	1147397	up	GO:0004497	5,17E-10	monooxygenase activity
Δwc1	12-day	1168026	up	GO:0004497	5,17E-10	monooxygenase activity
Δwc1	12-day	1173144	up	GO:0004497	5,17E-10	monooxygenase activity
Δwc1	12-day	1342670	up	GO:0004497	5,17E-10	monooxygenase activity
Δwc1	12-day	2213880	up	GO:0004497	5,17E-10	monooxygenase activity
Δwc1	12-day	2364606	up	GO:0004497	5,17E-10	monooxygenase activity
Δwc1	12-day	2490194	up	GO:0004497	5,17E-10	monooxygenase activity
Δwc1	12-day	2491624	up	GO:0004497	5,17E-10	monooxygenase activity
Δwc1	12-day	2501258	up	GO:0004497	5,17E-10	monooxygenase activity
Δwc1	12-day	2514636	up	GO:0004497	5,17E-10	monooxygenase activity
Δwc1	12-day	2537529	up	GO:0004497	5,17E-10	monooxygenase activity
Δwc1	12-day	2547557	up	GO:0004497	5,17E-10	monooxygenase activity
Δwc1	12-day	2559987	up	GO:0004497	5,17E-10	monooxygenase activity
Δwc1	12-day	2573393	up	GO:0004497	5,17E-10	monooxygenase activity
Δwc1	12-day	2573396	up	GO:0004497	5,17E-10	monooxygenase activity
Δwc1	12-day	2619716	up	GO:0004497	5,17E-10	monooxygenase activity
Δwc1	12-day	2620407	up	GO:0004497	5,17E-10	monooxygenase activity
Δwc1	12-day	2625023	up	GO:0004497	5,17E-10	monooxygenase activity
Δwc1	12-day	2626841	up	GO:0004497	5,17E-10	monooxygenase activity
Δwc1	12-day	2628300	up	GO:0004497	5,17E-10	monooxygenase activity
Δwc1	12-day	2629308	up	GO:0004497	5,17E-10	monooxygenase activity
Δwc1	12-day	2635949	up	GO:0004497	5,17E-10	monooxygenase activity
Δwc1	12-day	2637467	up	GO:0004497	5,17E-10	monooxygenase activity
Δwc1	12-day	2638350	up	GO:0004497	5,17E-10	monooxygenase activity
Δwc1	12-day	2640550	up	GO:0004497	5,17E-10	monooxygenase activity
Δwc1	12-day	2666440	up	GO:0004497	5,17E-10	monooxygenase activity
Δwc1	12-day	2695849	up	GO:0004497	5,17E-10	monooxygenase activity

Δwc1	12-day	2703927	up	GO:0004497	5,17E-10	monooxygenase activity
Δwc1	12-day	2703965	up	GO:0004497	5,17E-10	monooxygenase activity
Δwc1	12-day	1120318	up	GO:0006118	1,18E-08	electron transport
Δwc1	12-day	1147397	up	GO:0006118	1,18E-08	electron transport
Δwc1	12-day	1168026	up	GO:0006118	1,18E-08	electron transport
Δwc1	12-day	1173144	up	GO:0006118	1,18E-08	electron transport
Δwc1	12-day	1215660	up	GO:0006118	1,18E-08	electron transport
Δwc1	12-day	1342670	up	GO:0006118	1,18E-08	electron transport
Δwc1	12-day	2213880	up	GO:0006118	1,18E-08	electron transport
Δwc1	12-day	2364606	up	GO:0006118	1,18E-08	electron transport
Δwc1	12-day	2457440	up	GO:0006118	1,18E-08	electron transport
Δwc1	12-day	2490194	up	GO:0006118	1,18E-08	electron transport
Δwc1	12-day	2491624	up	GO:0006118	1,18E-08	electron transport
Δwc1	12-day	2501258	up	GO:0006118	1,18E-08	electron transport
Δwc1	12-day	2505277	up	GO:0006118	1,18E-08	electron transport
Δwc1	12-day	2508276	up	GO:0006118	1,18E-08	electron transport
Δwc1	12-day	2514636	up	GO:0006118	1,18E-08	electron transport
Δwc1	12-day	2537529	up	GO:0006118	1,18E-08	electron transport
Δwc1	12-day	2547557	up	GO:0006118	1,18E-08	electron transport
Δwc1	12-day	2559987	up	GO:0006118	1,18E-08	electron transport
Δwc1	12-day	2573393	up	GO:0006118	1,18E-08	electron transport
Δwc1	12-day	2573396	up	GO:0006118	1,18E-08	electron transport
Δwc1	12-day	2583399	up	GO:0006118	1,18E-08	electron transport
Δwc1	12-day	2594205	up	GO:0006118	1,18E-08	electron transport
Δwc1	12-day	2605889	up	GO:0006118	1,18E-08	electron transport
Δwc1	12-day	2608235	up	GO:0006118	1,18E-08	electron transport
Δwc1	12-day	2615184	up	GO:0006118	1,18E-08	electron transport
Δwc1	12-day	2616557	up	GO:0006118	1,18E-08	electron transport
Δwc1	12-day	2619716	up	GO:0006118	1,18E-08	electron transport
Δwc1	12-day	2620407	up	GO:0006118	1,18E-08	electron transport
Δwc1	12-day	2622733	up	GO:0006118	1,18E-08	electron transport
Δwc1	12-day	2625023	up	GO:0006118	1,18E-08	electron transport
Δwc1	12-day	2625730	up	GO:0006118	1,18E-08	electron transport
Δwc1	12-day	2626841	up	GO:0006118	1,18E-08	electron transport

Δwc1	12-day	2628120	up	GO:0006118	1,18E-08	electron transport
Δwc1	12-day	2628300	up	GO:0006118	1,18E-08	electron transport
Δwc1	12-day	2629308	up	GO:0006118	1,18E-08	electron transport
Δwc1	12-day	2635949	up	GO:0006118	1,18E-08	electron transport
Δwc1	12-day	2637467	up	GO:0006118	1,18E-08	electron transport
Δwc1	12-day	2638350	up	GO:0006118	1,18E-08	electron transport
Δwc1	12-day	2638473	up	GO:0006118	1,18E-08	electron transport
Δwc1	12-day	2640550	up	GO:0006118	1,18E-08	electron transport
Δwc1	12-day	2643274	up	GO:0006118	1,18E-08	electron transport
Δwc1	12-day	2645843	up	GO:0006118	1,18E-08	electron transport
Δwc1	12-day	2666009	up	GO:0006118	1,18E-08	electron transport
Δwc1	12-day	2666440	up	GO:0006118	1,18E-08	electron transport
Δwc1	12-day	2667289	up	GO:0006118	1,18E-08	electron transport
Δwc1	12-day	2695849	up	GO:0006118	1,18E-08	electron transport
Δwc1	12-day	2698211	up	GO:0006118	1,18E-08	electron transport
Δwc1	12-day	2703927	up	GO:0006118	1,18E-08	electron transport
Δwc1	12-day	2703965	up	GO:0006118	1,18E-08	electron transport
Δwc1	12-day	1120318	up	GO:0050381	3,23E-08	unspecific monooxygenase activity
Δwc1	12-day	2491624	up	GO:0050381	3,23E-08	unspecific monooxygenase activity
Δwc1	12-day	2514636	up	GO:0050381	3,23E-08	unspecific monooxygenase activity
Δwc1	12-day	2547557	up	GO:0050381	3,23E-08	unspecific monooxygenase activity
Δwc1	12-day	2559987	up	GO:0050381	3,23E-08	unspecific monooxygenase activity
Δwc1	12-day	2619716	up	GO:0050381	3,23E-08	unspecific monooxygenase activity
Δwc1	12-day	2620407	up	GO:0050381	3,23E-08	unspecific monooxygenase activity
Δwc1	12-day	2625023	up	GO:0050381	3,23E-08	unspecific monooxygenase activity
Δwc1	12-day	2637467	up	GO:0050381	3,23E-08	unspecific monooxygenase activity
Δwc1	12-day	2638350	up	GO:0050381	3,23E-08	unspecific monooxygenase activity
Δwc1	12-day	2666440	up	GO:0050381	3,23E-08	unspecific monooxygenase activity
Δwc1	12-day	2703965	up	GO:0050381	3,23E-08	unspecific monooxygenase activity
Δwc1	12-day	66483	up	GO:0005975	3,11E-07	carbohydrate metabolic process
Δwc1	12-day	85210	up	GO:0005975	3,11E-07	carbohydrate metabolic process
Δwc1	12-day	234329	up	GO:0005975	3,11E-07	carbohydrate metabolic process
Δwc1	12-day	2486953	up	GO:0005975	3,11E-07	carbohydrate metabolic process
Δwc1	12-day	2493241	up	GO:0005975	3,11E-07	carbohydrate metabolic process

Δwc1	12-day	2514546	up	GO:0005975	3,11E-07	carbohydrate metabolic process
Δwc1	12-day	2570936	up	GO:0005975	3,11E-07	carbohydrate metabolic process
Δwc1	12-day	2613657	up	GO:0005975	3,11E-07	carbohydrate metabolic process
Δwc1	12-day	2621806	up	GO:0005975	3,11E-07	carbohydrate metabolic process
Δwc1	12-day	2622563	up	GO:0005975	3,11E-07	carbohydrate metabolic process
Δwc1	12-day	2624823	up	GO:0005975	3,11E-07	carbohydrate metabolic process
Δwc1	12-day	2628008	up	GO:0005975	3,11E-07	carbohydrate metabolic process
Δwc1	12-day	2633791	up	GO:0005975	3,11E-07	carbohydrate metabolic process
Δwc1	12-day	2634915	up	GO:0005975	3,11E-07	carbohydrate metabolic process
Δwc1	12-day	2637065	up	GO:0005975	3,11E-07	carbohydrate metabolic process
Δwc1	12-day	2641020	up	GO:0005975	3,11E-07	carbohydrate metabolic process
Δwc1	12-day	2642958	up	GO:0005975	3,11E-07	carbohydrate metabolic process
Δwc1	12-day	2643740	up	GO:0005975	3,11E-07	carbohydrate metabolic process
Δwc1	12-day	2644666	up	GO:0005975	3,11E-07	carbohydrate metabolic process
Δwc1	12-day	2670422	up	GO:0005975	3,11E-07	carbohydrate metabolic process
Δwc1	12-day	2686544	up	GO:0005975	3,11E-07	carbohydrate metabolic process
Δwc1	12-day	2706622	up	GO:0005975	3,11E-07	carbohydrate metabolic process
Δwc1	12-day	85210	up	GO:0004553	4,47E-06	hydrolase activity, hydrolyzing O-glycosyl compounds
Δwc1	12-day	234329	up	GO:0004553	4,47E-06	hydrolase activity, hydrolyzing O-glycosyl compounds
Δwc1	12-day	2486953	up	GO:0004553	4,47E-06	hydrolase activity, hydrolyzing O-glycosyl compounds
Δwc1	12-day	2613657	up	GO:0004553	4,47E-06	hydrolase activity, hydrolyzing O-glycosyl compounds
Δwc1	12-day	2621806	up	GO:0004553	4,47E-06	hydrolase activity, hydrolyzing O-glycosyl compounds
Δwc1	12-day	2622563	up	GO:0004553	4,47E-06	hydrolase activity, hydrolyzing O-glycosyl compounds
Δwc1	12-day	2637065	up	GO:0004553	4,47E-06	hydrolase activity, hydrolyzing O-glycosyl compounds
Δwc1	12-day	2642958	up	GO:0004553	4,47E-06	hydrolase activity, hydrolyzing O-glycosyl compounds
Δwc1	12-day	2643740	up	GO:0004553	4,47E-06	hydrolase activity, hydrolyzing O-glycosyl compounds
Δwc1	12-day	2644666	up	GO:0004553	4,47E-06	hydrolase activity, hydrolyzing O-glycosyl compounds
Δwc1	12-day	2670422	up	GO:0004553	4,47E-06	hydrolase activity, hydrolyzing O-glycosyl compounds
Δwc1	12-day	2686544	up	GO:0004553	4,47E-06	hydrolase activity, hydrolyzing O-glycosyl compounds
Δwc1	12-day	2706622	up	GO:0004553	4,47E-06	hydrolase activity, hydrolyzing O-glycosyl compounds
Δwc1	12-day	258402	up	GO:0005524	1,55E-05	ATP binding
Δwc1	12-day	1129062	up	GO:0005524	1,55E-05	ATP binding
Δwc1	12-day	1158800	up	GO:0005524	1,55E-05	ATP binding
Δwc1	12-day	1172108	up	GO:0005524	1,55E-05	ATP binding

Δwc1	12-day	1174869	up	GO:0005524	1,55E-05	ATP binding
Δwc1	12-day	1178759	up	GO:0005524	1,55E-05	ATP binding
Δwc1	12-day	1181112	up	GO:0005524	1,55E-05	ATP binding
Δwc1	12-day	1186310	up	GO:0005524	1,55E-05	ATP binding
Δwc1	12-day	1196424	up	GO:0005524	1,55E-05	ATP binding
Δwc1	12-day	2253810	up	GO:0005524	1,55E-05	ATP binding
Δwc1	12-day	2279823	up	GO:0005524	1,55E-05	ATP binding
Δwc1	12-day	2481145	up	GO:0005524	1,55E-05	ATP binding
Δwc1	12-day	2484841	up	GO:0005524	1,55E-05	ATP binding
Δwc1	12-day	2486797	up	GO:0005524	1,55E-05	ATP binding
Δwc1	12-day	2487176	up	GO:0005524	1,55E-05	ATP binding
Δwc1	12-day	2490823	up	GO:0005524	1,55E-05	ATP binding
Δwc1	12-day	2493556	up	GO:0005524	1,55E-05	ATP binding
Δwc1	12-day	2503832	up	GO:0005524	1,55E-05	ATP binding
Δwc1	12-day	2510275	up	GO:0005524	1,55E-05	ATP binding
Δwc1	12-day	2511561	up	GO:0005524	1,55E-05	ATP binding
Δwc1	12-day	2527752	up	GO:0005524	1,55E-05	ATP binding
Δwc1	12-day	2533371	up	GO:0005524	1,55E-05	ATP binding
Δwc1	12-day	2535527	up	GO:0005524	1,55E-05	ATP binding
Δwc1	12-day	2550939	up	GO:0005524	1,55E-05	ATP binding
Δwc1	12-day	2553325	up	GO:0005524	1,55E-05	ATP binding
Δwc1	12-day	2554917	up	GO:0005524	1,55E-05	ATP binding
Δwc1	12-day	2563653	up	GO:0005524	1,55E-05	ATP binding
Δwc1	12-day	2565471	up	GO:0005524	1,55E-05	ATP binding
Δwc1	12-day	2569377	up	GO:0005524	1,55E-05	ATP binding
Δwc1	12-day	2585708	up	GO:0005524	1,55E-05	ATP binding
Δwc1	12-day	2601543	up	GO:0005524	1,55E-05	ATP binding
Δwc1	12-day	2605230	up	GO:0005524	1,55E-05	ATP binding
Δwc1	12-day	2606202	up	GO:0005524	1,55E-05	ATP binding
Δwc1	12-day	2610180	up	GO:0005524	1,55E-05	ATP binding
Δwc1	12-day	2611700	up	GO:0005524	1,55E-05	ATP binding
Δwc1	12-day	2612206	up	GO:0005524	1,55E-05	ATP binding
Δwc1	12-day	2612608	up	GO:0005524	1,55E-05	ATP binding
Δwc1	12-day	2614625	up	GO:0005524	1,55E-05	ATP binding

Δwc1	12-day	2614856	up	GO:0005524	1,55E-05	ATP binding
Δwc1	12-day	2620969	up	GO:0005524	1,55E-05	ATP binding
Δwc1	12-day	2621533	up	GO:0005524	1,55E-05	ATP binding
Δwc1	12-day	2621626	up	GO:0005524	1,55E-05	ATP binding
Δwc1	12-day	2621741	up	GO:0005524	1,55E-05	ATP binding
Δwc1	12-day	2623257	up	GO:0005524	1,55E-05	ATP binding
Δwc1	12-day	2623685	up	GO:0005524	1,55E-05	ATP binding
Δwc1	12-day	2631390	up	GO:0005524	1,55E-05	ATP binding
Δwc1	12-day	2632313	up	GO:0005524	1,55E-05	ATP binding
Δwc1	12-day	2632524	up	GO:0005524	1,55E-05	ATP binding
Δwc1	12-day	2638986	up	GO:0005524	1,55E-05	ATP binding
Δwc1	12-day	2641678	up	GO:0005524	1,55E-05	ATP binding
Δwc1	12-day	2645675	up	GO:0005524	1,55E-05	ATP binding
Δwc1	12-day	2661304	up	GO:0005524	1,55E-05	ATP binding
Δwc1	12-day	2662880	up	GO:0005524	1,55E-05	ATP binding
Δwc1	12-day	2672807	up	GO:0005524	1,55E-05	ATP binding
Δwc1	12-day	2675859	up	GO:0005524	1,55E-05	ATP binding
Δwc1	12-day	2682841	up	GO:0005524	1,55E-05	ATP binding
Δwc1	12-day	2684163	up	GO:0005524	1,55E-05	ATP binding
Δwc1	12-day	2684679	up	GO:0005524	1,55E-05	ATP binding
Δwc1	12-day	2693043	up	GO:0005524	1,55E-05	ATP binding
Δwc1	12-day	2693940	up	GO:0005524	1,55E-05	ATP binding
Δwc1	12-day	2702947	up	GO:0005524	1,55E-05	ATP binding
Δwc1	12-day	2725125	up	GO:0005524	1,55E-05	ATP binding
Δwc1	12-day	2737152	up	GO:0005524	1,55E-05	ATP binding
Δwc1	12-day	2498450	up	GO:0016810	0,007678636	hydrolase activity, acting on carbon-nitrogen (but not peptide) bonds
Δwc1	12-day	2554832	up	GO:0016810	0,007678636	hydrolase activity, acting on carbon-nitrogen (but not peptide) bonds
Δwc1	12-day	2641020	up	GO:0016810	0,007678636	hydrolase activity, acting on carbon-nitrogen (but not peptide) bonds
Δwc1	12-day	1136736	up	GO:0005215	0,013523922	transporter activity
Δwc1	12-day	1147635	up	GO:0005215	0,013523922	transporter activity
Δwc1	12-day	1196424	up	GO:0005215	0,013523922	transporter activity

Δwc1	12-day	1234296	up	GO:0005215	0,013523922	transporter activity
Δwc1	12-day	2481323	up	GO:0005215	0,013523922	transporter activity
Δwc1	12-day	2492230	up	GO:0005215	0,013523922	transporter activity
Δwc1	12-day	2498026	up	GO:0005215	0,013523922	transporter activity
Δwc1	12-day	2499364	up	GO:0005215	0,013523922	transporter activity
Δwc1	12-day	2502284	up	GO:0005215	0,013523922	transporter activity
Δwc1	12-day	2508421	up	GO:0005215	0,013523922	transporter activity
Δwc1	12-day	2515031	up	GO:0005215	0,013523922	transporter activity
Δwc1	12-day	2564524	up	GO:0005215	0,013523922	transporter activity
Δwc1	12-day	2612313	up	GO:0005215	0,013523922	transporter activity
Δwc1	12-day	2617636	up	GO:0005215	0,013523922	transporter activity
Δwc1	12-day	2618842	up	GO:0005215	0,013523922	transporter activity
Δwc1	12-day	2619010	up	GO:0005215	0,013523922	transporter activity
Δwc1	12-day	2622931	up	GO:0005215	0,013523922	transporter activity
Δwc1	12-day	2623043	up	GO:0005215	0,013523922	transporter activity
Δwc1	12-day	2628098	up	GO:0005215	0,013523922	transporter activity
Δwc1	12-day	2628982	up	GO:0005215	0,013523922	transporter activity
Δwc1	12-day	2630257	up	GO:0005215	0,013523922	transporter activity
Δwc1	12-day	2631348	up	GO:0005215	0,013523922	transporter activity
Δwc1	12-day	2632105	up	GO:0005215	0,013523922	transporter activity
Δwc1	12-day	2633206	up	GO:0005215	0,013523922	transporter activity
Δwc1	12-day	2633212	up	GO:0005215	0,013523922	transporter activity
Δwc1	12-day	2633596	up	GO:0005215	0,013523922	transporter activity
Δwc1	12-day	2637652	up	GO:0005215	0,013523922	transporter activity
Δwc1	12-day	2641095	up	GO:0005215	0,013523922	transporter activity
Δwc1	12-day	2641735	up	GO:0005215	0,013523922	transporter activity
Δwc1	12-day	2642894	up	GO:0005215	0,013523922	transporter activity
Δwc1	12-day	2660764	up	GO:0005215	0,013523922	transporter activity
Δwc1	12-day	2663452	up	GO:0005215	0,013523922	transporter activity
Δwc1	12-day	2680674	up	GO:0005215	0,013523922	transporter activity
Δwc1	12-day	2698170	up	GO:0005215	0,013523922	transporter activity
Δwc1	12-day	2704067	up	GO:0005215	0,013523922	transporter activity
Δwc1	12-day	13059	up	GO:0006508	0,0227299	proteolysis
Δwc1	12-day	54219	up	GO:0006508	0,0227299	proteolysis

Δwc1	12-day	75642	up	GO:0006508	0,0227299	proteolysis
Δwc1	12-day	1190859	up	GO:0006508	0,0227299	proteolysis
Δwc1	12-day	2268565	up	GO:0006508	0,0227299	proteolysis
Δwc1	12-day	2494345	up	GO:0006508	0,0227299	proteolysis
Δwc1	12-day	2495551	up	GO:0006508	0,0227299	proteolysis
Δwc1	12-day	2495563	up	GO:0006508	0,0227299	proteolysis
Δwc1	12-day	2499350	up	GO:0006508	0,0227299	proteolysis
Δwc1	12-day	2501086	up	GO:0006508	0,0227299	proteolysis
Δwc1	12-day	2508594	up	GO:0006508	0,0227299	proteolysis
Δwc1	12-day	2521109	up	GO:0006508	0,0227299	proteolysis
Δwc1	12-day	2525660	up	GO:0006508	0,0227299	proteolysis
Δwc1	12-day	2597093	up	GO:0006508	0,0227299	proteolysis
Δwc1	12-day	2607695	up	GO:0006508	0,0227299	proteolysis
Δwc1	12-day	2611979	up	GO:0006508	0,0227299	proteolysis
Δwc1	12-day	2614398	up	GO:0006508	0,0227299	proteolysis
Δwc1	12-day	2616091	up	GO:0006508	0,0227299	proteolysis
Δwc1	12-day	2625721	up	GO:0006508	0,0227299	proteolysis
Δwc1	12-day	2627176	up	GO:0006508	0,0227299	proteolysis
Δwc1	12-day	2632766	up	GO:0006508	0,0227299	proteolysis
Δwc1	12-day	2635416	up	GO:0006508	0,0227299	proteolysis
Δwc1	12-day	2638986	up	GO:0006508	0,0227299	proteolysis
Δwc1	12-day	2639300	up	GO:0006508	0,0227299	proteolysis
Δwc1	12-day	2668049	up	GO:0006508	0,0227299	proteolysis
Δwc1	12-day	2621806	up	GO:0006032	0,02741828	chitin catabolic process
Δwc1	12-day	2643740	up	GO:0006032	0,02741828	chitin catabolic process
Δwc1	12-day	2644666	up	GO:0006032	0,02741828	chitin catabolic process
Δwc1	12-day	2706622	up	GO:0006032	0,02741828	chitin catabolic process
Δwc1	12-day	1092260	down	GO:0008152	1,56E-07	metabolic process
Δwc1	12-day	1152293	down	GO:0008152	1,56E-07	metabolic process
Δwc1	12-day	1200798	down	GO:0008152	1,56E-07	metabolic process
Δwc1	12-day	2135332	down	GO:0008152	1,56E-07	metabolic process
Δwc1	12-day	2161304	down	GO:0008152	1,56E-07	metabolic process
Δwc1	12-day	2194185	down	GO:0008152	1,56E-07	metabolic process
Δwc1	12-day	2255031	down	GO:0008152	1,56E-07	metabolic process

Δwc1	12-day	2333133	down	GO:0008152	1,56E-07	metabolic process
Δwc1	12-day	2492878	down	GO:0008152	1,56E-07	metabolic process
Δwc1	12-day	2495032	down	GO:0008152	1,56E-07	metabolic process
Δwc1	12-day	2507164	down	GO:0008152	1,56E-07	metabolic process
Δwc1	12-day	2553610	down	GO:0008152	1,56E-07	metabolic process
Δwc1	12-day	2577440	down	GO:0008152	1,56E-07	metabolic process
Δwc1	12-day	2579143	down	GO:0008152	1,56E-07	metabolic process
Δwc1	12-day	2604917	down	GO:0008152	1,56E-07	metabolic process
Δwc1	12-day	2606988	down	GO:0008152	1,56E-07	metabolic process
Δwc1	12-day	2607837	down	GO:0008152	1,56E-07	metabolic process
Δwc1	12-day	2608048	down	GO:0008152	1,56E-07	metabolic process
Δwc1	12-day	2609150	down	GO:0008152	1,56E-07	metabolic process
Δwc1	12-day	2610372	down	GO:0008152	1,56E-07	metabolic process
Δwc1	12-day	2610587	down	GO:0008152	1,56E-07	metabolic process
Δwc1	12-day	2611868	down	GO:0008152	1,56E-07	metabolic process
Δwc1	12-day	2615598	down	GO:0008152	1,56E-07	metabolic process
Δwc1	12-day	2616880	down	GO:0008152	1,56E-07	metabolic process
Δwc1	12-day	2616916	down	GO:0008152	1,56E-07	metabolic process
Δwc1	12-day	2618904	down	GO:0008152	1,56E-07	metabolic process
Δwc1	12-day	2619714	down	GO:0008152	1,56E-07	metabolic process
Δwc1	12-day	2619840	down	GO:0008152	1,56E-07	metabolic process
Δwc1	12-day	2621873	down	GO:0008152	1,56E-07	metabolic process
Δwc1	12-day	2622287	down	GO:0008152	1,56E-07	metabolic process
Δwc1	12-day	2623301	down	GO:0008152	1,56E-07	metabolic process
Δwc1	12-day	2623956	down	GO:0008152	1,56E-07	metabolic process
Δwc1	12-day	2624525	down	GO:0008152	1,56E-07	metabolic process
Δwc1	12-day	2624675	down	GO:0008152	1,56E-07	metabolic process
Δwc1	12-day	2625561	down	GO:0008152	1,56E-07	metabolic process
Δwc1	12-day	2625782	down	GO:0008152	1,56E-07	metabolic process
Δwc1	12-day	2626510	down	GO:0008152	1,56E-07	metabolic process
Δwc1	12-day	2627172	down	GO:0008152	1,56E-07	metabolic process
Δwc1	12-day	2627304	down	GO:0008152	1,56E-07	metabolic process
Δwc1	12-day	2629850	down	GO:0008152	1,56E-07	metabolic process
Δwc1	12-day	2631112	down	GO:0008152	1,56E-07	metabolic process

Δwc1	12-day	2631237	down	GO:0008152	1,56E-07	metabolic process
Δwc1	12-day	2631774	down	GO:0008152	1,56E-07	metabolic process
Δwc1	12-day	2632206	down	GO:0008152	1,56E-07	metabolic process
Δwc1	12-day	2632253	down	GO:0008152	1,56E-07	metabolic process
Δwc1	12-day	2632611	down	GO:0008152	1,56E-07	metabolic process
Δwc1	12-day	2633167	down	GO:0008152	1,56E-07	metabolic process
Δwc1	12-day	2633632	down	GO:0008152	1,56E-07	metabolic process
Δwc1	12-day	2636100	down	GO:0008152	1,56E-07	metabolic process
Δwc1	12-day	2637104	down	GO:0008152	1,56E-07	metabolic process
Δwc1	12-day	2637643	down	GO:0008152	1,56E-07	metabolic process
Δwc1	12-day	2637755	down	GO:0008152	1,56E-07	metabolic process
Δwc1	12-day	2638484	down	GO:0008152	1,56E-07	metabolic process
Δwc1	12-day	2638921	down	GO:0008152	1,56E-07	metabolic process
Δwc1	12-day	2640472	down	GO:0008152	1,56E-07	metabolic process
Δwc1	12-day	2640484	down	GO:0008152	1,56E-07	metabolic process
Δwc1	12-day	2640992	down	GO:0008152	1,56E-07	metabolic process
Δwc1	12-day	2641506	down	GO:0008152	1,56E-07	metabolic process
Δwc1	12-day	2641520	down	GO:0008152	1,56E-07	metabolic process
Δwc1	12-day	2643650	down	GO:0008152	1,56E-07	metabolic process
Δwc1	12-day	2643891	down	GO:0008152	1,56E-07	metabolic process
Δwc1	12-day	2644127	down	GO:0008152	1,56E-07	metabolic process
Δwc1	12-day	2644281	down	GO:0008152	1,56E-07	metabolic process
Δwc1	12-day	2644330	down	GO:0008152	1,56E-07	metabolic process
Δwc1	12-day	2644430	down	GO:0008152	1,56E-07	metabolic process
Δwc1	12-day	2645207	down	GO:0008152	1,56E-07	metabolic process
Δwc1	12-day	2645668	down	GO:0008152	1,56E-07	metabolic process
Δwc1	12-day	2660684	down	GO:0008152	1,56E-07	metabolic process
Δwc1	12-day	2665516	down	GO:0008152	1,56E-07	metabolic process
Δwc1	12-day	2705421	down	GO:0008152	1,56E-07	metabolic process
Δwc1	12-day	2711060	down	GO:0008152	1,56E-07	metabolic process
Δwc1	12-day	2726410	down	GO:0008152	1,56E-07	metabolic process
Δwc1	12-day	2728826	down	GO:0008152	1,56E-07	metabolic process
Δwc1	12-day	237513	down	GO:0016491	3,08E-07	oxidoreductase activity
Δwc1	12-day	1038608	down	GO:0016491	3,08E-07	oxidoreductase activity

Δwc1	12-day	1152293	down	GO:0016491	3,08E-07	oxidoreductase activity
Δwc1	12-day	2255031	down	GO:0016491	3,08E-07	oxidoreductase activity
Δwc1	12-day	2312801	down	GO:0016491	3,08E-07	oxidoreductase activity
Δwc1	12-day	2333133	down	GO:0016491	3,08E-07	oxidoreductase activity
Δwc1	12-day	2376858	down	GO:0016491	3,08E-07	oxidoreductase activity
Δwc1	12-day	2490518	down	GO:0016491	3,08E-07	oxidoreductase activity
Δwc1	12-day	2497674	down	GO:0016491	3,08E-07	oxidoreductase activity
Δwc1	12-day	2503108	down	GO:0016491	3,08E-07	oxidoreductase activity
Δwc1	12-day	2507164	down	GO:0016491	3,08E-07	oxidoreductase activity
Δwc1	12-day	2515964	down	GO:0016491	3,08E-07	oxidoreductase activity
Δwc1	12-day	2516444	down	GO:0016491	3,08E-07	oxidoreductase activity
Δwc1	12-day	2532967	down	GO:0016491	3,08E-07	oxidoreductase activity
Δwc1	12-day	2543454	down	GO:0016491	3,08E-07	oxidoreductase activity
Δwc1	12-day	2571870	down	GO:0016491	3,08E-07	oxidoreductase activity
Δwc1	12-day	2606988	down	GO:0016491	3,08E-07	oxidoreductase activity
Δwc1	12-day	2608048	down	GO:0016491	3,08E-07	oxidoreductase activity
Δwc1	12-day	2609840	down	GO:0016491	3,08E-07	oxidoreductase activity
Δwc1	12-day	2610372	down	GO:0016491	3,08E-07	oxidoreductase activity
Δwc1	12-day	2610587	down	GO:0016491	3,08E-07	oxidoreductase activity
Δwc1	12-day	2611538	down	GO:0016491	3,08E-07	oxidoreductase activity
Δwc1	12-day	2611868	down	GO:0016491	3,08E-07	oxidoreductase activity
Δwc1	12-day	2615160	down	GO:0016491	3,08E-07	oxidoreductase activity
Δwc1	12-day	2616880	down	GO:0016491	3,08E-07	oxidoreductase activity
Δwc1	12-day	2616916	down	GO:0016491	3,08E-07	oxidoreductase activity
Δwc1	12-day	2619840	down	GO:0016491	3,08E-07	oxidoreductase activity
Δwc1	12-day	2621873	down	GO:0016491	3,08E-07	oxidoreductase activity
Δwc1	12-day	2623301	down	GO:0016491	3,08E-07	oxidoreductase activity
Δwc1	12-day	2625561	down	GO:0016491	3,08E-07	oxidoreductase activity
Δwc1	12-day	2625917	down	GO:0016491	3,08E-07	oxidoreductase activity
Δwc1	12-day	2627304	down	GO:0016491	3,08E-07	oxidoreductase activity
Δwc1	12-day	2629850	down	GO:0016491	3,08E-07	oxidoreductase activity
Δwc1	12-day	2630637	down	GO:0016491	3,08E-07	oxidoreductase activity
Δwc1	12-day	2631774	down	GO:0016491	3,08E-07	oxidoreductase activity
Δwc1	12-day	2633167	down	GO:0016491	3,08E-07	oxidoreductase activity

Δwc1	12-day	2633632	down	GO:0016491	3,08E-07	oxidoreductase activity
Δwc1	12-day	2636160	down	GO:0016491	3,08E-07	oxidoreductase activity
Δwc1	12-day	2637104	down	GO:0016491	3,08E-07	oxidoreductase activity
Δwc1	12-day	2637755	down	GO:0016491	3,08E-07	oxidoreductase activity
Δwc1	12-day	2638484	down	GO:0016491	3,08E-07	oxidoreductase activity
Δwc1	12-day	2639682	down	GO:0016491	3,08E-07	oxidoreductase activity
Δwc1	12-day	2641368	down	GO:0016491	3,08E-07	oxidoreductase activity
Δwc1	12-day	2641506	down	GO:0016491	3,08E-07	oxidoreductase activity
Δwc1	12-day	2641520	down	GO:0016491	3,08E-07	oxidoreductase activity
Δwc1	12-day	2643891	down	GO:0016491	3,08E-07	oxidoreductase activity
Δwc1	12-day	2644127	down	GO:0016491	3,08E-07	oxidoreductase activity
Δwc1	12-day	2644330	down	GO:0016491	3,08E-07	oxidoreductase activity
Δwc1	12-day	2644430	down	GO:0016491	3,08E-07	oxidoreductase activity
Δwc1	12-day	2661224	down	GO:0016491	3,08E-07	oxidoreductase activity
Δwc1	12-day	2671376	down	GO:0016491	3,08E-07	oxidoreductase activity
Δwc1	12-day	2688172	down	GO:0016491	3,08E-07	oxidoreductase activity
Δwc1	12-day	2697968	down	GO:0016491	3,08E-07	oxidoreductase activity
Δwc1	12-day	2705421	down	GO:0016491	3,08E-07	oxidoreductase activity
Δwc1	12-day	2630193	down	GO:0000786	9,56E-07	nucleosome
Δwc1	12-day	2630193	down	GO:0006334	3,60E-06	nucleosome assembly
Δwc1	12-day	85278	down	GO:0003824	3,60E-06	catalytic activity
Δwc1	12-day	237513	down	GO:0003824	3,60E-06	catalytic activity
Δwc1	12-day	1092260	down	GO:0003824	3,60E-06	catalytic activity
Δwc1	12-day	1152293	down	GO:0003824	3,60E-06	catalytic activity
Δwc1	12-day	1184723	down	GO:0003824	3,60E-06	catalytic activity
Δwc1	12-day	1185226	down	GO:0003824	3,60E-06	catalytic activity
Δwc1	12-day	1191999	down	GO:0003824	3,60E-06	catalytic activity
Δwc1	12-day	1200798	down	GO:0003824	3,60E-06	catalytic activity
Δwc1	12-day	2135332	down	GO:0003824	3,60E-06	catalytic activity
Δwc1	12-day	2161304	down	GO:0003824	3,60E-06	catalytic activity
Δwc1	12-day	2194185	down	GO:0003824	3,60E-06	catalytic activity
Δwc1	12-day	2255031	down	GO:0003824	3,60E-06	catalytic activity
Δwc1	12-day	2333133	down	GO:0003824	3,60E-06	catalytic activity
Δwc1	12-day	2368049	down	GO:0003824	3,60E-06	catalytic activity

Δwc1	12-day	2492878	down	GO:0003824	3,60E-06	catalytic activity
Δwc1	12-day	2495032	down	GO:0003824	3,60E-06	catalytic activity
Δwc1	12-day	2516444	down	GO:0003824	3,60E-06	catalytic activity
Δwc1	12-day	2598960	down	GO:0003824	3,60E-06	catalytic activity
Δwc1	12-day	2605122	down	GO:0003824	3,60E-06	catalytic activity
Δwc1	12-day	2606988	down	GO:0003824	3,60E-06	catalytic activity
Δwc1	12-day	2607034	down	GO:0003824	3,60E-06	catalytic activity
Δwc1	12-day	2607837	down	GO:0003824	3,60E-06	catalytic activity
Δwc1	12-day	2608048	down	GO:0003824	3,60E-06	catalytic activity
Δwc1	12-day	2610372	down	GO:0003824	3,60E-06	catalytic activity
Δwc1	12-day	2610587	down	GO:0003824	3,60E-06	catalytic activity
Δwc1	12-day	2615598	down	GO:0003824	3,60E-06	catalytic activity
Δwc1	12-day	2616491	down	GO:0003824	3,60E-06	catalytic activity
Δwc1	12-day	2616880	down	GO:0003824	3,60E-06	catalytic activity
Δwc1	12-day	2616916	down	GO:0003824	3,60E-06	catalytic activity
Δwc1	12-day	2617562	down	GO:0003824	3,60E-06	catalytic activity
Δwc1	12-day	2618252	down	GO:0003824	3,60E-06	catalytic activity
Δwc1	12-day	2618425	down	GO:0003824	3,60E-06	catalytic activity
Δwc1	12-day	2618479	down	GO:0003824	3,60E-06	catalytic activity
Δwc1	12-day	2618904	down	GO:0003824	3,60E-06	catalytic activity
Δwc1	12-day	2618997	down	GO:0003824	3,60E-06	catalytic activity
Δwc1	12-day	2619714	down	GO:0003824	3,60E-06	catalytic activity
Δwc1	12-day	2620388	down	GO:0003824	3,60E-06	catalytic activity
Δwc1	12-day	2621873	down	GO:0003824	3,60E-06	catalytic activity
Δwc1	12-day	2622287	down	GO:0003824	3,60E-06	catalytic activity
Δwc1	12-day	2623301	down	GO:0003824	3,60E-06	catalytic activity
Δwc1	12-day	2624675	down	GO:0003824	3,60E-06	catalytic activity
Δwc1	12-day	2625561	down	GO:0003824	3,60E-06	catalytic activity
Δwc1	12-day	2625782	down	GO:0003824	3,60E-06	catalytic activity
Δwc1	12-day	2625818	down	GO:0003824	3,60E-06	catalytic activity
Δwc1	12-day	2626510	down	GO:0003824	3,60E-06	catalytic activity
Δwc1	12-day	2627304	down	GO:0003824	3,60E-06	catalytic activity
Δwc1	12-day	2628678	down	GO:0003824	3,60E-06	catalytic activity
Δwc1	12-day	2629850	down	GO:0003824	3,60E-06	catalytic activity

Δwc1	12-day	2630634	down	GO:0003824	3,60E-06	catalytic activity
Δwc1	12-day	2631112	down	GO:0003824	3,60E-06	catalytic activity
Δwc1	12-day	2631237	down	GO:0003824	3,60E-06	catalytic activity
Δwc1	12-day	2632206	down	GO:0003824	3,60E-06	catalytic activity
Δwc1	12-day	2632253	down	GO:0003824	3,60E-06	catalytic activity
Δwc1	12-day	2632611	down	GO:0003824	3,60E-06	catalytic activity
Δwc1	12-day	2633144	down	GO:0003824	3,60E-06	catalytic activity
Δwc1	12-day	2633162	down	GO:0003824	3,60E-06	catalytic activity
Δwc1	12-day	2633167	down	GO:0003824	3,60E-06	catalytic activity
Δwc1	12-day	2633632	down	GO:0003824	3,60E-06	catalytic activity
Δwc1	12-day	2634918	down	GO:0003824	3,60E-06	catalytic activity
Δwc1	12-day	2635216	down	GO:0003824	3,60E-06	catalytic activity
Δwc1	12-day	2635219	down	GO:0003824	3,60E-06	catalytic activity
Δwc1	12-day	2637104	down	GO:0003824	3,60E-06	catalytic activity
Δwc1	12-day	2638484	down	GO:0003824	3,60E-06	catalytic activity
Δwc1	12-day	2638783	down	GO:0003824	3,60E-06	catalytic activity
Δwc1	12-day	2640484	down	GO:0003824	3,60E-06	catalytic activity
Δwc1	12-day	2640992	down	GO:0003824	3,60E-06	catalytic activity
Δwc1	12-day	2641022	down	GO:0003824	3,60E-06	catalytic activity
Δwc1	12-day	2641506	down	GO:0003824	3,60E-06	catalytic activity
Δwc1	12-day	2641520	down	GO:0003824	3,60E-06	catalytic activity
Δwc1	12-day	2642275	down	GO:0003824	3,60E-06	catalytic activity
Δwc1	12-day	2643650	down	GO:0003824	3,60E-06	catalytic activity
Δwc1	12-day	2643891	down	GO:0003824	3,60E-06	catalytic activity
Δwc1	12-day	2643902	down	GO:0003824	3,60E-06	catalytic activity
Δwc1	12-day	2644281	down	GO:0003824	3,60E-06	catalytic activity
Δwc1	12-day	2644330	down	GO:0003824	3,60E-06	catalytic activity
Δwc1	12-day	2645207	down	GO:0003824	3,60E-06	catalytic activity
Δwc1	12-day	2645530	down	GO:0003824	3,60E-06	catalytic activity
Δwc1	12-day	2645668	down	GO:0003824	3,60E-06	catalytic activity
Δwc1	12-day	2660684	down	GO:0003824	3,60E-06	catalytic activity
Δwc1	12-day	2688172	down	GO:0003824	3,60E-06	catalytic activity
Δwc1	12-day	2699217	down	GO:0003824	3,60E-06	catalytic activity
Δwc1	12-day	2705421	down	GO:0003824	3,60E-06	catalytic activity

Δwc1	12-day	2711060	down	GO:0003824	3,60E-06	catalytic activity
Δwc1	12-day	1153136	down	GO:0006810	9,12E-06	transport
Δwc1	12-day	1154281	down	GO:0006810	9,12E-06	transport
Δwc1	12-day	1267603	down	GO:0006810	9,12E-06	transport
Δwc1	12-day	1357952	down	GO:0006810	9,12E-06	transport
Δwc1	12-day	2138287	down	GO:0006810	9,12E-06	transport
Δwc1	12-day	2283957	down	GO:0006810	9,12E-06	transport
Δwc1	12-day	2481653	down	GO:0006810	9,12E-06	transport
Δwc1	12-day	2492878	down	GO:0006810	9,12E-06	transport
Δwc1	12-day	2494144	down	GO:0006810	9,12E-06	transport
Δwc1	12-day	2494727	down	GO:0006810	9,12E-06	transport
Δwc1	12-day	2514012	down	GO:0006810	9,12E-06	transport
Δwc1	12-day	2516374	down	GO:0006810	9,12E-06	transport
Δwc1	12-day	2520877	down	GO:0006810	9,12E-06	transport
Δwc1	12-day	2525305	down	GO:0006810	9,12E-06	transport
Δwc1	12-day	2576922	down	GO:0006810	9,12E-06	transport
Δwc1	12-day	2607025	down	GO:0006810	9,12E-06	transport
Δwc1	12-day	2607891	down	GO:0006810	9,12E-06	transport
Δwc1	12-day	2608678	down	GO:0006810	9,12E-06	transport
Δwc1	12-day	2609987	down	GO:0006810	9,12E-06	transport
Δwc1	12-day	2612806	down	GO:0006810	9,12E-06	transport
Δwc1	12-day	2613255	down	GO:0006810	9,12E-06	transport
Δwc1	12-day	2619645	down	GO:0006810	9,12E-06	transport
Δwc1	12-day	2620178	down	GO:0006810	9,12E-06	transport
Δwc1	12-day	2621828	down	GO:0006810	9,12E-06	transport
Δwc1	12-day	2623420	down	GO:0006810	9,12E-06	transport
Δwc1	12-day	2623492	down	GO:0006810	9,12E-06	transport
Δwc1	12-day	2626803	down	GO:0006810	9,12E-06	transport
Δwc1	12-day	2627703	down	GO:0006810	9,12E-06	transport
Δwc1	12-day	2628751	down	GO:0006810	9,12E-06	transport
Δwc1	12-day	2629091	down	GO:0006810	9,12E-06	transport
Δwc1	12-day	2629455	down	GO:0006810	9,12E-06	transport
Δwc1	12-day	2634793	down	GO:0006810	9,12E-06	transport
Δwc1	12-day	2636597	down	GO:0006810	9,12E-06	transport

Δwc1	12-day	2637190	down	GO:0006810	9,12E-06	transport
Δwc1	12-day	2637509	down	GO:0006810	9,12E-06	transport
Δwc1	12-day	2642018	down	GO:0006810	9,12E-06	transport
Δwc1	12-day	2645850	down	GO:0006810	9,12E-06	transport
Δwc1	12-day	2661224	down	GO:0006810	9,12E-06	transport
Δwc1	12-day	2662585	down	GO:0006810	9,12E-06	transport
Δwc1	12-day	2701571	down	GO:0006810	9,12E-06	transport
Δwc1	12-day	2712060	down	GO:0006810	9,12E-06	transport
Δwc1	12-day	1038608	down	GO:0016020	0,000202401	membrane
Δwc1	12-day	1153136	down	GO:0016020	0,000202401	membrane
Δwc1	12-day	1176319	down	GO:0016020	0,000202401	membrane
Δwc1	12-day	1267603	down	GO:0016020	0,000202401	membrane
Δwc1	12-day	2138287	down	GO:0016020	0,000202401	membrane
Δwc1	12-day	2234784	down	GO:0016020	0,000202401	membrane
Δwc1	12-day	2283957	down	GO:0016020	0,000202401	membrane
Δwc1	12-day	2376858	down	GO:0016020	0,000202401	membrane
Δwc1	12-day	2494727	down	GO:0016020	0,000202401	membrane
Δwc1	12-day	2495286	down	GO:0016020	0,000202401	membrane
Δwc1	12-day	2514012	down	GO:0016020	0,000202401	membrane
Δwc1	12-day	2516374	down	GO:0016020	0,000202401	membrane
Δwc1	12-day	2525305	down	GO:0016020	0,000202401	membrane
Δwc1	12-day	2569923	down	GO:0016020	0,000202401	membrane
Δwc1	12-day	2600584	down	GO:0016020	0,000202401	membrane
Δwc1	12-day	2605277	down	GO:0016020	0,000202401	membrane
Δwc1	12-day	2609987	down	GO:0016020	0,000202401	membrane
Δwc1	12-day	2610450	down	GO:0016020	0,000202401	membrane
Δwc1	12-day	2612806	down	GO:0016020	0,000202401	membrane
Δwc1	12-day	2613255	down	GO:0016020	0,000202401	membrane
Δwc1	12-day	2618425	down	GO:0016020	0,000202401	membrane
Δwc1	12-day	2618479	down	GO:0016020	0,000202401	membrane
Δwc1	12-day	2618895	down	GO:0016020	0,000202401	membrane
Δwc1	12-day	2622024	down	GO:0016020	0,000202401	membrane
Δwc1	12-day	2622595	down	GO:0016020	0,000202401	membrane
Δwc1	12-day	2623420	down	GO:0016020	0,000202401	membrane

Δwc1	12-day	2627703	down	GO:0016020	0,000202401	membrane
Δwc1	12-day	2629091	down	GO:0016020	0,000202401	membrane
Δwc1	12-day	2631182	down	GO:0016020	0,000202401	membrane
Δwc1	12-day	2636160	down	GO:0016020	0,000202401	membrane
Δwc1	12-day	2638261	down	GO:0016020	0,000202401	membrane
Δwc1	12-day	2639525	down	GO:0016020	0,000202401	membrane
Δwc1	12-day	2644280	down	GO:0016020	0,000202401	membrane
Δwc1	12-day	2645850	down	GO:0016020	0,000202401	membrane
Δwc1	12-day	2701571	down	GO:0016020	0,000202401	membrane
Δwc1	12-day	78628	down	GO:0004497	0,000278598	monooxygenase activity
Δwc1	12-day	81631	down	GO:0004497	0,000278598	monooxygenase activity
Δwc1	12-day	1131542	down	GO:0004497	0,000278598	monooxygenase activity
Δwc1	12-day	1139445	down	GO:0004497	0,000278598	monooxygenase activity
Δwc1	12-day	1188937	down	GO:0004497	0,000278598	monooxygenase activity
Δwc1	12-day	1189794	down	GO:0004497	0,000278598	monooxygenase activity
Δwc1	12-day	1193647	down	GO:0004497	0,000278598	monooxygenase activity
Δwc1	12-day	2327903	down	GO:0004497	0,000278598	monooxygenase activity
Δwc1	12-day	2484868	down	GO:0004497	0,000278598	monooxygenase activity
Δwc1	12-day	2486680	down	GO:0004497	0,000278598	monooxygenase activity
Δwc1	12-day	2510129	down	GO:0004497	0,000278598	monooxygenase activity
Δwc1	12-day	2512276	down	GO:0004497	0,000278598	monooxygenase activity
Δwc1	12-day	2583618	down	GO:0004497	0,000278598	monooxygenase activity
Δwc1	12-day	2604096	down	GO:0004497	0,000278598	monooxygenase activity
Δwc1	12-day	2604231	down	GO:0004497	0,000278598	monooxygenase activity
Δwc1	12-day	2604458	down	GO:0004497	0,000278598	monooxygenase activity
Δwc1	12-day	2604735	down	GO:0004497	0,000278598	monooxygenase activity
Δwc1	12-day	2611234	down	GO:0004497	0,000278598	monooxygenase activity
Δwc1	12-day	2616301	down	GO:0004497	0,000278598	monooxygenase activity
Δwc1	12-day	2619674	down	GO:0004497	0,000278598	monooxygenase activity
Δwc1	12-day	2619840	down	GO:0004497	0,000278598	monooxygenase activity
Δwc1	12-day	2623115	down	GO:0004497	0,000278598	monooxygenase activity
Δwc1	12-day	2623122	down	GO:0004497	0,000278598	monooxygenase activity
Δwc1	12-day	2623196	down	GO:0004497	0,000278598	monooxygenase activity
Δwc1	12-day	2628312	down	GO:0004497	0,000278598	monooxygenase activity

Δwc1	12-day	2628645	down	GO:0004497	0,000278598	monooxygenase activity
Δwc1	12-day	2628651	down	GO:0004497	0,000278598	monooxygenase activity
Δwc1	12-day	2629864	down	GO:0004497	0,000278598	monooxygenase activity
Δwc1	12-day	2632925	down	GO:0004497	0,000278598	monooxygenase activity
Δwc1	12-day	2634283	down	GO:0004497	0,000278598	monooxygenase activity
Δwc1	12-day	2635487	down	GO:0004497	0,000278598	monooxygenase activity
Δwc1	12-day	2635869	down	GO:0004497	0,000278598	monooxygenase activity
Δwc1	12-day	2636544	down	GO:0004497	0,000278598	monooxygenase activity
Δwc1	12-day	2638355	down	GO:0004497	0,000278598	monooxygenase activity
Δwc1	12-day	2644430	down	GO:0004497	0,000278598	monooxygenase activity
Δwc1	12-day	2668568	down	GO:0004497	0,000278598	monooxygenase activity
Δwc1	12-day	2703628	down	GO:0004497	0,000278598	monooxygenase activity
Δwc1	12-day	2703966	down	GO:0004497	0,000278598	monooxygenase activity
Δwc1	12-day	2735179	down	GO:0004497	0,000278598	monooxygenase activity
Δwc1	12-day	1036421	down	GO:0016021	0,000325821	integral to membrane
Δwc1	12-day	1038608	down	GO:0016021	0,000325821	integral to membrane
Δwc1	12-day	1153136	down	GO:0016021	0,000325821	integral to membrane
Δwc1	12-day	1154281	down	GO:0016021	0,000325821	integral to membrane
Δwc1	12-day	1172581	down	GO:0016021	0,000325821	integral to membrane
Δwc1	12-day	1186839	down	GO:0016021	0,000325821	integral to membrane
Δwc1	12-day	2138287	down	GO:0016021	0,000325821	integral to membrane
Δwc1	12-day	2283957	down	GO:0016021	0,000325821	integral to membrane
Δwc1	12-day	2481653	down	GO:0016021	0,000325821	integral to membrane
Δwc1	12-day	2494144	down	GO:0016021	0,000325821	integral to membrane
Δwc1	12-day	2503108	down	GO:0016021	0,000325821	integral to membrane
Δwc1	12-day	2514012	down	GO:0016021	0,000325821	integral to membrane
Δwc1	12-day	2516374	down	GO:0016021	0,000325821	integral to membrane
Δwc1	12-day	2524870	down	GO:0016021	0,000325821	integral to membrane
Δwc1	12-day	2525305	down	GO:0016021	0,000325821	integral to membrane
Δwc1	12-day	2572308	down	GO:0016021	0,000325821	integral to membrane
Δwc1	12-day	2602395	down	GO:0016021	0,000325821	integral to membrane
Δwc1	12-day	2604984	down	GO:0016021	0,000325821	integral to membrane
Δwc1	12-day	2607025	down	GO:0016021	0,000325821	integral to membrane
Δwc1	12-day	2607891	down	GO:0016021	0,000325821	integral to membrane

Δwc1	12-day	2608678	down	GO:0016021	0,000325821	integral to membrane
Δwc1	12-day	2611612	down	GO:0016021	0,000325821	integral to membrane
Δwc1	12-day	2613255	down	GO:0016021	0,000325821	integral to membrane
Δwc1	12-day	2619645	down	GO:0016021	0,000325821	integral to membrane
Δwc1	12-day	2620178	down	GO:0016021	0,000325821	integral to membrane
Δwc1	12-day	2621828	down	GO:0016021	0,000325821	integral to membrane
Δwc1	12-day	2623420	down	GO:0016021	0,000325821	integral to membrane
Δwc1	12-day	2623492	down	GO:0016021	0,000325821	integral to membrane
Δwc1	12-day	2626803	down	GO:0016021	0,000325821	integral to membrane
Δwc1	12-day	2627703	down	GO:0016021	0,000325821	integral to membrane
Δwc1	12-day	2629091	down	GO:0016021	0,000325821	integral to membrane
Δwc1	12-day	2629455	down	GO:0016021	0,000325821	integral to membrane
Δwc1	12-day	2636597	down	GO:0016021	0,000325821	integral to membrane
Δwc1	12-day	2637190	down	GO:0016021	0,000325821	integral to membrane
Δwc1	12-day	2637509	down	GO:0016021	0,000325821	integral to membrane
Δwc1	12-day	2638261	down	GO:0016021	0,000325821	integral to membrane
Δwc1	12-day	2642018	down	GO:0016021	0,000325821	integral to membrane
Δwc1	12-day	2667661	down	GO:0016021	0,000325821	integral to membrane
Δwc1	12-day	2686473	down	GO:0016021	0,000325821	integral to membrane
Δwc1	12-day	2691538	down	GO:0016021	0,000325821	integral to membrane
Δwc1	12-day	2701571	down	GO:0016021	0,000325821	integral to membrane
Δwc1	12-day	2704867	down	GO:0016021	0,000325821	integral to membrane
Δwc1	12-day	2712060	down	GO:0016021	0,000325821	integral to membrane
Δwc1	12-day	78628	down	GO:0006118	0,000581667	electron transport
Δwc1	12-day	81631	down	GO:0006118	0,000581667	electron transport
Δwc1	12-day	1038608	down	GO:0006118	0,000581667	electron transport
Δwc1	12-day	1084300	down	GO:0006118	0,000581667	electron transport
Δwc1	12-day	1131542	down	GO:0006118	0,000581667	electron transport
Δwc1	12-day	1139445	down	GO:0006118	0,000581667	electron transport
Δwc1	12-day	1188937	down	GO:0006118	0,000581667	electron transport
Δwc1	12-day	1189794	down	GO:0006118	0,000581667	electron transport
Δwc1	12-day	1193647	down	GO:0006118	0,000581667	electron transport
Δwc1	12-day	2327903	down	GO:0006118	0,000581667	electron transport
Δwc1	12-day	2450442	down	GO:0006118	0,000581667	electron transport

Δwc1	12-day	2484868	down	GO:0006118	0,000581667	electron transport
Δwc1	12-day	2486680	down	GO:0006118	0,000581667	electron transport
Δwc1	12-day	2490518	down	GO:0006118	0,000581667	electron transport
Δwc1	12-day	2497674	down	GO:0006118	0,000581667	electron transport
Δwc1	12-day	2503108	down	GO:0006118	0,000581667	electron transport
Δwc1	12-day	2510129	down	GO:0006118	0,000581667	electron transport
Δwc1	12-day	2512276	down	GO:0006118	0,000581667	electron transport
Δwc1	12-day	2532967	down	GO:0006118	0,000581667	electron transport
Δwc1	12-day	2571870	down	GO:0006118	0,000581667	electron transport
Δwc1	12-day	2583618	down	GO:0006118	0,000581667	electron transport
Δwc1	12-day	2604096	down	GO:0006118	0,000581667	electron transport
Δwc1	12-day	2604231	down	GO:0006118	0,000581667	electron transport
Δwc1	12-day	2604458	down	GO:0006118	0,000581667	electron transport
Δwc1	12-day	2604628	down	GO:0006118	0,000581667	electron transport
Δwc1	12-day	2604735	down	GO:0006118	0,000581667	electron transport
Δwc1	12-day	2609840	down	GO:0006118	0,000581667	electron transport
Δwc1	12-day	2611234	down	GO:0006118	0,000581667	electron transport
Δwc1	12-day	2616301	down	GO:0006118	0,000581667	electron transport
Δwc1	12-day	2619674	down	GO:0006118	0,000581667	electron transport
Δwc1	12-day	2619840	down	GO:0006118	0,000581667	electron transport
Δwc1	12-day	2621855	down	GO:0006118	0,000581667	electron transport
Δwc1	12-day	2623115	down	GO:0006118	0,000581667	electron transport
Δwc1	12-day	2623122	down	GO:0006118	0,000581667	electron transport
Δwc1	12-day	2623196	down	GO:0006118	0,000581667	electron transport
Δwc1	12-day	2625951	down	GO:0006118	0,000581667	electron transport
Δwc1	12-day	2628312	down	GO:0006118	0,000581667	electron transport
Δwc1	12-day	2628645	down	GO:0006118	0,000581667	electron transport
Δwc1	12-day	2628651	down	GO:0006118	0,000581667	electron transport
Δwc1	12-day	2629850	down	GO:0006118	0,000581667	electron transport
Δwc1	12-day	2629864	down	GO:0006118	0,000581667	electron transport
Δwc1	12-day	2632925	down	GO:0006118	0,000581667	electron transport
Δwc1	12-day	2633167	down	GO:0006118	0,000581667	electron transport
Δwc1	12-day	2634283	down	GO:0006118	0,000581667	electron transport
Δwc1	12-day	2635487	down	GO:0006118	0,000581667	electron transport

Δwc1	12-day	2635869	down	GO:0006118	0,000581667	electron transport
Δwc1	12-day	2636544	down	GO:0006118	0,000581667	electron transport
Δwc1	12-day	2637104	down	GO:0006118	0,000581667	electron transport
Δwc1	12-day	2637190	down	GO:0006118	0,000581667	electron transport
Δwc1	12-day	2638355	down	GO:0006118	0,000581667	electron transport
Δwc1	12-day	2639682	down	GO:0006118	0,000581667	electron transport
Δwc1	12-day	2644430	down	GO:0006118	0,000581667	electron transport
Δwc1	12-day	2662585	down	GO:0006118	0,000581667	electron transport
Δwc1	12-day	2668568	down	GO:0006118	0,000581667	electron transport
Δwc1	12-day	2671376	down	GO:0006118	0,000581667	electron transport
Δwc1	12-day	2703628	down	GO:0006118	0,000581667	electron transport
Δwc1	12-day	2703966	down	GO:0006118	0,000581667	electron transport
Δwc1	12-day	2735179	down	GO:0006118	0,000581667	electron transport
Δwc1	12-day	1153136	down	GO:0005215	0,004404998	transporter activity
Δwc1	12-day	1154281	down	GO:0005215	0,004404998	transporter activity
Δwc1	12-day	1357952	down	GO:0005215	0,004404998	transporter activity
Δwc1	12-day	2138287	down	GO:0005215	0,004404998	transporter activity
Δwc1	12-day	2283957	down	GO:0005215	0,004404998	transporter activity
Δwc1	12-day	2481653	down	GO:0005215	0,004404998	transporter activity
Δwc1	12-day	2492878	down	GO:0005215	0,004404998	transporter activity
Δwc1	12-day	2494144	down	GO:0005215	0,004404998	transporter activity
Δwc1	12-day	2516374	down	GO:0005215	0,004404998	transporter activity
Δwc1	12-day	2520877	down	GO:0005215	0,004404998	transporter activity
Δwc1	12-day	2525305	down	GO:0005215	0,004404998	transporter activity
Δwc1	12-day	2607025	down	GO:0005215	0,004404998	transporter activity
Δwc1	12-day	2607891	down	GO:0005215	0,004404998	transporter activity
Δwc1	12-day	2608678	down	GO:0005215	0,004404998	transporter activity
Δwc1	12-day	2613255	down	GO:0005215	0,004404998	transporter activity
Δwc1	12-day	2619645	down	GO:0005215	0,004404998	transporter activity
Δwc1	12-day	2620178	down	GO:0005215	0,004404998	transporter activity
Δwc1	12-day	2621828	down	GO:0005215	0,004404998	transporter activity
Δwc1	12-day	2623420	down	GO:0005215	0,004404998	transporter activity
Δwc1	12-day	2623492	down	GO:0005215	0,004404998	transporter activity
Δwc1	12-day	2626803	down	GO:0005215	0,004404998	transporter activity

Δwc1	12-day	2627703	down	GO:0005215	0,004404998	transporter activity
Δwc1	12-day	2628751	down	GO:0005215	0,004404998	transporter activity
Δwc1	12-day	2629091	down	GO:0005215	0,004404998	transporter activity
Δwc1	12-day	2629455	down	GO:0005215	0,004404998	transporter activity
Δwc1	12-day	2634793	down	GO:0005215	0,004404998	transporter activity
Δwc1	12-day	2636597	down	GO:0005215	0,004404998	transporter activity
Δwc1	12-day	2637509	down	GO:0005215	0,004404998	transporter activity
Δwc1	12-day	2642018	down	GO:0005215	0,004404998	transporter activity
Δwc1	12-day	2661224	down	GO:0005215	0,004404998	transporter activity
Δwc1	12-day	2701571	down	GO:0005215	0,004404998	transporter activity
Δwc1	12-day	2712060	down	GO:0005215	0,004404998	transporter activity
Δwc1	12-day	1152293	down	GO:0005488	0,009467052	binding
Δwc1	12-day	1267603	down	GO:0005488	0,009467052	binding
Δwc1	12-day	1357952	down	GO:0005488	0,009467052	binding
Δwc1	12-day	2085050	down	GO:0005488	0,009467052	binding
Δwc1	12-day	2135332	down	GO:0005488	0,009467052	binding
Δwc1	12-day	2161304	down	GO:0005488	0,009467052	binding
Δwc1	12-day	2194185	down	GO:0005488	0,009467052	binding
Δwc1	12-day	2255031	down	GO:0005488	0,009467052	binding
Δwc1	12-day	2333133	down	GO:0005488	0,009467052	binding
Δwc1	12-day	2481387	down	GO:0005488	0,009467052	binding
Δwc1	12-day	2492878	down	GO:0005488	0,009467052	binding
Δwc1	12-day	2494727	down	GO:0005488	0,009467052	binding
Δwc1	12-day	2520877	down	GO:0005488	0,009467052	binding
Δwc1	12-day	2606988	down	GO:0005488	0,009467052	binding
Δwc1	12-day	2608048	down	GO:0005488	0,009467052	binding
Δwc1	12-day	2610372	down	GO:0005488	0,009467052	binding
Δwc1	12-day	2610587	down	GO:0005488	0,009467052	binding
Δwc1	12-day	2612806	down	GO:0005488	0,009467052	binding
Δwc1	12-day	2616880	down	GO:0005488	0,009467052	binding
Δwc1	12-day	2616916	down	GO:0005488	0,009467052	binding
Δwc1	12-day	2618904	down	GO:0005488	0,009467052	binding
Δwc1	12-day	2619714	down	GO:0005488	0,009467052	binding
Δwc1	12-day	2621873	down	GO:0005488	0,009467052	binding

Δwc1	12-day	2623301	down	GO:0005488	0,009467052	binding
Δwc1	12-day	2624675	down	GO:0005488	0,009467052	binding
Δwc1	12-day	2625561	down	GO:0005488	0,009467052	binding
Δwc1	12-day	2625782	down	GO:0005488	0,009467052	binding
Δwc1	12-day	2626510	down	GO:0005488	0,009467052	binding
Δwc1	12-day	2627304	down	GO:0005488	0,009467052	binding
Δwc1	12-day	2628751	down	GO:0005488	0,009467052	binding
Δwc1	12-day	2629850	down	GO:0005488	0,009467052	binding
Δwc1	12-day	2631237	down	GO:0005488	0,009467052	binding
Δwc1	12-day	2632611	down	GO:0005488	0,009467052	binding
Δwc1	12-day	2633167	down	GO:0005488	0,009467052	binding
Δwc1	12-day	2633632	down	GO:0005488	0,009467052	binding
Δwc1	12-day	2634793	down	GO:0005488	0,009467052	binding
Δwc1	12-day	2637104	down	GO:0005488	0,009467052	binding
Δwc1	12-day	2638484	down	GO:0005488	0,009467052	binding
Δwc1	12-day	2641506	down	GO:0005488	0,009467052	binding
Δwc1	12-day	2641520	down	GO:0005488	0,009467052	binding
Δwc1	12-day	2644281	down	GO:0005488	0,009467052	binding
Δwc1	12-day	2644330	down	GO:0005488	0,009467052	binding
Δwc1	12-day	2645850	down	GO:0005488	0,009467052	binding
Δwc1	12-day	2660684	down	GO:0005488	0,009467052	binding
Δwc1	12-day	2661224	down	GO:0005488	0,009467052	binding
Δwc1	12-day	2705421	down	GO:0005488	0,009467052	binding
Δwc1	12-day	2711060	down	GO:0005488	0,009467052	binding
Δwc1	12-day	2744418	down	GO:0005488	0,009467052	binding
Δwc1	12-day	2285680	down	GO:0006629	0,009467052	lipid metabolic process
Δwc1	12-day	2376858	down	GO:0006629	0,009467052	lipid metabolic process
Δwc1	12-day	2636160	down	GO:0006629	0,009467052	lipid metabolic process
Δwc1	12-day	2641759	down	GO:0006629	0,009467052	lipid metabolic process
Δwc1	12-day	2493870	down	GO:0042626	0,014036571	ATPase activity, coupled to transmembrane movement of substances
Δwc1	12-day	2614908	down	GO:0042626	0,014036571	ATPase activity, coupled to transmembrane movement of substances
Δwc1	12-day	2637190	down	GO:0042626	0,014036571	ATPase activity, coupled to transmembrane movement of

						substances
Δwc1	12-day	2628225	down	GO:0005199	0,016634694	structural constituent of cell wall
Δwc1	12-day	2629603	down	GO:0005199	0,016634694	structural constituent of cell wall
Δwc1	12-day	2746250	down	GO:0005199	0,016634694	structural constituent of cell wall
Δwc1	12-day	59565	down	GO:0008843	0,016634694	endochitinase activity
Δwc1	12-day	2520104	down	GO:0008843	0,016634694	endochitinase activity
Δwc1	12-day	2623305	down	GO:0008843	0,016634694	endochitinase activity
Δwc1	12-day	2633726	down	GO:0008843	0,016634694	endochitinase activity
Δwc1	12-day	2706631	down	GO:0008843	0,016634694	endochitinase activity
Δwc1	12-day	78628	down	GO:0020037	0,016634694	heme binding
Δwc1	12-day	81631	down	GO:0020037	0,016634694	heme binding
Δwc1	12-day	1131542	down	GO:0020037	0,016634694	heme binding
Δwc1	12-day	1139445	down	GO:0020037	0,016634694	heme binding
Δwc1	12-day	1188937	down	GO:0020037	0,016634694	heme binding
Δwc1	12-day	1189794	down	GO:0020037	0,016634694	heme binding
Δwc1	12-day	1193647	down	GO:0020037	0,016634694	heme binding
Δwc1	12-day	2327903	down	GO:0020037	0,016634694	heme binding
Δwc1	12-day	2376858	down	GO:0020037	0,016634694	heme binding
Δwc1	12-day	2484868	down	GO:0020037	0,016634694	heme binding
Δwc1	12-day	2486680	down	GO:0020037	0,016634694	heme binding
Δwc1	12-day	2497674	down	GO:0020037	0,016634694	heme binding
Δwc1	12-day	2510129	down	GO:0020037	0,016634694	heme binding
Δwc1	12-day	2512276	down	GO:0020037	0,016634694	heme binding
Δwc1	12-day	2583618	down	GO:0020037	0,016634694	heme binding
Δwc1	12-day	2604096	down	GO:0020037	0,016634694	heme binding
Δwc1	12-day	2604231	down	GO:0020037	0,016634694	heme binding
Δwc1	12-day	2604458	down	GO:0020037	0,016634694	heme binding
Δwc1	12-day	2604628	down	GO:0020037	0,016634694	heme binding
Δwc1	12-day	2604735	down	GO:0020037	0,016634694	heme binding
Δwc1	12-day	2609840	down	GO:0020037	0,016634694	heme binding
Δwc1	12-day	2611234	down	GO:0020037	0,016634694	heme binding
Δwc1	12-day	2616301	down	GO:0020037	0,016634694	heme binding
Δwc1	12-day	2619674	down	GO:0020037	0,016634694	heme binding
Δwc1	12-day	2621855	down	GO:0020037	0,016634694	heme binding

Δwc1	12-day	2623115	down	GO:0020037	0,016634694	heme binding
Δwc1	12-day	2623122	down	GO:0020037	0,016634694	heme binding
Δwc1	12-day	2623196	down	GO:0020037	0,016634694	heme binding
Δwc1	12-day	2624504	down	GO:0020037	0,016634694	heme binding
Δwc1	12-day	2628312	down	GO:0020037	0,016634694	heme binding
Δwc1	12-day	2628645	down	GO:0020037	0,016634694	heme binding
Δwc1	12-day	2628651	down	GO:0020037	0,016634694	heme binding
Δwc1	12-day	2629864	down	GO:0020037	0,016634694	heme binding
Δwc1	12-day	2632925	down	GO:0020037	0,016634694	heme binding
Δwc1	12-day	2634283	down	GO:0020037	0,016634694	heme binding
Δwc1	12-day	2635487	down	GO:0020037	0,016634694	heme binding
Δwc1	12-day	2635869	down	GO:0020037	0,016634694	heme binding
Δwc1	12-day	2636160	down	GO:0020037	0,016634694	heme binding
Δwc1	12-day	2636544	down	GO:0020037	0,016634694	heme binding
Δwc1	12-day	2637190	down	GO:0020037	0,016634694	heme binding
Δwc1	12-day	2638355	down	GO:0020037	0,016634694	heme binding
Δwc1	12-day	2668568	down	GO:0020037	0,016634694	heme binding
Δwc1	12-day	2703628	down	GO:0020037	0,016634694	heme binding
Δwc1	12-day	2703966	down	GO:0020037	0,016634694	heme binding
Δwc1	12-day	2735179	down	GO:0020037	0,016634694	heme binding
Δwc1	12-day	59565	down	GO:0004568	0,016634694	chitinase activity
Δwc1	12-day	2520104	down	GO:0004568	0,016634694	chitinase activity
Δwc1	12-day	2623305	down	GO:0004568	0,016634694	chitinase activity
Δwc1	12-day	2633726	down	GO:0004568	0,016634694	chitinase activity
Δwc1	12-day	2706631	down	GO:0004568	0,016634694	chitinase activity
Δwc1	12-day	2614257	down	GO:0005618	0,019769883	cell wall
Δwc1	12-day	2628225	down	GO:0005618	0,019769883	cell wall
Δwc1	12-day	2629603	down	GO:0005618	0,019769883	cell wall
Δwc1	12-day	2577992	down	GO:0004194	0,020981612	pepsin A activity
Δwc1	12-day	2613917	down	GO:0004194	0,020981612	pepsin A activity
Δwc1	12-day	2616949	down	GO:0004194	0,020981612	pepsin A activity
Δwc1	12-day	2616991	down	GO:0004194	0,020981612	pepsin A activity
Δwc1	12-day	2619340	down	GO:0004194	0,020981612	pepsin A activity
Δwc1	12-day	2619343	down	GO:0004194	0,020981612	pepsin A activity

Δwc2	12-day	1083109	up	GO:0020037	7,28E-10	heme binding
Δwc2	12-day	1112937	up	GO:0020037	7,28E-10	heme binding
Δwc2	12-day	1120318	up	GO:0020037	7,28E-10	heme binding
Δwc2	12-day	1147397	up	GO:0020037	7,28E-10	heme binding
Δwc2	12-day	2075136	up	GO:0020037	7,28E-10	heme binding
Δwc2	12-day	2364606	up	GO:0020037	7,28E-10	heme binding
Δwc2	12-day	2491624	up	GO:0020037	7,28E-10	heme binding
Δwc2	12-day	2501258	up	GO:0020037	7,28E-10	heme binding
Δwc2	12-day	2514636	up	GO:0020037	7,28E-10	heme binding
Δwc2	12-day	2537529	up	GO:0020037	7,28E-10	heme binding
Δwc2	12-day	2547557	up	GO:0020037	7,28E-10	heme binding
Δwc2	12-day	2559987	up	GO:0020037	7,28E-10	heme binding
Δwc2	12-day	2583399	up	GO:0020037	7,28E-10	heme binding
Δwc2	12-day	2608091	up	GO:0020037	7,28E-10	heme binding
Δwc2	12-day	2619716	up	GO:0020037	7,28E-10	heme binding
Δwc2	12-day	2620407	up	GO:0020037	7,28E-10	heme binding
Δwc2	12-day	2622782	up	GO:0020037	7,28E-10	heme binding
Δwc2	12-day	2625023	up	GO:0020037	7,28E-10	heme binding
Δwc2	12-day	2625041	up	GO:0020037	7,28E-10	heme binding
Δwc2	12-day	2626841	up	GO:0020037	7,28E-10	heme binding
Δwc2	12-day	2628300	up	GO:0020037	7,28E-10	heme binding
Δwc2	12-day	2635949	up	GO:0020037	7,28E-10	heme binding
Δwc2	12-day	2637467	up	GO:0020037	7,28E-10	heme binding
Δwc2	12-day	2638350	up	GO:0020037	7,28E-10	heme binding
Δwc2	12-day	2640585	up	GO:0020037	7,28E-10	heme binding
Δwc2	12-day	2643274	up	GO:0020037	7,28E-10	heme binding
Δwc2	12-day	2666440	up	GO:0020037	7,28E-10	heme binding
Δwc2	12-day	2673121	up	GO:0020037	7,28E-10	heme binding
Δwc2	12-day	2695849	up	GO:0020037	7,28E-10	heme binding
Δwc2	12-day	1083109	up	GO:0005506	7,28E-10	iron ion binding
Δwc2	12-day	1120318	up	GO:0005506	7,28E-10	iron ion binding
Δwc2	12-day	1147397	up	GO:0005506	7,28E-10	iron ion binding
Δwc2	12-day	2075136	up	GO:0005506	7,28E-10	iron ion binding
Δwc2	12-day	2364606	up	GO:0005506	7,28E-10	iron ion binding

Δwc2	12-day	2491624	up	GO:0005506	7,28E-10	iron ion binding
Δwc2	12-day	2501258	up	GO:0005506	7,28E-10	iron ion binding
Δwc2	12-day	2514636	up	GO:0005506	7,28E-10	iron ion binding
Δwc2	12-day	2537529	up	GO:0005506	7,28E-10	iron ion binding
Δwc2	12-day	2547557	up	GO:0005506	7,28E-10	iron ion binding
Δwc2	12-day	2559987	up	GO:0005506	7,28E-10	iron ion binding
Δwc2	12-day	2619716	up	GO:0005506	7,28E-10	iron ion binding
Δwc2	12-day	2620407	up	GO:0005506	7,28E-10	iron ion binding
Δwc2	12-day	2622782	up	GO:0005506	7,28E-10	iron ion binding
Δwc2	12-day	2625023	up	GO:0005506	7,28E-10	iron ion binding
Δwc2	12-day	2626841	up	GO:0005506	7,28E-10	iron ion binding
Δwc2	12-day	2628300	up	GO:0005506	7,28E-10	iron ion binding
Δwc2	12-day	2635949	up	GO:0005506	7,28E-10	iron ion binding
Δwc2	12-day	2637467	up	GO:0005506	7,28E-10	iron ion binding
Δwc2	12-day	2638350	up	GO:0005506	7,28E-10	iron ion binding
Δwc2	12-day	2666440	up	GO:0005506	7,28E-10	iron ion binding
Δwc2	12-day	2695849	up	GO:0005506	7,28E-10	iron ion binding
Δwc2	12-day	1120318	up	GO:0004497	1,12E-08	monooxygenase activity
Δwc2	12-day	1147397	up	GO:0004497	1,12E-08	monooxygenase activity
Δwc2	12-day	1168026	up	GO:0004497	1,12E-08	monooxygenase activity
Δwc2	12-day	1342670	up	GO:0004497	1,12E-08	monooxygenase activity
Δwc2	12-day	2213880	up	GO:0004497	1,12E-08	monooxygenase activity
Δwc2	12-day	2364606	up	GO:0004497	1,12E-08	monooxygenase activity
Δwc2	12-day	2490194	up	GO:0004497	1,12E-08	monooxygenase activity
Δwc2	12-day	2491624	up	GO:0004497	1,12E-08	monooxygenase activity
Δwc2	12-day	2501258	up	GO:0004497	1,12E-08	monooxygenase activity
Δwc2	12-day	2514636	up	GO:0004497	1,12E-08	monooxygenase activity
Δwc2	12-day	2537529	up	GO:0004497	1,12E-08	monooxygenase activity
Δwc2	12-day	2547557	up	GO:0004497	1,12E-08	monooxygenase activity
Δwc2	12-day	2559987	up	GO:0004497	1,12E-08	monooxygenase activity
Δwc2	12-day	2573393	up	GO:0004497	1,12E-08	monooxygenase activity
Δwc2	12-day	2573396	up	GO:0004497	1,12E-08	monooxygenase activity
Δwc2	12-day	2619716	up	GO:0004497	1,12E-08	monooxygenase activity
Δwc2	12-day	2620407	up	GO:0004497	1,12E-08	monooxygenase activity

Δwc2	12-day	2622782	up	GO:0004497	1,12E-08	monooxygenase activity
Δwc2	12-day	2625023	up	GO:0004497	1,12E-08	monooxygenase activity
Δwc2	12-day	2626841	up	GO:0004497	1,12E-08	monooxygenase activity
Δwc2	12-day	2628300	up	GO:0004497	1,12E-08	monooxygenase activity
Δwc2	12-day	2635949	up	GO:0004497	1,12E-08	monooxygenase activity
Δwc2	12-day	2637467	up	GO:0004497	1,12E-08	monooxygenase activity
Δwc2	12-day	2638350	up	GO:0004497	1,12E-08	monooxygenase activity
Δwc2	12-day	2666440	up	GO:0004497	1,12E-08	monooxygenase activity
Δwc2	12-day	2695849	up	GO:0004497	1,12E-08	monooxygenase activity
Δwc2	12-day	2703927	up	GO:0004497	1,12E-08	monooxygenase activity
Δwc2	12-day	1120318	up	GO:0050381	9,26E-08	unspecific monooxygenase activity
Δwc2	12-day	2491624	up	GO:0050381	9,26E-08	unspecific monooxygenase activity
Δwc2	12-day	2514636	up	GO:0050381	9,26E-08	unspecific monooxygenase activity
Δwc2	12-day	2547557	up	GO:0050381	9,26E-08	unspecific monooxygenase activity
Δwc2	12-day	2559987	up	GO:0050381	9,26E-08	unspecific monooxygenase activity
Δwc2	12-day	2619716	up	GO:0050381	9,26E-08	unspecific monooxygenase activity
Δwc2	12-day	2620407	up	GO:0050381	9,26E-08	unspecific monooxygenase activity
Δwc2	12-day	2625023	up	GO:0050381	9,26E-08	unspecific monooxygenase activity
Δwc2	12-day	2637467	up	GO:0050381	9,26E-08	unspecific monooxygenase activity
Δwc2	12-day	2638350	up	GO:0050381	9,26E-08	unspecific monooxygenase activity
Δwc2	12-day	2666440	up	GO:0050381	9,26E-08	unspecific monooxygenase activity
Δwc2	12-day	1112937	up	GO:0006118	1,35E-07	electron transport
Δwc2	12-day	1120318	up	GO:0006118	1,35E-07	electron transport
Δwc2	12-day	1147397	up	GO:0006118	1,35E-07	electron transport
Δwc2	12-day	1168026	up	GO:0006118	1,35E-07	electron transport
Δwc2	12-day	1215660	up	GO:0006118	1,35E-07	electron transport
Δwc2	12-day	1342670	up	GO:0006118	1,35E-07	electron transport
Δwc2	12-day	2075136	up	GO:0006118	1,35E-07	electron transport
Δwc2	12-day	2213880	up	GO:0006118	1,35E-07	electron transport
Δwc2	12-day	2364606	up	GO:0006118	1,35E-07	electron transport
Δwc2	12-day	2457440	up	GO:0006118	1,35E-07	electron transport
Δwc2	12-day	2490194	up	GO:0006118	1,35E-07	electron transport
Δwc2	12-day	2491624	up	GO:0006118	1,35E-07	electron transport
Δwc2	12-day	2501258	up	GO:0006118	1,35E-07	electron transport

Δwc2	12-day	2505277	up	GO:0006118	1,35E-07	electron transport
Δwc2	12-day	2508276	up	GO:0006118	1,35E-07	electron transport
Δwc2	12-day	2514636	up	GO:0006118	1,35E-07	electron transport
Δwc2	12-day	2525011	up	GO:0006118	1,35E-07	electron transport
Δwc2	12-day	2537529	up	GO:0006118	1,35E-07	electron transport
Δwc2	12-day	2547557	up	GO:0006118	1,35E-07	electron transport
Δwc2	12-day	2559987	up	GO:0006118	1,35E-07	electron transport
Δwc2	12-day	2573393	up	GO:0006118	1,35E-07	electron transport
Δwc2	12-day	2573396	up	GO:0006118	1,35E-07	electron transport
Δwc2	12-day	2576903	up	GO:0006118	1,35E-07	electron transport
Δwc2	12-day	2583399	up	GO:0006118	1,35E-07	electron transport
Δwc2	12-day	2594205	up	GO:0006118	1,35E-07	electron transport
Δwc2	12-day	2605889	up	GO:0006118	1,35E-07	electron transport
Δwc2	12-day	2608235	up	GO:0006118	1,35E-07	electron transport
Δwc2	12-day	2615184	up	GO:0006118	1,35E-07	electron transport
Δwc2	12-day	2616557	up	GO:0006118	1,35E-07	electron transport
Δwc2	12-day	2619716	up	GO:0006118	1,35E-07	electron transport
Δwc2	12-day	2620407	up	GO:0006118	1,35E-07	electron transport
Δwc2	12-day	2622733	up	GO:0006118	1,35E-07	electron transport
Δwc2	12-day	2622782	up	GO:0006118	1,35E-07	electron transport
Δwc2	12-day	2625023	up	GO:0006118	1,35E-07	electron transport
Δwc2	12-day	2625041	up	GO:0006118	1,35E-07	electron transport
Δwc2	12-day	2626841	up	GO:0006118	1,35E-07	electron transport
Δwc2	12-day	2628120	up	GO:0006118	1,35E-07	electron transport
Δwc2	12-day	2628300	up	GO:0006118	1,35E-07	electron transport
Δwc2	12-day	2635949	up	GO:0006118	1,35E-07	electron transport
Δwc2	12-day	2637467	up	GO:0006118	1,35E-07	electron transport
Δwc2	12-day	2638350	up	GO:0006118	1,35E-07	electron transport
Δwc2	12-day	2638473	up	GO:0006118	1,35E-07	electron transport
Δwc2	12-day	2643274	up	GO:0006118	1,35E-07	electron transport
Δwc2	12-day	2666009	up	GO:0006118	1,35E-07	electron transport
Δwc2	12-day	2666440	up	GO:0006118	1,35E-07	electron transport
Δwc2	12-day	2667289	up	GO:0006118	1,35E-07	electron transport
Δwc2	12-day	2695849	up	GO:0006118	1,35E-07	electron transport

Δwc2	12-day	2698211	up	GO:0006118	1,35E-07	electron transport
Δwc2	12-day	2703927	up	GO:0006118	1,35E-07	electron transport
Δwc2	12-day	66483	up	GO:0005975	4,93E-07	carbohydrate metabolic process
Δwc2	12-day	85210	up	GO:0005975	4,93E-07	carbohydrate metabolic process
Δwc2	12-day	234329	up	GO:0005975	4,93E-07	carbohydrate metabolic process
Δwc2	12-day	2486953	up	GO:0005975	4,93E-07	carbohydrate metabolic process
Δwc2	12-day	2493241	up	GO:0005975	4,93E-07	carbohydrate metabolic process
Δwc2	12-day	2514546	up	GO:0005975	4,93E-07	carbohydrate metabolic process
Δwc2	12-day	2570936	up	GO:0005975	4,93E-07	carbohydrate metabolic process
Δwc2	12-day	2607436	up	GO:0005975	4,93E-07	carbohydrate metabolic process
Δwc2	12-day	2613657	up	GO:0005975	4,93E-07	carbohydrate metabolic process
Δwc2	12-day	2621806	up	GO:0005975	4,93E-07	carbohydrate metabolic process
Δwc2	12-day	2622563	up	GO:0005975	4,93E-07	carbohydrate metabolic process
Δwc2	12-day	2624823	up	GO:0005975	4,93E-07	carbohydrate metabolic process
Δwc2	12-day	2628008	up	GO:0005975	4,93E-07	carbohydrate metabolic process
Δwc2	12-day	2631621	up	GO:0005975	4,93E-07	carbohydrate metabolic process
Δwc2	12-day	2633791	up	GO:0005975	4,93E-07	carbohydrate metabolic process
Δwc2	12-day	2637065	up	GO:0005975	4,93E-07	carbohydrate metabolic process
Δwc2	12-day	2641020	up	GO:0005975	4,93E-07	carbohydrate metabolic process
Δwc2	12-day	2642958	up	GO:0005975	4,93E-07	carbohydrate metabolic process
Δwc2	12-day	2643740	up	GO:0005975	4,93E-07	carbohydrate metabolic process
Δwc2	12-day	2644666	up	GO:0005975	4,93E-07	carbohydrate metabolic process
Δwc2	12-day	2670422	up	GO:0005975	4,93E-07	carbohydrate metabolic process
Δwc2	12-day	2686544	up	GO:0005975	4,93E-07	carbohydrate metabolic process
Δwc2	12-day	85210	up	GO:0004553	2,01E-05	hydrolase activity, hydrolyzing O-glycosyl compounds
Δwc2	12-day	234329	up	GO:0004553	2,01E-05	hydrolase activity, hydrolyzing O-glycosyl compounds
Δwc2	12-day	2486953	up	GO:0004553	2,01E-05	hydrolase activity, hydrolyzing O-glycosyl compounds
Δwc2	12-day	2613657	up	GO:0004553	2,01E-05	hydrolase activity, hydrolyzing O-glycosyl compounds
Δwc2	12-day	2621806	up	GO:0004553	2,01E-05	hydrolase activity, hydrolyzing O-glycosyl compounds
Δwc2	12-day	2622563	up	GO:0004553	2,01E-05	hydrolase activity, hydrolyzing O-glycosyl compounds
Δwc2	12-day	2637065	up	GO:0004553	2,01E-05	hydrolase activity, hydrolyzing O-glycosyl compounds
Δwc2	12-day	2642958	up	GO:0004553	2,01E-05	hydrolase activity, hydrolyzing O-glycosyl compounds
Δwc2	12-day	2643740	up	GO:0004553	2,01E-05	hydrolase activity, hydrolyzing O-glycosyl compounds
Δwc2	12-day	2644666	up	GO:0004553	2,01E-05	hydrolase activity, hydrolyzing O-glycosyl compounds

Δwc2	12-day	2670422	up	GO:0004553	2,01E-05	hydrolase activity, hydrolyzing O-glycosyl compounds
Δwc2	12-day	2686544	up	GO:0004553	2,01E-05	hydrolase activity, hydrolyzing O-glycosyl compounds
Δwc2	12-day	1136736	up	GO:0005215	0,000853245	transporter activity
Δwc2	12-day	1171024	up	GO:0005215	0,000853245	transporter activity
Δwc2	12-day	1234296	up	GO:0005215	0,000853245	transporter activity
Δwc2	12-day	2481323	up	GO:0005215	0,000853245	transporter activity
Δwc2	12-day	2498026	up	GO:0005215	0,000853245	transporter activity
Δwc2	12-day	2499364	up	GO:0005215	0,000853245	transporter activity
Δwc2	12-day	2502284	up	GO:0005215	0,000853245	transporter activity
Δwc2	12-day	2508421	up	GO:0005215	0,000853245	transporter activity
Δwc2	12-day	2550496	up	GO:0005215	0,000853245	transporter activity
Δwc2	12-day	2564524	up	GO:0005215	0,000853245	transporter activity
Δwc2	12-day	2612313	up	GO:0005215	0,000853245	transporter activity
Δwc2	12-day	2617636	up	GO:0005215	0,000853245	transporter activity
Δwc2	12-day	2618842	up	GO:0005215	0,000853245	transporter activity
Δwc2	12-day	2619010	up	GO:0005215	0,000853245	transporter activity
Δwc2	12-day	2622931	up	GO:0005215	0,000853245	transporter activity
Δwc2	12-day	2623043	up	GO:0005215	0,000853245	transporter activity
Δwc2	12-day	2625041	up	GO:0005215	0,000853245	transporter activity
Δwc2	12-day	2628098	up	GO:0005215	0,000853245	transporter activity
Δwc2	12-day	2628982	up	GO:0005215	0,000853245	transporter activity
Δwc2	12-day	2630257	up	GO:0005215	0,000853245	transporter activity
Δwc2	12-day	2631348	up	GO:0005215	0,000853245	transporter activity
Δwc2	12-day	2632105	up	GO:0005215	0,000853245	transporter activity
Δwc2	12-day	2633206	up	GO:0005215	0,000853245	transporter activity
Δwc2	12-day	2633212	up	GO:0005215	0,000853245	transporter activity
Δwc2	12-day	2633596	up	GO:0005215	0,000853245	transporter activity
Δwc2	12-day	2637652	up	GO:0005215	0,000853245	transporter activity
Δwc2	12-day	2641095	up	GO:0005215	0,000853245	transporter activity
Δwc2	12-day	2641735	up	GO:0005215	0,000853245	transporter activity
Δwc2	12-day	2642894	up	GO:0005215	0,000853245	transporter activity
Δwc2	12-day	2660764	up	GO:0005215	0,000853245	transporter activity
Δwc2	12-day	2663452	up	GO:0005215	0,000853245	transporter activity
Δwc2	12-day	2680674	up	GO:0005215	0,000853245	transporter activity

Δwc2	12-day	2698170	up	GO:0005215	0,000853245	transporter activity
Δwc2	12-day	2704067	up	GO:0005215	0,000853245	transporter activity
Δwc2	12-day	83108	up	GO:0005524	0,006553369	ATP binding
Δwc2	12-day	258402	up	GO:0005524	0,006553369	ATP binding
Δwc2	12-day	1129062	up	GO:0005524	0,006553369	ATP binding
Δwc2	12-day	1157948	up	GO:0005524	0,006553369	ATP binding
Δwc2	12-day	1158800	up	GO:0005524	0,006553369	ATP binding
Δwc2	12-day	1172108	up	GO:0005524	0,006553369	ATP binding
Δwc2	12-day	1174869	up	GO:0005524	0,006553369	ATP binding
Δwc2	12-day	1178759	up	GO:0005524	0,006553369	ATP binding
Δwc2	12-day	1181112	up	GO:0005524	0,006553369	ATP binding
Δwc2	12-day	1186310	up	GO:0005524	0,006553369	ATP binding
Δwc2	12-day	1191914	up	GO:0005524	0,006553369	ATP binding
Δwc2	12-day	2253810	up	GO:0005524	0,006553369	ATP binding
Δwc2	12-day	2481145	up	GO:0005524	0,006553369	ATP binding
Δwc2	12-day	2484841	up	GO:0005524	0,006553369	ATP binding
Δwc2	12-day	2485795	up	GO:0005524	0,006553369	ATP binding
Δwc2	12-day	2486797	up	GO:0005524	0,006553369	ATP binding
Δwc2	12-day	2490024	up	GO:0005524	0,006553369	ATP binding
Δwc2	12-day	2490823	up	GO:0005524	0,006553369	ATP binding
Δwc2	12-day	2493556	up	GO:0005524	0,006553369	ATP binding
Δwc2	12-day	2495301	up	GO:0005524	0,006553369	ATP binding
Δwc2	12-day	2503832	up	GO:0005524	0,006553369	ATP binding
Δwc2	12-day	2510275	up	GO:0005524	0,006553369	ATP binding
Δwc2	12-day	2511561	up	GO:0005524	0,006553369	ATP binding
Δwc2	12-day	2514780	up	GO:0005524	0,006553369	ATP binding
Δwc2	12-day	2520682	up	GO:0005524	0,006553369	ATP binding
Δwc2	12-day	2527752	up	GO:0005524	0,006553369	ATP binding
Δwc2	12-day	2530387	up	GO:0005524	0,006553369	ATP binding
Δwc2	12-day	2533371	up	GO:0005524	0,006553369	ATP binding
Δwc2	12-day	2535079	up	GO:0005524	0,006553369	ATP binding
Δwc2	12-day	2535527	up	GO:0005524	0,006553369	ATP binding
Δwc2	12-day	2536562	up	GO:0005524	0,006553369	ATP binding
Δwc2	12-day	2540501	up	GO:0005524	0,006553369	ATP binding

Δwc2	12-day	2550266	up	GO:0005524	0,006553369	ATP binding
Δwc2	12-day	2550939	up	GO:0005524	0,006553369	ATP binding
Δwc2	12-day	2553325	up	GO:0005524	0,006553369	ATP binding
Δwc2	12-day	2554917	up	GO:0005524	0,006553369	ATP binding
Δwc2	12-day	2563653	up	GO:0005524	0,006553369	ATP binding
Δwc2	12-day	2565471	up	GO:0005524	0,006553369	ATP binding
Δwc2	12-day	2568673	up	GO:0005524	0,006553369	ATP binding
Δwc2	12-day	2569377	up	GO:0005524	0,006553369	ATP binding
Δwc2	12-day	2583917	up	GO:0005524	0,006553369	ATP binding
Δwc2	12-day	2585476	up	GO:0005524	0,006553369	ATP binding
Δwc2	12-day	2585708	up	GO:0005524	0,006553369	ATP binding
Δwc2	12-day	2601543	up	GO:0005524	0,006553369	ATP binding
Δwc2	12-day	2605230	up	GO:0005524	0,006553369	ATP binding
Δwc2	12-day	2605455	up	GO:0005524	0,006553369	ATP binding
Δwc2	12-day	2606202	up	GO:0005524	0,006553369	ATP binding
Δwc2	12-day	2610180	up	GO:0005524	0,006553369	ATP binding
Δwc2	12-day	2610629	up	GO:0005524	0,006553369	ATP binding
Δwc2	12-day	2611113	up	GO:0005524	0,006553369	ATP binding
Δwc2	12-day	2611700	up	GO:0005524	0,006553369	ATP binding
Δwc2	12-day	2612206	up	GO:0005524	0,006553369	ATP binding
Δwc2	12-day	2612608	up	GO:0005524	0,006553369	ATP binding
Δwc2	12-day	2612626	up	GO:0005524	0,006553369	ATP binding
Δwc2	12-day	2614761	up	GO:0005524	0,006553369	ATP binding
Δwc2	12-day	2614856	up	GO:0005524	0,006553369	ATP binding
Δwc2	12-day	2616339	up	GO:0005524	0,006553369	ATP binding
Δwc2	12-day	2620969	up	GO:0005524	0,006553369	ATP binding
Δwc2	12-day	2621533	up	GO:0005524	0,006553369	ATP binding
Δwc2	12-day	2621741	up	GO:0005524	0,006553369	ATP binding
Δwc2	12-day	2623214	up	GO:0005524	0,006553369	ATP binding
Δwc2	12-day	2623257	up	GO:0005524	0,006553369	ATP binding
Δwc2	12-day	2623685	up	GO:0005524	0,006553369	ATP binding
Δwc2	12-day	2626895	up	GO:0005524	0,006553369	ATP binding
Δwc2	12-day	2631390	up	GO:0005524	0,006553369	ATP binding
Δwc2	12-day	2632313	up	GO:0005524	0,006553369	ATP binding

Δwc2	12-day	2632374	up	GO:0005524	0,006553369	ATP binding
Δwc2	12-day	2633881	up	GO:0005524	0,006553369	ATP binding
Δwc2	12-day	2636368	up	GO:0005524	0,006553369	ATP binding
Δwc2	12-day	2636577	up	GO:0005524	0,006553369	ATP binding
Δwc2	12-day	2638986	up	GO:0005524	0,006553369	ATP binding
Δwc2	12-day	2641678	up	GO:0005524	0,006553369	ATP binding
Δwc2	12-day	2645169	up	GO:0005524	0,006553369	ATP binding
Δwc2	12-day	2645675	up	GO:0005524	0,006553369	ATP binding
Δwc2	12-day	2661304	up	GO:0005524	0,006553369	ATP binding
Δwc2	12-day	2671089	up	GO:0005524	0,006553369	ATP binding
Δwc2	12-day	2672807	up	GO:0005524	0,006553369	ATP binding
Δwc2	12-day	2675859	up	GO:0005524	0,006553369	ATP binding
Δwc2	12-day	2682841	up	GO:0005524	0,006553369	ATP binding
Δwc2	12-day	2684163	up	GO:0005524	0,006553369	ATP binding
Δwc2	12-day	2689581	up	GO:0005524	0,006553369	ATP binding
Δwc2	12-day	2693043	up	GO:0005524	0,006553369	ATP binding
Δwc2	12-day	2693940	up	GO:0005524	0,006553369	ATP binding
Δwc2	12-day	2702947	up	GO:0005524	0,006553369	ATP binding
Δwc2	12-day	2725125	up	GO:0005524	0,006553369	ATP binding
Δwc2	12-day	2753335	up	GO:0005524	0,006553369	ATP binding
Δwc2	12-day	2606344	down	GO:0000786	7,17E-08	nucleosome
Δwc2	12-day	2630193	down	GO:0000786	7,17E-08	nucleosome
Δwc2	12-day	2606344	down	GO:0006334	2,72E-07	nucleosome assembly
Δwc2	12-day	2630193	down	GO:0006334	2,72E-07	nucleosome assembly
Δwc2	12-day	1189569	down	GO:0003677	1,33E-06	DNA binding
Δwc2	12-day	1192535	down	GO:0003677	1,33E-06	DNA binding
Δwc2	12-day	2045557	down	GO:0003677	1,33E-06	DNA binding
Δwc2	12-day	2483746	down	GO:0003677	1,33E-06	DNA binding
Δwc2	12-day	2503711	down	GO:0003677	1,33E-06	DNA binding
Δwc2	12-day	2511242	down	GO:0003677	1,33E-06	DNA binding
Δwc2	12-day	2517503	down	GO:0003677	1,33E-06	DNA binding
Δwc2	12-day	2596857	down	GO:0003677	1,33E-06	DNA binding
Δwc2	12-day	2605931	down	GO:0003677	1,33E-06	DNA binding
Δwc2	12-day	2606344	down	GO:0003677	1,33E-06	DNA binding

Δwc2	12-day	2607126	down	GO:0003677	1,33E-06	DNA binding
Δwc2	12-day	2609231	down	GO:0003677	1,33E-06	DNA binding
Δwc2	12-day	2613007	down	GO:0003677	1,33E-06	DNA binding
Δwc2	12-day	2613388	down	GO:0003677	1,33E-06	DNA binding
Δwc2	12-day	2618564	down	GO:0003677	1,33E-06	DNA binding
Δwc2	12-day	2620175	down	GO:0003677	1,33E-06	DNA binding
Δwc2	12-day	2622267	down	GO:0003677	1,33E-06	DNA binding
Δwc2	12-day	2625706	down	GO:0003677	1,33E-06	DNA binding
Δwc2	12-day	2628620	down	GO:0003677	1,33E-06	DNA binding
Δwc2	12-day	2629597	down	GO:0003677	1,33E-06	DNA binding
Δwc2	12-day	2630193	down	GO:0003677	1,33E-06	DNA binding
Δwc2	12-day	2634413	down	GO:0003677	1,33E-06	DNA binding
Δwc2	12-day	2666726	down	GO:0003677	1,33E-06	DNA binding
Δwc2	12-day	2687845	down	GO:0003677	1,33E-06	DNA binding
Δwc2	12-day	1152293	down	GO:0008152	6,77E-06	metabolic process
Δwc2	12-day	2135332	down	GO:0008152	6,77E-06	metabolic process
Δwc2	12-day	2194185	down	GO:0008152	6,77E-06	metabolic process
Δwc2	12-day	2255031	down	GO:0008152	6,77E-06	metabolic process
Δwc2	12-day	2333133	down	GO:0008152	6,77E-06	metabolic process
Δwc2	12-day	2492878	down	GO:0008152	6,77E-06	metabolic process
Δwc2	12-day	2495032	down	GO:0008152	6,77E-06	metabolic process
Δwc2	12-day	2507164	down	GO:0008152	6,77E-06	metabolic process
Δwc2	12-day	2509783	down	GO:0008152	6,77E-06	metabolic process
Δwc2	12-day	2511020	down	GO:0008152	6,77E-06	metabolic process
Δwc2	12-day	2515850	down	GO:0008152	6,77E-06	metabolic process
Δwc2	12-day	2553610	down	GO:0008152	6,77E-06	metabolic process
Δwc2	12-day	2577440	down	GO:0008152	6,77E-06	metabolic process
Δwc2	12-day	2587289	down	GO:0008152	6,77E-06	metabolic process
Δwc2	12-day	2604917	down	GO:0008152	6,77E-06	metabolic process
Δwc2	12-day	2606988	down	GO:0008152	6,77E-06	metabolic process
Δwc2	12-day	2607837	down	GO:0008152	6,77E-06	metabolic process
Δwc2	12-day	2608099	down	GO:0008152	6,77E-06	metabolic process
Δwc2	12-day	2609150	down	GO:0008152	6,77E-06	metabolic process
Δwc2	12-day	2610372	down	GO:0008152	6,77E-06	metabolic process

Δwc2	12-day	2610587	down	GO:0008152	6,77E-06	metabolic process
Δwc2	12-day	2611306	down	GO:0008152	6,77E-06	metabolic process
Δwc2	12-day	2611868	down	GO:0008152	6,77E-06	metabolic process
Δwc2	12-day	2615598	down	GO:0008152	6,77E-06	metabolic process
Δwc2	12-day	2616602	down	GO:0008152	6,77E-06	metabolic process
Δwc2	12-day	2616880	down	GO:0008152	6,77E-06	metabolic process
Δwc2	12-day	2616916	down	GO:0008152	6,77E-06	metabolic process
Δwc2	12-day	2618904	down	GO:0008152	6,77E-06	metabolic process
Δwc2	12-day	2619714	down	GO:0008152	6,77E-06	metabolic process
Δwc2	12-day	2619840	down	GO:0008152	6,77E-06	metabolic process
Δwc2	12-day	2621873	down	GO:0008152	6,77E-06	metabolic process
Δwc2	12-day	2622287	down	GO:0008152	6,77E-06	metabolic process
Δwc2	12-day	2623068	down	GO:0008152	6,77E-06	metabolic process
Δwc2	12-day	2623301	down	GO:0008152	6,77E-06	metabolic process
Δwc2	12-day	2623956	down	GO:0008152	6,77E-06	metabolic process
Δwc2	12-day	2624525	down	GO:0008152	6,77E-06	metabolic process
Δwc2	12-day	2624675	down	GO:0008152	6,77E-06	metabolic process
Δwc2	12-day	2625667	down	GO:0008152	6,77E-06	metabolic process
Δwc2	12-day	2625706	down	GO:0008152	6,77E-06	metabolic process
Δwc2	12-day	2625782	down	GO:0008152	6,77E-06	metabolic process
Δwc2	12-day	2626510	down	GO:0008152	6,77E-06	metabolic process
Δwc2	12-day	2627304	down	GO:0008152	6,77E-06	metabolic process
Δwc2	12-day	2629548	down	GO:0008152	6,77E-06	metabolic process
Δwc2	12-day	2631237	down	GO:0008152	6,77E-06	metabolic process
Δwc2	12-day	2631774	down	GO:0008152	6,77E-06	metabolic process
Δwc2	12-day	2632206	down	GO:0008152	6,77E-06	metabolic process
Δwc2	12-day	2632611	down	GO:0008152	6,77E-06	metabolic process
Δwc2	12-day	2633167	down	GO:0008152	6,77E-06	metabolic process
Δwc2	12-day	2633632	down	GO:0008152	6,77E-06	metabolic process
Δwc2	12-day	2636100	down	GO:0008152	6,77E-06	metabolic process
Δwc2	12-day	2637708	down	GO:0008152	6,77E-06	metabolic process
Δwc2	12-day	2637755	down	GO:0008152	6,77E-06	metabolic process
Δwc2	12-day	2638484	down	GO:0008152	6,77E-06	metabolic process
Δwc2	12-day	2640472	down	GO:0008152	6,77E-06	metabolic process

Δwc2	12-day	2640484	down	GO:0008152	6,77E-06	metabolic process
Δwc2	12-day	2640992	down	GO:0008152	6,77E-06	metabolic process
Δwc2	12-day	2641506	down	GO:0008152	6,77E-06	metabolic process
Δwc2	12-day	2641520	down	GO:0008152	6,77E-06	metabolic process
Δwc2	12-day	2643650	down	GO:0008152	6,77E-06	metabolic process
Δwc2	12-day	2644127	down	GO:0008152	6,77E-06	metabolic process
Δwc2	12-day	2644281	down	GO:0008152	6,77E-06	metabolic process
Δwc2	12-day	2644330	down	GO:0008152	6,77E-06	metabolic process
Δwc2	12-day	2644430	down	GO:0008152	6,77E-06	metabolic process
Δwc2	12-day	2645207	down	GO:0008152	6,77E-06	metabolic process
Δwc2	12-day	2645668	down	GO:0008152	6,77E-06	metabolic process
Δwc2	12-day	2660684	down	GO:0008152	6,77E-06	metabolic process
Δwc2	12-day	2665516	down	GO:0008152	6,77E-06	metabolic process
Δwc2	12-day	2673857	down	GO:0008152	6,77E-06	metabolic process
Δwc2	12-day	2696261	down	GO:0008152	6,77E-06	metabolic process
Δwc2	12-day	2703509	down	GO:0008152	6,77E-06	metabolic process
Δwc2	12-day	2705421	down	GO:0008152	6,77E-06	metabolic process
Δwc2	12-day	2711060	down	GO:0008152	6,77E-06	metabolic process
Δwc2	12-day	2726410	down	GO:0008152	6,77E-06	metabolic process
Δwc2	12-day	2728826	down	GO:0008152	6,77E-06	metabolic process
Δwc2	12-day	85278	down	GO:0003824	6,77E-06	catalytic activity
Δwc2	12-day	237513	down	GO:0003824	6,77E-06	catalytic activity
Δwc2	12-day	1152293	down	GO:0003824	6,77E-06	catalytic activity
Δwc2	12-day	1184723	down	GO:0003824	6,77E-06	catalytic activity
Δwc2	12-day	1185226	down	GO:0003824	6,77E-06	catalytic activity
Δwc2	12-day	1191999	down	GO:0003824	6,77E-06	catalytic activity
Δwc2	12-day	2135332	down	GO:0003824	6,77E-06	catalytic activity
Δwc2	12-day	2194185	down	GO:0003824	6,77E-06	catalytic activity
Δwc2	12-day	2255031	down	GO:0003824	6,77E-06	catalytic activity
Δwc2	12-day	2333133	down	GO:0003824	6,77E-06	catalytic activity
Δwc2	12-day	2368049	down	GO:0003824	6,77E-06	catalytic activity
Δwc2	12-day	2492878	down	GO:0003824	6,77E-06	catalytic activity
Δwc2	12-day	2495032	down	GO:0003824	6,77E-06	catalytic activity
Δwc2	12-day	2509783	down	GO:0003824	6,77E-06	catalytic activity

Δwc2	12-day	2511020	down	GO:0003824	6,77E-06	catalytic activity
Δwc2	12-day	2512873	down	GO:0003824	6,77E-06	catalytic activity
Δwc2	12-day	2513424	down	GO:0003824	6,77E-06	catalytic activity
Δwc2	12-day	2516444	down	GO:0003824	6,77E-06	catalytic activity
Δwc2	12-day	2587289	down	GO:0003824	6,77E-06	catalytic activity
Δwc2	12-day	2598960	down	GO:0003824	6,77E-06	catalytic activity
Δwc2	12-day	2605122	down	GO:0003824	6,77E-06	catalytic activity
Δwc2	12-day	2606988	down	GO:0003824	6,77E-06	catalytic activity
Δwc2	12-day	2607034	down	GO:0003824	6,77E-06	catalytic activity
Δwc2	12-day	2607837	down	GO:0003824	6,77E-06	catalytic activity
Δwc2	12-day	2610372	down	GO:0003824	6,77E-06	catalytic activity
Δwc2	12-day	2610587	down	GO:0003824	6,77E-06	catalytic activity
Δwc2	12-day	2615598	down	GO:0003824	6,77E-06	catalytic activity
Δwc2	12-day	2616491	down	GO:0003824	6,77E-06	catalytic activity
Δwc2	12-day	2616602	down	GO:0003824	6,77E-06	catalytic activity
Δwc2	12-day	2616880	down	GO:0003824	6,77E-06	catalytic activity
Δwc2	12-day	2616916	down	GO:0003824	6,77E-06	catalytic activity
Δwc2	12-day	2617562	down	GO:0003824	6,77E-06	catalytic activity
Δwc2	12-day	2618252	down	GO:0003824	6,77E-06	catalytic activity
Δwc2	12-day	2618425	down	GO:0003824	6,77E-06	catalytic activity
Δwc2	12-day	2618479	down	GO:0003824	6,77E-06	catalytic activity
Δwc2	12-day	2618904	down	GO:0003824	6,77E-06	catalytic activity
Δwc2	12-day	2618997	down	GO:0003824	6,77E-06	catalytic activity
Δwc2	12-day	2619714	down	GO:0003824	6,77E-06	catalytic activity
Δwc2	12-day	2621873	down	GO:0003824	6,77E-06	catalytic activity
Δwc2	12-day	2622287	down	GO:0003824	6,77E-06	catalytic activity
Δwc2	12-day	2623068	down	GO:0003824	6,77E-06	catalytic activity
Δwc2	12-day	2623301	down	GO:0003824	6,77E-06	catalytic activity
Δwc2	12-day	2624675	down	GO:0003824	6,77E-06	catalytic activity
Δwc2	12-day	2625667	down	GO:0003824	6,77E-06	catalytic activity
Δwc2	12-day	2625782	down	GO:0003824	6,77E-06	catalytic activity
Δwc2	12-day	2625818	down	GO:0003824	6,77E-06	catalytic activity
Δwc2	12-day	2626510	down	GO:0003824	6,77E-06	catalytic activity
Δwc2	12-day	2627304	down	GO:0003824	6,77E-06	catalytic activity

Δwc2	12-day	2628678	down	GO:0003824	6,77E-06	catalytic activity
Δwc2	12-day	2629548	down	GO:0003824	6,77E-06	catalytic activity
Δwc2	12-day	2630634	down	GO:0003824	6,77E-06	catalytic activity
Δwc2	12-day	2631237	down	GO:0003824	6,77E-06	catalytic activity
Δwc2	12-day	2631394	down	GO:0003824	6,77E-06	catalytic activity
Δwc2	12-day	2632206	down	GO:0003824	6,77E-06	catalytic activity
Δwc2	12-day	2632611	down	GO:0003824	6,77E-06	catalytic activity
Δwc2	12-day	2633144	down	GO:0003824	6,77E-06	catalytic activity
Δwc2	12-day	2633162	down	GO:0003824	6,77E-06	catalytic activity
Δwc2	12-day	2633167	down	GO:0003824	6,77E-06	catalytic activity
Δwc2	12-day	2633632	down	GO:0003824	6,77E-06	catalytic activity
Δwc2	12-day	2634918	down	GO:0003824	6,77E-06	catalytic activity
Δwc2	12-day	2635216	down	GO:0003824	6,77E-06	catalytic activity
Δwc2	12-day	2635219	down	GO:0003824	6,77E-06	catalytic activity
Δwc2	12-day	2638484	down	GO:0003824	6,77E-06	catalytic activity
Δwc2	12-day	2638783	down	GO:0003824	6,77E-06	catalytic activity
Δwc2	12-day	2640484	down	GO:0003824	6,77E-06	catalytic activity
Δwc2	12-day	2640992	down	GO:0003824	6,77E-06	catalytic activity
Δwc2	12-day	2641022	down	GO:0003824	6,77E-06	catalytic activity
Δwc2	12-day	2641506	down	GO:0003824	6,77E-06	catalytic activity
Δwc2	12-day	2641520	down	GO:0003824	6,77E-06	catalytic activity
Δwc2	12-day	2643650	down	GO:0003824	6,77E-06	catalytic activity
Δwc2	12-day	2643902	down	GO:0003824	6,77E-06	catalytic activity
Δwc2	12-day	2644281	down	GO:0003824	6,77E-06	catalytic activity
Δwc2	12-day	2644330	down	GO:0003824	6,77E-06	catalytic activity
Δwc2	12-day	2645207	down	GO:0003824	6,77E-06	catalytic activity
Δwc2	12-day	2645668	down	GO:0003824	6,77E-06	catalytic activity
Δwc2	12-day	2660684	down	GO:0003824	6,77E-06	catalytic activity
Δwc2	12-day	2673857	down	GO:0003824	6,77E-06	catalytic activity
Δwc2	12-day	2688172	down	GO:0003824	6,77E-06	catalytic activity
Δwc2	12-day	2696261	down	GO:0003824	6,77E-06	catalytic activity
Δwc2	12-day	2699217	down	GO:0003824	6,77E-06	catalytic activity
Δwc2	12-day	2703509	down	GO:0003824	6,77E-06	catalytic activity
Δwc2	12-day	2705421	down	GO:0003824	6,77E-06	catalytic activity

Δwc2	12-day	2711060	down	GO:0003824	6,77E-06	catalytic activity
Δwc2	12-day	2725020	down	GO:0003824	6,77E-06	catalytic activity
Δwc2	12-day	2493870	down	GO:0008094	3,64E-05	DNA-dependent ATPase activity
Δwc2	12-day	2614908	down	GO:0008094	3,64E-05	DNA-dependent ATPase activity
Δwc2	12-day	2493870	down	GO:0015462	3,64E-05	protein-transmembrane transporting ATPase activity
Δwc2	12-day	2614908	down	GO:0015462	3,64E-05	protein-transmembrane transporting ATPase activity
Δwc2	12-day	2493870	down	GO:0004004	3,64E-05	ATP-dependent RNA helicase activity
Δwc2	12-day	2614908	down	GO:0004004	3,64E-05	ATP-dependent RNA helicase activity
Δwc2	12-day	2493870	down	GO:0004003	3,64E-05	ATP-dependent DNA helicase activity
Δwc2	12-day	2614908	down	GO:0004003	3,64E-05	ATP-dependent DNA helicase activity
Δwc2	12-day	2493870	down	GO:0017116	3,64E-05	single-stranded DNA-dependent ATP-dependent DNA helicase activity
Δwc2	12-day	2614908	down	GO:0017116	3,64E-05	single-stranded DNA-dependent ATP-dependent DNA helicase activity
Δwc2	12-day	2493870	down	GO:0042623	3,64E-05	ATPase activity, coupled
Δwc2	12-day	2614908	down	GO:0042623	3,64E-05	ATPase activity, coupled
Δwc2	12-day	2493870	down	GO:0042625	3,64E-05	ATPase activity, coupled to transmembrane movement of ions
Δwc2	12-day	2614908	down	GO:0042625	3,64E-05	ATPase activity, coupled to transmembrane movement of ions
Δwc2	12-day	2493870	down	GO:0042624	3,64E-05	ATPase activity, uncoupled
Δwc2	12-day	2614908	down	GO:0042624	3,64E-05	ATPase activity, uncoupled
Δwc2	12-day	237513	down	GO:0016491	3,64E-05	oxidoreductase activity
Δwc2	12-day	1038608	down	GO:0016491	3,64E-05	oxidoreductase activity
Δwc2	12-day	1147332	down	GO:0016491	3,64E-05	oxidoreductase activity
Δwc2	12-day	1152293	down	GO:0016491	3,64E-05	oxidoreductase activity
Δwc2	12-day	2255031	down	GO:0016491	3,64E-05	oxidoreductase activity
Δwc2	12-day	2333133	down	GO:0016491	3,64E-05	oxidoreductase activity
Δwc2	12-day	2376858	down	GO:0016491	3,64E-05	oxidoreductase activity
Δwc2	12-day	2483752	down	GO:0016491	3,64E-05	oxidoreductase activity
Δwc2	12-day	2490518	down	GO:0016491	3,64E-05	oxidoreductase activity
Δwc2	12-day	2497674	down	GO:0016491	3,64E-05	oxidoreductase activity
Δwc2	12-day	2502495	down	GO:0016491	3,64E-05	oxidoreductase activity
Δwc2	12-day	2503108	down	GO:0016491	3,64E-05	oxidoreductase activity
Δwc2	12-day	2507164	down	GO:0016491	3,64E-05	oxidoreductase activity
Δwc2	12-day	2515850	down	GO:0016491	3,64E-05	oxidoreductase activity

Δwc2	12-day	2516444	down	GO:0016491	3,64E-05	oxidoreductase activity
Δwc2	12-day	2532967	down	GO:0016491	3,64E-05	oxidoreductase activity
Δwc2	12-day	2543454	down	GO:0016491	3,64E-05	oxidoreductase activity
Δwc2	12-day	2571870	down	GO:0016491	3,64E-05	oxidoreductase activity
Δwc2	12-day	2587289	down	GO:0016491	3,64E-05	oxidoreductase activity
Δwc2	12-day	2606988	down	GO:0016491	3,64E-05	oxidoreductase activity
Δwc2	12-day	2607165	down	GO:0016491	3,64E-05	oxidoreductase activity
Δwc2	12-day	2609840	down	GO:0016491	3,64E-05	oxidoreductase activity
Δwc2	12-day	2610372	down	GO:0016491	3,64E-05	oxidoreductase activity
Δwc2	12-day	2610587	down	GO:0016491	3,64E-05	oxidoreductase activity
Δwc2	12-day	2611538	down	GO:0016491	3,64E-05	oxidoreductase activity
Δwc2	12-day	2611868	down	GO:0016491	3,64E-05	oxidoreductase activity
Δwc2	12-day	2615160	down	GO:0016491	3,64E-05	oxidoreductase activity
Δwc2	12-day	2616880	down	GO:0016491	3,64E-05	oxidoreductase activity
Δwc2	12-day	2616916	down	GO:0016491	3,64E-05	oxidoreductase activity
Δwc2	12-day	2619840	down	GO:0016491	3,64E-05	oxidoreductase activity
Δwc2	12-day	2621035	down	GO:0016491	3,64E-05	oxidoreductase activity
Δwc2	12-day	2621873	down	GO:0016491	3,64E-05	oxidoreductase activity
Δwc2	12-day	2623301	down	GO:0016491	3,64E-05	oxidoreductase activity
Δwc2	12-day	2625667	down	GO:0016491	3,64E-05	oxidoreductase activity
Δwc2	12-day	2627304	down	GO:0016491	3,64E-05	oxidoreductase activity
Δwc2	12-day	2630637	down	GO:0016491	3,64E-05	oxidoreductase activity
Δwc2	12-day	2631774	down	GO:0016491	3,64E-05	oxidoreductase activity
Δwc2	12-day	2632136	down	GO:0016491	3,64E-05	oxidoreductase activity
Δwc2	12-day	2633167	down	GO:0016491	3,64E-05	oxidoreductase activity
Δwc2	12-day	2633632	down	GO:0016491	3,64E-05	oxidoreductase activity
Δwc2	12-day	2637755	down	GO:0016491	3,64E-05	oxidoreductase activity
Δwc2	12-day	2638484	down	GO:0016491	3,64E-05	oxidoreductase activity
Δwc2	12-day	2639682	down	GO:0016491	3,64E-05	oxidoreductase activity
Δwc2	12-day	2641368	down	GO:0016491	3,64E-05	oxidoreductase activity
Δwc2	12-day	2641506	down	GO:0016491	3,64E-05	oxidoreductase activity
Δwc2	12-day	2641520	down	GO:0016491	3,64E-05	oxidoreductase activity
Δwc2	12-day	2644127	down	GO:0016491	3,64E-05	oxidoreductase activity
Δwc2	12-day	2644330	down	GO:0016491	3,64E-05	oxidoreductase activity

Δwc2	12-day	2644430	down	GO:0016491	3,64E-05	oxidoreductase activity
Δwc2	12-day	2661224	down	GO:0016491	3,64E-05	oxidoreductase activity
Δwc2	12-day	2663372	down	GO:0016491	3,64E-05	oxidoreductase activity
Δwc2	12-day	2688172	down	GO:0016491	3,64E-05	oxidoreductase activity
Δwc2	12-day	2697968	down	GO:0016491	3,64E-05	oxidoreductase activity
Δwc2	12-day	2705421	down	GO:0016491	3,64E-05	oxidoreductase activity
Δwc2	12-day	2482745	down	GO:0016887	3,64E-05	ATPase activity
Δwc2	12-day	2493870	down	GO:0016887	3,64E-05	ATPase activity
Δwc2	12-day	2614908	down	GO:0016887	3,64E-05	ATPase activity
Δwc2	12-day	2637190	down	GO:0016887	3,64E-05	ATPase activity
Δwc2	12-day	2493870	down	GO:0008186	3,64E-05	RNA-dependent ATPase activity
Δwc2	12-day	2614908	down	GO:0008186	3,64E-05	RNA-dependent ATPase activity
Δwc2	12-day	2512973	down	GO:0034061	9,57E-05	DNA polymerase activity
Δwc2	12-day	2493870	down	GO:0042626	0,000109734	ATPase activity, coupled to transmembrane movement of substances
Δwc2	12-day	2614908	down	GO:0042626	0,000109734	ATPase activity, coupled to transmembrane movement of substances
Δwc2	12-day	2637190	down	GO:0042626	0,000109734	ATPase activity, coupled to transmembrane movement of substances
Δwc2	12-day	2512973	down	GO:0003887	0,000169381	DNA-directed DNA polymerase activity
Δwc2	12-day	1165803	down	GO:0008026	0,000321384	ATP-dependent helicase activity
Δwc2	12-day	2482187	down	GO:0008026	0,000321384	ATP-dependent helicase activity
Δwc2	12-day	2493870	down	GO:0008026	0,000321384	ATP-dependent helicase activity
Δwc2	12-day	2614908	down	GO:0008026	0,000321384	ATP-dependent helicase activity
Δwc2	12-day	2695418	down	GO:0008026	0,000321384	ATP-dependent helicase activity
Δwc2	12-day	1144263	down	GO:0006810	0,00037026	transport
Δwc2	12-day	1153136	down	GO:0006810	0,00037026	transport
Δwc2	12-day	1154281	down	GO:0006810	0,00037026	transport
Δwc2	12-day	1267603	down	GO:0006810	0,00037026	transport
Δwc2	12-day	1357952	down	GO:0006810	0,00037026	transport
Δwc2	12-day	2122325	down	GO:0006810	0,00037026	transport
Δwc2	12-day	2138287	down	GO:0006810	0,00037026	transport
Δwc2	12-day	2162840	down	GO:0006810	0,00037026	transport
Δwc2	12-day	2481653	down	GO:0006810	0,00037026	transport

Δwc2	12-day	2488542	down	GO:0006810	0,00037026	transport
Δwc2	12-day	2492878	down	GO:0006810	0,00037026	transport
Δwc2	12-day	2494144	down	GO:0006810	0,00037026	transport
Δwc2	12-day	2494727	down	GO:0006810	0,00037026	transport
Δwc2	12-day	2514012	down	GO:0006810	0,00037026	transport
Δwc2	12-day	2520877	down	GO:0006810	0,00037026	transport
Δwc2	12-day	2525305	down	GO:0006810	0,00037026	transport
Δwc2	12-day	2576922	down	GO:0006810	0,00037026	transport
Δwc2	12-day	2600551	down	GO:0006810	0,00037026	transport
Δwc2	12-day	2607025	down	GO:0006810	0,00037026	transport
Δwc2	12-day	2607891	down	GO:0006810	0,00037026	transport
Δwc2	12-day	2608678	down	GO:0006810	0,00037026	transport
Δwc2	12-day	2609987	down	GO:0006810	0,00037026	transport
Δwc2	12-day	2611816	down	GO:0006810	0,00037026	transport
Δwc2	12-day	2612806	down	GO:0006810	0,00037026	transport
Δwc2	12-day	2613255	down	GO:0006810	0,00037026	transport
Δwc2	12-day	2616602	down	GO:0006810	0,00037026	transport
Δwc2	12-day	2619645	down	GO:0006810	0,00037026	transport
Δwc2	12-day	2620178	down	GO:0006810	0,00037026	transport
Δwc2	12-day	2620830	down	GO:0006810	0,00037026	transport
Δwc2	12-day	2621828	down	GO:0006810	0,00037026	transport
Δwc2	12-day	2622598	down	GO:0006810	0,00037026	transport
Δwc2	12-day	2623420	down	GO:0006810	0,00037026	transport
Δwc2	12-day	2623492	down	GO:0006810	0,00037026	transport
Δwc2	12-day	2626803	down	GO:0006810	0,00037026	transport
Δwc2	12-day	2627703	down	GO:0006810	0,00037026	transport
Δwc2	12-day	2628751	down	GO:0006810	0,00037026	transport
Δwc2	12-day	2629091	down	GO:0006810	0,00037026	transport
Δwc2	12-day	2629455	down	GO:0006810	0,00037026	transport
Δwc2	12-day	2636597	down	GO:0006810	0,00037026	transport
Δwc2	12-day	2637190	down	GO:0006810	0,00037026	transport
Δwc2	12-day	2637509	down	GO:0006810	0,00037026	transport
Δwc2	12-day	2642018	down	GO:0006810	0,00037026	transport
Δwc2	12-day	2645850	down	GO:0006810	0,00037026	transport

Δwc2	12-day	2661224	down	GO:0006810	0,00037026	transport
Δwc2	12-day	2662585	down	GO:0006810	0,00037026	transport
Δwc2	12-day	2701571	down	GO:0006810	0,00037026	transport
Δwc2	12-day	2712060	down	GO:0006810	0,00037026	transport
Δwc2	12-day	2738153	down	GO:0006810	0,00037026	transport
Δwc2	12-day	78628	down	GO:0006118	0,004112859	electron transport
Δwc2	12-day	81631	down	GO:0006118	0,004112859	electron transport
Δwc2	12-day	1038608	down	GO:0006118	0,004112859	electron transport
Δwc2	12-day	1084300	down	GO:0006118	0,004112859	electron transport
Δwc2	12-day	1131542	down	GO:0006118	0,004112859	electron transport
Δwc2	12-day	1189794	down	GO:0006118	0,004112859	electron transport
Δwc2	12-day	1193647	down	GO:0006118	0,004112859	electron transport
Δwc2	12-day	2327903	down	GO:0006118	0,004112859	electron transport
Δwc2	12-day	2450442	down	GO:0006118	0,004112859	electron transport
Δwc2	12-day	2484868	down	GO:0006118	0,004112859	electron transport
Δwc2	12-day	2486680	down	GO:0006118	0,004112859	electron transport
Δwc2	12-day	2490518	down	GO:0006118	0,004112859	electron transport
Δwc2	12-day	2497674	down	GO:0006118	0,004112859	electron transport
Δwc2	12-day	2503108	down	GO:0006118	0,004112859	electron transport
Δwc2	12-day	2507891	down	GO:0006118	0,004112859	electron transport
Δwc2	12-day	2510129	down	GO:0006118	0,004112859	electron transport
Δwc2	12-day	2510618	down	GO:0006118	0,004112859	electron transport
Δwc2	12-day	2512276	down	GO:0006118	0,004112859	electron transport
Δwc2	12-day	2515850	down	GO:0006118	0,004112859	electron transport
Δwc2	12-day	2532967	down	GO:0006118	0,004112859	electron transport
Δwc2	12-day	2571870	down	GO:0006118	0,004112859	electron transport
Δwc2	12-day	2583618	down	GO:0006118	0,004112859	electron transport
Δwc2	12-day	2604096	down	GO:0006118	0,004112859	electron transport
Δwc2	12-day	2604231	down	GO:0006118	0,004112859	electron transport
Δwc2	12-day	2604458	down	GO:0006118	0,004112859	electron transport
Δwc2	12-day	2604628	down	GO:0006118	0,004112859	electron transport
Δwc2	12-day	2604735	down	GO:0006118	0,004112859	electron transport
Δwc2	12-day	2609840	down	GO:0006118	0,004112859	electron transport
Δwc2	12-day	2611234	down	GO:0006118	0,004112859	electron transport

Δwc2	12-day	2614998	down	GO:0006118	0,004112859	electron transport
Δwc2	12-day	2616301	down	GO:0006118	0,004112859	electron transport
Δwc2	12-day	2619674	down	GO:0006118	0,004112859	electron transport
Δwc2	12-day	2619840	down	GO:0006118	0,004112859	electron transport
Δwc2	12-day	2621855	down	GO:0006118	0,004112859	electron transport
Δwc2	12-day	2623115	down	GO:0006118	0,004112859	electron transport
Δwc2	12-day	2623122	down	GO:0006118	0,004112859	electron transport
Δwc2	12-day	2623196	down	GO:0006118	0,004112859	electron transport
Δwc2	12-day	2625667	down	GO:0006118	0,004112859	electron transport
Δwc2	12-day	2625951	down	GO:0006118	0,004112859	electron transport
Δwc2	12-day	2628312	down	GO:0006118	0,004112859	electron transport
Δwc2	12-day	2628645	down	GO:0006118	0,004112859	electron transport
Δwc2	12-day	2628651	down	GO:0006118	0,004112859	electron transport
Δwc2	12-day	2629864	down	GO:0006118	0,004112859	electron transport
Δwc2	12-day	2632136	down	GO:0006118	0,004112859	electron transport
Δwc2	12-day	2632925	down	GO:0006118	0,004112859	electron transport
Δwc2	12-day	2633167	down	GO:0006118	0,004112859	electron transport
Δwc2	12-day	2633776	down	GO:0006118	0,004112859	electron transport
Δwc2	12-day	2634283	down	GO:0006118	0,004112859	electron transport
Δwc2	12-day	2635487	down	GO:0006118	0,004112859	electron transport
Δwc2	12-day	2635869	down	GO:0006118	0,004112859	electron transport
Δwc2	12-day	2636544	down	GO:0006118	0,004112859	electron transport
Δwc2	12-day	2637190	down	GO:0006118	0,004112859	electron transport
Δwc2	12-day	2638355	down	GO:0006118	0,004112859	electron transport
Δwc2	12-day	2639682	down	GO:0006118	0,004112859	electron transport
Δwc2	12-day	2644430	down	GO:0006118	0,004112859	electron transport
Δwc2	12-day	2662585	down	GO:0006118	0,004112859	electron transport
Δwc2	12-day	2663372	down	GO:0006118	0,004112859	electron transport
Δwc2	12-day	2668568	down	GO:0006118	0,004112859	electron transport
Δwc2	12-day	2703628	down	GO:0006118	0,004112859	electron transport
Δwc2	12-day	2703966	down	GO:0006118	0,004112859	electron transport
Δwc2	12-day	2735179	down	GO:0006118	0,004112859	electron transport
Δwc2	12-day	1038608	down	GO:0016020	0,008510813	membrane
Δwc2	12-day	1144263	down	GO:0016020	0,008510813	membrane

Δwc2	12-day	1153136	down	GO:0016020	0,008510813	membrane
Δwc2	12-day	1176319	down	GO:0016020	0,008510813	membrane
Δwc2	12-day	1267603	down	GO:0016020	0,008510813	membrane
Δwc2	12-day	1352010	down	GO:0016020	0,008510813	membrane
Δwc2	12-day	2035630	down	GO:0016020	0,008510813	membrane
Δwc2	12-day	2138287	down	GO:0016020	0,008510813	membrane
Δwc2	12-day	2162840	down	GO:0016020	0,008510813	membrane
Δwc2	12-day	2376858	down	GO:0016020	0,008510813	membrane
Δwc2	12-day	2488542	down	GO:0016020	0,008510813	membrane
Δwc2	12-day	2494727	down	GO:0016020	0,008510813	membrane
Δwc2	12-day	2514012	down	GO:0016020	0,008510813	membrane
Δwc2	12-day	2525305	down	GO:0016020	0,008510813	membrane
Δwc2	12-day	2569923	down	GO:0016020	0,008510813	membrane
Δwc2	12-day	2600551	down	GO:0016020	0,008510813	membrane
Δwc2	12-day	2600584	down	GO:0016020	0,008510813	membrane
Δwc2	12-day	2602793	down	GO:0016020	0,008510813	membrane
Δwc2	12-day	2604437	down	GO:0016020	0,008510813	membrane
Δwc2	12-day	2605277	down	GO:0016020	0,008510813	membrane
Δwc2	12-day	2607008	down	GO:0016020	0,008510813	membrane
Δwc2	12-day	2609987	down	GO:0016020	0,008510813	membrane
Δwc2	12-day	2610450	down	GO:0016020	0,008510813	membrane
Δwc2	12-day	2612806	down	GO:0016020	0,008510813	membrane
Δwc2	12-day	2613255	down	GO:0016020	0,008510813	membrane
Δwc2	12-day	2616602	down	GO:0016020	0,008510813	membrane
Δwc2	12-day	2618425	down	GO:0016020	0,008510813	membrane
Δwc2	12-day	2618479	down	GO:0016020	0,008510813	membrane
Δwc2	12-day	2618895	down	GO:0016020	0,008510813	membrane
Δwc2	12-day	2618950	down	GO:0016020	0,008510813	membrane
Δwc2	12-day	2621162	down	GO:0016020	0,008510813	membrane
Δwc2	12-day	2622595	down	GO:0016020	0,008510813	membrane
Δwc2	12-day	2622598	down	GO:0016020	0,008510813	membrane
Δwc2	12-day	2623420	down	GO:0016020	0,008510813	membrane
Δwc2	12-day	2626064	down	GO:0016020	0,008510813	membrane
Δwc2	12-day	2627703	down	GO:0016020	0,008510813	membrane

Δwc2	12-day	2627998	down	GO:0016020	0,008510813	membrane
Δwc2	12-day	2629091	down	GO:0016020	0,008510813	membrane
Δwc2	12-day	2638261	down	GO:0016020	0,008510813	membrane
Δwc2	12-day	2639525	down	GO:0016020	0,008510813	membrane
Δwc2	12-day	2640951	down	GO:0016020	0,008510813	membrane
Δwc2	12-day	2640999	down	GO:0016020	0,008510813	membrane
Δwc2	12-day	2642553	down	GO:0016020	0,008510813	membrane
Δwc2	12-day	2644280	down	GO:0016020	0,008510813	membrane
Δwc2	12-day	2645850	down	GO:0016020	0,008510813	membrane
Δwc2	12-day	2663372	down	GO:0016020	0,008510813	membrane
Δwc2	12-day	2670534	down	GO:0016020	0,008510813	membrane
Δwc2	12-day	2673672	down	GO:0016020	0,008510813	membrane
Δwc2	12-day	2701571	down	GO:0016020	0,008510813	membrane
Δwc2	12-day	2738153	down	GO:0016020	0,008510813	membrane
Δwc2	12-day	78628	down	GO:0004497	0,011860413	monooxygenase activity
Δwc2	12-day	81631	down	GO:0004497	0,011860413	monooxygenase activity
Δwc2	12-day	1131542	down	GO:0004497	0,011860413	monooxygenase activity
Δwc2	12-day	1189794	down	GO:0004497	0,011860413	monooxygenase activity
Δwc2	12-day	1193647	down	GO:0004497	0,011860413	monooxygenase activity
Δwc2	12-day	2327903	down	GO:0004497	0,011860413	monooxygenase activity
Δwc2	12-day	2484868	down	GO:0004497	0,011860413	monooxygenase activity
Δwc2	12-day	2486680	down	GO:0004497	0,011860413	monooxygenase activity
Δwc2	12-day	2510129	down	GO:0004497	0,011860413	monooxygenase activity
Δwc2	12-day	2510618	down	GO:0004497	0,011860413	monooxygenase activity
Δwc2	12-day	2512276	down	GO:0004497	0,011860413	monooxygenase activity
Δwc2	12-day	2515850	down	GO:0004497	0,011860413	monooxygenase activity
Δwc2	12-day	2583618	down	GO:0004497	0,011860413	monooxygenase activity
Δwc2	12-day	2604096	down	GO:0004497	0,011860413	monooxygenase activity
Δwc2	12-day	2604231	down	GO:0004497	0,011860413	monooxygenase activity
Δwc2	12-day	2604458	down	GO:0004497	0,011860413	monooxygenase activity
Δwc2	12-day	2604735	down	GO:0004497	0,011860413	monooxygenase activity
Δwc2	12-day	2611234	down	GO:0004497	0,011860413	monooxygenase activity
Δwc2	12-day	2616301	down	GO:0004497	0,011860413	monooxygenase activity
Δwc2	12-day	2619674	down	GO:0004497	0,011860413	monooxygenase activity

Δwc2	12-day	2619840	down	GO:0004497	0,011860413	monooxygenase activity
Δwc2	12-day	2623115	down	GO:0004497	0,011860413	monooxygenase activity
Δwc2	12-day	2623122	down	GO:0004497	0,011860413	monooxygenase activity
Δwc2	12-day	2623196	down	GO:0004497	0,011860413	monooxygenase activity
Δwc2	12-day	2628312	down	GO:0004497	0,011860413	monooxygenase activity
Δwc2	12-day	2628645	down	GO:0004497	0,011860413	monooxygenase activity
Δwc2	12-day	2628651	down	GO:0004497	0,011860413	monooxygenase activity
Δwc2	12-day	2629864	down	GO:0004497	0,011860413	monooxygenase activity
Δwc2	12-day	2632925	down	GO:0004497	0,011860413	monooxygenase activity
Δwc2	12-day	2633776	down	GO:0004497	0,011860413	monooxygenase activity
Δwc2	12-day	2634283	down	GO:0004497	0,011860413	monooxygenase activity
Δwc2	12-day	2635487	down	GO:0004497	0,011860413	monooxygenase activity
Δwc2	12-day	2635869	down	GO:0004497	0,011860413	monooxygenase activity
Δwc2	12-day	2636544	down	GO:0004497	0,011860413	monooxygenase activity
Δwc2	12-day	2638355	down	GO:0004497	0,011860413	monooxygenase activity
Δwc2	12-day	2644430	down	GO:0004497	0,011860413	monooxygenase activity
Δwc2	12-day	2668568	down	GO:0004497	0,011860413	monooxygenase activity
Δwc2	12-day	2703628	down	GO:0004497	0,011860413	monooxygenase activity
Δwc2	12-day	2703966	down	GO:0004497	0,011860413	monooxygenase activity
Δwc2	12-day	2735179	down	GO:0004497	0,011860413	monooxygenase activity
Δwc2	12-day	52195	down	GO:0005524	0,012210054	ATP binding
Δwc2	12-day	1165803	down	GO:0005524	0,012210054	ATP binding
Δwc2	12-day	1169398	down	GO:0005524	0,012210054	ATP binding
Δwc2	12-day	1184723	down	GO:0005524	0,012210054	ATP binding
Δwc2	12-day	1284934	down	GO:0005524	0,012210054	ATP binding
Δwc2	12-day	2018969	down	GO:0005524	0,012210054	ATP binding
Δwc2	12-day	2459580	down	GO:0005524	0,012210054	ATP binding
Δwc2	12-day	2482187	down	GO:0005524	0,012210054	ATP binding
Δwc2	12-day	2482745	down	GO:0005524	0,012210054	ATP binding
Δwc2	12-day	2493870	down	GO:0005524	0,012210054	ATP binding
Δwc2	12-day	2512873	down	GO:0005524	0,012210054	ATP binding
Δwc2	12-day	2563608	down	GO:0005524	0,012210054	ATP binding
Δwc2	12-day	2614908	down	GO:0005524	0,012210054	ATP binding
Δwc2	12-day	2615950	down	GO:0005524	0,012210054	ATP binding

Δwc2	12-day	2616602	down	GO:0005524	0,012210054	ATP binding
Δwc2	12-day	2617727	down	GO:0005524	0,012210054	ATP binding
Δwc2	12-day	2618609	down	GO:0005524	0,012210054	ATP binding
Δwc2	12-day	2619461	down	GO:0005524	0,012210054	ATP binding
Δwc2	12-day	2621581	down	GO:0005524	0,012210054	ATP binding
Δwc2	12-day	2636914	down	GO:0005524	0,012210054	ATP binding
Δwc2	12-day	2637190	down	GO:0005524	0,012210054	ATP binding
Δwc2	12-day	2642188	down	GO:0005524	0,012210054	ATP binding
Δwc2	12-day	2664859	down	GO:0005524	0,012210054	ATP binding
Δwc2	12-day	2683832	down	GO:0005524	0,012210054	ATP binding
Δwc2	12-day	2690708	down	GO:0005524	0,012210054	ATP binding
Δwc2	12-day	2695418	down	GO:0005524	0,012210054	ATP binding
Δwc2	12-day	2702315	down	GO:0005524	0,012210054	ATP binding
Δwc2	12-day	2748506	down	GO:0005524	0,012210054	ATP binding
Δwc2	12-day	2628225	down	GO:0005199	0,015745001	structural constituent of cell wall
Δwc2	12-day	2629603	down	GO:0005199	0,015745001	structural constituent of cell wall
Δwc2	12-day	2629632	down	GO:0005199	0,015745001	structural constituent of cell wall
Δwc2	12-day	2746250	down	GO:0005199	0,015745001	structural constituent of cell wall
Δwc2	12-day	2514012	down	GO:0006865	0,016123014	amino acid transport
Δwc2	12-day	2514012	down	GO:0015171	0,016123014	amino acid transmembrane transporter activity
Δwc2	12-day	2068434	down	GO:0030170	0,016261102	pyridoxal phosphate binding
Δwc2	12-day	2553610	down	GO:0030170	0,016261102	pyridoxal phosphate binding
Δwc2	12-day	2608099	down	GO:0030170	0,016261102	pyridoxal phosphate binding
Δwc2	12-day	2623956	down	GO:0030170	0,016261102	pyridoxal phosphate binding
Δwc2	12-day	2614257	down	GO:0005618	0,020523197	cell wall
Δwc2	12-day	2628225	down	GO:0005618	0,020523197	cell wall
Δwc2	12-day	2629603	down	GO:0005618	0,020523197	cell wall
Δwc2	12-day	2629632	down	GO:0005618	0,020523197	cell wall
Δwc2	12-day	1144263	down	GO:0005215	0,023675553	transporter activity
Δwc2	12-day	1153136	down	GO:0005215	0,023675553	transporter activity
Δwc2	12-day	1154281	down	GO:0005215	0,023675553	transporter activity
Δwc2	12-day	1357952	down	GO:0005215	0,023675553	transporter activity
Δwc2	12-day	2122325	down	GO:0005215	0,023675553	transporter activity
Δwc2	12-day	2138287	down	GO:0005215	0,023675553	transporter activity

Δwc2	12-day	2162840	down	GO:0005215	0,023675553	transporter activity
Δwc2	12-day	2481653	down	GO:0005215	0,023675553	transporter activity
Δwc2	12-day	2492878	down	GO:0005215	0,023675553	transporter activity
Δwc2	12-day	2494144	down	GO:0005215	0,023675553	transporter activity
Δwc2	12-day	2520877	down	GO:0005215	0,023675553	transporter activity
Δwc2	12-day	2525305	down	GO:0005215	0,023675553	transporter activity
Δwc2	12-day	2600551	down	GO:0005215	0,023675553	transporter activity
Δwc2	12-day	2607025	down	GO:0005215	0,023675553	transporter activity
Δwc2	12-day	2607891	down	GO:0005215	0,023675553	transporter activity
Δwc2	12-day	2608678	down	GO:0005215	0,023675553	transporter activity
Δwc2	12-day	2611816	down	GO:0005215	0,023675553	transporter activity
Δwc2	12-day	2613255	down	GO:0005215	0,023675553	transporter activity
Δwc2	12-day	2619645	down	GO:0005215	0,023675553	transporter activity
Δwc2	12-day	2620178	down	GO:0005215	0,023675553	transporter activity
Δwc2	12-day	2620830	down	GO:0005215	0,023675553	transporter activity
Δwc2	12-day	2621828	down	GO:0005215	0,023675553	transporter activity
Δwc2	12-day	2622598	down	GO:0005215	0,023675553	transporter activity
Δwc2	12-day	2623420	down	GO:0005215	0,023675553	transporter activity
Δwc2	12-day	2623492	down	GO:0005215	0,023675553	transporter activity
Δwc2	12-day	2626803	down	GO:0005215	0,023675553	transporter activity
Δwc2	12-day	2627703	down	GO:0005215	0,023675553	transporter activity
Δwc2	12-day	2628751	down	GO:0005215	0,023675553	transporter activity
Δwc2	12-day	2629091	down	GO:0005215	0,023675553	transporter activity
Δwc2	12-day	2629455	down	GO:0005215	0,023675553	transporter activity
Δwc2	12-day	2636597	down	GO:0005215	0,023675553	transporter activity
Δwc2	12-day	2637509	down	GO:0005215	0,023675553	transporter activity
Δwc2	12-day	2640951	down	GO:0005215	0,023675553	transporter activity
Δwc2	12-day	2642018	down	GO:0005215	0,023675553	transporter activity
Δwc2	12-day	2642553	down	GO:0005215	0,023675553	transporter activity
Δwc2	12-day	2661224	down	GO:0005215	0,023675553	transporter activity
Δwc2	12-day	2701571	down	GO:0005215	0,023675553	transporter activity
Δwc2	12-day	2712060	down	GO:0005215	0,023675553	transporter activity
Δwc2	12-day	2738153	down	GO:0005215	0,023675553	transporter activity
Δwc2	12-day	2577992	down	GO:0004194	0,02444899	pepsin A activity

Δwc2	12-day	2613917	down	GO:0004194	0,02444899	pepsin A activity
Δwc2	12-day	2616949	down	GO:0004194	0,02444899	pepsin A activity
Δwc2	12-day	2616991	down	GO:0004194	0,02444899	pepsin A activity
Δwc2	12-day	2619340	down	GO:0004194	0,02444899	pepsin A activity
Δwc2	12-day	2619343	down	GO:0004194	0,02444899	pepsin A activity
Δwc2	12-day	78628	down	GO:0020037	0,026908024	heme binding
Δwc2	12-day	81631	down	GO:0020037	0,026908024	heme binding
Δwc2	12-day	1131542	down	GO:0020037	0,026908024	heme binding
Δwc2	12-day	1189794	down	GO:0020037	0,026908024	heme binding
Δwc2	12-day	1193647	down	GO:0020037	0,026908024	heme binding
Δwc2	12-day	2327903	down	GO:0020037	0,026908024	heme binding
Δwc2	12-day	2376858	down	GO:0020037	0,026908024	heme binding
Δwc2	12-day	2484868	down	GO:0020037	0,026908024	heme binding
Δwc2	12-day	2486680	down	GO:0020037	0,026908024	heme binding
Δwc2	12-day	2497674	down	GO:0020037	0,026908024	heme binding
Δwc2	12-day	2507891	down	GO:0020037	0,026908024	heme binding
Δwc2	12-day	2510129	down	GO:0020037	0,026908024	heme binding
Δwc2	12-day	2510618	down	GO:0020037	0,026908024	heme binding
Δwc2	12-day	2512276	down	GO:0020037	0,026908024	heme binding
Δwc2	12-day	2583618	down	GO:0020037	0,026908024	heme binding
Δwc2	12-day	2604096	down	GO:0020037	0,026908024	heme binding
Δwc2	12-day	2604231	down	GO:0020037	0,026908024	heme binding
Δwc2	12-day	2604458	down	GO:0020037	0,026908024	heme binding
Δwc2	12-day	2604628	down	GO:0020037	0,026908024	heme binding
Δwc2	12-day	2604735	down	GO:0020037	0,026908024	heme binding
Δwc2	12-day	2609840	down	GO:0020037	0,026908024	heme binding
Δwc2	12-day	2611234	down	GO:0020037	0,026908024	heme binding
Δwc2	12-day	2614998	down	GO:0020037	0,026908024	heme binding
Δwc2	12-day	2616301	down	GO:0020037	0,026908024	heme binding
Δwc2	12-day	2619674	down	GO:0020037	0,026908024	heme binding
Δwc2	12-day	2621855	down	GO:0020037	0,026908024	heme binding
Δwc2	12-day	2623115	down	GO:0020037	0,026908024	heme binding
Δwc2	12-day	2623122	down	GO:0020037	0,026908024	heme binding
Δwc2	12-day	2623196	down	GO:0020037	0,026908024	heme binding

Δwc2	12-day	2624504	down	GO:0020037	0,026908024	heme binding
Δwc2	12-day	2628312	down	GO:0020037	0,026908024	heme binding
Δwc2	12-day	2628645	down	GO:0020037	0,026908024	heme binding
Δwc2	12-day	2628651	down	GO:0020037	0,026908024	heme binding
Δwc2	12-day	2629864	down	GO:0020037	0,026908024	heme binding
Δwc2	12-day	2632925	down	GO:0020037	0,026908024	heme binding
Δwc2	12-day	2633776	down	GO:0020037	0,026908024	heme binding
Δwc2	12-day	2634283	down	GO:0020037	0,026908024	heme binding
Δwc2	12-day	2635487	down	GO:0020037	0,026908024	heme binding
Δwc2	12-day	2635869	down	GO:0020037	0,026908024	heme binding
Δwc2	12-day	2636544	down	GO:0020037	0,026908024	heme binding
Δwc2	12-day	2637190	down	GO:0020037	0,026908024	heme binding
Δwc2	12-day	2638355	down	GO:0020037	0,026908024	heme binding
Δwc2	12-day	2668568	down	GO:0020037	0,026908024	heme binding
Δwc2	12-day	2703628	down	GO:0020037	0,026908024	heme binding
Δwc2	12-day	2703966	down	GO:0020037	0,026908024	heme binding
Δwc2	12-day	2704129	down	GO:0020037	0,026908024	heme binding
Δwc2	12-day	2735179	down	GO:0020037	0,026908024	heme binding
Δwc2	12-day	2285680	down	GO:0006629	0,030389288	lipid metabolic process
Δwc2	12-day	2376858	down	GO:0006629	0,030389288	lipid metabolic process
Δwc2	12-day	2641759	down	GO:0006629	0,030389288	lipid metabolic process
Δwc2	12-day	1036421	down	GO:0016021	0,039729267	integral to membrane
Δwc2	12-day	1038608	down	GO:0016021	0,039729267	integral to membrane
Δwc2	12-day	1144263	down	GO:0016021	0,039729267	integral to membrane
Δwc2	12-day	1147332	down	GO:0016021	0,039729267	integral to membrane
Δwc2	12-day	1153136	down	GO:0016021	0,039729267	integral to membrane
Δwc2	12-day	1154281	down	GO:0016021	0,039729267	integral to membrane
Δwc2	12-day	1172581	down	GO:0016021	0,039729267	integral to membrane
Δwc2	12-day	1186839	down	GO:0016021	0,039729267	integral to membrane
Δwc2	12-day	2122325	down	GO:0016021	0,039729267	integral to membrane
Δwc2	12-day	2138287	down	GO:0016021	0,039729267	integral to membrane
Δwc2	12-day	2481653	down	GO:0016021	0,039729267	integral to membrane
Δwc2	12-day	2483267	down	GO:0016021	0,039729267	integral to membrane
Δwc2	12-day	2494144	down	GO:0016021	0,039729267	integral to membrane

Δwc2	12-day	2503108	down	GO:0016021	0,039729267	integral to membrane
Δwc2	12-day	2514012	down	GO:0016021	0,039729267	integral to membrane
Δwc2	12-day	2524870	down	GO:0016021	0,039729267	integral to membrane
Δwc2	12-day	2525305	down	GO:0016021	0,039729267	integral to membrane
Δwc2	12-day	2572308	down	GO:0016021	0,039729267	integral to membrane
Δwc2	12-day	2604984	down	GO:0016021	0,039729267	integral to membrane
Δwc2	12-day	2607025	down	GO:0016021	0,039729267	integral to membrane
Δwc2	12-day	2607891	down	GO:0016021	0,039729267	integral to membrane
Δwc2	12-day	2608678	down	GO:0016021	0,039729267	integral to membrane
Δwc2	12-day	2611612	down	GO:0016021	0,039729267	integral to membrane
Δwc2	12-day	2611816	down	GO:0016021	0,039729267	integral to membrane
Δwc2	12-day	2613255	down	GO:0016021	0,039729267	integral to membrane
Δwc2	12-day	2616602	down	GO:0016021	0,039729267	integral to membrane
Δwc2	12-day	2619645	down	GO:0016021	0,039729267	integral to membrane
Δwc2	12-day	2620178	down	GO:0016021	0,039729267	integral to membrane
Δwc2	12-day	2620830	down	GO:0016021	0,039729267	integral to membrane
Δwc2	12-day	2621828	down	GO:0016021	0,039729267	integral to membrane
Δwc2	12-day	2622598	down	GO:0016021	0,039729267	integral to membrane
Δwc2	12-day	2623420	down	GO:0016021	0,039729267	integral to membrane
Δwc2	12-day	2623492	down	GO:0016021	0,039729267	integral to membrane
Δwc2	12-day	2626803	down	GO:0016021	0,039729267	integral to membrane
Δwc2	12-day	2627703	down	GO:0016021	0,039729267	integral to membrane
Δwc2	12-day	2629091	down	GO:0016021	0,039729267	integral to membrane
Δwc2	12-day	2629455	down	GO:0016021	0,039729267	integral to membrane
Δwc2	12-day	2636597	down	GO:0016021	0,039729267	integral to membrane
Δwc2	12-day	2637190	down	GO:0016021	0,039729267	integral to membrane
Δwc2	12-day	2637509	down	GO:0016021	0,039729267	integral to membrane
Δwc2	12-day	2638261	down	GO:0016021	0,039729267	integral to membrane
Δwc2	12-day	2641709	down	GO:0016021	0,039729267	integral to membrane
Δwc2	12-day	2642018	down	GO:0016021	0,039729267	integral to membrane
Δwc2	12-day	2663372	down	GO:0016021	0,039729267	integral to membrane
Δwc2	12-day	2667661	down	GO:0016021	0,039729267	integral to membrane
Δwc2	12-day	2686473	down	GO:0016021	0,039729267	integral to membrane
Δwc2	12-day	2691538	down	GO:0016021	0,039729267	integral to membrane

Δwc2	12-day	2701571	down	GO:0016021	0,039729267	integral to membrane
Δwc2	12-day	2704867	down	GO:0016021	0,039729267	integral to membrane
Δwc2	12-day	2712060	down	GO:0016021	0,039729267	integral to membrane
Δwc2	12-day	2376858	down	GO:0016717	0,048950793	oxidoreductase activity, acting on paired donors

Supplementary Table S4. Impact of deletion of transcription factor genes on whole genome expression. Enrichment of GO terms in up- and downregulated genes of 12-day-old colonies of Δ*wc-1*Δ*wc-1* and transcription factor deletion strains Δ*wc-2*Δ*wc-2*, Δ*hom2*Δ*hom2*, Δ*fst4*Δ*fst4*, Δ*c2h2*Δ*c2h2*, Δ*fst3*Δ*fst3*, Δ*hom1*Δ*hom1*, Δ*gat1*Δ*gat1*, and Δ*briI*Δ*briI* when compared to the fruiting wild-type strain. Analysis is based on biological duplicates.

Δwc-1Δwc-1

Up-regulated genes in mutant strain		Down-regulated genes in mutant strain	
GO term	Functional annotation	GO term	Functional annotation
0005506	iron ion binding	0008152	metabolic process
0020037	heme binding	0016491	oxidoreductase activity
0004497	monooxygenase activity	0000786	nucleosome
0006118	electron transport	0006334	nucleosome assembly
0050381	unspecific monooxygenase activity	0003824	catalytic activity
0005975	carbohydrate metabolic process	0006810	transport
0004553	hydrolase activity, hydrolyzing O-glycosyl compounds	0016020	membrane
0005524	ATP binding	0004497	monooxygenase activity
0005215	transporter activity	0016021	integral to membrane
0016810	hydrolase activity, acting on carbon-nitrogen (but not peptide)	0006118	electron transport

Δwc-2Δwc-2

Up-regulated genes in mutant strain		Down-regulated genes in mutant strain	
GO term	Functional annotation	GO term	Functional annotation
0020037	heme binding	0000786	nucleosome
0005506	iron ion binding	0006334	nucleosome assembly
0004497	monooxygenase activity	0003677	DNA binding
0050381	unspecific monooxygenase activity	0003824	catalytic activity
0005975	carbohydrate metabolic process	0008152	metabolic process
0006118	electron transport	0006260	DNA replication
0004553	hydrolase activity, hydrolyzing O-glycosyl compounds	0042624	ATPase activity, uncoupled
0005215	transporter activity	0016491	oxidoreductase activity
0005524	ATP binding	0042623	ATPase activity, coupled
0006032	chitin catabolic process	0008186	RNA-dependent ATPase activity

Δhom2Δhom2

Up-regulated genes in mutant strain		Down-regulated genes in mutant strain	
GO term	Functional annotation	GO term	Functional annotation

0003735	structural constituent of ribosome	0006810	transport
0005840	ribosome	0005215	transporter activity
0006412	translation	0008152	metabolic process
0005975	carbohydrate metabolic process	0016020	membrane
0005506	iron ion binding	0016021	integral to membrane
0020037	heme binding	0016491	oxidoreductase activity
0004553	hydrolase activity, hydrolyzing O-glycosyl compounds	0003824	catalytic activity
0005622	intracellular	0006865	amino acid transport
0004497	monooxygenase activity	0015171	amino acid transmembrane transporter activity
0006118	electron transport	0004497	monooxygenase activity

Δfst4Δfst4

Up-regulated genes in mutant strain		Down-regulated genes in mutant strain	
GO term	Functional annotation	GO term	Functional annotation
0005975	carbohydrate metabolic process	0016491	oxidoreductase activity
0005215	transporter activity	0008152	metabolic process
0006508	proteolysis	0003824	catalytic activity
0005506	iron ion binding	0000786	nucleosome
0020037	heme binding	0030170	pyridoxal phosphate binding
0050381	unspecific monooxygenase activity	0006334	nucleosome assembly
0004553	hydrolase activity, hydrolyzing O-glycosyl compounds	0006118	electron transport
0004497	monooxygenase activity	0006810	transport
0006118	electron transport	0008483	transaminase activity
0008236	serine-type peptidase activity	0005199	structural constituent of cell wall

Δc2h2Δc2h2

Up-regulated genes in mutant strain		Down-regulated genes in mutant strain	
GO term	Functional annotation	GO term	Functional annotation
0005506	iron ion binding	0003824	catalytic activity
0020037	heme binding	0008152	metabolic process
0004497	monooxygenase activity	0030170	pyridoxal phosphate binding
0006118	electron transport	0005199	structural constituent of cell wall
0050381	unspecic monooxygenase activity	0005618	cell wall

0016711	flavonoid 3'-monooxygenase activity	0005992	trehalose biosynthetic process
0004553	hydrolase activity, hydrolyzing O-glycosyl compounds	0005488	binding
0005975	hydrolase activity, hydrolyzing O-glycosyl compounds	0004316	3-oxoacyl-[acyl-carrier-protein] reductase activity
0006032	carbohydrate metabolic process	0033754	indoleamine 2,3-dioxygenase activity
0005618	chitin catabolic process	0006164	purine nucleotide biosynthetic process

Δfst3Δfst3

Up-regulated genes in mutant strain		Down-regulated genes in mutant strain	
GO term	Functional annotation	GO term	Functional annotation
0004565	beta-galactosidase activity	0004568	chitinase activity
0009341	beta-galactosidase complex	0008843	endochitinase activity
0008168	methyltransferase activity	0005576	extracellular region
0005975	carbohydrate metabolic process	0004553	hydrolase activity, hydrolyzing O-glycosyl compounds
0009316	3-isopropylmalate dehydratase complex	0006032	chitin catabolic process
0003861	3-isopropylmalate dehydratase activity	0005199	structural constituent of cell wall
0004035	alkaline phosphatase activity	0005618	cell wall
0005385	zinc ion transmembrane transporter activity	0030246	carbohydrate binding
0006829	zinc ion transport	0050381	unspecific monooxygenase activity
0005315	inorganic phosphate transmembrane transporter activity	0005975	carbohydrate metabolic process

Δhom1Δhom1

Up-regulated genes in mutant strain		Down-regulated genes in mutant strain	
GO term	Functional annotation	GO term	Functional annotation
	NONE	0003824	catalytic activity
		0016491	oxidoreductase activity
		0004316	3-oxoacyl-[acyl-carrier-protein] reductase activity
		0008152	metabolic process
		0006526	arginine biosynthetic process
		0016021	integral to membrane
		0003939	L-iditol 2-dehydrogenase activity
		0004932	mating-type factor pheromone receptor activity
		0003991	acetylglutamate kinase activity
		0004358	glutamate N-acetyltransferase activity

Δgat1Δgat1

Up-regulated genes in mutant strain		Down-regulated genes in mutant strain	
GO term	Functional annotation	GO term	Functional annotation
0017059	serine C-palmitoyltransferase complex	0005840	ribosome
0004758	serine C-palmitoyltransferase activity	0003735	structural constituent of ribosome
		0006412	translation
		0005622	intracellular
		0005839	proteasome core complex
		0005199	structural constituent of cell wall
		0005618	cell wall
		0008152	metabolic process
		0006118	electron transport
		0006526	arginine biosynthetic process

Δbri1Δbri1

Up-regulated genes in mutant strain		Down-regulated genes in mutant strain	
GO term	Functional annotation	GO term	Functional annotation
	NONE	0005975	carbohydrate metabolic process
		0004553	hydrolase activity, hydrolyzing O-glycosyl compounds
		0016491	oxidoreductase activity
		0004364	glutathione transferase activity
		0005618	cell wall
		0003824	catalytic activity
		0004568	chitinase activity
		0005199	structural constituent of cell wall
		0008843	endochitinase activity
		0016564	transcription repressor activity

Supplementary Table S5. Impact of deletion of transcription factor genes on other transcription factor genes. ProteinIDs and annotations of transcription factors significantly up- or downregulated among Δ*hom1*, Δ*gat1* and Δ*fst3* at fruiting body stage and among Δ*wc-2*, Δ*hom2* and Δ*fst4* at aggregate stage. Analysis is based on biological duplicates.

Fruiting Body

UPREGULATED

**Δ*hom1***		**Δ*gat1***		**Δ*fst3***	
2609438	Zinc finger, C2H2-type	2631700	Zn2Cys6	**2622753**	Zn2Cys6
		2568997	Dimeric alpha-beta barrel	**2641338**	Zinc finger, C2H2-type
		**2686461**	High mobility group box, HMG1/HMG2	2605931	Zn2Cys6
		**2529565**	Fork head	2644742	Zinc finger, C2H2-type
		1163017	ABC transporter/ Zinc finger, C2H2-type		
		**1183987**	Forkhead		
		2693949	Homeodomain		
		**2585708**	Zinc Finger		
		1201831	Zn2Cys6		
		**2743676**	Zinc finger, C2H2-type		
		**2511560**	Zinc finger, C2H2-type		
		2526629	Homeodomain		
		2690968	Nuclease		
		**2687474**	Zinc finger, C2H2-type		
		**2604883**	Zinc finger, C2H2-type		
		**2520583**	Zinc finger, C2H2-type		
		2629777	Zinc finger, C2H2-type		
		**2674381**	Fungal specific transcription factor		

Δ*hom1* and Δ*gat1*

2481568	TEA/ATTS
**2625140**	APSES
**2594118**	Zinc finger, C2H2-type
**2668745**	Homeodomain
2524459	Zn2Cys6
2667924	Zinc finger, C2H2-type
**1157466**	Basic-leucine zipper
2533051	Zn2Cys6
2686265	Zinc finger, C2H2-type
**2493743**	Zn2Cys6

Δ*hom1* and Δ*gat1* and Δ*fst3*

2621155	HMG-I/HMG-Y

DOWNREGULATED

**Δ*hom1***		**Δ*gat1***		**Δ*fst3***	
2603970	Homeodomain	2634258	GHMP kinase	**2629275**	Zn2Cys6
		2617618	Zinc finger, C2H2-type		
		**2635354**	TEA/ATTS		

		2605931	Zn2Cys6		
		2516476	Zinc finger, C2H2-type		
		2625706	Cyanate lyase / lambda repressor-like DNA binding domain		
		2517503	High mobility group box, HMG1/HMG2		
		**2634413**	LAG1		
		**2045557**	High mobility group box, HMG1/HMG2		
		2619914	Zinc finger, C2H2-type		
		**2665755**	Zinc finger, C2H2-type		
		**2635786**	Helix-turn-helix, AraC type		
		**269971**	Zinc finger, C2H2-type		
		2642686	Zinc finger, C2H2-type		
		1115145	Homeodomain		
		2646024	Zinc finger, CCHC		
		**2633839**	Zn2Cys6		
		2644742	Zinc finger, C2H2-type		
		2632395	Zn2Cys6		

Δ*gat1* and Δ*fst3*

2703923	Zinc finger, C2H2-type

Δ*hom1* and Δ*gat1*

2620880	Fungal specific transcription factor
**2629570**	Zinc finger, C2H2-type
**2608261**	High mobility group box, HMG1/HMG2
2488050	Zinc finger, C2H2-type
**2573899**	Zn2Cys6
2532312	Zinc finger, C2H2-type

Aggregate

UPREGULATED

**Δ*wc-2***		**Δ*hom2***		**Δ*fst4***	
**1095746**	Fungal specific transcription factor	**2058879**	High mobility group box, HMG1/HMG2	**2645939**	HLH, helix-loop-helix DNA-binding domain
**2627285**	Zinc finger, C2H2-type	**2548256**	Zn2Cys6	**2509453**	Zinc finger, C2H2-type
2633009		2517503	High mobility group box, HMG1/HMG2	**2662836**	Zinc finger, C2H2-type
2488050	Zinc finger, C2H2-type	**1191002**	Fungal specific transcription factor	**2596857**	Fungal specific transcription factor
**2543273**	Zinc finger, CCHC-type	**2503711**	High mobility group box, HMG1/HMG2	**2610822**	Zinc finger, DHHC-type
2686265	Zinc finger, C2H2-type	**2625438**	Zinc finger, GATA-type	**2515078**	Zn2Cys6
		**2445444**	Zinc finger, CCHC-type	**2703742**	Zn2Cys6
		**1039007**	Zinc finger, C2H2-type	**2642040**	Fork head transcription factor
				2632395	Zn2Cys6

				**2619245**	Zinc finger, C2H2-type
				**2620175**	Zn2Cys6
				**2607126**	Helix-turn-helix type 3
				2625706	Cyanate lyase / lambda repressor-like DNA binding domain
				**2679824**	Zinc finger, C2H2-type
				2510481	Fungal specific transcription factor
				2667924	Zinc finger, C2H2-type
				**2626375**	Zinc finger, C2H2-type
				**2517617**	Zinc finger, C2H2-type
				**2354220**	Zinc finger, C2H2-type
				**250298**	Zn2Cys6
				**2690519**	Zinc finger, C2H2-type
				**1156706**	Zn2Cys6
				**2627369**	HLH, helix-loop-helix DNA-binding domain
				2605931	Zn2Cys6
				**2608348**	Zn2Cys6
				2623333	High mobility group box, HMG1/HMG2
				**2642807**	Zinc finger, C2H2-type
				**1192535**	SANT, DNA-binding
				**248956**	Zinc finger, C2H2-type
				**2507652**	Zinc finger, C2H2-type
				**2750667**	Zinc finger, DHHC-type
				**2620554**	Zinc finger, C2H2-type
				**2610202**	HLH, helix-loop-helix DNA-binding domain
				**2615561**	Zn2Cys6

Δ*wc-2* and Δ*hom2*

**2610649**	Basic-leucine zipper (bZIP) transcription factor
**2511242**	Zn2Cys6
**2497230**	Zinc finger, C2H2-type
2642686	Zinc finger, C2H2-type
2629777	Zinc finger, C2H2-type
1163017	ABC transporter/ Zinc finger, C2H2-type

Δ*wc-2* and Δ*fst4*

2621155	HMG-I/HMG-Y
2532312	Zinc finger, C2H2-type
2598013	Zn2Cys6

DOWNREGULATED

Δ*wc-2*		Δ*hom2*		Δ*fst4*	
**2521386**	Basic-leucine zipper (bZIP)	**2606207**	Transcription factor, MADS-box	2631700	Zn2Cys6

	transcription factor				
2533051	Zn2Cys6	2481568	TEA/ATTS	2568997	Dimeric alpha-beta barrel
2625706	Cyanate lyase / lambda repressor-like DNA binding domain	2516476	Zinc finger, C2H2-type	2603970	Homeodomain
		**2495664**	Zn2Cys6	**2628438**	HMG-I and HMG-Y, DNA-binding
		**2638002**	Basic-leucine zipper (bZIP) transcription factor	**2489133**	HMG-I and HMG-Y, DNA-binding
		1201831	Zn2Cys6	**2629009**	HMG-I and HMG-Y, DNA-binding
		**2631376**	Basic-leucine zipper (bZIP) transcription factor	**2631854**	Homeodomain
		**2661334**	High mobility group box, HMG	2526629	Homeodomain
		1115145	Homeodomain	**2616011**	SANT, DNA-binding
		**2634429**	Homeodomain	**2624428**	Zn2Cys6
				**2670552**	HMG-I and HMG-Y, DNA-binding
				**2666385**	HMG-I and HMG-Y, DNA-binding
				**2481326**	Jumonji
				**2499005**	Zinc finger, C2H2-type
				2609438	Zinc finger, C2H2-type
				**2197600**	Zn2Cys6
				2634258	GHMP kinase
				**2551688**	HMG-I and HMG-Y, DNA-binding
				**2663071**	Zinc finger, CCCH-type
				**2586122**	Homeodomain
				**70520**	Zinc finger, NF-X1-type
				**2496919**	Endonuclease
				**2632356**	Homeodomain
				**250177**	Fungal specific transcription factor
				**2558936**	Lambda repressor-like, DNA-binding
				2619914	Zinc finger, C2H2-type
				**2495286**	SANT, DNA-binding
				**2665806**	Ankyrin
				**2628233**	Zinc finger, TFIIS-type
				**2513276**	Zinc finger, C2H2-type
				2642686	Zinc finger, C2H2-type
				**2645097**	Zinc finger, CCHC-type
				2646024	Zinc finger, CCHC
				**2128569**	Transcription factor, MADS-box
				**2682446**	SANT, DNA-binding

		2690968	Nuclease
		**2696654**	Homeodomain
		**2645990**	High mobility group box, HMG1/HMG2
		2693949	Homeodomain
		**2616096**	Fungal specific transcription factor
		**2663876**	HMG-I and HMG-Y, DNA-binding
		**2615463**	Zn2Cys6
		**2486034**	Zinc finger, GATA-type

Δ*wc-2* and Δ*hom2*

**2630358**	Zinc finger, C2H2-type
**2525437**	Fungal specific transcription factor
2601101	Zinc finger, C2H2-type
2623333	High mobility group box, HMG1/HMG2

Δ*hom2* and Δ*fst4*

**2731703**	Jumonji
1194438	Lambda repressor-like, DNA-binding
**2620880**	Fungal specific transcription factor
**2668417**	Basic-leucine zipper (bZIP) transcription factor

Δ*wc-2* and Δ*hom2* and Δ*fst4*

2703923	Zinc finger, C2H2-type
2519514	TEA/ATTS

Analysis has been performed in version 3 of the *S. commune* genome annotation (http://genome.jgi.doe.gov/Schco3). Transcription factors are categorized into groups up- or downregulated exclusive or mutual to deletion strains. ProteinIDs in bold exist only once in this list.

Supplementary Table S6. Primers used in this study.

Δ2519514ufw	GGCCTAATAGGCCTAGAATGCGCTCTCCGTC
Δ2519514urv	GGCCTCGCAGGCCAGGGAGGATGACGCAAAG
Δ2519514dfw	GGCCTGCGAGGCCGTCCGTGTTCTTGGATAC
Δ2519514drv	GGCCTATTAGGCCGTGCGTTGTTTCGTTTCC
2519514ufcfw	TCCACGCTGGCTGAATAG
2519514dfcrv	TCGATGTGAGGTACTGTC
nourdelrev	TAAGCCGTGTCGTCA
sc3tersqf	CCGGGAATTCCAGAT
Δ2703923ufw	GGCCTAATAGGCCCTGTCACGCACCAGTACG
Δ2703923urv	GGCCTCGCAGGCCGGGCGAACGTGAGATAAG
Δ2703923dfw	GGCCTGCGAGGCCGCTGTGGACGGTCTTAAC
Δ2703923drv	GGCCTATTAGGCCCATTGCACGAGTCCATTC
c2h2dufcfw	AACGAGGGTCGTTGTATC
c2h2ddfcrv	GATGAAGACGCCCTCAATCG
hom2rtfw	GAGAGGTGTACAGGTCTG
hom2rtrv	ACTGGTTCGAGGTCATAG
tea1fw1	CACCTGCTATACATGCAGCGCGACGATAG
tea1rv1	GGATCCTTAGATCATGAAAGCGCCGCC

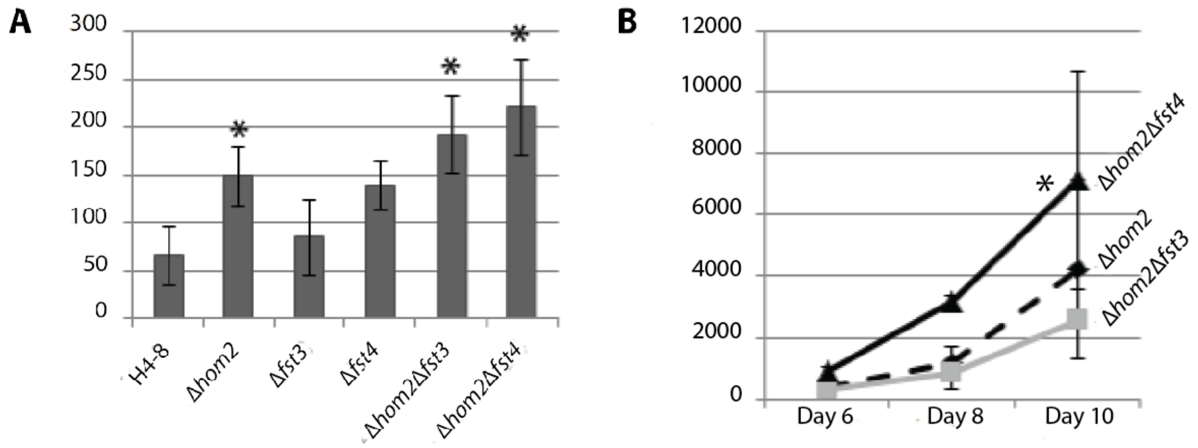

Supplemental Figure 1. Transcription factors of *S. commune* that affect biomass formation. Biomass of biological triplicates of 6-day-old dark-grown cultures of the wild-type dikaryon and transcription factor deletion strains that had been grown on agar medium (A) or as liquid shaken cultures (B) using glucose as a carbon source. *Indicates significant difference with wild-type strain H4-8 (A) or the Δ*hom2*Δ*hom2* strain (B).

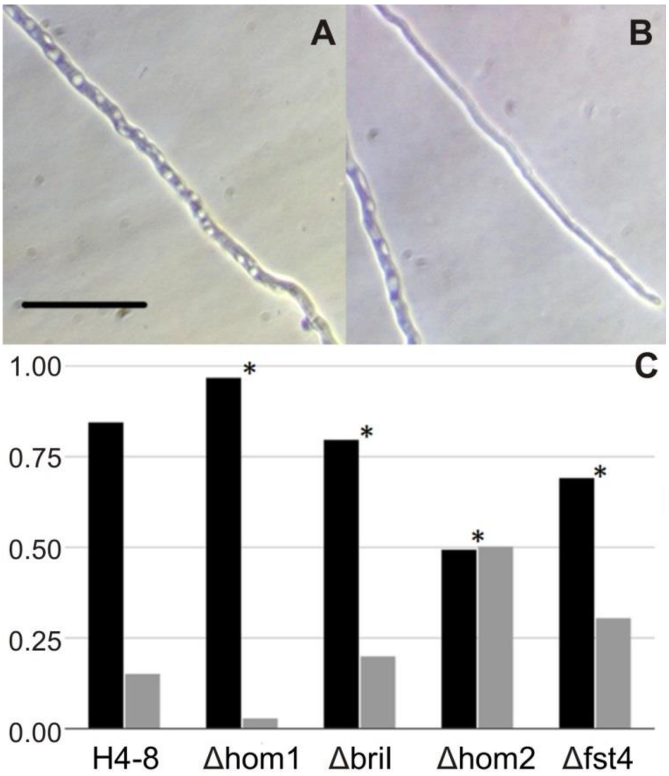

Supplemental Figure 2. *S. commune* forms vacuole-rich wide hyphae (A, B) and thin hyphae with few vacuoles (B). The ratio between the abundance of these hyphae differs between dikaryotic wild-type and the TF deletion strains when cultures were grown in the dark. * indicates significant difference ($p < 0,05$) using a χ^2 test. Bar represents 100 µm.

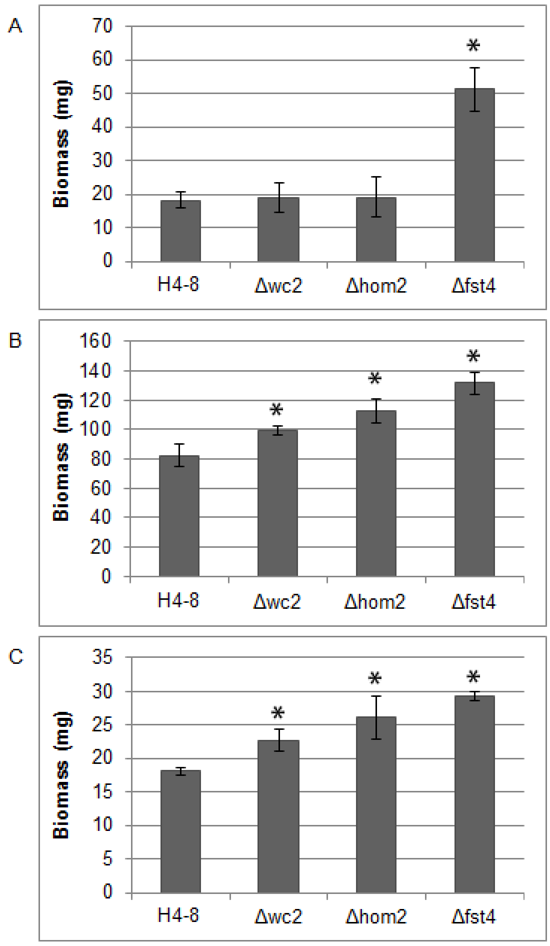

Supplementary Figure 3. Biomass of biological triplicates of 6-day-old dark-grown agar cultures of the wild-type dikaryon and transcription factor deletion strains using xylose (A), sucrose (B), and pectin (C) as carbon source. *Indicates significant difference with wild-type strain H4-8.

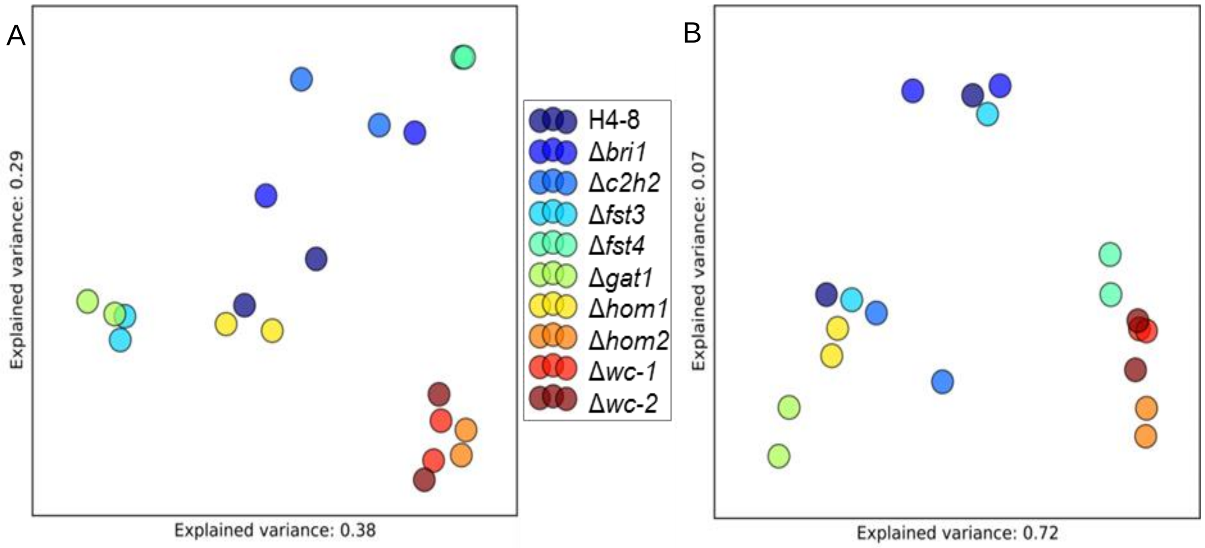

Supplementary Figure 4. Principal component analysis of whole genome expression profiles of biological duplicates of the transcription factor deletion strains of *S. commune* during aggregation (A) and fruiting body formation (B).

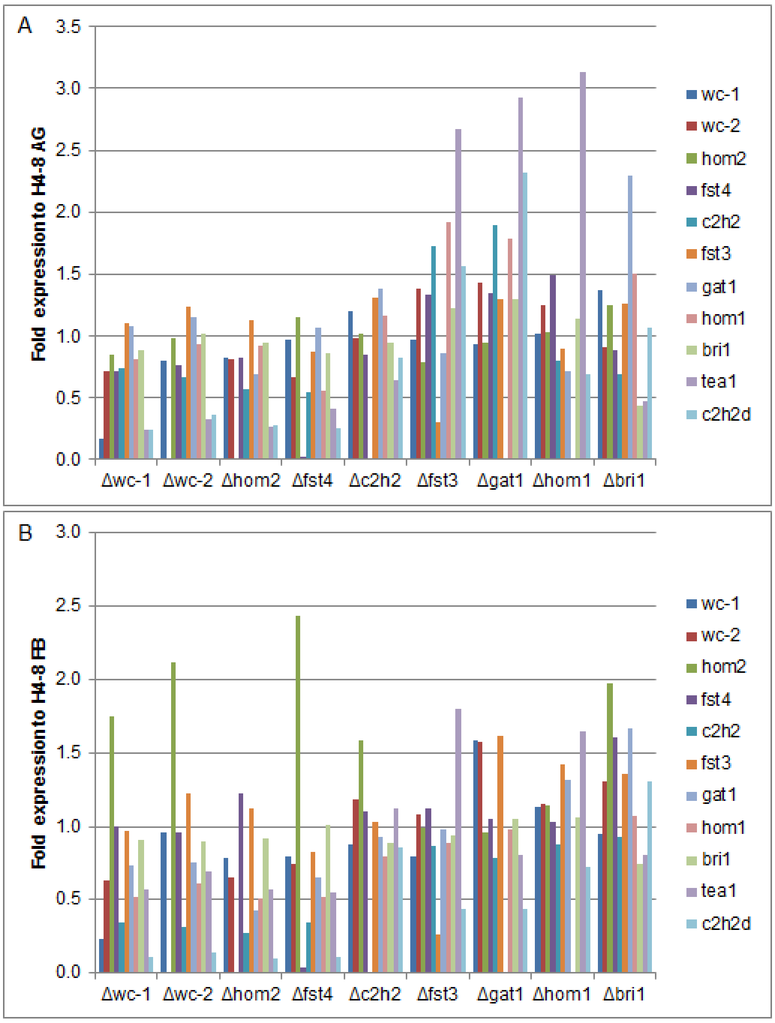

Supplementary Figure 5. Expression analysis of transcription factor deletion strains of *S. commune.* Expression of *wc-1*, *wc-2*, *hom2*, *fst4*, *c2h2*, *fst3*, *gat1*, *hom1*, *bri1*, *tea1,* and *c2h2d* in dikaryotic transcription factor deletion strains when compared to the wild-type during aggregation (A) and fruiting body formation (B). Analysis is based on biological duplicates.
